# Multi-Label Random Forest Model for Tuberculosis Drug Resistance Classification and Mutation Ranking

**DOI:** 10.3389/fmicb.2020.00667

**Published:** 2020-04-22

**Authors:** Samaneh Kouchaki, Yang Yang, Alexander Lachapelle, Timothy M. Walker, A. Sarah Walker, CRyPTIC Consortium, Timothy E. A. Peto, Derrick W. Crook, David A. Clifton

**Affiliations:** ^1^Department of Engineering Science, Institute of Biomedical Engineering, University of Oxford, Oxford, United Kingdom; ^2^Oxford-Suzhou Centre for Advanced Research, Suzhou, China; ^3^Nuffield Department of Medicine, University of Oxford, Oxford, United Kingdom; ^4^National Institute of Health Research Oxford Biomedical Research Centre, John Radcliffe Hospital, Oxford, United Kingdom; ^5^Oxford University Clinical Research Unit, Ho Chi Minh City, Vietnam; ^6^NIHR Biomedical Research Centre, Oxford, United Kingdom

**Keywords:** drug resistance, mutation ranking, MLRF, SLRF, tuberculosis

## Abstract

Resistance prediction and mutation ranking are important tasks in the analysis of Tuberculosis sequence data. Due to standard regimens for the use of first-line antibiotics, resistance co-occurrence, in which samples are resistant to multiple drugs, is common. Analysing all drugs simultaneously should therefore enable patterns reflecting resistance co-occurrence to be exploited for resistance prediction. Here, multi-label random forest (MLRF) models are compared with single-label random forest (SLRF) for both predicting phenotypic resistance from whole genome sequences and identifying important mutations for better prediction of four first-line drugs in a dataset of 13402 *Mycobacterium tuberculosis* isolates. Results confirmed that MLRFs can improve performance compared to conventional clinical methods (by 18.10%) and SLRFs (by 0.91%). In addition, we identified a list of candidate mutations that are important for resistance prediction or that are related to resistance co-occurrence. Moreover, we found that retraining our analysis to a subset of top-ranked mutations was sufficient to achieve satisfactory performance. The source code can be found at http://www.robots.ox.ac.uk/~davidc/code.php.

## 1. Introduction

As reported by the World Health Organization, resistance co-occurrence is very common, and is especially so between first-line drugs for treating tuberculosis (TB): isoniazid (INH), ethambutol (EMB), rifampicin (RIF), and pyrazinamide (PZA) (World Health Organization, 2017). Two types of resistance co-occurrence are especially important: (i) multi-drug resistant TB (MDR-TB) defined as cases that are resistant to at least INH and RIF; and (ii) extensively drug-resistant TB (XDR-TB), defined as isolates that are resistant to INH and RIF plus any of the fluoroquinolones such as levofloxacin or moxifloxacin and at least one of the three injectable second-line drugs, including amikacin, capreomycin, or kanamycin. Hence, resistance co-occurrence to anti-TB drugs has become an urgent public health concern (World Health Organization, 2017).

Conventional methods for resistance prediction from whole genome sequences are usually based on identifying specific known resistance-conferring variants (i.e., single nucleotide polymorphisms; insertions or deletions) and interpreting (i) the presence of any of them as indicating resistance; and (ii) the absence of all of them as indicating susceptibility to an individual drug (Schleusener et al., 2017). Most techniques are based on a library of resistance-conferring variants for each individual drug (Georghiou et al., 2012; Coll et al., 2015; Walker et al., 2015). However, due to high dimensionality of the sequencing data and unknown resistance mechanisms, these techniques do not necessarily result in high classification performance especially for less-studied drugs. Moreover, such methods predict resistance drug-by-drug based on known mutations for each drug, rather than by jointly predicting MDR- or XDR-TB.

Some mutations are commonly seen in strains that are resistant to multiple drugs (e.g., MDR-TB and XDR-TB isolates). This is likely to be because they have no, or very limited, fitness cost (Eldholm et al., 2015). This suggests that predicting the global phenotype (e.g., MDR-TB), rather than individually predicted phenotypes (e.g., resistance to INH), could be a promising approach. *katG*_315 was the most common MDR-TB mutation in a dataset of 608 susceptible and 403 MDR-TB isolates in Hazbón et al. (2006) and also a recent study of 5310 isolates (Manson et al., 2017). Moreover, the proportion of isolates with *katG*_315 mutations was higher in MDR-TB isolates than mono-resistant isolates, supporting the hypothesis that these strains have a lower fitness cost and are better able to acquire and tolerate additional mutations. Similarly, *katG*_315, *rpoB*_445, and *rpoB*_450 mutations were found to be associated with MDR-TB isolates in another study (Van Rie et al., 2001) which identified 90% of all MDR-TB in their 5-year dataset. Borrell et al. (2013) observed that the *gyrA*_D94G mutation was associated with greater fitness than the *gyrA*_G88C mutation when co-existing with *rpoB* mutations in strains that are resistant to both RIF and quinolones. The later points to a likely epistatic interaction between *gyrA*_D94G and *rpoB*.

Multi-label learning provides a potential solution to such challenges. Multi-label learning is an important classification technique if each sample in a dataset is associated with multiple labels (e.g., resistance/susceptibility to multiple drugs) and if there are correlations between labels (e.g., for resistance co-occurrence, there are around 2,000 isolates that are resistant to both INH and RIF). In this case, learning each label independently, ignoring correlations between labels, results in lower performance. Instead of considering resistance to each drug individually, the multi-label technique learns a single model for all drugs, and makes a prediction at the sample level. This method is closer to the clinical reality, where drug resistance phenotypes are not typically independent of one another due to using regimens made up of a combination of drugs. Resistance co-occurrence is especially common in first-line drugs, since standard regimens require them to be used together. Existing machine learning methods for TB prediction in the literature have focused on single-drug prediction (Periwal et al., 2011; Zhang et al., 2013; Farhat et al., 2016; Yang et al., 2018; Deelder et al., 2019), and ignored epistasis and correlation of resistance between drugs. Building a multi-label model to account for both of the latter may improve predictive performance and be useful for extracting important MDR- or XDR-TB resistance-associated mutations. In the context of this study, we compared multi-label random forests (MLRFs) with single-label random forests (SLRFs) for the prediction of phenotypic TB resistance. Analysing drugs with high resistance co-occurrence (e.g., RIF and INH) simultaneously should enable patterns reflecting resistance co-occurrence to be exploited for resistance prediction. MLRF and SLRF models, on the other hand, would perform closely for drugs that the resistance co-occurrence is less common. We also conduct feature analysis for mutation ranking. We trained our models on a database of 13402 isolates with resistance phenotypes for up to 11 first- and second-line anti-TB drugs (INH, EMB, PZA, RIF, streptomycin, amikacin, moxifloxacin, fluoroquinolones-ofloxacin, kanamycin, capreomycin, ciprofloxacin). Resistance/susceptibility to all first-line drugs individually, MDR-TB, and cases with resistance to the four first-line drugs (denoted FDR-TB) were considered as labels (i.e., classification “ground truth”) for the analysis. There were few XDR-TB cases (245 isolates) in our dataset due to the high percentage of missing labels, hence XDR-TB was not considered in our study. MLRF predicts labels for all considered drugs simultaneously and also can rank all associated mutations that are important in drug resistance prediction. Such analysis can also help to find mutations associated with resistance co-occurrence. In a substudy, the models were retrained (and the classification performance was recalculated) on a subset of ranked features instead of using all available features; this substudy allows us to evaluate the influence of selected highly-ranked features on the classification performance (as might be useful in creating a lightweight system for use in real-time, in practice).

In summary, to date, RF-based studies for drug resistance prediction have only considered each drug individually (Farhat et al., 2016; Kouchaki et al., 2019). However, greater power may be obtained with RFs through multi-label analysis incorporating information from all drugs to include the co-occurrence of drug resistance and epistasis. Being an ensemble method, the MLRF also has advantages considering that there are fewer resistant examples available than susceptible isolates (i.e., datasets are highly imbalanced) that are common in the study of TB genomics. We focus on comparing MLRFs and SLRFs in terms of classification performance, mutation ranking, and the effect of feature selection on the performance.

## 2. Materials and Methods

We studied a diverse and large dataset collected from 16 countries across six continents.

### 2.1. Whole Genome Sequencing

Details of DNA sequencing and our data source (including the European Nucleotide Archive/Sequence Read Archive accession numbers) are presented in Walker et al. (2015) and CRyPTIC Consortium and the 100,000 Genomes Project (2018) and Supplementary I. Sequenced reads were aligned to the reference MTB strain, and nucleotide bases were filtered based on the sequencing and alignment quality, and per-base coverage. Low confidence nucleotide bases were denoted as null calls. There are several ways to treat a null call in an isolate: (i) removing the sample completely from the analysis, which greatly reduces the sample size (since 34% of isolates have one or more null calls in the genetic regions of interest) and generalisability; (ii) considering the null calls as no variants (i.e., mutation presence = 0), which is a conservative option and means that performance will be an underestimate of true performance if all variants were known; (iii) considering null values as missing and impute their values, on either a single or multiple basis. We chose the second option (assuming absence of variant) because the total number of variant positions across the genetic regions of interest (5919 positions) and across all isolates (13402) with null calls was very small (0.19%); and because of the complexity of multiple imputation models that would be needed for (iii), based on the 5919 positions. This approach is effectively a “single” hard (i.e., conservative) imputation.

### 2.2. Data Description

The dataset used in this paper contains 13402 isolates collected from across the world. In this study, we followed previous work in which 23 genes (Table S1) were targeted containing known resistance-associated mutations (Walker et al., 2015). For each isolate, the presence/absence of a variant was represented by a binary variable, with 1 indicating presence and 0 indicating absence. Across the 23 candidate genes, in total, 5919 variants were found across isolates, including multiple variants at the same position. The mean number of variants per isolate was 14, ranging between 1 and 132. Hence, a binary vector of length 5919 was formed for each isolate, and considered to be our feature space (i.e., set of input variables). For each drug and isolate, a binary label of resistance/susceptible was considered. The “ground truth” phenotypic information was available for up to 11 anti-TB drugs using culture and confirmed selective culturing on Lowenstein-Jensen media. Not all samples were tested against all drugs with missing values, especially for second-line drugs where missingness of the phenotypical label was substantial. There were only a few XDR-TB cases (245 isolates) in our dataset due to the high percentage of missing labels and hence XDR-TB was not considered in our study.

**Figure 1:**
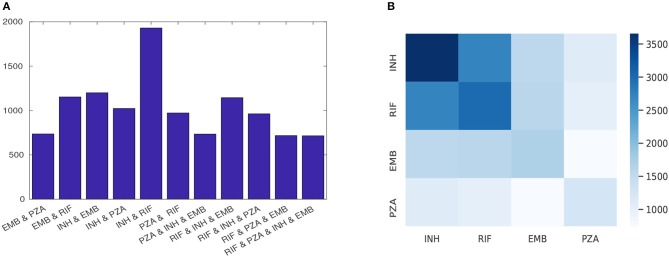
The phenotypic profile of first-line drugs; **(A)** each row shows the number of isolates that are resistant to at least the indicated drugs combination and **(B)** heatmap quantifying the number of instances of resistance co-occurrence between drugs. Off-diagonal elements show resistance co-occurrence between different drugs and diagonal elements show resistant to a single drug.

For the four first-line drugs, more isolates were susceptible than were resistant. For example, more than 88% of isolates tested for EMB and PZA and 75% for INH and RIF were susceptible. Moreover, there were several isolates with multiple drug resistance considering the four first-line drugs (Figure 1).

### 2.3. Predicting TB Drug Resistance From Sequence

Existing methods predominantly classify drug resistance as present or absent based on a library of predetermined variants from the literature. These methods, here denoted direct association (DA), use a logical “OR” rule to classify an isolate against a given drug: the isolate is labeled as resistant if any of its mutations is a previously-known resistant variant. Otherwise, it is classified as susceptible (i.e., if only susceptible variants exist in the isolate). The library described by Walker et al. (2015) was used throughout the classification comparison here.

### 2.4. MLRF for TB Classification

The RF is an ensemble method that is based on building several independent decision tree classifiers on different subsets of the dataset. It considers the combination (often the average) of the output of each independent classifier to improve performance in producing overall predictions.

Multi-label learning is a supervised problem in which several labels are learned simultaneously. In the TB data, there are many cases of MDR-TB, as shown in Figure 1 (World Health Organization, 2017). Using multi-labels (i.e., all phenotypes simultaneously, rather than considering each independently) can reduce the training time as only one model is learned, and predictive performance can be increased (Evgeniou and Pontil, 2004) due to learning correlation between inputs and the multiple outputs. The RF model can be extended to learn and predict multiple drugs simultaneously considering a joint score (Gini index) across all considered drugs (Faddoul et al., 2012). Specifically in each decision tree, for each pair (*f*, *x*) of a feature *f* (mutation) and a value *x* (isolate) with a label *y* (resistance phenotype) at node (*t*):

(1) $$\begin{array}{l} \text{Gini index, }{\text{GI}}_{J}\left(t,f,x\right)=\underset{y\in Y}{\sum }{\text{GI}}_{y}\left(t,f,x\right) \end{array}$$

where *Y* is the number of labels (two for MDR-TB and four for FDR-TB) and GI_*J*_ and GI_*y*_ are the joint and per-label Gini indices, respectively. The objective is to minimize Equation (1) and hence (*f, x*) is selected to best separate (defined by a lower joint Gini index) the data at each node in the tree. Hence, during training, it can compute the importance of each feature by averaging the impurity decrease associated with each mutation.

Figure S1 shows a sample decision tree from a forest learned by MLRF for the four first-line drugs (EMB, INH, RIF, and PZA). In comparison, a tree learned by SLRF for EMB is shown in Figure S2. The tree grows in the best-node-first fashion (defined by impurity reduction^1^).

*katG*_S315T, *rpoB*_S450L, *embB*_M306V, *embB*_Q497R, and *embB*_M306I are common mutations in both trees. *katG*_S315T was the most highly-ranked feature in both trees, but other rankings of features vary between models. A feature (mutation) that results in the lowest Gini index is selected to best split the data at each node. The MLRF learns a joint Gini index (Equation 1), and hence finds that feature that best splits the data considering all drugs. In contrast, the SLRF only considers the Gini index based on one drug at each node (e.g., EMB). After the node split, another feature is selected that further reduces the Gini index. Building various trees on different subsets of the data can then automatically pick important features. Consequently, MLRF ranks mutations to best classify resistance to *all* drugs. Hence, it also helps the model learn mutations associated with resistance co-occurrence. Conversely, SLRF ranks mutations to best classify an *individual* drug ignoring any co-occurrence. The SLRF also ranks some mutations from *other* drugs as being important as seen in Supplementary B, which effectively reflects underlying interaction between phenotypes. After building the models, samples traverse each tree by starting at the root node, reaching a leaf node. The classification is calculated at the leaf node by majority vote and the final classification is obtained by averaging results across trees.

### 2.5. Multi-Label Stratification

Stratified sampling (i.e., taking an equal proportion from each class) is especially important in TB analysis due to the imbalanced nature of the data and the co-occurrence of drug resistance for different drugs, with some resistance patterns being much rarer than others (Table S4). Hence, an iterative algorithm termed multi-label stratified cross-validation (Sechidis et al., 2011) was considered here to avoid the use of subsets without any examples of rare labels. Multi-label stratified cross-validation starts with a label combination that has the fewest samples. Considering rare label combinations before more frequent combinations increases the chance of distributing these rare examples evenly among prediction of the data between training and test sets. In each iteration, one sample from the most rare combination is selected and added to a partition depending on the number of samples with that label already in each partition. Then, the partitioning continues with another sample with the same label if any remain; otherwise, a sample from the second-most rare label combination is considered. This process continues until all samples are assigned to a subset.

### 2.6. Feature Spaces

To evaluate the performance of our model and to obtain feature rankings, five feature sets were considered: [F1] the baseline feature space of all variants found within 23 candidate genes (*N* = 5,918); [F2] as a subset of feature set F1 includes only drug-associated genes for a particular drug (*N* = 3,366 that obtained by only considering the variants within the genes that are known to be associated with the first line drugs, Supplementary A); [F3] known variants from (Walker et al., 2015) for all first-line drugs (*N* = 1874); [F4] and [F5] are obtained by dropping isolates with any known resistance-associated mutations from feature sets F1 and F2, respectively — that is, feature sets F4 and F5 allow us to investigate whether phenotypically resistant isolates without well-known resistance mutations can be identified from other sequence variations (*N* = 4,755 and 2,417, respectively). Feature set F1 includes all variants spaces, which is preferable for less-studied drugs. For well-studied drugs, using the known catalog of resistance-associated mutations has been shown to perform well.

### 2.7. Training and Testing

For all experiments, model construction and evaluation was performed over 10 iterations of five-fold multi-label stratified cross-validation. In each iteration, 20% of the dataset was used as the test set and the remaining 80% of the data as the training set. Here, the “internal” cross-validation on the 80% training dataset was used to select a decision threshold that maximizes the accuracy; this threshold was then used for prediction in the test set. Moreover, we considered fixed RF hyper-parameters for both techniques (50 estimators with maximum depth of two and maximum features as the square root of input variants). The performance in terms of accuracy, sensitivity, specificity, and area-under-the-ROC-curve (AUC) was calculated for the test set (for reporting final “hold-out” results).

(2) $$\begin{array}{l} \text{Accuracy}=\frac{\text{TP}+\text{TN}}{\text{TP}+\text{TN}+\text{FP}+\text{FN}}\\ \text{Sensitivity}=\frac{\text{TP}}{\text{TP + FN}},\text{ Specificity}=\frac{\text{TN}}{\text{TN}+\text{FP}}. \end{array}$$

where TP, TN, FP, and FN are true positive, true negative, false positive, and false negative, respectively, and where P and N are resistance and susceptible samples, respectively. The output of the models is a probability estimate *P*(*C*_1_|**X**) of the posterior probability of input feature vector **X** belonging to class *C*_1_ (resistant). We then define a threshold *k* on *P*(*C*_1_|**X**), such that a classification of **X** ↦ *C*_1_ (i.e., resistant) is made if *P* > *k*, and a classification of **X** ↦ *C*_0_ (i.e., susceptible) if *P* ≤ *k*. Varying threshold *k* results in different TP, FP, FN, and TN rates and thus sensitivity and specificity vary according to the value of *k* ∈ [0, 1]. However, AUC is calculated over all value of *k*, and is therefore insensitive to any particular choice of decision threshold *k*. The workflow of examined classifiers can be seen in Supplementary C.

## 3. Results

### 3.1. Comparison of Top Performing Classifier and DA

Table 1 compares the performance of DA and the best performing model considering feature sets F1-F5 for INH, EMB, RIF, PZA, MDR-TB, and FDR-TB. Our results show that the MLRF is the best performing model for all drugs except for PZA. feature set F3 was the best feature set for INH, RIF, and MDR-TB, while feature F1 was the best feature set for EMB, PZA, and FDR-TB all in terms of AUC. DA showed higher specificity in comparison with the best performing model, but had lower sensitivity and AUC in all cases.

**Table 1:** Performance of the best machine learning classifier and DA considering INH, EMB, RIF, PZA, MDR-TB, and FDR-TB.

	**DA**			**Best method**			
**Drugs**	**Sensitivity**	**Specificity**	**AUC**	**Feature set + Classifier**	**Sensitivity**	**Specificity**	**AUC**
INH	91.15 ± 1.19	98.96 ± 0.25	95.05 ± 0.60	F3 + MLRF	93.76^*^ ± 0.80	97.79 ± 0.35	96.01^*^ ± 0.47
EMB	85.10 ± 1.79	94.91 ± 0.38	90.00 ± 0.97	F1 + MLRF	91.75^*^ ± 1.81	91.58^*^ ± 0.77	91.70^*^ ± 0.75
RIF	91.52 ± 1.34	98.68 ± 0.21	95.10 ± 0.65	F3 + MLRF	93.16^*^ ± 0.80	98.02 ± 0.32	96.00^*^ ± 0.40
PZA	43.21 ± 2.72	98.58 ± 0.23	70.89 ± 1.35	F1 + SLRF	87.27^*^ ± 1.74	90.71^*^ ± 0.72	88.99^*^ ± 0.84
FDR-TB	37.34 ± 3.97	98.59 ± 0.22	67.96 ± 1.99	F1 + MLRF	87.58^*^ ± 2.79	92.98^*^ ± 0.45	90.28^*^ ± 1.23
MDR-TB	89.84 ± 1.34	99.12 ± 0.178	94.48 ± 0.69	F3 + MLRF	93.70^*^ ± 0.76	97.45 ± 0.36	95.58^*^ ± 0.41

Sensitivity, specificity and AUC (mean ± standard error) were reported. The Wilcoxon signed-rank test was used to calculate the p-value of each method compared with the DA and

* *p < 0.01 vs. DA*.

### 3.2. Detailed Comparison of MLRF, SLRF, and DA

Supplementary D provides further details of the classification results. In terms of classification performance, both SLRF and MLRF perform fairly similarly with slight improvements in AUC and sensitivity considering MLRF especially for INH and RIF (*p* < 0.01). Compared to DA, sensitivity increased for all drugs (considering feature sets F1 and F3) and for all drugs except RIF when considering feature set F2. Both MLRF and SLRF had higher AUC than DA considering feature sets F1-F3 for EMB, considering feature set F3 for INH and RIF, considering feature sets F1 and F3 for MDR-TB and considering all feature sets for PZA and FDR-TB.

### 3.3. Mutation Ranking

The 10 most important mutations based on MLRF and SLRF and feature sets F1-F5 is shown in Supplementary E. In summary:

- There were several known mutations that were commonly ranked as being important for the purpose of prediction, regardless of model (MLRF and SLRF) and drug: (i) *katG*_S315T, *rpoB*_S450L and *embB*_M306V for feature set F1; and (ii) the latter three mutations along with *embB*_M306I for feature sets F2-F3. These are the most common known resistance mutations associated with INH, RIF, and EMB, respectively (Walker et al., 2015). However, each of these highly-related mutations had different importance values and resulted in different classification performance across various MLRFs and SLRFs trained on different feature sets.
- Analysis using feature set F4 identified several important mutations from other genes related to second-line drugs (e.g., *rrs*_G349A and *eis*_C-12T).
- There was considerable overlap between mutations ranked for all first-line drugs and FDR-TB. In other words, SLRF ranking for a given drug indicated multiple mutations that are associated with *other* drugs.
- Several mutations selected as being important were not in the DA library and were not lineage defining. Some of these mutations occurred within genes associated with a given first-line drug. Detailed information of their occurrence in isolates is shown in Supplementary F.
- Considering (i) feature set F1, (ii) all variants in drug-associated genes for a given drug from feature set F2, and (iii) known drug-resistant variants for a given drug extracted from feature set F3, resulted in identifying a list of candidate mutations that are important for resistance prediction or are related to resistance co-occurrence (Supplementary G).

### 3.4. MLRF and SLRF Performance on a Subset of Important Features

**Figure 2:**
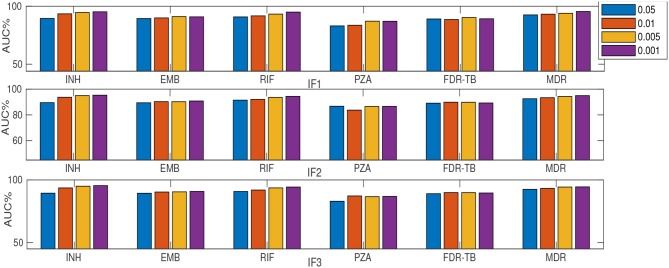
AUC (%) comparison considering MLRF and four thresholds {0.05, 0.01, 0.005, and 0.001} for feature selection. “I” prefix refers to our substudy.

**Figure 3:**
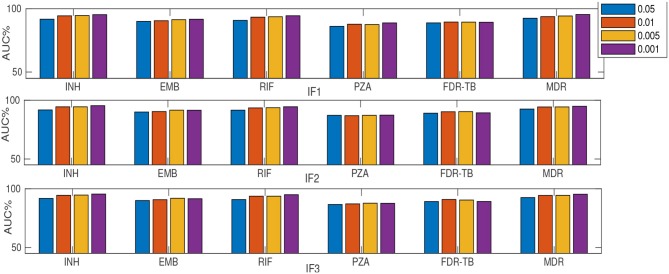
AUC (%) comparison considering SLRF and four thresholds {0.05, 0.01, 0.005, and 0.001} for feature selection. “I” prefix refers to our substudy.

As described earlier, a substudy introduced retraining models on a subset of ranked features (instead of using feature sets F1-F5). Table 2 and Figures 2, 3 summarize the performance of the different classifiers when the feature set is restricted to that subset of mutations in feature sets F1-F3 ranked above importance thresholds of {0.05, 0.01, 0.005, and 0.001} (details in Supplementary E). In summary:

- The best model for each drug (Table 2) still performs better than DA even when using a subset of important mutations (16–37 mutations) for INH, EMB, PZA, MDR-TB, and FDR-TB in terms of AUC and sensitivity (*p* < 0.01).
- Considering only 16–37 features rather than the larger feature sets F1-F5 resulted in better performance for EMB and FDR-TB and very similar performance for others (Table 2).
- The SLRF performed better for EMB, PZA, and FDR-TB when restricted to using highly-related mutations in this way.
- Increasing the number of features (i.e., decreasing the threshold on feature importance used to select features in this substudy) did not always improve the performance (e.g., FDR-TB).
- Increasing the number of features usually increased sensitivity while reducing specificity.

**Table 2:** Performance of best models restricting to only important mutations for classification.

**Drug**	**INH**	**EMB**	**RIF**	**PZA**	**FDR-TB**	**MDR-TB**
Best model	IF3 (0.001) + MLRF	IF3 (0.005) + SLRF	IF3 (0.001) + MLRF	IF1 (0.001) + SLRF	IF3 (0.01) + SLRF	IF3 (0.001) + MLRF
Number of mutations	37	17	37	32	16	37
Sensitivity	92.88 (↓0.28) ± 0.93	91.10 (↓0.65) ± 1.76	92.19 (↓0.07) ± 1.10	84.73 (↓2.54) ± 2.49	91.74 (↑4.16) ± 3.37	93.76 (↑0.06) ± 1.33
Specificity	97.88 (↑0.09) ± 0.31	92.70 (↑1.12) ± 0.51	97.77 (↓0.22) ± 0.52	92.83 (↓2.12) ± 0.52	90.06 (↓2.92) ± 0.61	97.38 (↓0.07) ± 0.49
AUC	95.48 (↓0.53) ± 0.40	91.90 (↑0.20) ± 0.82	94.98 (↓1.02) ± 0.53	88.78 (↓0.21) ± 1.17	90.90 (↑0.62) ± 1.56	95.47 (↓0.11) ± 0.62

*The number of mutations used for the classification, best model and performance for INH, EMB, RIF, PZA, MDR-TB, and FDR-TB are shown. Increase/decrease in performance in comparison with the best model in Table 1 are indicated with up/down arrows, respectively. “I” prefix refers to our substudy*.

## 4. Discussion

Our analysis demonstrates that machine learning methods, specifically MLRF (considering feature sets F1-F3), had higher sensitivity but lower specificity compared with DA (at their points of higher accuracy). Sensitivity and AUC increased substantially for PZA and FDR-TB when using MLRFs. There may be several reasons for this finding, including (i) the existence of additional resistance-associated mutations to those reported in the literature; (ii) the existence of certain combinational patterns of resistance-related and epistasis and lineage-related mutations; and (iii) co-occurrence of resistance (for the 23 genes considered in this paper). Supplementary G provides a list of possible candidates for (i) and (iii). Lower specificity could be due to the existence of several isolates with resistance-associated mutations that were incorrectly labeled as susceptible. It could be because of limitations in the routine phenotyping relating to dichotomous thresholds of “resistant” vs. “susceptible” applied to a continuous measure of the minimum inhibitory concentration, as is well-known for M306V for example (Khan et al., 2019). This could also have some additional negative effects on prediction of co-occurring resistance. Another reason could be the threshold setting for obtaining sensitivity and specificity. There is a trade-off between sensitivity and specificity in which increasing one can result in decreasing the other. The use of feature sets F4 and F5 resulted in lower prediction performance then other feature sets mainly because of very low numbers of resistant isolates left after dropping those with known resistant-associated mutations (Supplementary H).

The best method based on MLRF had only slightly higher sensitivity and AUC compared to SLRF for most drugs (Supplementary D), possibly because of several common MDR-TB mutations, i.e. *katG*_315 being a strong resistance-conferring variant, as in Hazbón et al. (2006). As the feature space is the same for MLRF and SLRF models, both techniques can take advantage of using the occurrence of mutations that is more likely to occur in multi-drug resistant samples. However, learning one model for all labels as in MLRF makes better use of such mutations as it learns all drugs simultaneously. Consequently, MLRF also enhances performance for single drugs by using existing resistance co-occurrence. PZA was a notable exception, potentially due to the existence of many less strong variants related to PZA resistance. Another reason for the very close AUC between MLRF and SLRF could be that we fixed the RF hyper-parameters (number of decision trees, maximum number of variant for each decision tree,…) for both techniques. Future work introducing a separate parameter optimization could possibly increase the difference in performance.

Our results confirmed the importance of several known mutations with resistance co-occurrence (e.g., *katG*_S315T, *rpoB*_S450L, and *embB*_M306V). Feature set F3 was the best feature set for well-studied drugs (INH, RIF, and MDR-TB) but feature F1 was better for others. This shows that there are additional mutations that are *not* within the current library of known mutations (used for DA) but which are important in classifying resistance; additional co-occurrence patterns of mutations may exist, as might weak interactions between mutations that may have joint effects. Classification based on MLRF and feature sets F1 and F2 mainly identified known resistance-associated mutations as being important. This builds confidence in our approach. However, after removing isolates with any known variants, several mutations were ranked as being important (i) from other genes (e.g., related to second-line drugs); (ii) from known lineage-defining variants; and (iii) that were not in the library and were not lineage-defining (by checking if they occurred in more than one lineage, Supplementary F, G). Our results thereby confirm the possibility of additional important mutations (for prediction) to those already known to be important for TB resistance classification. We note that the tree depth was not limited for the learning procedure. Consequently, as we go deeper in the trees learned based on feature set F1, all other features can be seen. However, in TB there are a few strong mutations with high importance values (e.g., *katG*_S350L) which result in very low importance values for other mutations. Removing the impact of such highly important mutations as in feature sets F4 and F5 would allow investigation of whether or not phenotypically resistant isolates without well-known resistance mutations can be identified from other sequence variations. In other words, although a deeper tree can see wider spectrum of mutations, feature sets F4 and F5 can zoom in other sequence variations by avoiding the impact of highly important mutations.

Considering only the top-ranked mutations (as in our substudy) resulted in higher AUC compared to DA for all drugs except RIF (Table 2). Thus a small number (16-37) of important features are generally sufficient for RF-based classification. Similar to considering the whole feature set, IF1 and IF3 outperformed IF2, IF4 and IF5 (where “I” prefix refers to our substudy). However, the MLRF only performed better then the SLRF for INH, RIF, and MDR-TB. Considering IF1-IF5, the SLRF was trained on important mutations for each drug and not on the highest-ranking mutations based on MLRF. Hence, different feature sets were used for SLRF and MLRF training. SLRF based on only important features for PZA, FDR-TB, and EMB had better performance compared with the common features based on MLRF. The MLRF was better for INH, RIF, and MDR-TB, possibly because the variants related to these drugs were stronger predictors, while those of PZA and FDR-TB reflect a potential combination effect between variants that are individually weak prediction of resistance. That is, the pattern of resistance for INH, RIF, and MDR-TB dominates the multi label learning, while the other can be captured by the SLRF. Moreover, errors in routine phenotypes of individual drugs impact MLRF more than SLRF. One limitation of the SLRF model is that it ranked highly many weak variants that are lineage-related mutations (Supplementary D). We need to note that lineage defining mutations might be helpful where resistance is over-represented in one lineage (e.g., MDR-TB in lineage 2). Figure 2 demonstrates that increasing the number of features by reducing the feature selection threshold usually increases AUC, but this is not always the case; e.g., IF1 and IF2 for FDR-TB (Figure 2). Consequently, our results indicate the importance of feature ranking to reduce the effect of unrelated mutations in the learning process. Another important conclusion of our work is that by increasing the number of features used, sensitivity improved at the expense of related specificity, confirming that a smaller feature set better predicts susceptible samples while there is a need to have more features to better predict resistant samples (Supplementary E). A trade-off typically exists between sensitivity and specificity.

We note there are several limitations regarding our analysis. An assumption of feature ranking should be that the input features are independent; if there are some highly correlated features, any of them could be selected as an important feature. In other words, machine learning techniques, including RF, aim to identify patterns in the data that contribute to predictions. After selecting one such feature, the importance of other correlated features is decreased considering the classification performance. From a classification point of view, it is actually useful to do this as it removes the features whose effect is already described by other closely-related features. Hence, SLRF and MLRF are typically based on correlation and not causation, which means that lineage associate mutations, in addition to mutations conferring resistance to other drugs, can be used in the learning. However, ranking such mutations as important is a limitation of existing machine learning techniques in general. This mainly impacts performance in local settings, where the level of resistance co-occurrence between first- and second-line drugs is different, or where such mutations are completely absent or very abundant. Considering population level structure and cluster effect in the learning will be considered as a future work. For feature selection, an additional step might be helpful to indicate the correlated variants. Such effects can be decreased by random selection of features but they cannot be removed completely.

Random selection may also affect the selection of rare but important mutations. We note that the dataset in our application, which reflects the imbalance encountered in clinical practice, with (for example) a high percentage of samples resistant to INH + RIF that can bias feature ranking in favor of those more common labels. Finally, other limitations include any errors in phenotypes that may exist; considering equal importance for all mutations; and ignoring data with missing labels.

## 5. Conclusion

MLRF and SLRF classifiers were investigated for TB resistance classification and mutation ranking considering different subsets of extracted variants. Several common mutations were identified as important which could confirm the existence of several MDR- and FDR-TB associated patterns. Furthermore, restricting analysis to the 16–37 top-ranked mutations might be useful in creating a lightweight system for use in practice. The main advantage of machine learning methods, especially in our application with a large number of features, is hence capturing any association between the feature space and the prediction of resistance, in addition to learning potentially new mutations associated with MDR-TB and FDR-TB (rather than simply predicting resistance to independent drugs).

## Data Availability Statement

The datasets analyzed for this study can be found in the Walker et al. (2015) and CRyPTIC Consortium and the 100,000 Genomes Project (2018).

## Author Contributions

DWC, SK, TP, AW, TW, YY, and DAC contributed toward study design. SK, YY, and DAC contributed toward data analysis. SK wrote the manuscript with comments from YY, AW, TW, and DAC. All authors contributed feedback on the manuscript.

## Members of the CRyPTIC Consortium

Derrick W. Crook, Timothy E. A. Peto, A. Sarah Walker, Sarah J. Hoosdally, Ana L. Gibertoni Cruz, Joshua Carter, Clara Grazian, Sarah G. Earle, Samaneh Kouchaki, Alexander Lachapelle, Yang Yang, David A. Clifton and Philip W. Fowler, University of Oxford; Zamin Iqbal, Martin Hunt and Jeffrey Knaggs, European Bioinformatics Institute; E. Grace Smith, Priti Rathod, Lisa Jarrett and Daniela Matias, Public Health England, Birmingham; Daniela M. Cirillo, Emanuele Borroni, Simone Battaglia, Arash Ghodousi, Andrea Spitaleri and Andrea Cabibbe, Emerging Bacterial Pathogens Unit, IRCCS San Raffaele Scientific Institute, Milan; Sabira Tahseen, National Tuberculosis Control Program Pakistan, Islamabad; Kayzad Nilgiriwala and Sanchi Shah, The Foundation for Medical Research, Mumbai; Camilla Rodrigues, Priti Kambli, Utkarsha Surve and Rukhsar Khot, P. D. Hinduja National Hospital and Medical Research Centre, Mumbai; Stefan Niemann, Thomas A. Kohl and Matthias Merker, Research Center Borstel; Harald Hoffmann, Katharina Todt and Sara Plesnik, Institute of Microbiology & Laboratory Medicine, IML red, Gauting; Nazir Ismail, Shaheed Vally Omar and Lavania Joseph, National Institute for Communicable Diseases, Johannesburg; Guy Thwaites, Thuong Nguyen Thuy Thuong, Nhung Hoang Ngoc, Vijay Srinivasan, and Timothy M. Walker, Oxford University Clinical Research Unit, Ho Chi Minh City; David Moore, Jorge Coronel, and Walter Solano, London School of Hygiene and Tropical Medicine and Universidad Peruana Cayetano Heredia, Lima; George F. Gao, Guangxue He, Yanlin Zhao and Chunfa Liu, China CDC, Beijing; Aijing Ma, Shenzhen Third People's Hospital, Shenzhen; Baoli Zhu, Institute of Microbiology, CAS, Beijing; Ian Laurenson and Pauline Claxton, Scottish Mycobacteria Reference Laboratory, Edinburgh; Anastasia Koch, Robert Wilkinson, University of Cape Town; Ajit Lalvani, Imperial College London; James Posey, CDC Atlanta; Jennifer Gardy, University of British Columbia; Jim Werngren, Public Health Agency of Sweden; Nicholas Paton, National University of Singapore; Ruwen Jou, Mei-Hua Wu, Wan-Hsuan Lin, CDC Taiwan; Lucilaine Ferrazoli, Rosangela Siqueira de Oliveira, Institute Adolfo Lutz, Saulo.

Authors contributing to the CRyPTIC consortium are (in alphabetical order): Irena Arandjelovic, Faculty of Medicine, Institute of Microbiology and Immunology, University of Belgrade, Belgrade, Serbia; Angkana Chaiprasert, Faculty of Medicine Siriraj Hospital, Mahidol University, Thailand; I Comas, Instituto de Biomedicina de Valencia (IBV-CSIC). Calle Jaime Roig, Valencia, Spain; FISABIO Public Health, Valencia, Spain; CIBER in Epidemiology and Public Health, Madrid, Spain; Francis A. Drobniewski, Imperial College, London, United Kingdom; Maha R. Farhat, Harvard Medical School, Boston, MA, United States; Qian Gao, Shanghai Medical College, Fudan University, Shanghai, China; Rick Ong Twee Hee, Saw Swee Hock School of Public Health, National University of Singapore, Singapore; Vitali Sintchenko, Centre for Infectious Diseases and Microbiology–Public Health, University of Sydney, Sydney, NSW, Australia; Philip Supply, Univ. Lille, CNRS, Inserm, CHU Lille, Institut Pasteur de Lille, U1019 - UMR 8204 - CIIL - Centre d'Infection et d'immunité de Lille, Lille, France; and Dick van Soolingen, National Institute for Public Health and the Environment (RIVM), Bilthoven, Netherlands.

## Conflict of Interest

The authors declare that the research was conducted in the absence of any commercial or financial relationships that could be construed as a potential conflict of interest.

## Footnotes

^1^A node split decreases the gini impurity criterion for the two descendent nodes.

## Supplementary Material

Data Sheet 1 **SRA/ENA accession numbers and phenotypes to four first-line drugs**.
