# Supplementary material for: Multi-Label Random Forest Model for Tuberculosis Drug Resistance Classification and Mutation Ranking
Source: Front Microbiol. 2020 Apr 22;11:667. doi: 10.3389/fmicb.2020.00667 (PMC7188832; doi:10.3389/fmicb.2020.00667)
Supplement: Supplementary file 1 [file Data_Sheet_1.pdf]

Accession Numbers, INH, EMB, RIF, PZA
ERR2516654 R R R U
ERR2516675 R R R R
ERR2516705 R R R R
ERR2516592 S S S U
ERR2516463 R R R R
ERR2516649 R R R U
ERR2516632 R R R R
ERR2516690 R R R U
ERR2516664 R R R R
ERR2516576 R R R U
ERR2516599 R R R R
ERR2516458 R R R R
ERR2516706 R R R S
ERR2516443 R R R S
ERR2516573 R R R R
ERR234556 R R R S
ERR108459 S S S S
ERR234564 R S S S
ERR108460 S S S S
ERR108461 S S S S
ERR108462 R R R R
ERR234580 S S S S
ERR108463 S S S S
ERR108514 R R R S
ERR067614 S S S S
ERR067615 R R S R
ERR067616 S S S S
ERR158611 R R R S
ERR067600 R S S S
ERR133798 R R R S
ERR234596 R R R S
ERR108515 R R S S
ERR117449 S S S S
ERR067617 S S S S
ERR067618 R R S R
ERR133799 R S S S
ERR067619 R R R S
ERR234620 R S R R
ERR067620 R R R S
ERR234557 R R R S
ERR067622 R R R S
ERR133800 R R S S
ERR108498 R R R R
ERR108499 R R S S
ERR108500 R R S S
ERR108501 S S S S
ERR108502 R R S S
ERR117469 R S S S
ERR234565 R R S S
ERR234573 R R S S
ERR234581 S S S S
ERR234589 R R R S

ERR234597	R	R	S	S
ERR067608	R	R	S	S
ERR2516525	R	R	R	R
ERR2516560	R	R	R	R
ERR2516405	R	R	R	R
ERR2516488	R	R	S	S
ERR2516608	R	R	R	S
ERR2516618	R	R	S	R
ERR2516464	R	R	S	R
ERR2516472	R	S	R	R
ERR2516687	R	R	S	R
ERR2516734	R	R	R	R
ERR2516424	R	R	R	R
ERR2516414	R	R	R	R
ERR2516697	R	R	S	R
ERR2516534	R	R	R	R
ERR2516645	R	R	R	R
ERR2516647	R	R	R	R
ERR2516418	R	R	R	R
ERR2516434	R	R	R	R
ERR2516500	R	R	R	R
ERR2516631	R	R	R	S
ERR2516509	R	R	R	R
ERR2516732	R	R	R	R
ERR2516646	R	R	R	R
ERR2516610	R	R	R	R
ERR2516547	R	R	R	R
ERR2516422	R	R	R	R
ERR2516572	R	R	R	R
ERR2516698	R	R	R	S
ERR2516503	R	R	R	R
ERR2516557	R	R	R	R
ERR2516425	R	R	R	R
ERR2516430	R	R	S	R
ERR2516709	R	R	R	R
ERR2516467	R	R	S	S
ERR2516404	R	R	R	R
ERR2516412	R	R	R	S
ERR2516490	R	R	R	R
ERR2516591	R	R	R	U
ERR2516427	R	R	R	R
ERR2516717	R	R	S	R
ERR2516669	R	R	R	S
ERR2516554	R	R	S	R
ERR067601	S	S	S	S
ERR133801	R	R	R	S
ERR133802	S	S	S	S
ERR234637	R	R	R	R
ERR067602	R	S	S	R
ERR133803	R	R	S	S
ERR133804	R	S	S	S
ERR133805	R	R	S	R
ERR133806	R	R	S	S

ERR133807	S	S	S	S
ERR133808	S	S	S	S
ERR133809	R	S	S	S
ERR133810	S	S	S	S
ERR133811	S	S	S	S
ERR133812	R	S	S	S
ERR158613	R	R	R	S
ERR133814	R	S	S	S
ERR133815	R	R	S	R
ERR067767	S	S	S	S
ERR067718	R	R	R	S
ERR067719	R	S	R	S
ERR067576	R	R	R	S
ERR067577	R	R	S	S
ERR067578	R	R	S	R
ERR067579	R	R	R	S
ERR067580	S	S	S	S
ERR067581	R	R	R	R
ERR067582	R	S	R	S
ERR234645	R	R	R	S
ERR067721	R	R	R	S
ERR067722	R	R	R	S
ERR234558	S	S	S	R
ERR133818	S	S	S	S
ERR2516512	S	S	S	U
ERR2516456	S	S	S	U
ERR2516530	S	S	S	U
ERR2516600	S	S	S	U
ERR2516635	S	S	S	U
ERR2516585	S	S	S	U
ERR2516677	S	S	S	U
ERR2516710	R	R	R	R
ERR2516548	R	R	R	S
ERR2516552	R	R	S	S
ERR2516673	R	R	R	R
ERR2516486	R	R	S	R
ERR2516502	R	R	R	U
ERR2516662	R	R	R	S
ERR2516595	R	R	R	S
ERR2516602	R	R	S	S
ERR2516429	R	R	R	S
ERR2516721	R	R	R	R
ERR2516616	R	R	R	S
ERR2516612	S	S	S	S
ERR2516650	R	R	R	R
ERR2516540	R	R	S	R
ERR2516514	R	R	R	R
ERR2516693	R	R	R	R
ERR2516704	R	R	R	R
ERR2516564	R	R	S	R
ERR2516699	R	R	R	R
ERR2516605	R	S	S	S
ERR2516528	R	R	R	R

ERR2516541	R	R	R	R
ERR2516485	R	R	R	R
ERR2516598	R	R	R	S
ERR2516497	R	R	S	R
ERR2516433	S	S	S	S
ERR2516420	S	S	S	S
ERR2516637	S	S	S	S
ERR2516581	S	S	S	S
ERR2516683	S	S	S	S
ERR2516436	S	S	S	S
ERR2516611	S	S	S	S
ERR2516507	S	S	S	S
ERR2516518	S	S	S	S
ERR2516691	S	S	S	S
ERR2516587	S	S	S	S
ERR2516688	S	S	S	U
ERR2516469	S	S	S	U
ERR2516701	S	S	S	U
ERR2516428	S	S	S	S
ERR2516511	R	R	R	R
ERR2516731	R	R	S	R
ERR2516408	R	R	R	R
ERR2516625	R	R	R	R
ERR2516462	R	R	R	R
ERR2516724	R	R	R	R
ERR2516708	R	R	R	S
ERR2516538	R	S	S	R
ERR2516517	R	R	S	R
ERR2516523	R	R	R	R
ERR2516685	R	R	R	S
ERR2516437	R	R	S	S
ERR2516409	R	R	R	R
ERR2516441	R	R	R	S
ERR2516558	R	R	R	R
ERR2516570	R	R	R	R
ERR2516630	R	R	R	R
ERR2516658	R	R	R	R
ERR2516451	R	R	R	S
ERR2516682	R	R	R	R
ERR2516665	R	R	R	R
ERR2516702	R	S	R	S
ERR2516648	R	R	R	R
ERR2516624	R	R	R	R
ERR2516716	R	R	R	R
ERR2516681	S	S	S	S
ERR2516623	R	R	R	S
ERR2516452	R	S	S	S
ERR2516532	R	R	R	R
ERR2516738	R	R	R	R
ERR2516678	R	R	R	R
ERR2516496	R	R	R	R
ERR2516666	R	R	R	R
ERR133819	S	S	S	S

ERR067583	R	R	R	S
ERR067584	R	R	R	S
ERR067585	S	S	S	S
ERR067607	R	R	R	R
ERR133820	S	S	S	S
ERR067586	R	S	R	R
ERR133822	S	R	S	R
ERR133823	R	R	R	S
ERR133824	R	R	R	S
ERR133826	R	R	R	S
ERR133827	R	R	R	R
ERR133829	S	S	S	S
ERR133830	S	S	S	S
ERR133831	R	R	R	S
ERR133832	S	S	S	R
ERR133833	S	S	S	S
ERR133834	R	R	S	S
ERR133835	R	S	S	R
ERR133836	R	R	S	S
ERR133837	R	R	S	S
ERR234574	R	R	S	S
ERR234582	S	S	S	S
ERR067723	R	R	S	S
ERR117450	S	S	S	S
ERR133838	R	S	S	S
ERR234590	R	R	R	S
ERR133839	R	R	R	S
ERR133840	S	S	S	S
ERR2516446	S	S	S	S
ERR2516737	S	S	S	S
ERR2516707	S	S	S	U
ERR2516644	R	S	R	R
ERR2516455	R	R	R	R
ERR2516589	R	R	S	S
ERR2516492	R	R	R	R
ERR2516597	R	R	R	R
ERR2516493	R	R	R	R
ERR2516491	R	R	R	R
ERR2516628	R	R	R	R
ERR2516546	R	R	R	R
ERR2516476	R	R	R	S
ERR2516508	R	R	R	R
ERR2516636	R	R	R	R
ERR2516501	R	R	R	R
ERR2516529	R	S	R	S
ERR2516480	R	R	R	R
ERR2516479	R	R	R	S
ERR2516639	S	S	S	S
ERR2516487	S	S	S	S
ERR2516515	S	S	S	S
ERR2516520	S	S	S	S
ERR2516565	S	S	S	S
ERR2516450	S	S	S	S

ERR2516431	R	R	R	R
ERR2516619	R	R	R	R
ERR2516544	R	R	R	R
ERR2516533	R	R	R	R
ERR2516620	S	S	S	S
ERR2516568	R	S	S	S
ERR2516481	S	S	S	S
ERR2516440	R	R	R	R
ERR2516615	R	R	R	S
ERR2516465	R	R	S	R
ERR2516457	R	R	R	R
ERR2516733	R	S	R	R
ERR2516513	R	R	R	R
ERR2516668	R	R	R	R
ERR2516667	R	R	R	R
ERR2516728	R	R	R	R
ERR2516723	R	R	R	R
ERR2516526	R	R	R	S
ERR2516531	R	R	R	R
ERR2516400	R	R	R	R
ERR133841	R	S	S	S
ERR133842	R	R	R	S
ERR067603	R	R	R	R
ERR133844	R	R	R	S
ERR067609	R	R	S	S
ERR067610	R	S	S	S
ERR133846	S	S	S	S
ERR234598	R	R	R	S
ERR234606	R	S	R	S
ERR234614	R	R	R	R
ERR133847	S	S	S	S
ERR067606	S	S	S	S
ERR234622	R	R	R	R
ERR067612	R	S	S	R
ERR067587	R	R	S	S
ERR133849	R	R	S	R
ERR067692	R	R	R	S
ERR067693	S	S	S	S
ERR067694	R	R	S	S
ERR117451	S	S	S	S
ERR133850	S	S	S	S
ERR158616	R	R	R	R
ERR133851	S	S	S	S
ERR234630	R	R	R	R
ERR234638	S	S	S	S
ERR067695	R	R	S	S
ERR133852	S	R	S	S
ERR067696	R	R	S	S
ERR067697	R	R	S	R
ERR067735	R	S	S	S
ERR067736	S	S	S	S
ERR067737	S	S	S	S
ERR067740	S	S	S	S

ERR234646	R	R	S	S
ERR133854	R	R	R	S
ERR067724	R	R	S	R
ERR234559	S	S	S	S
ERR133855	R	R	S	S
ERR133856	R	S	S	S
ERR067624	R	R	S	S
ERR133857	S	S	S	S
ERR2516686	R	R	R	R
ERR2516578	R	R	R	R
ERR2516504	R	R	R	R
ERR133858	R	R	R	S
ERR133859	S	S	S	S
ERR067588	R	R	S	S
ERR067589	S	S	S	S
ERR067590	R	R	R	R
ERR133860	R	R	S	S
ERR067742	R	R	S	S
ERR067743	S	S	S	S
ERR067744	R	R	S	S
ERR067745	R	S	S	S
ERR234575	R	S	S	R
ERR067725	S	S	S	S
ERR067726	S	S	S	S
ERR133862	R	S	S	S
ERR133863	S	S	S	S
ERR133864	S	S	S	S
ERR133865	R	S	S	S
ERR067727	S	S	S	S
ERR067625	R	R	R	S
ERR234599	S	S	S	S
ERR234607	R	R	S	R
ERR067627	R	R	S	R
ERR067748	R	R	S	S
ERR067750	R	R	R	R
ERR067751	S	S	S	R
ERR133866	R	R	S	S
ERR067591	R	R	R	S
ERR067630	R	R	R	S
ERR234623	R	R	S	S
ERR234631	R	R	S	S
ERR067631	R	R	S	S
ERR234639	R	R	R	R
ERR234647	S	S	S	S
ERR234560	R	R	R	S
ERR067753	S	S	S	S
ERR067754	R	R	S	S
ERR133868	R	R	S	S
ERR234576	R	R	R	S
ERR133870	S	S	S	S
ERR067757	S	S	S	S
ERR133871	S	S	S	S
ERR067728	R	R	S	S

ERR067758	R	R	R	S
ERR067759	R	S	S	S
ERR067761	R	R	S	S
ERR067762	R	R	S	S
ERR067592	R	R	S	R
ERR133872	S	S	S	S
ERR133873	R	R	S	R
ERR067594	R	R	R	R
ERR133874	S	S	S	S
ERR234584	R	R	R	R
ERR133875	S	S	R	S
ERR133876	S	S	S	S
ERR133877	S	S	R	S
ERR133878	R	R	R	R
ERR133879	R	R	R	R
ERR133880	R	R	S	R
ERR133881	S	R	S	S
ERR133882	S	R	S	S
ERR133883	S	S	S	S
ERR234600	R	R	R	R
ERR234608	R	R	S	S
ERR067729	S	S	S	S
ERR234616	R	R	S	S
ERR067730	S	S	S	S
ERR067731	S	S	S	S
ERR234624	R	S	S	S
ERR067732	R	S	R	R
ERR133885	R	R	R	S
ERR067763	S	S	S	S
ERR133886	R	R	S	S
ERR067765	R	R	S	S
ERR067733	R	R	S	S
ERR234640	S	S	S	S
ERR234648	R	R	S	S
ERR067604	S	S	S	R
ERR133888	R	R	R	S
ERR133889	R	R	R	S
ERR133890	R	R	S	S
ERR133891	S	S	S	S
ERR133892	R	R	R	U
ERR133893	S	S	S	S
ERR117452	R	R	S	S
ERR234561	S	S	S	R
ERR234569	R	S	S	S
ERR234577	R	S	S	S
ERR133895	R	R	S	S
ERR067595	S	S	S	S
ERR234585	R	S	S	S
ERR133896	R	R	S	S
ERR133897	S	S	S	S
ERR067734	S	S	S	S
ERR234593	R	R	S	S
ERR067613	R	R	S	S

ERR117453	R	R	S	S
ERR067766	S	S	S	S
ERR067596	R	S	S	S
ERR234609	R	R	R	S
ERR133898	S	S	S	S
ERR067599	R	R	R	S
ERR234617	S	S	S	S
ERR234633	R	R	R	S
ERR234641	R	R	S	S
ERR234649	R	R	S	R
ERR234562	R	R	R	S
ERR133899	R	R	R	S
ERR234570	R	R	S	S
ERR234578	S	S	S	S
ERR234586	S	S	S	S
ERR234594	R	R	S	S
ERR234602	U	R	S	S
ERR234610	R	R	S	S
ERR234618	R	R	R	S
ERR133900	R	R	R	R
ERR133901	R	R	R	S
ERR133902	R	R	S	S
ERR133903	R	R	R	R
ERR133904	S	S	S	S
ERR234634	S	S	R	S
ERR133905	S	R	S	S
ERR234642	S	S	S	S
ERR133906	R	R	R	R
ERR133907	R	R	R	S
ERR133908	R	R	R	R
ERR133909	R	R	S	R
ERR133911	R	S	S	S
ERR117454	R	R	S	R
ERR108455	R	R	S	R
ERR133912	S	S	S	S
ERR133913	R	S	S	S
ERR133914	R	R	R	R
ERR108456	S	S	S	S
ERR133915	R	R	S	R
ERR108457	R	S	R	R
ERR133917	R	R	S	S
ERR234650	R	S	R	S
ERR117455	S	S	S	S
ERR133919	R	R	R	S
ERR133920	S	S	S	S
ERR133921	R	R	R	S
ERR133922	R	R	S	S
ERR133924	R	S	S	S
ERR133925	S	S	S	S
ERR133926	S	S	S	S
ERR108467	R	R	R	S
ERR108468	S	S	S	S
ERR108469	R	R	R	S

ERR133929	S	S	S	S
ERR133930	R	R	S	S
ERR133932	S	S	S	S
ERR108470	S	S	S	S
ERR133934	S	S	S	S
ERR133935	R	R	R	S
ERR133937	R	R	S	S
ERR133938	S	S	S	S
ERR133939	S	S	S	S
ERR133941	S	S	S	S
ERR133942	R	R	R	R
ERR133944	R	R	S	S
ERR2516390	R	R	R	U
ERR2516344	R	R	R	U
ERR2516291	R	R	R	R
ERR2516176	R	R	R	U
ERR2516320	R	R	R	U
ERR2516249	R	R	R	U
ERR2516359	R	R	R	U
ERR2516237	R	R	R	U
ERR2516232	R	R	R	U
ERR2516229	R	R	R	U
ERR2516327	R	R	R	S
ERR2516325	R	R	S	S
ERR2516330	R	R	R	S
ERR2516387	R	R	R	R
ERR133945	R	S	S	R
ERR133946	S	R	S	S
ERR067647	R	R	S	S
ERR067649	R	R	S	S
ERR067650	R	R	S	S
ERR067651	R	R	R	S
ERR133947	S	R	S	S
ERR133948	S	S	S	S
ERR133949	S	S	S	S
ERR133950	S	S	S	S
ERR133951	R	R	R	R
ERR133952	S	S	S	S
ERR133953	R	R	R	S
ERR133954	R	R	R	R
ERR067653	R	R	R	S
ERR067654	R	R	R	U
ERR067655	R	R	S	S
ERR067656	R	R	S	S
ERR067657	R	R	S	S
ERR133955	R	S	S	S
ERR067658	S	S	S	S
ERR067659	R	R	S	S
ERR067660	R	R	S	R
ERR067661	R	S	S	S
ERR067663	R	R	R	S
ERR067664	S	S	S	S
ERR067665	R	R	R	R

ERR067666	S	S	S	S
ERR067667	R	R	R	S
ERR067636	R	S	S	R
ERR067637	S	S	S	S
ERR117457	S	S	S	S
ERR108439	R	R	R	S
ERR133958	S	S	S	S
ERR133959	R	R	S	S
ERR108440	S	S	S	S
ERR133961	R	S	S	S
ERR108441	S	S	S	S
ERR133962	R	R	R	R
ERR133963	R	S	S	S
ERR133964	S	S	S	S
ERR133965	S	S	S	S
ERR108442	S	S	S	S
ERR234563	R	R	S	R
ERR108458	R	R	S	S
ERR133966	R	R	S	S
ERR2516271	R	R	R	S
ERR2516255	R	R	R	R
ERR2516208	R	R	R	S
ERR2516217	R	R	R	R
ERR2516219	R	R	S	S
ERR2516183	R	R	R	R
ERR2516329	R	R	R	R
ERR2516266	R	R	R	S
ERR2516256	R	R	R	R
ERR2516297	R	R	R	U
ERR2516190	R	R	S	U
ERR2516282	R	R	R	U
ERR2516399	R	R	R	R
ERR2516182	S	S	S	R
ERR2516209	R	R	R	R
ERR2516391	R	R	R	R
ERR2514755	S	S	S	S
ERR2513494	S	S	S	S
ERR2514614	S	S	S	S
ERR2513918	S	S	S	S
ERR2514870	S	S	S	S
ERR2514914	S	S	S	S
ERR2512632	S	S	S	S
ERR2513684	S	S	S	S
ERR2513909	S	S	S	S
ERR2513079	S	S	S	S
ERR2513945	S	S	S	S
ERR2514099	S	S	S	S
ERR2514251	S	S	S	S
ERR2514988	S	S	S	U
ERR2514589	S	S	S	S
ERR2514009	S	S	S	S
ERR2512935	S	S	S	S
ERR2513802	S	S	S	S

ERR2512736	S	S	S	S
ERR2515076	S	S	S	S
ERR2515046	S	S	S	S
ERR2513228	S	S	S	S
ERR2514021	R	S	S	S
ERR2514587	S	S	S	S
ERR2513920	S	S	S	S
ERR2513805	S	S	S	S
ERR2513002	S	S	S	S
ERR2514824	S	S	S	S
ERR2514886	R	S	S	S
ERR2514158	S	S	S	S
ERR2514618	R	S	S	S
ERR2513914	R	S	S	S
ERR2512851	U	S	S	S
ERR2514432	S	S	S	S
ERR2513854	S	S	S	S
ERR2513784	R	S	S	S
ERR2513154	S	S	S	S
ERR2514420	S	S	S	S
ERR2514190	R	S	S	S
ERR2512957	S	S	S	S
ERR2514687	S	S	S	S
ERR2512787	S	S	S	S
ERR133967	S	S	S	S
ERR133968	R	S	S	S
ERR133969	R	S	S	S
ERR067668	R	R	R	R
ERR133970	R	S	S	R
ERR067670	R	S	S	S
ERR067671	R	R	R	S
ERR133971	S	S	S	S
ERR067672	R	R	S	S
ERR067673	R	R	S	S
ERR117458	S	S	S	S
ERR133973	S	S	S	S
ERR133974	S	S	S	S
ERR117460	R	R	R	S
ERR067639	R	R	R	R
ERR067640	S	S	S	S
ERR067641	R	R	S	R
ERR133975	R	S	S	S
ERR067643	R	R	R	S
ERR067644	R	R	R	R
ERR067645	R	R	S	S
ERR067674	R	R	R	S
ERR067675	S	S	S	S
ERR067635	R	R	S	S
ERR133977	R	S	S	S
ERR108473	R	R	S	S
ERR133978	S	S	S	S
ERR108474	R	R	S	R
ERR108475	R	R	R	S

ERR108478 S S S S
ERR108479 S S S S
ERR108480 R R R R
ERR108481 S S S S
ERR108482 R R S S
ERR108483 S S S S
ERR133979 S S S S
ERR133980 R S S S
ERR2516251 R R R U
ERR2516186 R R R S
ERR2516285 R R R R
ERR2516367 R R R R
ERR2516226 R R R R
ERR2516280 R R R U
ERR2516356 R R S S
SRS935938 SRP058221 R R S U
SRS935937 SRP058221 R R R U
SRS935948 SRP058221 R R S U
SRS935959 SRP058221 R R S U
SRS935967 SRP058221 R R S U
SRS935986 SRP058221 R R R U
SRS935999 SRP058221 R R R U
SRS936002 SRP058221 R R S U
SRS936026 SRP058221 R R R U
SRS936040 SRP058221 R R S U
SRS935936 SRP058221 R R R U
SRS935926 SRP058221 R R S U
SRS935941 SRP058221 R R R U
SRS935956 SRP058221 R R S U
SRS935955 SRP058221 R R R U
SRS935954 SRP058221 R R R U
SRS935953 SRP058221 R R S U
SRS935952 SRP058221 R R R U
SRS935951 SRP058221 R R R U
SRS935950 SRP058221 R R R U
SRS935949 SRP058221 R R R U
SRS935947 SRP058221 R R R U
SRS935966 SRP058221 R R R U
SRS935957 SRP058221 R R R U
SRS935964 SRP058221 R R S U
SRS935965 SRP058221 R R S U
SRS935962 SRP058221 R R R U
SRS935961 SRP058221 R R R U
SRS935963 SRP058221 R R R U
SRS935958 SRP058221 R R R U
SRS935960 SRP058221 R R S U
SRS935976 SRP058221 R R S U
SRS935975 SRP058221 R R R U
SRS935974 SRP058221 R R S U
SRS935973 SRP058221 R R S U
SRS935972 SRP058221 R R R U
ERR2513880 R R R S
ERR2512421 S S S S

ERR2513896 S S S S
ERR2512522 S S S S
ERR2514255 S S S S
ERR2514198 S S S S
ERR2512926 U U U S
ERR2512573 S S S S
ERR2514640 S S S S
ERR2512531 S S S S
ERR2513260 S S S S
ERR2515052 S S S S
ERR2514293 R S S S
ERR2514777 S S S S
ERR2514332 S S S S
ERR2514249 S S S S
ERR2512696 S S S S
ERR2514991 S S S S
ERR2513352 R R R R
ERR2514641 S S S S
ERR2514284 S S S S
ERR2514482 S S S S
ERR2513269 S S S S
ERR2514656 S S S S
ERR2514662 S S S S
ERR2199740 ERR2199741 S S S S
ERR2199742 S S S S
ERS458164 S S S S
ERS457923 R S R S
ERS457837 S S S S
ERR551085 S S S S
ERR2199743 S S S S
ERR2199744 S S S S
ERR2199745 S S S S
ERR551223 S S S S
ERR552204 S S S S
ERR551150 S S S S
ERR553349 R S S S
ERR2199746 S S S S
ERR552113 R R R R
ERR552505 S S S S
ERR552942 S S S S
ERR552440 S S S S
ERR550646,ERR550647,ERR550645 R R S R
ERR552659 S S S S
ERR551166 R R R R
ERR2199748 ERR552077 R R R R
ERR2199749 S S S U
ERR498373,ERR498374 S S S S
ERR133981 R R R S
ERR2199750 ERR2199751 ERR553354 R R R R
ERR108485 R R S S
ERR551858 S S S S
ERR550975 S S S S
ERR552676 S S S S

ERR551751 S S S S
ERR552249 S S S S
ERR2199752 ERR2199753 S S S S
ERR133982 R R S S
ERR108486 S S S S
ERR2199754 S S S S
ERS457959 S S S S
ERS457317 S S S S
ERS458356 S S S S
ERR552232 S S S S
ERR552845 S S S S
ERR552813 S S S S
ERR553054 S S S S
ERR2199755 ERR2199756 S S S S
ERR551932 S S S S
ERR553379 S S S S
ERR553296 S S S S
ERR550955 S S S S
ERR551839 S S S S
ERR108487 S S S S
ERR552984 S S S S
ERR552619 ERR552620 ERR552621 ERR552622 S S S U
ERR552295 ERR552296 R R R U
ERR2199757 ERR552496 R R R R
ERR552875 ERR552876 ERR552877 ERR552878 R R R R
ERR108488 R R S R
ERR2199758 ERR2199759 ERR551523 S R S S
ERR108489 R R S S
ERR551652 S S S S
ERR2199760 ERR2199761 S S S S
ERR552019 S S S S
ERR551507 S S S S
ERS457396 R R R S
ERS457075 R S R S
ERR553140 S S S S
ERR552371 S S S S
ERR551276 S S S S
ERR551313 S S S S
ERR2199762 ERR2199763 R R S S
ERR551836 S S S S
ERR108490 S S S S
ERR551508 S S S S
ERR551803 S S S S
ERR2199764 ERR2199765 S S S S
ERR133983 S S S S
ERR550653 S S S S
ERR551257 S S S S
ERR552941 S S S S
ERR2199766 ERR2199767 ERR552118 R U R R
ERR552405 R R R R
ERR552672 R R R R
ERR552253 ERR552254 R R R R
ERR551231 R R S S

ERR552502 S S S S
ERR553196 S S S S
ERR552220 S S S S
ERR552736 S S S S
ERR108492 S S S S
ERR553076 S S S S
ERR550698 R S S S
ERR2199769 S S S S
ERR2199770 S S S S
ERR133984 R R R R
ERR552238,ERR552239,ERR552240 R R R R
ERR551178 S S S S
ERR108420 R R R S
ERR551716 S S S S
ERR552273 R R R R
ERR551246 R R R U
ERR551319 R R R R
ERR2199771 S S S U
ERS458617 S R S S
ERR551697,ERR551698 R R R R
ERR550887,ERR550886 R R R S
ERR108421 R R R R
ERR552906,ERR552907 R R R R
ERR551138,ERR551137 R R R R
ERR552051 S S S S
ERR550923,ERR550922 R R R R
ERR553217,ERR553218 R R R R
ERR550764 R R S R
ERR552200,ERR552199 R R S R
ERR552377,ERR552376 R R R S
ERR551489 S S S S
ERR108422 R R S R
ERR552104,ERR552103 R R R R
ERR551095 S S S S
ERR2199772 S S S S
ERR552886 S S S S
ERR133985 R S S S
ERR550701 S S S S
ERR552898 S S S S
ERR552363,ERR552362,ERR552364 R R S S
ERR551846 S S S S
ERR553350 R R R S
ERR552050 S S S R
ERR551269 S S S S
ERR2199773 S S S S
ERR552320 S S S S
ERR551538 S S S S
ERR552846 S S S S
ERR2199776 S S S S
ERR2199774 ERR2199775 S S S U
ERR108423 R S S R
ERR551468,ERR551469 R R R S
ERR553138 S S S S

ERR552317 S S S S
ERR108425 R R S S
ERR108426 R R R S
ERR551900 S S S S
ERR108427 S S S S
ERR2199777 S S S S
ERR551462 R S S S
ERR552870 R U R R
ERR552074 ERR552075 R R R U
ERR133987 S S S S
ERR553056 S S S S
ERR551294 S S S S
ERR108428 R R R S
ERR108429 R S S S
ERR553194,ERR553195 R R R R
ERR553153,ERR553152 R R R S
ERR553139 R R S S
ERR550650 S S S S
ERR551638,ERR551637 R R R S
ERR552586,ERR552585 R R R R
ERR551927 ERR551928 S S S S
ERR552982,ERR552983 R R R R
ERR108443 R R R R
ERR108444 S S S S
ERR2199779 R R R R
ERR108445 S S S R
ERR2199780 S S S S
ERR108446 S S S S
ERR2041682 R R S S
ERR2199781 S S S S
ERR133988 S S S S
ERR2199782 R S S S
ERR108447 R S S S
ERR552090 R R S S
ERR2199783 S S S S
ERR551266 S S S S
ERR133989 S S S S
ERR550997 S S S S
ERR108448 S S S S
ERR552591 S S S S
ERR2041683 R R R S
ERR550876 S S S S
ERR2199784 S S S S
ERR2199785 S S S R
ERR551479 S S S S
ERR2199786 R R R R
ERR2516167 R R R R
ERR2516263 R R S R
ERR2516177 R R R R
ERR2516252 R R S R
ERR2516377 R R S S
SRS935971 SRP058221 R R R U
SRS935970 SRP058221 R R S U

SRS935969	SRP058221	R	R	S	U
SRS935968	SRP058221	R	R	R	U
SRS935994	SRP058221	R	R	S	U
SRS935993	SRP058221	R	R	S	U
SRS935992	SRP058221	R	R	R	U
SRS935991	SRP058221	R	R	R	U
SRS935990	SRP058221	R	R	S	U
SRS935989	SRP058221	R	R	R	U
SRS935988	SRP058221	R	R	R	U
SRS935987	SRP058221	R	R	S	U
SRS935978	SRP058221	R	R	S	U
SRS935977	SRP058221	R	R	S	U
SRS935985	SRP058221	R	R	R	U
SRS935984	SRP058221	R	R	R	U
SRS935979	SRP058221	R	R	R	U
SRS935983	SRP058221	R	R	S	U
SRS935982	SRP058221	R	R	R	U
SRS935981	SRP058221	R	R	R	U
SRS936010	SRP058221	R	R	R	U
SRS936009	SRP058221	R	R	R	U
SRS936008	SRP058221	R	R	R	U
SRS935998	SRP058221	R	R	R	U
SRS935997	SRP058221	R	R	R	U
SRS935996	SRP058221	R	R	S	U
SRS935995	SRP058221	R	R	R	U
SRS936007	SRP058221	R	R	R	U
SRS936006	SRP058221	R	R	R	U
SRS936000	SRP058221	R	R	R	U
SRS936005	SRP058221	R	R	R	U
SRS936004	SRP058221	R	R	R	U
SRS936003	SRP058221	R	R	R	U
SRS936029	SRP058221	R	R	R	U
SRS936028	SRP058221	R	R	S	U
SRS936027	SRP058221	R	R	S	U
SRS936016	SRP058221	R	R	S	U
SRS936015	SRP058221	R	R	S	U
SRS936014	SRP058221	R	R	S	U
SRS936013	SRP058221	R	R	S	U
PRJNA413593	S	S	S	U	
PRJNA413593	S	S	S	U	
PRJNA413593	S	S	S	U	
PRJNA413593	S	S	S	U	
PRJNA413593	S	S	S	U	
PRJNA413593	S	S	S	U	
PRJNA413593	S	S	R	U	
PRJNA413593	S	S	S	U	
PRJNA413593	S	S	S	U	
PRJNA413593	S	S	S	U	
PRJNA413593	S	S	S	U	
PRJNA413593	S	S	S	U	
PRJNA413593	S	S	S	U	
PRJNA413593	S	S	S	U	
PRJNA413593	S	S	S	S	

PRJNA413593	S	S	S	U
PRJNA413593	S	S	S	U
PRJNA413593	S	S	S	U
PRJNA413593	S	S	S	U
PRJNA413593	S	S	S	U
PRJNA413593	S	S	S	U
PRJNA413593	S	S	S	U
PRJNA413593	S	S	S	U
PRJNA413593	S	S	S	U
PRJNA413593	S	S	S	U
PRJNA413593	S	S	S	U
PRJNA413593	S	S	S	U
PRJNA413593	S	S	S	U
PRJNA413593	R	S	S	S
PRJNA413593	R	S	S	S
PRJNA413593	S	S	S	U
PRJNA413593	S	S	S	U
PRJNA413593	R	S	S	S
PRJNA413593	S	S	S	U
PRJNA413593	S	S	S	U
PRJNA413593	S	S	S	U
PRJNA413593	S	S	S	U
PRJNA413593	S	S	S	U
PRJNA413593	S	S	S	U
PRJNA413593	S	S	S	U
PRJNA413593	S	S	S	U
PRJNA413593	R	S	S	S
PRJNA413593	S	S	S	U
PRJNA413593	R	S	S	S
PRJNA413593	S	S	S	U
PRJNA413593	S	S	S	U
PRJNA413593	S	S	S	U
PRJNA413593	R	R	S	R
PRJNA413593	R	S	S	S
PRJNA413593	S	S	S	U
PRJNA413593	S	S	S	U
PRJNA413593	S	S	S	U
PRJNA413593	S	S	S	U
PRJNA413593	S	S	S	S
PRJNA413593	S	S	S	U
PRJNA413593	S	S	S	U
PRJNA413593	S	S	S	U
PRJNA413593	S	S	S	U
PRJNA413593	S	S	S	U
PRJNA413593	S	S	S	U
PRJNA413593	S	S	S	U
PRJNA413593	S	S	S	U
PRJNA413593	S	S	S	U
PRJNA413593	S	S	S	U
PRJNA413593	S	S	S	U
PRJNA413593	R	S	S	S
PRJNA413593	S	S	S	U
PRJNA413593	S	S	S	U

PRJNA413593	S	S	S	U
PRJNA413593	S	S	S	U
PRJNA413593	R	S	S	S
PRJNA413593	R	S	S	S
PRJNA413593	S	S	S	S
PRJNA413593	S	S	S	U
PRJNA413593	S	S	S	U
PRJNA413593	S	S	S	U
PRJNA413593	S	S	S	U
PRJNA413593	S	S	S	U
PRJNA413593	S	S	S	U
PRJNA413593	S	S	S	U
PRJNA413593	S	S	S	U
PRJNA413593	S	S	S	U
PRJNA413593	R	S	S	S
PRJNA413593	S	S	S	U
PRJNA413593	S	S	S	U
PRJNA413593	R	S	S	S
PRJNA413593	S	S	S	U
PRJNA413593	S	S	S	U
PRJNA413593	S	S	S	U
PRJNA413593	S	S	S	U
PRJNA413593	S	S	S	U
PRJNA413593	S	S	S	U
PRJNA413593	S	S	S	U
PRJNA413593	S	S	S	U
PRJNA413593	S	S	S	U
PRJNA413593	S	S	S	U
PRJNA413593	S	S	S	U
PRJNA413593	S	S	S	U
PRJNA413593	S	S	S	U
PRJNA413593	R	S	S	S
PRJNA413593	S	S	S	U
PRJNA413593	S	S	S	U
PRJNA413593	S	S	S	U
PRJNA413593	S	S	S	U
PRJNA413593	R	S	S	S
PRJNA413593	S	S	S	U
PRJNA413593	S	S	S	U
PRJNA413593	S	S	S	U
PRJNA413593	S	S	S	U
PRJNA413593	S	S	S	U
PRJNA413593	R	S	S	S
PRJNA413593	R	S	S	S
PRJNA413593	S	S	S	U
PRJNA413593	S	S	S	U
PRJNA413593	S	S	S	U
PRJNA413593	S	S	S	U
PRJNA413593	R	S	S	S
PRJNA413593	S	S	S	U
PRJNA413593	S	S	S	U
PRJNA413593	S	S	S	U
PRJNA413593	R	S	S	S

ERR2512720	S	S	S	S
ERR2513113	R	S	S	S
ERR2512762	S	S	S	S
ERR2512482	S	S	S	S
ERR2513272	S	S	S	S
ERR2514334	S	S	S	S
ERR2512478	R	S	S	S
ERR2513031	S	S	S	S
ERR2513498	S	S	S	S
ERR2514969	S	S	S	S
ERR2513340	S	S	S	S
ERR2514373	S	S	S	S
ERR2514040	U	S	S	S
ERR2513673	S	S	S	S
ERR2514776	S	S	S	S
ERR2513668	S	S	S	S
ERR2514583	S	S	S	R
ERR2512447	S	S	S	S
ERR2512962	S	S	S	R
ERR2513164	S	S	S	S
ERR2513648	S	S	S	S
ERR2515038	S	S	S	S
ERR2515066	S	S	S	S
ERR2513256	S	S	S	S
ERR2514035	S	S	S	S
ERR2513135	S	S	S	S
ERR2513344	S	S	S	S
ERR2512684	U	S	S	S
ERR2513320	S	S	S	S
ERR2514104	S	S	S	S
ERR2514469	S	S	S	S
ERR2514775	S	S	S	S
ERR2514782	S	S	S	S
ERR2512430	S	S	S	S
ERR2513798	S	S	S	S
ERR2514791	S	S	S	S
ERR2515100	S	S	S	S
ERR2514247	S	S	S	S
ERR2514992	S	S	S	S
ERR2512742	S	S	S	S
ERR2514647	S	S	S	S
ERR2514416	S	S	S	S
ERR2514374	S	S	S	S
ERR2513553	S	S	S	S
ERR2513433	S	S	S	R
ERR2513580	S	S	S	S
ERR2513859	S	S	S	S
ERR2514839	S	S	S	S
ERR2514538	S	S	S	S
ERR2513978	S	S	S	S
ERR2513921	S	S	S	S
ERR2513725	S	S	S	S
ERR2514603	S	S	S	S

ERR2513776 S S S U
ERR2513323 S S S S
ERR2514380 S S S S
ERR2199787 S S S U
ERR2041684 R R R S
ERR550861 R R R R
ERR552720 R R R U
ERR2199788 S S S S
ERR137191 S S S S
ERR552030 R S S S
ERR551302 S S S S
ERR2199789 S S S S
ERR2199790 ERR2199791 S S S S
ERR551856 S S S S
ERS458701 R S S S
ERS458377 S S S S
ERR2041685 R R R S
ERR551917 S S S R
ERR550689 S S S S
ERR550878 S S S S
ERR2199792 S S S S
ERR550820 S S S S
ERR2041686 ERR2041687 R R U R
ERR553245 S S S S
ERR552608 S S S S
ERR2199793 ERR553059 R R R R
ERR550754 R R R R
ERR551739 R R R R
ERR553321 R R R R
ERR553109 S S S S
ERR2199794 S S S S
ERR108493 R R R S
ERR2199795 R S S S
ERR108494 R R R R
ERR108495 R R R S
ERR2041688 R R R R
ERR550877 S S S S
ERR551476 S S S S
ERR551400 ERR551401 S S S S
ERR551703 S S S S
ERR551342,ERR551343 R R R R
ERR551130,ERR551131 R R R R
ERR550651,ERR550652 R R R S
ERR553169,ERR553168 R R R R
ERR550742 S S S S
ERR108496 S S S S
ERR552852 S S S S
ERR553188,ERR553189,ERR553190 R R R R
ERR108497 S S S S
ERR2199796 S S S S
ERR2041689 R R R R
ERR551691 S S S S
ERR551498 S S S S

ERR552292 S S S S
ERR552334 S S S S
ERR2199797 S S S S
ERR2199798 S S S S
ERR553341 S S S S
ERR550803 S S S S
ERR551355 S S S S
ERR553240 S S S S
ERR2041690 ERR2041691 R R U R
ERR551001 S S S S
ERR551163 S S S S
ERR551323 S S S S
ERR553100 S S S S
ERR550702 S S S S
ERR552872,ERR552873,ERR552874 R S S S
ERR2041692 ERR2041693 R R R S
ERR551756 S S S S
ERR551473 S S S S
ERR553257 S S S S
ERR2199799 R R R S
ERR551402 S S S S
ERR553008 S S S S
ERR550954 S S S R
ERR137192 R R R R
ERR2199800 S S S S
ERR137193 S S S S
ERR551144 S S S S
ERR550760 S S S S
ERR551669 S S S S
ERR552571,ERR552569,ERR552570 R R R S
ERR553053 R R R R
ERR2041694 ERR2041695 R R U R
ERR551288 S S S S
ERR553331 S S S S
ERR552167 S S S S
ERR137195 S S S S
ERR2199801 S S S S
ERR552012,ERR552011 R R R R
ERR552221 R S S S
ERR551593 S S S S
ERR2199802 ERR2199803 S S S S
ERS457211 S S S S
ERS457267 S S S S
ERS458241 R S R S
ERR137197 R S R S
ERR552742 S S S S
ERR551002 S S S S
ERR551046 S S S S
ERR551656 S S S S
ERR552903 S S S S
ERR552338 S S S S
ERR137198 R S S S
ERR067676 S S S S

ERR552613 S S S S
ERR2199804 R S S S
ERR137199 R S S S
ERR137200 S S S S
ERR553208 R R R R
ERR067677 R R S R
ERR137201 R S S S
ERR067678 R R S S
ERR2199806 S S S S
ERR552438 S S S S
ERR550789 S S S S
ERR553307 S S S S
ERR067679 R R S S
ERR552525 S S S S
ERR552114 S S S S
ERR2516303 R R S S
ERR2516373 R R R R
ERR2516198 R R R S
ERR2516382 R R R R
ERR2516181 R R R R
ERR2516361 R R S R
ERR2516301 R R S S
ERR2516254 R R S R
ERR2516333 R R R R
ERR2516375 R R R S
ERR2516352 R R R R
ERR2516289 R R S R
ERR2516302 R R R R
ERR2516206 R R R R
SRS936012 SRP058221 R R R U
SRS936011 SRP058221 R R S U
SRS936025 SRP058221 R R S U
SRS936024 SRP058221 R R R U
SRS936023 SRP058221 R R R U
SRS936022 SRP058221 R R R U
SRS936021 SRP058221 R R R U
SRS936020 SRP058221 R R S U
SRS936019 SRP058221 R R S U
SRS936018 SRP058221 R R S U
SRS936017 SRP058221 R R R U
SRS936041 SRP058221 R R S U
SRS936039 SRP058221 R R R U
SRS936038 SRP058221 R R R U
SRS936037 SRP058221 R R R U
SRS936036 SRP058221 R R R U
SRS936033 SRP058221 R R S U
SRS936032 SRP058221 R R S U
SRS936031 SRP058221 R R R U
PRJNA413593 S S S U
PRJNA413593 R S S R
PRJNA413593 S S S U
PRJNA413593 S S S U
PRJNA413593 S S S U

PRJNA413593	S	S	S	S
PRJNA413593	S	S	S	U
PRJNA413593	S	S	S	U
PRJNA413593	S	S	S	U
PRJNA413593	S	S	S	U
PRJNA413593	S	S	S	U
PRJNA413593	S	S	S	U
PRJNA413593	S	S	S	U
PRJNA413593	R	S	S	S
PRJNA413593	R	S	S	S
PRJNA413593	S	S	S	U
PRJNA413593	R	S	S	S
PRJNA413593	R	S	S	S
PRJNA413593	S	S	S	U
PRJNA413593	S	S	S	U
PRJNA413593	R	S	S	S
PRJNA413593	S	S	S	U
PRJNA413593	S	S	S	U
PRJNA413593	S	S	S	U
PRJNA413593	S	S	S	U
PRJNA413593	S	R	S	S
PRJNA413593	R	S	S	S
PRJNA413593	S	S	S	U
PRJNA413593	R	S	S	S
PRJNA413593	R	S	S	S
PRJNA413593	S	S	S	U
PRJNA413593	S	S	S	U
PRJNA413593	S	S	S	U
PRJNA413593	S	S	S	U
PRJNA413593	S	S	S	U
PRJNA413593	S	S	S	U
PRJNA413593	R	S	S	S
PRJNA413593	S	S	S	U
PRJNA413593	S	S	S	U
PRJNA413593	S	S	S	U
PRJNA413593	S	S	S	U
PRJNA413593	S	S	S	U
PRJNA413593	S	S	S	U
PRJNA413593	R	S	S	S
PRJNA413593	S	S	S	U
PRJNA413593	R	S	S	S
PRJNA413593	S	S	S	U
PRJNA413593	S	S	S	U
PRJNA413593	S	S	S	U
PRJNA413593	S	S	S	U
PRJNA413593	S	S	S	U
PRJNA413593	S	S	S	U
PRJNA413593	S	S	S	U
PRJNA413593	S	S	S	U
PRJNA413593	S	S	S	U
PRJNA413593	S	S	S	U
PRJNA413593	S	S	S	U
PRJNA413593	S	S	S	U

PRJNA413593	S	S	S	U
PRJNA413593	S	S	S	U
PRJNA413593	S	S	S	U
PRJNA413593	S	S	S	U
PRJNA413593	R	S	S	S
PRJNA413593	S	S	S	U
PRJNA413593	S	S	S	U
PRJNA413593	S	S	S	U
PRJNA413593	S	S	S	U
PRJNA413593	S	S	S	U
PRJNA413593	R	S	S	S
PRJNA413593	R	S	S	S
PRJNA413593	R	S	S	S
PRJNA413593	R	S	S	S
PRJNA413593	S	S	S	U
PRJNA413593	S	S	S	U
PRJNA413593	S	S	S	U
PRJNA413593	S	S	S	U
PRJNA413593	S	S	S	U
PRJNA413593	S	S	S	U
PRJNA413593	S	S	S	U
PRJNA413593	S	S	S	U
PRJNA413593	S	S	S	U
PRJNA413593	S	S	S	U
PRJNA413593	S	S	S	U
PRJNA413593	S	S	S	U
PRJNA413593	S	S	S	U
PRJNA413593	S	S	S	U
PRJNA413593	S	S	S	U
PRJNA413593	S	S	S	U
PRJNA413593	S	S	S	U
PRJNA413593	R	S	S	S
PRJNA413593	S	S	S	U
PRJNA413593	S	S	S	U
PRJNA413593	S	S	S	U
PRJNA413593	S	S	S	U
PRJNA413593	S	S	S	U
PRJNA413593	S	S	S	U
PRJNA413593	S	S	S	U
PRJNA413593	S	S	S	U
PRJNA413593	S	S	S	U
PRJNA413593	R	S	S	S
PRJNA413593	R	S	S	S
PRJNA413593	R	S	S	S
PRJNA413593	S	S	S	U
PRJNA413593	S	S	S	U
PRJNA413593	S	S	S	U
PRJNA413593	R	S	S	S
PRJNA413593	S	S	S	U
PRJNA413593	S	S	S	U
PRJNA413593	S	S	S	U
PRJNA413593	S	S	S	U
PRJNA413593	S	S	S	U

PRJNA413593	S	S	S	U
PRJNA413593	S	S	S	U
PRJNA413593	S	S	S	U
PRJNA413593	S	S	S	U
PRJNA413593	S	S	S	U
PRJNA413593	S	S	S	U
PRJNA413593	S	S	S	U
PRJNA413593	S	S	S	U
PRJNA413593	S	S	S	U
PRJNA413593	R	R	S	R
PRJNA413593	S	S	S	U
PRJNA413593	S	S	S	U
PRJNA413593	S	S	S	U
PRJNA413593	S	S	S	U
PRJNA413593	S	S	S	U
PRJNA413593	R	S	S	S
PRJNA413593	S	S	S	U
PRJNA413593	S	S	S	U
PRJNA413593	S	S	S	U
PRJNA413593	S	S	S	U
PRJNA413593	R	S	S	S
ERR2512747	S	S	S	S
ERR2512816	S	S	S	S
ERR2514506	S	S	S	S
ERR2512515	S	S	S	S
ERR2514188	R	S	S	S
ERR2513293	S	S	S	S
ERR2515092	S	S	S	S
ERR2515057	S	S	S	S
ERR2513944	S	S	S	S
ERR2513206	S	S	S	S
ERR2513963	S	S	S	S
ERR2514110	S	S	S	S
ERR2514466	S	S	S	S
ERR2513867	S	S	S	S
ERR2514510	S	S	S	S
ERR2513552	S	S	S	S
ERR2513373	S	S	S	S
ERR2513697	S	S	S	S
ERR2514452	S	S	S	S
ERR2512988	S	S	S	S
ERR2514453	S	S	S	S
ERR2513463	S	S	S	S
ERR2513326	S	S	S	S
ERR2512925	S	S	S	S
ERR2512772	S	S	S	S
ERR2513816	S	S	S	S
ERR2514903	S	S	S	S
ERR2513962	S	S	S	S
ERR2514963	R	S	S	S
ERR2513719	S	S	S	S
ERR2514932	S	S	S	S
ERR2513186	S	S	S	S

ERR2513760	S	S	S	S
ERR2515049	S	S	S	S
ERR2513698	S	S	S	S
ERR2514890	S	S	S	S
ERR2512654	S	S	S	S
ERR2513341	S	S	S	S
ERR2514225	S	S	S	S
ERR2513586	S	S	S	S
ERR2514620	S	S	S	S
ERR2514309	S	S	S	S
ERR2514975	S	S	S	S
ERR2514921	S	S	S	S
ERR2514828	S	S	S	S
ERR2514180	S	S	S	S
ERR2514144	S	S	S	R
ERR2513993	S	S	S	S
ERR2514229	S	S	S	S
ERR2513096	S	S	S	S
ERR2514236	S	S	S	S
ERR2513422	S	S	S	S
ERR2513893	S	S	S	S
ERR2513022	S	S	S	S
ERR2513513	S	S	S	S
ERR2514746	S	S	S	S
ERR2514936	S	S	S	S
ERR2513457	S	S	S	S
ERR2514066	S	S	S	S
ERR2512435	S	S	S	S
ERR2514600	S	S	S	S
ERR2513074	S	S	S	S
ERR2514208	S	S	S	S
ERR2514520	S	S	S	S
ERR2513677	S	S	S	S
ERR2513651	S	S	S	S
ERR2512579	S	S	S	S
ERR2514396	S	S	S	S
ERR2513339	S	S	S	S
ERR2512466	S	S	S	S
ERR2514697	S	S	S	S
ERR2513034	S	S	S	R
ERR2512711	S	S	S	S
ERR2513452	S	S	S	S
ERR2513765	S	S	S	S
ERR2515045	S	S	S	S
ERR2514023	S	S	S	S
ERR2513161	S	S	S	S
ERR2513556	S	S	S	S
ERR2514480	S	S	S	S
ERR2514900	S	S	S	S
ERR2513077	S	S	S	S
ERR2514946	S	S	S	S
ERR2514804	S	S	S	S
ERR2515000	S	S	S	S

ERR2513891	S	S	S	S
ERR2514044	S	S	S	S
ERR2514177	S	S	S	S
ERR2514113	S	S	S	S
ERR2514554	S	S	S	S
ERR2513492	S	S	S	S
ERR2512385	S	S	S	S
ERR2514872	S	S	S	S
ERR2514359	S	S	S	S
ERR2514072	S	S	S	S
ERR2514473	S	S	S	S
ERR2514704	S	S	S	S
ERR2514680	S	S	S	S
ERR2514323	S	S	S	S
ERR2514818	S	S	S	S
ERR2515086	S	S	S	S
ERR2514220	S	S	U	S
ERR2512386	S	R	S	S
ERR2515030	S	S	S	S
ERR2512387	S	S	S	S
ERR2514701	S	S	S	S
ERR2514500	S	S	S	S
ERR2514684	S	S	S	S
ERR2513058	S	S	S	S
ERR2514195	S	S	S	S
ERR2513350	S	S	S	S
ERR2512388	S	S	S	S
ERR2513368	S	S	S	S
ERR2514549	S	S	S	S
ERR2514122	S	S	S	S
ERR2513952	S	S	S	S
ERR2512389	S	S	S	S
ERR2514280	S	S	S	S
ERR2513620	S	S	S	S
ERR2513212	S	S	S	S
ERR2514461	S	S	S	S
ERR2513749	S	S	S	S
ERR2514020	S	S	S	S
ERR2514716	S	S	S	S
ERR2512390	S	S	S	S
ERR2514145	S	S	S	S
ERR2514058	S	S	S	S
ERR2513822	S	S	S	S
ERR2512534	S	S	S	S
ERR2512666	S	S	S	S
ERR2514971	S	S	S	S
ERR2514770	S	S	S	S
ERR2514143	S	S	S	S
ERR2514186	S	S	S	S
ERR2513544	R	S	S	S
ERR2513799	S	S	S	S
ERR2514527	S	S	S	S
ERR2515012	S	S	S	S

ERR2514312	S	S	S	S
ERR2512438	S	S	S	S
ERR2514719	S	S	S	S
ERR2515015	S	S	S	S
ERR2512391	R	S	S	S
ERR2512392	S	S	S	S
ERR2514371	S	S	S	S
ERR2513786	R	R	R	R
ERR2512793	S	S	S	S
ERR2512393	S	S	S	S
ERR2512394	S	S	S	S
ERR2514000	S	S	S	S
ERR2514349	S	S	S	S
ERR2512979	R	R	R	R
ERR2512784	S	S	S	S
ERR2514882	S	S	S	S
ERR2512891	S	S	S	S
ERR2515054	S	S	S	S
ERR2512583	S	S	S	S
ERR2512920	S	S	S	S
ERR2513466	S	S	S	S
ERR2514616	S	S	S	S
ERR2514835	S	S	S	S
ERR2513102	S	S	S	S
ERR2514407	S	S	S	S
ERR2512539	S	S	S	S
ERR2513370	S	S	S	S
ERR2513374	S	S	S	S
ERR2513479	S	S	S	S
ERR2512771	S	S	S	S
ERR2513083	S	S	S	S
ERR2514905	S	S	S	S
ERR2512395	S	S	S	S
ERR2514187	S	S	S	S
ERR2514856	S	S	S	S
ERR2512396	S	S	S	S
ERR2513014	S	S	S	S
ERR2513751	S	S	S	S
ERR2514610	S	S	S	S
ERR2514884	S	S	S	S
ERR2514391	S	S	S	S
ERR2513996	S	S	S	S
ERR2514166	S	S	S	S
ERR2514032	S	S	S	S
ERR2514339	S	S	S	S
ERR2513214	S	S	S	S
ERR2514496	S	S	S	S
ERR2512448	S	S	S	S
ERR2513057	S	S	S	S
ERR2512665	S	S	S	S
ERR2513232	R	S	S	S
ERR2513548	S	S	S	S
ERR2515053	S	S	S	S

ERR2513506	S	S	S	S
ERR2512562	S	S	S	S
ERR2513721	S	S	S	S
ERR2512397	S	S	S	S
ERR2514569	S	S	S	S
ERR2513917	S	S	S	S
ERR2513158	S	S	S	S
ERR2512917	S	S	S	S
ERR2513696	S	S	S	S
ERR2514402	S	S	S	S
ERR2512985	S	S	S	S
ERR2514542	S	S	S	S
ERR2512809	S	S	S	S
ERR2512398	S	S	S	S
ERR2512476	S	S	S	S
ERR2513455	S	S	S	S
ERR2515080	S	S	S	S
ERR2514512	S	S	S	S
ERR2512839	S	S	S	R
ERR2514337	S	S	S	S
ERR2512901	S	S	S	S
ERR2513085	R	S	S	S
ERR2513468	S	S	S	S
ERR2512399	S	S	S	S
ERR2513515	S	S	S	S
ERR2513358	S	S	S	S
ERR2513693	S	S	S	S
ERR2512593	S	S	S	S
ERR2514922	S	S	S	S
ERR2514378	S	S	S	S
ERR2513355	S	S	S	S
ERR2515037	S	S	S	S
ERR2512980	S	S	S	S
ERR2512813	S	S	S	S
ERR2515061	S	S	S	S
ERR2512400	S	S	S	S
ERR2512713	S	S	S	S
ERR2512401	S	S	S	S
ERR2512402	R	S	S	S
ERR2512807	S	S	S	S
ERR2513284	S	S	S	S
ERR2512585	R	S	S	S
ERR2513781	S	S	S	S
ERR2514115	S	S	S	S
ERR2514999	S	S	S	S
ERR2512569	S	S	S	S
ERR2515023	S	S	S	S
ERR2512461	S	S	S	S
ERR2514598	S	S	S	S
ERR2515028	S	S	S	S
ERR2514501	S	S	S	S
ERR2513685	S	S	S	S
ERR2514594	S	S	S	S

ERR2513437	S	S	S	S
ERR2512462	S	S	S	S
ERR2512928	S	S	S	S
ERR2513036	S	S	S	S
ERR2513524	S	S	S	S
ERR2514065	S	S	S	S
ERR2514783	S	S	S	S
ERR2513928	S	S	S	S
ERR2513008	S	S	S	S
ERR2513934	S	S	S	S
ERR2514930	S	S	S	S
ERR2512776	S	S	S	S
ERR2512611	S	S	S	S
ERR2514976	S	S	S	S
ERR2512910	S	S	S	S
ERR2512403	S	S	S	S
ERR2512782	S	S	S	S
ERR2512933	S	S	S	S
ERR2512404	S	S	S	S
ERR2514007	S	S	S	S
ERR2512405	S	S	S	S
ERR2513097	S	S	S	S
ERR2512458	S	S	S	S
ERR2514964	S	S	S	S
ERR2513974	S	S	S	S
ERR2512588	S	S	S	S
ERR2513166	S	S	S	S
ERR2514437	S	S	S	S
ERR2513435	S	S	S	S
ERR2514536	R	S	S	S
ERR2513540	S	S	S	S
ERR2514033	S	S	S	S
ERR2514367	S	S	S	S
ERR2512879	S	S	S	S
ERR2513870	S	S	S	S
ERR2514376	S	S	S	S
ERR2514459	S	S	S	S
ERR2512551	S	S	S	S
ERR2513695	S	S	S	S
ERR2514666	S	S	S	R
ERR2515056	S	S	S	S
ERR2513956	S	S	S	S
ERR2514201	S	S	S	S
ERR2513499	S	S	S	S
ERR2514387	S	S	S	S
ERR2512406	S	S	S	S
ERR2514242	S	S	S	S
ERR2513627	S	S	S	S
ERR2513294	S	S	S	S
ERR2513888	S	S	S	S
ERR2512423	S	S	S	S
ERR2514615	S	S	S	S
ERR2514199	S	S	S	S

ERR2514754	S	S	S	S
ERR2514399	S	S	S	S
ERR2512794	S	S	S	S
ERR2513301	S	S	S	S
ERR2514027	S	S	S	S
ERR2514347	S	S	S	S
ERR2513601	S	S	S	S
ERR2513312	S	S	S	S
ERR2513584	S	S	S	S
ERR2513629	S	S	S	S
ERR2512857	S	S	S	S
ERR2513456	S	S	S	S
ERR2514545	S	S	S	R
ERR2514003	S	S	S	S
ERR2512498	S	S	S	S
ERR2514847	S	S	S	S
ERR2513839	S	S	S	S
ERR2512439	S	S	S	S
ERR2512889	S	S	S	S
ERR2512781	U	U	U	S
ERR2512407	S	S	S	S
ERR2513947	S	S	S	S
ERR2513894	S	S	S	S
ERR2513211	S	S	S	S
ERR2513943	S	S	S	S
ERR2514456	S	S	S	S
ERR2514206	S	S	S	S
ERR2512646	S	S	S	S
ERR2514322	S	S	S	S
ERR2513654	S	S	S	S
ERR2512907	R	S	S	S
ERR2514223	S	S	S	S
ERR2514874	S	S	S	S
ERR2512607	S	S	S	S
ERR2513550	S	S	S	S
ERR2512876	U	U	U	S
ERR2514384	S	S	S	S
ERR2513472	S	S	S	S
ERR2514631	R	S	S	S
ERR2512799	S	S	S	S
ERR2512514	S	S	S	S
ERR2514901	S	S	S	S
ERR2514808	S	S	S	S
ERR2513857	S	S	S	S
ERR2512659	S	S	S	S
ERR2514050	S	S	S	S
ERR2513112	S	S	S	S
ERR2514320	S	S	S	S
ERR2514184	S	S	S	S
ERR2513771	S	S	S	S
ERR2513915	S	S	S	S
ERR552384,ERR552383	R	R	R	S
ERR553368	S	S	S	S

ERR551692 R R R R
ERR552061 S S S S
ERR553052 S S S S
ERR2199807 S S S U
ERR2199808 S S S S
ERR2199809 S S S S
ERR551660,ERR551659 R R S R
ERR2199810 S S S S
ERR552795 S S S S
ERR550686 S S S S
ERR2516343 R R R R
ERR550925 S S S S
ERR2199812 S S S S
ERR2199813 ERR2199814 ERR2199815 S S S U
ERR2199816 S S S S
ERR137203 S S S S
ERR551405 S S S S
ERR137204 R R R R
ERR550793 S S S S
ERR137205 R S R S
ERR551233,ERR551234 R R S S
ERR552694 S S S S
ERR552256 S S S S
ERR2199817 S S S S
ERR137206 R R S S
ERR2199818 R R R R
ERR2199819 S S S S
ERR137207 S S R S
ERR552283 ERR552284 S S S S
ERS458627 S R S R
ERR137208 R R R R
ERR137209 R R S R
ERR137210 R R S S
ERR2199820 S S S S
ERR2199821 R S S S
ERR2199822 S S S S
ERR2199823 S S S S
ERR108472 S S S S
ERR2516287 R S R R
ERR2516357 R R S S
ERR2516274 R R S S
ERR2516270 R R R R
ERR2516295 R R S S
ERR2516392 R R S S
ERR2516364 R R R R
ERR2516261 R R R R
ERR2516347 R R S R
ERR2516365 R R S R
ERR2516257 R R R S
ERR2516349 R R R R
ERR2516166 R R R R
ERR2516286 R R R S
ERR2516175 R R S R

SRS936030	SRP058221	R	R	S	U
SRS936035	SRP058221	R	R	R	U
SRS936034	SRP058221	R	R	R	U
SRS935935	SRP058221	R	R	R	U
SRS935934	SRP058221	R	R	S	U
SRS935932	SRP058221	R	R	S	U
SRS935933	SRP058221	R	R	S	U
SRS935920	SRP058221	R	R	R	U
SRS935921	SRP058221	R	R	R	U
SRS935931	SRP058221	R	R	R	U
SRS935930	SRP058221	R	R	R	U
SRS935929	SRP058221	R	R	R	U
SRS935928	SRP058221	R	R	S	U
SRS935927	SRP058221	R	R	R	U
SRS935922	SRP058221	R	R	R	U
SRS935925	SRP058221	R	R	S	U
SRS935924	SRP058221	R	R	R	U
SRS935923	SRP058221	R	R	R	U
SRS935946	SRP058221	R	R	S	U
SRS935944	SRP058221	R	R	R	U
SRS935945	SRP058221	R	R	R	U
SRS935943	SRP058221	R	R	R	U
SRS935942	SRP058221	R	R	R	U
SRS935940	SRP058221	R	R	R	U
SRS935939	SRP058221	R	R	R	U
PRJNA413593	S	S	S	U	
PRJNA413593	S	S	S	U	
PRJNA413593	S	S	S	U	
PRJNA413593	S	S	S	U	
PRJNA413593	S	S	S	U	
PRJNA413593	R	S	S	S	
PRJNA413593	S	S	S	U	
PRJNA413593	S	S	S	U	
PRJNA413593	S	S	S	U	
PRJNA413593	S	S	S	U	
PRJNA413593	S	S	S	U	
PRJNA413593	S	S	S	U	
PRJNA413593	R	S	S	S	
PRJNA413593	R	S	S	S	
PRJNA413593	S	S	S	U	
PRJNA413593	R	S	S	S	
PRJNA413593	S	S	S	U	
PRJNA413593	R	S	S	S	
PRJNA413593	S	S	S	U	
PRJNA413593	S	S	S	U	
PRJNA413593	S	S	S	U	
PRJNA413593	S	S	S	U	
PRJNA413593	S	S	S	U	
PRJNA413593	S	S	S	U	
PRJNA413593	S	S	S	U	
PRJNA413593	S	S	S	U	
PRJNA413593	R	S	S	S	
PRJNA413593	S	S	S	U	

PRJNA413593	S	S	S	U
PRJNA413593	S	S	S	U
PRJNA413593	S	S	S	U
PRJNA413593	S	S	S	U
PRJNA413593	S	S	S	U
PRJNA413593	S	S	S	U
PRJNA413593	S	S	S	U
PRJNA413593	S	S	S	U
PRJNA413593	S	S	S	U
PRJNA413593	S	S	S	U
PRJNA413593	R	S	S	S
PRJNA413593	S	S	S	U
PRJNA413593	S	S	S	U
PRJNA413593	S	S	S	U
PRJNA413593	S	S	S	U
PRJNA413593	S	S	S	U
PRJNA413593	S	S	S	U
PRJNA413593	S	S	S	U
PRJNA413593	S	S	S	U
PRJNA413593	S	S	S	U
PRJNA413593	S	S	S	U
PRJNA413593	S	S	S	U
PRJNA413593	S	S	S	U
PRJNA413593	R	S	S	S
PRJNA413593	S	S	S	U
PRJNA413593	S	S	S	U
PRJNA413593	S	S	S	U
PRJNA413593	S	S	S	U
PRJNA413593	S	S	S	U
PRJNA413593	S	S	S	U
PRJNA413593	S	S	S	U
PRJNA413593	S	S	S	U
PRJNA413593	S	S	S	U
PRJNA413593	S	S	S	U
PRJNA413593	S	S	S	U
PRJNA413593	S	S	S	U
PRJNA413593	S	S	S	U
PRJNA413593	R	S	S	S
PRJNA413593	S	S	S	U
PRJNA413593	R	R	R	R
PRJNA413593	S	S	S	U
PRJNA413593	S	S	S	U
PRJNA413593	S	S	S	U
PRJNA413593	R	S	S	S
PRJNA413593	R	S	S	S
PRJNA413593	S	S	S	U
PRJNA413593	S	S	S	U
PRJNA413593	S	S	S	U
PRJNA413593	S	S	S	U
PRJNA413593	S	S	S	U
PRJNA413593	S	S	S	U
PRJNA413593	S	S	S	U
PRJNA413593	S	S	S	U

PRJNA413593	S	S	S	U
PRJNA413593	S	S	S	U
PRJNA413593	S	S	S	U
PRJNA413593	S	S	S	U
PRJNA413593	S	S	S	U
PRJNA413593	S	S	S	U
PRJNA413593	S	S	S	U
PRJNA413593	S	S	S	U
PRJNA413593	S	S	S	U
PRJNA413593	S	S	S	U
PRJNA413593	S	S	S	U
PRJNA413593	S	S	S	U
PRJNA413593	S	S	S	U
PRJNA413593	S	S	S	S
PRJNA413593	S	S	S	U
PRJNA413593	S	S	S	U
PRJNA413593	R	R	S	R
PRJNA413593	R	S	S	S
PRJNA413593	S	S	S	U
PRJNA413593	S	S	S	U
PRJNA413593	S	S	S	U
PRJNA413593	S	S	S	U
PRJNA413593	S	S	S	S
PRJNA413593	R	S	S	S
PRJNA413593	S	S	S	U
PRJNA413593	S	S	S	U
PRJNA413593	S	S	S	U
PRJNA413593	S	S	S	U
PRJNA413593	R	S	S	S
PRJNA413593	S	S	S	U
PRJNA413593	S	S	S	U
PRJNA413593	S	S	S	U
PRJNA413593	S	S	S	U
PRJNA413593	S	S	S	U
PRJNA413593	S	S	S	U
PRJNA413593	S	S	S	U
PRJNA413593	S	S	S	U
PRJNA413593	S	S	S	U
PRJNA413593	R	S	S	S
PRJNA413593	S	S	S	U
PRJNA413593	S	S	S	U
PRJNA413593	R	S	S	S
PRJNA413593	S	S	S	U
PRJNA413593	S	S	S	U
PRJNA413593	S	S	S	U
PRJNA413593	S	S	S	U
PRJNA413593	S	S	S	U
PRJNA413593	S	S	S	U
PRJNA413593	S	S	S	U
PRJNA413593	S	S	S	U
PRJNA413593	S	S	S	U
PRJNA413593	S	S	S	U
PRJNA413593	S	S	S	U

PRJNA413593	S	S	S	U
ERR2512688	S	S	S	S
ERR2514258	S	S	S	S
ERR2513507	S	S	S	S
ERR2513390	S	S	S	S
ERR2514077	S	S	S	S
ERR2513567	S	S	S	S
ERR2513856	S	S	S	S
ERR2512408	S	S	S	S
ERR2512959	S	S	S	S
ERR2512409	S	S	S	S
ERR2514919	S	S	S	S
ERR2514080	S	S	S	S
ERR2515020	S	S	S	S
ERR2513202	S	S	S	S
ERR2512712	S	S	S	S
ERR2514664	S	S	S	S
ERR2514286	S	S	S	S
ERR2512940	S	S	S	S
ERR2513377	S	S	S	S
ERR2512908	S	S	S	S
ERR2514064	S	S	S	S
ERR2514595	S	S	S	S
ERR2514713	S	S	S	S
ERR2513153	R	S	S	S
ERR2512586	S	S	S	S
ERR2513549	S	S	S	S
ERR2514054	S	S	S	S
ERR2512410	S	S	S	S
ERR2513461	S	S	S	S
ERR2512411	S	S	S	S
ERR2513189	S	S	S	S
ERR2513331	S	S	S	S
ERR2512412	S	S	S	S
ERR2514790	S	S	S	S
ERR2513347	S	S	S	S
ERR2514001	R	S	S	S
ERR2513369	S	S	S	S
ERR2512843	S	S	S	S
ERR2514842	R	S	S	S
ERR2513493	S	S	S	S
ERR2512451	S	S	S	S
ERR2514642	S	S	S	S
ERR2512431	S	S	S	S
ERR2513824	S	S	S	S
ERR2514307	S	S	S	S
ERR2513310	S	S	S	S
ERR2513234	S	S	S	S
ERR2514162	S	S	S	S
ERR2514663	S	S	S	S
ERR2512801	S	S	S	S
ERR2512413	S	S	S	S
ERR2513151	S	S	S	S

ERR2514042	S	S	S	S
ERR2514406	S	S	S	S
ERR2512414	S	S	S	S
ERR2514507	S	S	S	S
ERR2514570	S	S	S	S
ERR2514051	S	S	S	S
ERR2515008	S	S	S	S
ERR2514518	S	S	S	S
ERR2513527	S	S	S	S
ERR2512509	S	S	S	S
ERR2512456	R	R	S	S
ERR2514861	S	S	S	S
ERR2512670	S	S	S	S
ERR2513173	S	S	S	S
ERR2514082	S	S	S	S
ERR2514262	S	S	S	S
ERR2512415	S	S	S	S
ERR2515101	S	S	S	S
ERR2513881	S	S	S	S
ERR2513487	S	S	S	S
ERR2514139	S	S	S	S
ERR2514029	S	S	S	S
ERR2512905	S	S	S	S
ERR2512420	S	S	S	S
ERR2513634	S	S	S	S
ERR2513274	S	S	S	S
ERR2513363	S	S	S	S
ERR2513325	S	S	S	S
ERR2514019	S	S	S	S
ERR2513346	S	S	S	S
ERR2514127	S	S	S	S
ERR2513730	R	S	S	S
ERR2514353	S	S	S	S
ERR2513858	S	S	S	S
ERR2514965	S	S	S	S
ERR2514795	S	S	S	S
ERR2513241	R	R	S	R
ERR2513220	S	S	S	S
ERR2512428	S	S	S	S
ERR2514911	S	S	S	S
ERR2513844	S	S	S	S
ERR2513908	S	S	S	S
ERR2514438	S	S	S	S
ERR2513694	S	S	S	S
ERR2514197	S	S	S	S
ERR2514543	S	S	S	S
ERR2514071	S	S	S	S
ERR2513747	S	S	S	S
ERR2514958	S	S	S	S
ERR2513810	S	S	S	S
ERR2514629	R	S	S	S
ERR2513120	S	S	S	S
ERR2513175	S	S	S	S

ERR2512470	S	S	S	S
ERR2513585	S	S	S	S
ERR2513679	S	S	S	S
ERR2514694	S	S	S	S
ERR2513845	S	S	S	S
ERR2513442	S	S	S	S
ERR2514026	S	S	S	S
ERR2514202	S	S	S	S
ERR2514814	S	S	S	S
ERR2512685	S	S	S	S
ERR2513811	U	U	U	R
ERR2512836	S	S	S	S
ERR2512881	S	S	S	S
ERR2513288	S	S	S	S
ERR2514605	S	S	S	S
ERR2512459	S	S	S	S
ERR2514827	S	S	S	S
ERR2514846	S	S	S	S
ERR2512790	S	S	S	S
ERR2514871	S	S	S	S
ERR2515003	S	S	S	S
ERR2514504	S	S	S	S
ERR2513659	S	S	S	S
ERR2512480	S	S	S	S
ERR2513380	S	S	S	S
ERR2514085	S	S	S	S
ERR2512833	S	S	S	S
ERR2512723	S	S	S	S
ERR2514517	S	S	S	S
ERR2512745	S	S	S	S
ERR2513785	S	S	S	S
ERR2512488	S	S	S	S
ERR2513276	S	S	S	S
ERR2512963	S	S	S	S
ERR2514025	S	S	S	S
ERR2512486	S	S	S	S
ERR2514774	S	S	S	S
ERR2513402	S	S	S	S
ERR2514129	S	S	S	S
ERR2512516	S	S	S	S
ERR2514016	S	S	S	S
ERR2513304	S	S	S	S
ERR2514888	S	S	S	S
ERR2513329	S	S	S	S
ERR2514498	S	S	S	S
ERR2514935	R	S	S	S
ERR2513213	S	S	S	S
ERR2514980	S	S	S	S
ERR2514831	S	S	S	S
ERR2514577	S	S	S	S
ERR2512530	S	S	S	S
ERR2513554	S	S	S	S
ERR2512668	S	S	S	S

ERR2513733	S	S	S	S
ERR2513225	S	S	S	S
ERR2512521	S	S	S	S
ERR2512533	S	S	S	S
ERR2512868	R	S	S	S
ERR2512968	S	S	S	S
ERR2512540	S	S	S	S
ERR2514648	S	S	S	S
ERR2514265	S	S	S	S
ERR2513024	S	S	S	S
ERR2513933	S	S	S	S
ERR2512680	S	S	S	S
ERR2513650	S	S	S	S
ERR2513579	S	S	S	S
ERR2513639	S	S	S	S
ERR2514299	S	S	S	S
ERR2512819	S	S	S	S
ERR2513510	S	S	S	S
ERR2514128	S	S	S	S
ERR2512722	S	S	S	S
ERR2512547	S	S	S	S
ERR2513224	R	S	S	S
ERR2512505	S	S	S	S
ERR2514669	S	S	S	S
ERR2512628	S	S	S	S
ERR2514245	S	S	S	S
ERR2513804	S	S	S	S
ERR2513528	S	S	S	S
ERR2514028	S	S	S	S
ERR2514773	S	S	S	S
ERR2514593	S	S	S	S
ERR2513727	S	S	S	S
ERR2514850	S	S	S	S
ERR2512474	S	S	S	S
ERR2512560	S	S	S	S
ERR2515004	S	S	S	S
ERR2514978	S	S	S	S
ERR2512589	S	S	S	S
ERR2512597	S	S	S	S
ERR2514650	S	S	S	S
ERR2514860	S	S	S	S
ERR2512823	S	S	S	S
ERR2513486	S	S	S	S
ERR2512951	S	S	S	S
ERR2512616	S	S	S	S
ERR2513489	S	S	S	S
ERR2514355	S	S	S	S
ERR2512620	S	S	S	S
ERR2514825	S	S	S	S
ERR2514566	S	S	S	S
ERR2514588	S	S	S	S
ERR2512938	S	S	S	S
ERR2514141	S	S	S	S

ERR2513445	S	S	S	S
ERR2512609	S	S	S	S
ERR2512886	S	S	S	S
ERR2514153	S	S	S	S
ERR2513613	S	S	S	S
ERR2513901	S	S	S	S
ERR2514989	S	S	S	S
ERR2512623	S	S	S	S
ERR2515081	S	S	S	S
ERR2512758	S	S	S	S
ERR2512634	S	S	S	S
ERR2512831	S	S	S	S
ERR2514984	S	S	S	S
ERR2512971	S	S	S	S
ERR2512641	S	S	S	S
ERR2514341	S	S	S	S
ERR2514960	S	S	S	S
ERR2512663	S	S	S	S
ERR2512854	S	S	S	S
ERR2513519	S	S	S	S
ERR2513787	S	S	S	S
ERR2513885	S	S	S	S
ERR2512909	S	S	S	S
ERR2514531	S	S	S	S
ERR2512673	S	S	S	S
ERR2514893	S	S	S	S
ERR2512913	S	S	S	S
ERR2513290	S	S	S	S
ERR2513892	S	S	S	S
ERR2514131	S	S	S	S
ERR2514763	S	S	S	S
ERR2515099	R	S	S	S
ERR2512610	S	S	S	S
ERR2512542	S	S	S	S
ERR2514046	S	S	S	S
ERR2514671	S	S	S	S
ERR2514168	S	S	S	S
ERR2512691	R	R	S	U
ERR2513265	S	S	S	S
ERR2513846	S	S	S	S
ERR2512637	S	S	S	S
ERR2513726	S	S	S	S
ERR2512635	S	S	S	S
ERR2512946	S	S	S	S
ERR2514414	S	S	S	S
ERR2514034	S	S	S	S
ERR2514478	S	S	S	S
ERR2512697	S	S	S	S
ERR2512999	S	S	S	S
ERR2514401	S	S	S	S
ERR2513404	S	S	S	S
ERR2512902	S	S	S	S
ERR2513432	S	S	S	S

ERR2514678	S	S	S	S
ERR2514994	R	S	R	S
ERR2513485	S	S	S	S
ERR2514227	S	S	S	S
ERR2512469	S	S	S	S
ERR2514983	R	S	S	S
ERR2515067	S	S	S	S
ERR2514568	S	S	S	S
ERR2512795	S	S	S	S
ERR2512835	S	S	S	S
ERR2512695	S	S	S	S
ERR2512552	S	S	S	S
ERR2513615	S	S	S	S
ERR2513094	S	S	S	S
ERR2513671	U	U	U	S
ERR2514957	S	S	S	S
ERR2514571	S	S	S	S
ERR2512662	S	S	S	S
ERR2514878	S	S	S	S
ERR2513874	S	S	S	S
ERR2514338	S	S	S	S
ERR2513678	S	S	S	S
ERR2514997	S	S	S	S
ERR2512508	S	S	S	S
ERR2513465	S	S	S	S
ERR2513511	S	S	S	U
ERR2513587	S	S	S	S
ERR2512612	S	S	S	S
ERR2512992	S	S	S	S
ERR2515068	S	S	S	S
ERR2513940	S	S	S	S
ERR2512727	S	S	S	S
ERR2513359	S	S	S	S
ERR2513441	R	R	R	S
ERR2515019	S	S	S	S
ERR2513860	S	S	S	S
ERR2513797	S	S	S	S
ERR2512740	S	S	S	S
ERR2513208	S	S	S	S
ERR2513427	S	S	S	S
ERR2513599	S	S	S	S
ERR2512834	S	S	S	S
ERR2512701	S	S	S	S
ERR2512997	S	S	S	S
ERR2515050	S	S	S	S
ERR2513376	S	S	S	S
ERR2512491	S	S	S	S
ERR2512820	S	S	S	S
ERR2513692	S	S	S	S
ERR2513157	S	S	S	S
ERR2514484	S	S	S	S
ERR2512527	S	S	S	S
ERR2513111	S	S	S	S

ERR2512792	S	S	S	S
ERR2512703	S	S	S	S
ERR2514257	S	S	S	S
ERR2513676	S	S	S	S
ERR2512717	S	S	S	S
ERR2513259	S	S	S	S
ERR2515094	R	S	S	S
ERR2512455	S	S	S	S
ERR2512576	S	S	S	S
ERR2513103	S	S	S	S
ERR2512619	S	S	S	S
ERR2514388	U	U	U	S
ERR2514219	S	S	S	S
ERR2513990	S	S	S	S
ERR2513889	S	S	S	S
ERR2514729	R	S	S	S
ERR2514526	S	S	S	S
ERR2513736	S	S	S	S
ERR2513395	S	S	S	S
ERR2512918	S	S	S	S
ERR2513525	S	S	S	S
ERR2512464	S	S	S	S
ERR2514639	S	S	S	S
ERR2513049	S	S	S	S
ERR2514985	S	S	S	S
ERR2513813	S	S	S	S
ERR2514499	S	S	S	S
ERR2513612	S	S	S	S
ERR2512721	S	S	S	S
ERR2513353	S	S	S	S
ERR2512765	S	S	S	S
ERR2513557	S	S	S	S
ERR2512648	S	S	S	S
ERR2513471	S	S	S	S
ERR2514329	S	S	S	S
ERR2514876	S	S	S	S
ERR2512705	S	S	S	S
ERR2513068	S	S	S	S
ERR2513115	S	S	S	S
ERR2513913	S	S	S	S
ERR2512814	S	S	S	S
ERR2512561	S	S	S	S
ERR2514423	S	S	S	S
ERR2513763	S	S	S	S
ERR2513575	S	S	S	S
ERR2513287	S	S	S	S
ERR2515096	S	S	S	S
ERR2515018	S	S	S	S
ERR2513935	S	S	S	S
ERR2514192	S	S	S	S
ERR2513190	S	S	S	S
ERR2514636	S	S	S	S
ERR2512863	S	S	S	S

ERR2514434	S	S	S	S
ERR2514737	S	S	S	S
ERR2512744	S	S	S	S
ERR2514966	S	S	S	S
ERR2512827	S	S	S	S
ERR2514224	S	S	S	S
ERR2512896	S	S	S	S
ERR2514311	S	S	S	S
ERR2513555	S	S	S	S
ERR2512563	S	S	S	S
ERR2512869	S	S	S	S
ERR2514934	S	S	S	S
ERR2514918	S	S	S	S
ERR2513926	S	S	S	S
ERR2512755	S	S	S	R
ERR2512927	S	S	S	S
ERR2512427	S	S	S	S
ERR2514698	S	S	S	S
ERR2512966	S	S	S	S
ERR2514731	S	S	S	S
ERR2514597	S	S	S	S
ERR2514799	S	S	S	S
ERR2515072	R	S	S	S
ERR2514721	S	S	S	S
ERR2513714	S	S	S	S
ERR2512878	R	R	S	R
ERR2513003	S	S	S	S
ERR2512840	S	S	S	S
ERR2513286	S	S	S	S
ERR2513280	S	S	S	S
ERR2513081	S	S	S	S
ERR2514779	S	S	S	U
ERR2514725	S	S	S	S
ERR2514271	S	S	S	S
ERR2512731	S	S	S	S
ERR2512622	S	S	S	S
ERR2512888	R	S	S	S
ERR2513236	S	S	S	S
ERR2515090	S	S	S	S
ERR2512950	S	S	S	S
ERR2514360	S	S	S	S
ERR2513769	S	S	S	S
ERR2513414	S	S	S	S
ERR2513938	S	S	S	S
ERR2513239	S	S	S	S
ERR2514909	R	S	S	S
ERR2512600	R	S	S	S
ERR2512479	S	S	S	S
ERR2513474	S	S	S	S
ERR2512651	S	S	S	S
ERR2513308	S	S	S	S
ERR2514264	S	S	S	S
ERR2514720	S	S	S	S

ERR2514584	S	S	S	S
ERR2513614	S	S	S	S
ERR2512501	S	S	S	S
ERR2513235	S	S	S	S
ERR2514996	S	S	S	U
ERR2512678	S	S	S	S
ERR2512861	S	S	S	S
ERR2514611	S	S	S	S
ERR2512973	S	S	S	S
ERR2512984	S	S	S	S
ERR2514114	S	S	S	S
ERR2514185	S	S	S	S
ERR2512786	S	S	S	S
ERR2513865	S	S	S	S
ERR2514321	S	S	S	S
ERR2512981	S	S	S	S
ERR2514254	S	S	S	S
ERR2513017	S	S	S	S
ERR2515005	S	S	S	S
ERR2514464	S	S	S	S
ERR2512916	S	S	S	S
ERR2514889	S	S	S	S
ERR2513899	S	S	S	S
ERR2513906	S	S	S	S
ERR2512555	S	S	S	S
ERR2514212	S	S	S	S
ERR2514942	S	S	S	S
ERR2513424	S	S	S	S
ERR2514625	S	S	S	S
ERR2513722	S	S	S	S
ERR2514179	S	S	S	S
ERR2514607	S	S	S	S
ERR2513375	S	S	S	S
ERR2514152	S	S	S	S
ERR2514465	S	S	S	S
ERR2514632	R	S	S	S
ERR2513967	S	S	S	S
ERR2513277	S	S	S	S
ERR2513988	S	S	S	S
ERR2513706	S	S	S	S
ERR2513482	S	S	S	S
ERR2514781	S	S	S	S
ERR2512432	S	S	S	S
ERR2515058	S	S	S	S
ERR2513606	S	S	S	S
ERR2513099	R	S	S	S
ERR2512426	S	S	S	S
ERR2514674	S	S	S	S
ERR2513019	S	S	S	S
ERR2512998	S	S	S	S
ERR2513530	S	S	S	S
ERR2514585	S	S	S	S
ERR2512874	S	S	S	S

ERR2512852	S	S	S	S
ERR2513833	S	S	S	S
ERR2514031	S	S	S	S
ERR2514155	R	S	R	S
ERR2512995	S	S	S	U
ERR2514707	S	S	S	S
ERR2514358	S	S	S	S
ERR2514172	S	S	S	S
ERR2514134	S	S	S	S
ERR2514863	S	S	S	S
ERR2513021	S	S	S	S
ERR2514532	S	S	S	S
ERR2514734	S	S	S	S
ERR2513418	S	S	S	S
ERR2513028	S	S	S	S
ERR2512805	S	S	S	S
ERR2512440	R	R	S	R
ERR2513033	S	S	S	S
ERR2514440	S	S	S	S
ERR2514448	S	S	S	S
ERR2514169	S	S	S	S
ERR2513898	R	S	S	S
ERR2513595	S	S	S	S
ERR2514079	S	S	S	S
ERR2514751	S	S	S	S
ERR2513222	S	S	S	S
ERR2514419	S	S	S	S
ERR2514429	S	S	S	S
ERR2514915	S	S	S	S
ERR2514931	S	S	S	S
ERR2514626	S	S	S	S
ERR2514826	S	S	S	S
ERR2513035	S	S	S	S
ERR2513199	S	S	S	S
ERR2513066	S	S	S	S
ERR2514844	S	S	S	S
ERR2514489	S	S	S	S
ERR2514260	S	S	S	S
ERR2513132	S	S	S	S
ERR2513315	S	S	S	S
ERR2512915	S	S	S	S
ERR2514519	S	S	S	S
ERR2514845	S	S	S	S
ERR2514467	S	S	S	S
ERR2514868	S	S	S	S
ERR2512640	S	S	S	S
ERR2513278	S	S	S	S
ERR2513105	S	S	S	S
ERR2514285	S	S	S	R
ERR2513761	S	S	S	S
ERR2515073	S	S	S	S
ERR2515031	S	S	S	S
ERR2512499	S	S	S	S

ERR2513616	S	S	S	S
ERR2513134	S	S	S	S
ERR2513227	S	S	S	S
ERR2513675	S	S	S	S
ERR2512763	S	S	S	S
ERR2513388	S	S	S	S
ERR2513791	S	U	S	S
ERR2512626	S	U	S	S
ERR2512452	S	S	S	S
ERR2512710	S	S	S	S
ERR2512595	R	S	S	S
ERR2513745	S	S	S	S
ERR2512565	S	S	S	S
ERR2513263	S	S	S	S
ERR2515029	S	S	S	S
ERR2514670	S	S	S	S
ERR2514203	S	S	S	S
ERR2513219	R	S	S	S
ERR2512506	S	S	S	S
ERR2513122	R	S	S	S
ERR2513046	S	S	S	S
ERR2513281	S	S	S	S
ERR2514209	S	S	S	S
ERR2513937	S	S	S	S
ERR2513282	S	S	S	S
ERR2512798	S	S	S	S
ERR2513484	S	S	S	S
ERR2514004	S	S	S	S
ERR2513405	S	S	S	S
ERR2513362	S	S	S	S
ERR2512671	S	S	S	S
ERR2512614	S	S	S	S
ERR2513119	S	S	S	S
ERR2515043	S	S	S	S
ERR2513178	S	S	S	S
ERR2514472	S	S	S	S
ERR2513047	R	S	S	S
ERR2515002	S	S	S	S
ERR2513073	S	S	S	S
ERR2513444	S	S	S	S
ERR2513407	S	S	S	S
ERR2512903	S	S	S	S
ERR2514987	S	S	S	S
ERR2513689	S	S	S	S
ERR2513931	S	S	S	S
ERR2513156	S	S	S	S
ERR2513267	R	S	S	S
ERR2514792	S	S	S	S
ERR2514967	S	S	S	S
ERR2514289	S	S	S	S
ERR2515010	S	S	S	S
ERR2514865	S	S	S	S
ERR2512953	S	S	S	S

ERR2514986	S	S	S	S
ERR2512845	S	S	S	S
ERR2514927	S	S	S	S
ERR2514917	S	S	S	S
ERR2513855	S	S	S	S
ERR2514268	S	S	S	S
ERR2513029	S	S	S	S
ERR2513095	S	S	S	S
ERR2514634	S	S	S	S
ERR2513209	S	S	S	S
ERR2513703	S	S	S	S
ERR2512446	S	S	S	S
ERR2513268	S	S	S	S
ERR2513215	S	S	S	S
ERR2514726	S	S	S	S
ERR2514433	S	S	S	S
ERR2513226	S	S	S	S
ERR2514802	S	S	S	S
ERR2514333	S	S	S	S
ERR2513570	S	S	S	S
ERR2512895	S	S	S	S
ERR2513720	S	S	S	R
ERR2512424	S	S	S	S
ERR2514214	S	S	S	S
ERR2513477	S	S	S	S
ERR2514324	S	S	S	S
ERR2515014	U	U	U	S
ERR2514912	S	S	S	S
ERR2514892	S	S	S	S
ERR2513230	S	S	S	S
ERR2514950	S	S	S	S
ERR2514939	S	S	S	S
ERR2513246	S	S	S	S
ERR2513890	S	S	S	S
ERR2514970	S	S	S	S
ERR2513927	S	S	S	S
ERR2513732	S	S	S	S
ERR2512502	S	S	S	S
ERR2513069	S	S	S	S
ERR2513930	S	S	S	S
ERR2512567	S	S	S	S
ERR2514436	S	S	S	S
ERR2514248	S	S	S	S
ERR2513249	S	S	S	S
ERR2514244	R	S	S	S
ERR2512764	S	S	S	S
ERR2513174	S	S	S	S
ERR2513864	S	S	S	S
ERR2512885	S	S	S	S
ERR2513475	S	S	S	S
ERR2513715	S	S	S	S
ERR2512481	S	S	S	S
ERR2513623	S	S	S	S

ERR2512468	U	S	S	S
ERR2514786	S	S	S	S
ERR2514596	S	S	S	S
ERR2513139	S	S	S	S
ERR2513039	S	S	S	S
ERR2513997	S	S	S	S
ERR2513114	S	S	S	S
ERR2513869	S	S	S	S
ERR2514163	S	S	S	S
ERR2512945	S	S	S	S
ERR2514833	S	S	S	S
ERR2513252	S	S	S	S
ERR2512417	S	S	S	S
ERR2514830	S	S	S	S
ERR2512652	S	S	S	S
ERR2513687	S	S	S	S
ERR2514147	S	S	S	S
ERR2513958	S	S	S	S
ERR2513998	R	S	S	S
ERR2514945	R	S	S	S
ERR2512865	S	S	S	S
ERR2514267	R	R	S	S
ERR2514843	S	S	S	S
ERR2512887	S	S	S	S
ERR2512669	S	S	S	S
ERR2512596	R	R	S	U
ERR2514089	S	S	S	S
ERR2513129	S	S	S	S
ERR2514103	S	S	S	S
ERR2514132	S	S	S	S
ERR2513985	S	S	S	S
ERR2513968	S	S	S	S
ERR2514156	R	S	S	S
ERR2514049	S	S	S	S
ERR2513851	S	S	S	S
ERR2514336	S	S	S	S
ERR2513357	S	S	S	S
ERR2512706	S	S	S	S
ERR2513012	S	S	S	S
ERR2513916	S	S	S	S
ERR2513809	S	S	S	S
ERR2513503	S	S	S	S
ERR2513295	R	R	R	U
ERR2513336	S	S	S	S
ERR2512707	S	S	S	S
ERR2512664	S	S	S	U
ERR2513123	S	S	S	S
ERR2512759	S	S	S	S
ERR2513560	S	S	S	S
ERR2514895	S	S	S	S
ERR2514762	S	S	S	S
ERR2512687	S	S	S	S
ERR2513637	S	S	S	S

ERR2513821 S S S S
ERR552266 S S S S
ERR552501,ERR552500 R R R R
ERR2199824 ERR2199825 S S S S
ERR137212 R S S S
ERR551727,ERR551726 R R R S
ERR551923,ERR551922 R R R R
ERR551305,ERR551304 R R R R
ERR553163,ERR553161,ERR553162 R R S S
ERR553291 S S S S
ERR552708,ERR552709 R R R R
ERR550735,ERR550736 R R R R
ERR2516313 R R S R
ERR551409,ERR551410 R R S S
ERR1768629 R R R R
ERS458560 R S S S
ERR2199826 ERR2199827 S S S S
ERR551268 S S S S
ERR108503 R R R S
ERS458209 R R R S
ERR108504 S S R S
ERS458677 S S S S
ERR551368 S S S S
ERR137213 R S S S
ERR137214 S S S S
ERR551934 S S S S
ERR552062 S S S S
ERR137215 R R S S
ERR552831 S S S S
ERR552459 R S S S
ERR117470 R R R S
ERR137216 R S S R
ERR551327 S S S S
ERR234595 R S S S
ERR137218 R R R S
ERR234603 S S S S
ERR137220 R R R S
ERR234619 S S S S
ERR137221 R S S S
ERR553238 R R R S
ERR551916 S S S S
ERR234627 R R S S
ERR234635 S S S S
ERR553065 S S S S
ERR2199828 S S S S
ERR137222 R R S S
ERR137223 R R R S
ERR137224 S S S S
ERR552752 S S S S
ERR2516281 R R R R
ERR2516308 R R R R
ERR2516345 R R S R
ERR2516245 R R R S

ERR2516336	R	R	R	R
ERR2516351	R	R	S	S
ERR2516203	R	R	S	S
ERR2516321	S	S	S	S
ERR2516334	S	S	S	S
ERR2516170	S	S	S	S
ERR2516283	S	S	S	S
ERR2516355	S	S	S	S
ERR2516239	R	R	R	R
ERR2516213	R	R	R	R
ERR2516277	S	S	S	S
ERR2516337	S	S	S	S
ERR2516233	S	S	S	S
ERR2516200	S	S	S	S
ERR2516292	S	S	S	S
ERR2516393	S	S	S	S
ERR2516189	S	S	S	R
ERR2516195	S	S	S	S
ERR2516169	S	S	S	S
ERR2516326	S	S	S	S
ERR2516363	S	S	S	S
ERR2516215	S	S	S	R
ERR2516279	S	S	S	S
ERR2516346	S	S	S	S
ERR2516264	R	R	R	R
ERR2516173	S	S	S	S
ERR2516290	R	S	S	S
ERR2516310	S	S	S	S
ERR2516386	S	S	S	S
ERR2516284	S	S	S	S
ERR2516398	S	S	S	R
ERR2516342	S	S	S	S
ERR2516314	S	S	S	S
ERR2516201	S	S	S	S
ERR2516276	S	S	S	S
ERR2516171	S	S	S	S
ERR2516191	S	S	S	R
ERR2516168	S	S	S	S
ERR2516394	S	S	S	R
ERR2516385	S	S	S	S
ERR2516339	S	S	S	R
ERR2516228	S	S	S	S
ERR2516350	S	S	S	S
ERR2516244	S	S	S	S
ERR2516315	S	S	S	S
ERR2516366	S	S	S	S
ERR2516273	S	S	S	R
ERR2516235	S	S	S	S
ERR2516379	S	S	S	S
ERR2516259	S	S	S	S
ERR2516372	S	S	S	S
ERR2516323	S	S	S	S
ERR2516278	S	S	S	S

ERR2516275	S	S	S	S
ERR2516204	S	S	S	S
ERR2516360	S	S	S	S
ERR2516328	S	S	S	S
ERR2516376	S	S	S	R
ERR2516312	S	S	S	S
ERR2516383	S	S	S	S
ERR2516267	S	S	S	S
ERR2516294	S	S	S	S
ERR2516216	S	S	S	S
ERR2516223	S	S	S	S
ERR2516369	S	S	S	S
ERR2516299	S	S	S	S
ERR2516172	S	S	S	S
ERR2516272	S	S	S	S
ERR2516193	S	S	S	S
ERR2516370	S	S	S	S
ERR2516384	S	S	S	S
ERR2516332	S	S	S	S
ERR2516224	S	S	S	S
ERR2516260	S	S	S	S
ERR2516197	S	S	S	S
ERR2516348	S	S	S	S
ERR2516380	S	S	S	S
ERR2516225	S	S	S	S
ERR2516248	S	S	S	S
ERR2516242	S	S	S	S
ERR2516179	S	S	S	S
ERR2516236	S	S	S	S
ERR2516178	R	R	S	S
ERR2516241	S	S	S	S
ERR2516212	S	S	S	S
ERR2516210	S	S	S	S
ERR2516227	S	S	S	S
ERR2516362	S	S	S	S
ERR2516184	S	S	S	S
ERR2516319	S	S	S	S
ERR2516205	S	S	S	S
ERR2516305	S	S	S	S
ERR2516306	S	S	S	S
ERR2516293	S	S	S	S
ERR2516174	S	S	S	S
ERR2516230	R	R	R	R
ERR2516218	S	S	S	S
ERR2516250	S	S	S	S
ERR2516322	S	S	S	S
ERR2516194	S	S	S	S
ERR2516207	S	S	S	R
ERR2516300	S	S	S	S
ERR2516316	R	R	S	R
ERR2516371	S	S	S	S
ERR2516353	S	S	S	S
ERR2516269	S	S	S	S

ERR2516341	S	S	S	S
ERR2516318	S	S	S	S
ERR2516246	S	S	S	S
ERR2516307	S	S	S	R
ERR2516262	S	S	S	R
ERR2516335	S	S	S	R
ERR2516324	S	S	S	S
ERR2516340	R	R	R	S
ERR2516258	S	S	S	S
ERR2516202	S	S	S	S
ERR2516378	S	S	S	S
ERR2516388	S	S	S	S
ERR2516374	S	S	S	S
ERR2516381	S	S	S	S
ERR2516238	S	S	S	S
ERR2516354	S	S	S	S
ERR2516298	S	S	S	S
ERR2516234	S	S	S	S
ERR2516358	S	S	S	S
ERR2516211	S	S	S	S
ERR2516268	S	S	S	S
ERR2516317	S	S	S	S
ERR2516309	S	S	S	S
ERR2516247	S	S	S	S
ERR2516304	S	S	S	S
ERR2516331	S	S	S	S
ERR2516231	S	S	S	S
ERR2516296	S	S	S	S
ERR2516221	S	S	S	S
ERR2516180	S	S	S	S
ERR2516253	S	S	S	S
ERR2516311	S	S	S	R
ERR2516265	S	S	S	S
ERR2516187	S	S	S	S
ERR2516395	S	S	S	S
ERR2516185	S	S	S	S
ERR2516240	S	S	S	S
ERR2516368	S	S	S	S
ERR2516222	S	S	S	S
ERR2516338	R	R	S	R
ERR2516288	R	R	R	R
PRJNA413593	S	S	S	U
PRJNA413593	S	S	S	U
PRJNA413593	S	S	S	U
PRJNA413593	S	S	S	U
PRJNA413593	S	S	S	U
PRJNA413593	S	S	S	U
PRJNA413593	S	S	S	U
PRJNA413593	S	S	S	U
PRJNA413593	S	S	S	U
PRJNA413593	S	S	S	U
PRJNA413593	S	S	S	U
PRJNA413593	S	S	S	U

PRJNA413593	S	S	S	U
PRJNA413593	S	S	S	S
PRJNA413593	R	S	S	S
PRJNA413593	S	S	S	U
PRJNA413593	S	S	S	U
PRJNA413593	R	S	S	S
PRJNA413593	R	S	S	S
PRJNA413593	R	S	S	S
PRJNA413593	S	S	S	U
PRJNA413593	S	S	S	U
PRJNA413593	S	S	S	U
PRJNA413593	R	S	S	S
PRJNA413593	S	S	S	U
PRJNA413593	S	S	S	U
PRJNA413593	R	S	S	S
PRJNA413593	S	S	S	U
PRJNA413593	S	S	S	U
PRJNA413593	S	S	S	U
PRJNA413593	S	S	S	U
PRJNA413593	S	S	S	U
PRJNA413593	R	S	S	S
PRJNA413593	S	S	S	U
PRJNA413593	S	S	S	U
PRJNA413593	S	S	S	U
PRJNA413593	S	S	S	U
PRJNA413593	S	S	S	U
PRJNA413593	S	S	S	U
PRJNA413593	S	S	S	U
PRJNA413593	S	S	S	U
PRJNA413593	S	S	S	U
PRJNA413593	S	S	S	U
PRJNA413593	R	S	S	S
PRJNA413593	S	S	S	U
PRJNA413593	S	S	S	U
PRJNA413593	S	S	S	U
PRJNA413593	S	S	S	U
PRJNA413593	S	S	S	U
PRJNA413593	S	S	S	U
PRJNA413593	R	S	S	S
PRJNA413593	R	S	S	S
PRJNA413593	S	S	S	U
PRJNA413593	S	S	S	U
PRJNA413593	S	S	S	U
PRJNA413593	S	S	S	U
PRJNA413593	R	S	S	S
PRJNA413593	S	S	S	U
PRJNA413593	S	S	S	U
PRJNA413593	S	S	S	U
PRJNA413593	S	S	S	U
PRJNA413593	R	S	S	S
PRJNA413593	S	S	S	U
PRJNA413593	S	S	S	U
PRJNA413593	S	S	S	U

PRJNA413593	S	S	S	U
PRJNA413593	S	S	S	U
PRJNA413593	S	S	S	U
PRJNA413593	S	S	S	U
PRJNA413593	S	S	S	U
PRJNA413593	S	S	S	U
PRJNA413593	S	S	S	U
PRJNA413593	S	S	S	U
PRJNA413593	S	S	S	U
PRJNA413593	S	S	S	U
PRJNA413593	S	S	S	U
PRJNA413593	S	S	S	U
PRJNA413593	S	S	S	U
PRJNA413593	R	S	S	S
PRJNA413593	S	S	S	U
PRJNA413593	S	S	S	U
PRJNA413593	R	S	S	S
PRJNA413593	S	S	S	U
PRJNA413593	S	S	S	U
PRJNA413593	S	S	S	U
PRJNA413593	S	S	S	U
PRJNA413593	R	S	S	S
PRJNA413593	S	S	S	U
PRJNA413593	S	S	S	U
PRJNA413593	S	S	S	U
PRJNA413593	S	S	S	U
PRJNA413593	S	S	S	U
PRJNA413593	S	S	S	U
PRJNA413593	S	S	S	U
PRJNA413593	S	S	S	U
PRJNA413593	S	S	S	U
PRJNA413593	S	S	S	U
PRJNA413593	S	S	S	U
PRJNA413593	S	S	S	U
PRJNA413593	S	S	S	U
PRJNA413593	S	S	S	U
PRJNA413593	S	S	S	U
PRJNA413593	S	S	S	U
PRJNA413593	S	S	S	U
PRJNA413593	R	S	S	S
PRJNA413593	S	S	S	U
PRJNA413593	S	S	S	U
PRJNA413593	S	S	S	U
PRJNA413593	S	S	S	U
PRJNA413593	R	S	S	S
PRJNA413593	S	S	S	U
PRJNA413593	S	S	S	U
PRJNA413593	S	S	S	U
PRJNA413593	S	S	S	U
PRJNA413593	R	S	S	S
PRJNA413593	S	S	S	U
PRJNA413593	S	S	S	U
PRJNA413593	S	S	S	U

ERR2512906	S	S	S	S
ERR2513505	S	S	S	S
ERR2513162	S	S	S	S
ERR2512472	S	S	S	S
ERR2513351	S	S	S	S
ERR2512810	S	S	S	S
ERR2514644	S	S	S	S
ERR2514612	S	S	S	S
ERR2514313	S	S	S	S
ERR2513327	S	S	S	S
ERR2513010	S	S	S	S
ERR2512844	S	S	S	S
ERR2513093	S	S	S	S
ERR2514176	S	S	S	S
ERR2512760	S	S	S	S
ERR2513558	S	S	S	S
ERR2513271	S	S	S	S
ERR2514693	S	S	S	S
ERR2514541	S	S	S	S
ERR2513244	S	S	S	S
ERR2513741	S	S	S	S
ERR2513305	S	S	S	S
ERR2515085	S	S	S	S
ERR2512605	S	S	S	S
ERR2514875	S	S	S	S
ERR2514149	S	S	S	S
ERR2514820	S	S	S	S
ERR2513150	S	S	S	S
ERR2514470	S	S	S	S
ERR2512746	S	S	S	R
ERR2514696	S	S	S	S
ERR2513141	S	S	S	S
ERR2514879	S	S	S	S
ERR2514769	S	S	S	S
ERR2514787	S	S	S	S
ERR2513480	S	S	S	S
ERR2512683	S	S	S	S
ERR2513142	S	S	S	S
ERR2514062	S	S	S	S
ERR2512537	S	S	S	S
ERR2514849	S	S	S	S
ERR2513960	S	S	S	S
ERR2512692	S	S	S	S
ERR2513753	S	S	S	S
ERR2513314	S	S	S	S
ERR2515044	S	S	S	S
ERR2513980	S	S	S	S
ERR2512475	S	S	S	S
ERR2513393	S	S	S	S
ERR2513577	S	S	S	S
ERR2513110	S	S	S	S
ERR2513055	S	S	S	S
ERR2512639	S	S	S	S

ERR2514015	S	S	S	S
ERR2513333	S	S	S	S
ERR2514490	S	S	S	S
ERR2513823	S	S	S	S
ERR2512574	S	S	S	S
ERR2514235	S	S	S	S
ERR2513818	S	S	S	S
ERR2514273	S	S	S	S
ERR2513541	S	S	S	S
ERR2514449	S	S	S	S
ERR2512778	S	S	S	S
ERR2514297	S	S	S	S
ERR2514581	S	S	S	S
ERR2514239	S	S	S	S
ERR2513608	R	R	S	R
ERR2514637	S	S	S	S
ERR2512513	S	S	S	S
ERR2513537	S	S	S	S
ERR2513128	S	S	S	S
ERR2513337	S	S	S	S
ERR2513740	S	S	S	S
ERR2512894	S	S	S	S
ERR2513754	S	S	S	S
ERR2513674	S	S	S	S
ERR2512649	S	S	S	S
ERR2513367	S	S	S	S
ERR2514809	S	S	S	S
ERR2513372	S	S	S	S
ERR2512952	S	S	S	S
ERR2513127	S	S	S	S
ERR2514096	S	S	S	S
ERR2514718	S	S	S	S
ERR2514100	S	S	S	S
ERR2513979	S	S	S	S
ERR2513203	S	S	S	S
ERR2514916	S	S	S	S
ERR2514736	S	S	S	S
ERR2512806	S	S	S	S
ERR2513543	S	S	S	S
ERR2513382	S	S	S	S
ERR2512893	S	S	S	S
ERR2514560	S	S	S	S
ERR2512690	S	S	S	S
ERR2514287	S	S	S	S
ERR2514972	S	S	S	S
ERR2513118	S	S	S	S
ERR2512645	S	S	S	S
ERR2512581	S	S	S	S
ERR2512996	S	S	S	S
ERR2513863	S	S	S	S
ERR2513591	S	S	S	S
ERR2513602	S	S	S	S
ERR2514801	S	S	S	S

ERR2514160	S	S	S	S
ERR2514447	S	S	S	S
ERR2512709	S	S	S	S
SAMN05276505	S	S	S	S
ERR2512587	S	S	S	S
ERR2513391	S	S	S	S
ERR2512729	S	S	S	S
ERR2514563	S	S	S	S
ERR2513410	S	S	S	S
ERR2514221	S	S	S	S
ERR2513218	R	S	S	S
ERR2512856	S	S	S	S
ERR2513330	S	S	S	S
ERR2512751	S	S	S	S
ERR2513366	S	S	S	S
ERR2513644	S	S	S	S
ERR2514294	S	S	S	S
ERR2513449	S	S	S	S
ERR2513060	S	S	S	S
ERR2514362	U	U	U	S
ERR2513205	S	S	S	S
ERR2514523	S	S	S	S
ERR2512686	S	S	S	S
ERR2514430	S	S	S	S
ERR2514363	S	S	S	S
ERR2513439	S	S	S	S
ERR2514445	S	S	S	S
ERR2513887	S	S	S	S
ERR2515006	S	S	S	S
ERR2514682	S	S	S	S
ERR2513191	S	S	S	S
ERR2512672	S	S	S	S
ERR2514572	S	S	S	S
ERR2514386	S	S	S	S
ERR2513387	S	S	S	S
ERR2512830	S	S	S	S
ERR2513147	S	S	S	S
ERR2512702	S	S	S	S
ERR2514397	S	S	S	S
ERR2514331	S	S	S	S
ERR2514564	R	S	S	S
ERR2512681	S	S	S	S
ERR2513086	S	S	S	S
ERR2512989	S	S	S	S
ERR2512618	S	S	S	S
ERR2513545	S	S	S	S
ERR2513207	S	S	S	S
ERR2513400	S	S	S	S
ERR2513196	S	S	S	S
ERR2513429	S	S	S	S
ERR2514654	S	S	S	S
ERR2515074	S	S	S	S
ERR2514505	S	S	S	S

ERR2514937	S	S	S	S
ERR2514586	S	S	S	S
ERR2514534	S	S	S	S
ERR2513849	S	S	S	S
ERR2514314	S	S	S	S
ERR2513403	S	S	S	S
ERR2513827	S	S	S	S
ERR2514047	S	S	S	S
ERR2513768	S	S	S	S
ERR2512473	S	S	S	S
ERR2514094	R	S	S	S
ERR2512559	S	S	S	S
ERR2513610	S	S	S	S
ERR2514492	S	S	S	S
ERR2512899	S	S	S	S
ERR2513062	S	S	S	S
ERR2512803	S	S	S	S
ERR2513772	S	S	S	S
ERR2513409	S	S	S	S
ERR2514405	S	S	S	S
ERR2514036	S	S	S	S
ERR2513411	S	S	S	S
ERR2514282	S	S	S	S
ERR2513322	S	S	S	S
ERR2513131	S	S	S	S
ERR2515084	S	S	S	S
ERR2512463	S	S	S	S
ERR2512939	S	S	S	S
ERR2514382	S	S	S	S
ERR2513030	S	S	S	S
ERR2513309	S	S	S	S
ERR2514573	S	S	S	S
ERR2515098	R	S	S	S
ERR2512564	S	S	S	S
ERR2513198	S	S	S	S
ERR2512977	S	S	S	S
ERR2515095	S	S	S	S
ERR2514883	S	S	S	S
ERR2515091	S	S	S	U
ERR2514582	S	S	S	S
ERR2514383	S	S	S	S
ERR2513825	S	S	S	S
ERR2514544	S	S	S	S
ERR2514370	S	S	S	S
ERR2514780	S	S	S	S
ERR2513752	R	S	S	S
ERR2512617	S	S	S	S
ERR2512518	S	S	S	S
ERR2515009	S	S	S	S
ERR2514785	S	S	S	S
ERR2512785	S	S	S	S
ERR2513421	S	S	S	S
ERR2512603	S	S	S	S

ERR2514296	S	S	S	S
ERR2512507	S	S	S	S
ERR2514732	S	S	S	S
ERR2514243	S	S	S	S
ERR2513876	S	S	S	S
ERR2513040	S	S	S	S
ERR2515025	S	S	S	S
ERR2512800	S	S	S	S
ERR2514951	S	S	S	S
ERR2513385	S	S	S	S
ERR2512650	S	S	S	S
ERR2513136	S	S	S	S
ERR2513464	S	S	S	S
ERR2512919	S	S	S	S
ERR2513777	S	S	S	S
ERR2514715	S	S	S	S
ERR2513469	S	S	S	S
ERR2513197	S	S	S	S
ERR2513299	S	S	S	S
ERR2514993	S	S	S	S
ERR2512676	S	S	S	R
ERR2513604	S	S	S	S
ERR2513386	S	S	S	S
ERR2514392	S	S	S	S
ERR2514300	S	S	S	S
ERR2513514	S	S	S	S
ERR2513279	S	S	S	S
ERR2515075	S	S	S	S
ERR2513460	R	R	R	R
ERR2513807	R	S	S	S
ERR2513522	S	S	S	S
ERR2512510	S	S	S	S
ERR2514189	S	S	S	S
ERR2513321	S	S	S	S
ERR2513041	S	S	S	S
ERR2513446	S	S	S	S
ERR2513532	S	S	S	S
ERR2512575	S	S	S	S
ERR2513562	S	S	S	S
ERR2514462	S	S	S	S
ERR2512730	R	R	R	R
ERR2513188	S	S	S	S
ERR2512822	S	S	S	S
ERR2513564	R	R	S	R
ERR2514052	S	S	S	S
ERR2514444	S	S	S	S
ERR2514074	S	S	S	S
ERR2514458	S	S	S	S
ERR2512932	S	S	S	S
ERR2514237	S	S	S	S
ERR2513583	S	S	S	S
ERR2513100	S	S	S	S
ERR2513800	S	S	S	S

ERR2515024	R	S	S	S
SAMN05276400	S	S	S	S
ERR2512930	R	R	S	S
ERR2514356	S	S	S	S
ERR2514266	S	S	S	S
ERR2515033	S	S	S	S
ERR2512582	R	S	S	S
ERR2513266	S	S	S	S
SAMN05276482	S	S	S	S
ERR2512454	S	S	S	S
ERR2513660	S	S	S	S
SAMN05276469	S	S	S	S
SAMN05276305	S	S	S	S
ERR2514252	S	S	S	S
ERR2513192	S	S	S	S
ERR2513285	S	S	S	S
ERR2512969	S	S	S	S
SAMN05276412	S	S	S	S
ERR2514982	R	S	S	R
SAMN05276426	S	S	S	S
SAMN05276431	S	S	S	S
SAMN05276548	S	S	S	S
SAMN05276318	S	S	S	S
SAMN05276556	S	S	S	S
ERR2512425	S	S	S	S
SAMN05276450	S	S	S	S
SAMN05276378	R	R	S	S
ERR2514894	S	S	S	S
SAMN05276311	R	R	R	S
ERR2514800	R	S	S	S
SAMN05276513	S	S	S	S
ERR2514940	S	S	S	S
SAMN05276309	S	S	S	S
SAMN05276558	S	S	S	S
ERR2514081	S	S	S	S
SAMN05276489	S	S	S	S
ERR2514123	R	R	R	S
SAMN05276312	R	S	R	R
ERR2512602	S	S	S	S
ERR2514463	S	S	S	S
SAMN05276536	S	S	S	S
ERR2513453	S	S	S	S
SAMN05276471	S	S	S	S
SAMN05276547	S	S	S	S
ERR2513531	S	S	S	S
ERR2514417	S	S	S	S
ERR2513756	S	S	S	S
ERR2512471	S	S	S	S
ERR2513253	S	S	S	S
ERR2514622	S	S	S	S
ERR2513518	S	S	S	S
ERR2514295	R	S	S	S
ERR2514807	S	S	S	S

ERR2514164	S	S	S	S
ERR2514279	S	S	S	S
ERR2514933	S	S	S	S
ERR2514159	R	R	S	S
ERR2512942	S	S	S	S
ERR2513080	S	S	S	S
ERR2514810	S	S	S	S
ERR2513759	S	S	S	S
ERR2514130	S	S	S	S
ERR2513949	S	S	S	S
ERR2513624	S	S	S	S
ERR2514591	U	U	U	S
ERR2512529	S	S	S	S
ERR2512956	S	S	S	R
ERR2513201	S	S	S	S
ERR2513691	S	S	S	S
ERR2513838	S	S	S	S
ERR2514897	S	S	S	S
ERR2513185	S	S	S	S
ERR2513649	S	S	S	S
ERR2513072	S	S	S	S
ERR2512644	S	S	S	S
ERR2513247	S	S	S	S
ERR2513023	S	S	S	S
ERR2514005	R	R	S	S
ERR2513551	S	S	S	S
ERR2514238	S	S	S	S
ERR2514136	S	S	S	S
ERR2512647	S	S	S	R
ERR2513775	S	S	S	S
ERR2514008	R	S	S	R
ERR2513987	R	R	S	S
ERR2514140	S	S	S	S
ERR2513145	R	S	S	S
ERR2513792	S	S	S	S
ERR2514365	S	S	S	S
ERR2514379	S	S	S	U
ERR2514906	S	S	S	S
ERR2514768	S	S	S	S
ERR2512978	S	S	S	S
ERR2514524	S	S	S	S
ERR2514621	S	S	S	S
ERR2514441	S	S	S	S
ERR2514784	S	S	S	S
ERR2513788	S	S	S	S
ERR2512796	S	S	S	S
ERR2513053	S	S	S	S
ERR2514956	S	S	S	S
ERR2514699	S	S	S	S
ERR2513992	S	S	S	S
ERR2514041	S	S	S	S
ERR2514304	S	S	S	S
ERR2512982	S	S	S	S

ERR2514948	S	S	S	R
ERR2514375	S	S	S	S
ERR2512675	S	S	S	R
ERR2513319	S	S	S	S
ERR2513582	S	S	S	S
ERR2514101	S	S	S	S
ERR2514574	S	S	S	S
ERR2513534	S	S	S	S
ERR2513729	S	S	S	S
ERR2514910	S	S	S	S
ERR2513238	S	S	S	S
ERR2512873	S	S	S	S
ERR2513289	S	S	S	S
ERR2514269	S	S	S	S
ERR2512777	S	S	S	S
ERR2514812	S	S	S	S
ERR2513982	S	S	S	S
ERR2514955	S	S	S	R
ERR2513171	S	S	S	S
ERR2513879	S	S	S	S
ERR2512580	S	S	S	S
ERR2512870	R	S	S	S
ERR2514959	S	S	S	S
ERR2514651	S	S	S	S
ERR2514292	S	S	S	S
ERR2514990	S	S	S	S
ERR2513365	S	S	S	S
ERR2514431	S	S	S	S
ERR2512629	S	S	U	S
ERR2514805	S	S	S	S
ERR2513862	S	S	S	S
ERR2514443	S	S	S	S
ERR2514060	S	S	S	S
ERR2512571	S	S	S	S
ERR2514196	S	S	S	S
ERR2513148	S	S	S	S
ERR2512788	S	S	S	S
ERR2512974	S	S	S	S
ERR2513667	S	S	S	S
ERR2515048	S	S	S	S
ERR2514562	S	S	S	S
ERR2512859	S	S	S	S
ERR2513381	S	S	S	S
ERR2514606	S	S	S	S
ERR2514468	S	S	S	S
ERR2513922	S	S	S	S
ERR2513814	S	S	S	S
ERR2513973	S	S	S	S
ERR2512944	S	S	S	S
ERR2512739	S	S	S	S
ERR2514483	S	S	S	R
ERR2514450	S	S	S	S
ERR2513566	S	S	S	S

ERR2512419	R	R	R	R
ERR2514426	S	S	S	S
ERR2514344	S	S	S	S
ERR2514672	S	S	S	S
ERR2513300	S	S	S	S
ERR2514537	S	S	S	S
ERR2514302	S	S	S	S
ERR2512708	S	S	S	S
ERR2513521	S	S	S	S
ERR2512591	R	R	R	R
ERR2513640	S	S	S	S
ERR2514747	R	S	S	S
ERR2514133	S	S	S	S
ERR2513666	S	S	S	S
ERR2513261	S	S	S	S
ERR2513739	S	S	S	S
ERR2513847	S	S	S	S
ERR2513091	S	S	S	S
ERR2513037	S	S	S	S
ERR2515088	S	S	S	S
ERR2515097	S	S	S	S
ERR2512584	S	S	S	S
ERR2514653	S	S	S	S
ERR2513306	S	S	S	S
ERR2513090	S	S	S	S
ERR2514601	S	S	S	S
ERR2514602	S	S	S	S
ERR2513635	S	S	S	S
ERR2513983	S	S	S	S
ERR2515047	S	S	S	S
ERR2512824	R	S	S	S
ERR2512898	S	S	S	S
ERR2514010	S	S	S	S
ERR2513075	S	S	S	S
ERR2513116	S	S	S	S
ERR2513793	S	S	S	S
ERR2514530	S	S	S	S
ERR2514683	S	S	S	S
ERR2513184	S	S	S	S
ERR2513636	S	S	S	S
ERR2512734	S	S	S	S
ERR2513638	S	S	S	S
ERR2514232	S	S	S	S
ERR2512860	S	S	S	S
ERR2514691	S	S	S	S
ERR2512627	S	S	S	S
ERR2514676	S	S	S	S
ERR2513176	S	S	S	S
ERR2514428	S	S	S	S
ERR2512802	R	S	S	S
ERR2513000	R	S	R	S
ERR2513167	S	S	S	S
ERR2514604	S	S	S	S

ERR2514761	S	S	S	S
ERR2512719	S	S	S	S
ERR2515065	S	S	S	S
ERR2514511	S	S	S	S
ERR2513643	S	S	S	S
ERR2512554	S	S	S	S
ERR2514819	S	S	S	S
ERR2514497	S	S	S	S
ERR2514944	S	S	S	S
ERR2513250	S	S	S	S
ERR2512761	S	S	S	S
ERR2513707	S	S	S	S
ERR2513774	S	S	S	S
ERR2514211	S	S	S	S
ERR2513303	S	S	S	S
ERR2513389	S	S	S	S
ERR2513170	S	S	S	S
ERR2512512	S	S	S	S
ERR2513681	S	S	S	S
ERR2512853	S	S	S	S
ERR2513647	S	S	S	S
ERR2514764	R	S	S	S
ERR2512884	S	S	S	S
ERR2512445	S	S	S	S
ERR2513981	S	S	S	S
ERR2512766	S	S	S	S
ERR2512791	S	S	S	S
ERR2512749	S	S	S	S
ERR2514630	S	S	S	S
ERR2513529	S	S	S	S
ERR2513815	S	S	S	S
ERR2514404	S	S	S	S
ERR2513292	S	S	S	S
ERR2514834	S	S	S	S
ERR2514853	S	S	S	S
ERR2513378	S	S	S	S
ERR2513690	S	S	S	S
ERR2513038	S	S	S	S
ERR2513819	R	R	S	S
ERR2513712	S	S	S	S
ERR2514627	S	S	S	S
ERR2513076	S	S	S	S
ERR2513882	S	S	S	S
ERR2514439	S	S	S	S
ERR2514494	S	S	S	S
ERR2513731	S	S	S	S
ERR2512986	S	S	S	S
ERR2513454	S	S	S	S
ERR2514730	S	S	S	S
ERR2514742	S	S	S	S
ERR2512519	S	S	S	S
ERR2514556	S	S	S	S
ERR2514493	S	S	S	S

ERR2513622	S	S	S	S
ERR2514204	S	S	S	S
ERR2513596	S	S	S	S
ERR2513778	S	S	S	S
ERR2513783	R	S	S	S
ERR2514093	S	S	S	S
ERR2512914	S	S	S	S
ERR2514495	S	S	S	S
ERR2514645	S	S	S	S
ERR2513182	S	S	S	S
ERR2513984	S	S	S	S
ERR2514759	S	S	S	S
ERR2513476	S	S	S	S
ERR2513425	S	S	S	S
ERR2513379	U	U	U	S
ERR2513087	S	S	S	S
ERR2513780	S	S	S	S
ERR2514486	S	S	S	S
ERR2514135	S	S	S	S
ERR2515051	S	S	S	S
ERR2514226	S	S	S	S
ERR2514798	S	S	S	S
ERR2514318	S	S	S	S
ERR2512768	S	S	S	S
ERR2513965	S	S	S	S
ERR2514561	S	S	S	S
ERR2514471	S	S	S	S
ERR2513027	R	R	S	S
ERR2513104	R	R	S	U
ERR2514121	S	S	S	S
ERR2514421	S	S	S	S
ERR2512924	S	S	S	S
ERR2513939	S	S	S	S
ERR2512538	S	S	S	S
ERR2513598	S	S	S	S
ERR2512489	S	S	S	S
ERR2513526	S	S	S	S
ERR2514154	S	S	S	S
ERR2514816	S	S	S	S
ERR2512738	S	S	S	S
ERR2513957	R	U	S	S
ERR2514675	S	S	S	S
ERR2513702	S	S	S	S
ERR2513051	S	S	S	S
ERR2514385	S	S	S	S
ERR2512752	R	S	S	S
ERR2514979	U	U	U	R
ERR2514056	S	S	S	S
ERR2512545	S	S	S	S
ERR2512543	S	S	S	S
ERR2513829	R	S	S	S
ERR2513216	S	S	S	S
ERR2512779	S	S	S	S

ERR2513316	S	S	S	S
ERR2513832	S	S	S	S
ERR2514222	S	S	S	S
ERR2512718	R	R	S	R
ERR2512704	S	S	S	S
ERR2513835	R	R	R	R
ERR2513711	S	S	S	S
ERR2512517	S	S	S	S
ERR2513757	S	S	S	S
ERR2513143	S	S	S	S
ERR2513574	S	S	S	S
ERR2513607	U	S	S	R
ERR2513609	S	S	S	S
ERR2513195	S	S	S	S
ERR2514624	S	S	S	S
ERR2513946	S	S	S	S
ERR2513401	R	S	S	S
ERR2514283	S	S	S	S
ERR2512815	S	S	S	S
ERR2514539	S	S	S	S
ERR2513481	S	S	S	S
ERR2512832	S	S	S	S
ERR2515011	S	S	S	S
ERR2514213	S	S	S	S
ERR2514372	S	S	S	S
ERR2514709	S	S	S	S
ERR2514522	U	U	U	S
ERR2512965	S	S	S	S
ERR2512993	R	S	S	S
ERR2514609	R	S	S	S
ERR2514194	S	S	S	S
ERR2513569	S	S	S	S
ERR2512418	S	S	S	S
ERR2513853	S	S	S	S
ERR2512808	U	U	U	S
ERR2513625	U	S	S	S
ERR2513163	S	S	S	U
ERR2514330	S	S	S	U
ERR2512477	S	S	S	S
ERR2513826	U	U	U	S
ERR2514613	S	S	S	S
ERR2513594	S	S	S	S
ERR2515021	S	S	S	S
ERR2514553	S	S	S	S
ERR2514700	S	S	S	S
ERR2512535	S	S	S	S
ERR2513458	S	S	S	S
ERR2514171	S	S	S	S
ERR2515064	U	U	U	S
ERR2515034	S	S	S	S
ERR2514508	S	S	S	S
ERR2513013	S	S	S	S
ERR2512726	S	S	S	S

ERR2513007	S	S	S	S
ERR2514867	R	R	S	U
ERR2513517	U	U	U	S
ERR2513394	S	S	S	S
ERR2513547	S	S	S	S
ERR2512983	S	S	S	S
ERR2514278	S	S	S	S
ERR2513392	S	S	S	S
ERR2514608	S	S	S	S
ERR2513701	S	S	S	S
ERR2513770	R	R	R	U
ERR2512500	U	U	U	S
ERR2513194	S	S	S	R
ERR2513886	S	S	S	S
ERR2514039	S	S	S	S
ERR2514368	S	S	S	S
ERR2512679	S	S	S	S
ERR2513646	S	S	S	S
ERR2512789	S	S	S	S
ERR2514182	S	S	S	S
ERR2513572	U	U	U	S
ERR2513042	S	S	S	S
ERR2514457	S	S	S	S
ERR2514037	S	S	S	S
ERR2513307	S	S	S	S
ERR2514753	S	S	S	S
ERR2514102	S	S	S	S
ERR2514151	S	S	S	S
ERR2514117	S	S	S	S
ERR2514838	S	S	S	S
ERR137225	R	R	S	R
ERR137227	R	S	S	S
ERR137228	R	S	S	S
ERR137229	R	R	R	R
ERR2199829	S	S	S	S
ERR137230	R	R	R	S
ERR551664	S	S	S	S
ERR552063	S	S	S	S
ERR137231	R	R	R	R
ERR551008	S	S	S	S
ERR108513	S	S	S	S
ERR137232	S	S	S	S
ERR137233	R	S	R	S
ERR067700	R	R	R	U
ERR550769	S	S	S	S
ERR137234	R	R	S	R
ERR234651	R	S	R	R
ERR552773	S	S	S	S
ERR137235	S	S	S	S
ERR067703	S	S	S	S
ERR067704	R	R	R	S
ERR551335	S	S	S	S
ERR067705	R	R	S	R

ERR551876	S	S	S	S
ERR067706	S	S	S	S
ERR550790	S	S	S	S
ERR067707	R	R	R	S
ERR2510189	R	R	U	U
ERR2510169	S	R	U	U
ERR2510204	R	S	U	U
ERR2510200	R	S	U	U
ERR2510173	R	S	U	U
ERR2510206	S	S	U	U
ERR2510143	S	S	U	U
ERR2510220	S	S	U	U
ERR2510138	R	R	U	U
ERR2510177	R	S	U	U
ERR2510237	R	S	U	U
ERR2510233	S	R	U	U
ERR2510211	R	R	U	U
ERR2510216	R	S	U	U
ERR2510183	S	S	U	U
ERR2510186	S	S	U	U
ERR2510242	R	R	U	U
ERR2510182	S	R	U	U
ERR2510203	R	S	U	U
ERR2510230	R	S	U	U
ERR2510157	S	S	U	U
ERR2510240	R	S	U	U
ERR2510239	S	S	U	U
ERR2510166	R	S	U	U
ERR2510212	S	R	U	U
ERR2510129	R	R	U	U
ERR2510163	R	S	U	U
ERR2510226	S	R	U	U
ERR2510213	R	S	U	U
ERR2510185	R	R	U	U
ERR2510210	R	S	U	U
ERR2510179	R	S	U	U
ERR2510191	R	R	U	U
ERR2510175	R	R	U	U
ERR2510162	R	S	U	U
ERR2510228	S	R	U	U
ERR2510209	R	S	U	U
ERR2510181	R	R	U	U
ERR067708	S	S	S	S
ERR067709	R	R	R	R
ERR067711	R	R	R	S
ERR551270	S	S	S	S
ERR551958	S	S	S	S
ERR067713	S	S	S	S
ERR2510174	R	R	U	U
ERR2510142	R	S	U	U
ERR2510199	R	R	U	U
ERR2510155	R	S	U	U
ERR2510227	R	S	U	U

ERR2510229	S	S	U	U
ERR2510147	R	S	U	U
ERR2510193	R	S	U	U
ERR2510235	R	R	U	U
ERR2510149	R	S	U	U
ERR2510130	R	S	U	U
ERR2510139	R	S	U	U
ERR2510132	R	R	U	U
ERR2510156	R	S	U	U
ERR2510223	R	S	U	U
ERR2510218	R	R	U	U
ERR2510133	R	R	U	U
ERR2510192	R	S	U	U
ERR2510215	R	R	U	U
ERR2510158	S	S	U	U
ERR2510146	R	R	U	U
ERR2510188	R	R	U	U
ERR2510224	R	R	U	U
ERR2510134	R	S	U	U
ERR2510194	R	R	U	U
ERR2510184	S	R	U	U
ERR2510180	R	S	U	U
ERR2510205	R	S	U	U
ERR2510198	R	S	U	U
ERR2510154	R	R	U	U
ERR2510131	R	S	U	U
ERR2510167	R	R	U	U
ERR2510214	S	R	U	U
ERR2510164	R	R	U	U
ERR2510150	S	R	U	U
ERR2510144	R	S	U	U
ERR2510168	R	R	U	U
ERR2510172	S	R	U	U
ERR2510241	R	S	U	U
ERR2510234	R	R	U	U
ERR2510208	R	S	U	U
ERR2510136	R	R	U	U
ERR2510153	R	R	U	U
ERR2510201	S	R	U	U
ERR2510236	R	S	U	U
ERR2510161	R	S	U	U
ERR2510135	R	R	U	U
ERR2510141	S	R	U	U
ERR2510238	R	R	U	U
ERR2510145	S	R	U	U
ERR2510195	S	R	U	U
ERR2510128	S	R	U	U
ERR2510151	S	S	U	U
ERR2510170	R	R	U	U
ERR2510222	R	S	U	U
ERR2510231	S	R	U	U
ERR2510207	R	S	U	U
ERR2510190	R	R	U	U

ERR2510159 R R U U
ERR2510225 R R U U
ERR2510176 S S U U
ERR2510217 R R U U
ERR2510196 R R U U
ERR2510219 R R U U
ERR2510178 S S U U
ERR2510140 R R U U
ERR2510187 R R U U
ERR2510160 R R U U
ERR2510221 R R U U
ERR2510152 R S U U
ERR2510232 R R U U
ERR2510165 R R U U
ERR2510148 R R U U
ERR227982 R R R S
ERR2516196 R R R R
ERR2516243 R R S R
ERR2516188 R R R R
ERR2516199 R R R R
ERR2516396 R R R R
ERR2516220 R R S R
ERR2516397 R R S R
ERR2516214 R R S R
PRJNA413593 S S S U
PRJNA413593 S S S U
PRJNA413593 S S S U
PRJNA413593 S S S U
PRJNA413593 S S S U
PRJNA413593 S S S U
PRJNA413593 S S S U
PRJNA413593 R S S S
PRJNA413593 R S S S
PRJNA413593 S S S U
PRJNA413593 S S S U
PRJNA413593 S S S U
PRJNA413593 S S S U
PRJNA413593 S S S U
PRJNA413593 S S S U
PRJNA413593 S S S U
PRJNA413593 S S S U
PRJNA413593 S S S U
PRJNA413593 S S S U
PRJNA413593 S S S U
PRJNA413593 S S S U
PRJNA413593 R S S S
PRJNA413593 S S S U
PRJNA413593 S S S U
PRJNA413593 S S S U
PRJNA413593 S S S U
PRJNA413593 S S S U
PRJNA413593 S S S U
PRJNA413593 R S S R

PRJNA413593	S	S	S	U
PRJNA413593	R	S	S	S
PRJNA413593	S	S	S	U
PRJNA413593	R	R	S	R
PRJNA413593	S	S	S	S
PRJNA413593	S	S	S	U
PRJNA413593	S	S	S	U
PRJNA413593	R	R	R	R
PRJNA413593	S	S	S	U
PRJNA413593	S	R	S	S
PRJNA413593	S	S	S	U
PRJNA413593	S	S	S	U
PRJNA413593	S	S	S	U
PRJNA413593	S	S	S	U
PRJNA413593	S	S	S	U
PRJNA413593	S	S	S	U
PRJNA413593	R	S	S	S
PRJNA413593	S	S	S	U
PRJNA413593	S	S	S	S
PRJNA413593	S	S	S	U
PRJNA413593	S	S	S	U
PRJNA413593	S	S	S	U
PRJNA413593	R	R	S	S
PRJNA413593	S	S	S	U
PRJNA413593	R	S	S	S
PRJNA413593	S	S	S	U
PRJNA413593	S	S	S	U
PRJNA413593	R	S	S	S
PRJNA413593	S	S	S	U
PRJNA413593	S	S	S	U
PRJNA413593	S	S	S	U
PRJNA413593	S	S	S	U
PRJNA413593	R	S	S	S
PRJNA413593	S	S	S	U
PRJNA413593	R	R	S	S
PRJNA413593	S	S	S	U
PRJNA413593	R	S	S	S
PRJNA413593	S	S	S	U
PRJNA413593	S	S	S	U
PRJNA413593	R	S	S	S
PRJNA413593	S	S	S	U
PRJNA413593	R	S	S	S
PRJNA413593	S	S	S	U
PRJNA413593	S	S	S	U
PRJNA413593	S	S	S	U
PRJNA413593	S	S	S	U
PRJNA413593	R	R	S	S
PRJNA413593	S	S	S	U
PRJNA413593	S	S	S	U
PRJNA413593	R	S	S	S
PRJNA413593	S	S	S	U
PRJNA413593	S	S	S	U
PRJNA413593	S	S	S	U

PRJNA413593	S	S	S	U
PRJNA413593	R	S	S	S
PRJNA413593	S	S	S	U
PRJNA413593	S	S	S	U
PRJNA413593	R	S	S	S
PRJNA413593	S	S	S	U
PRJNA413593	S	S	S	U
PRJNA413593	S	R	S	S
PRJNA413593	R	S	S	S
PRJNA413593	S	S	S	U
PRJNA413593	S	S	S	U
PRJNA413593	R	S	S	S
PRJNA413593	S	S	S	U
PRJNA413593	S	S	S	U
PRJNA413593	S	S	S	U
PRJNA413593	S	S	S	U
PRJNA413593	R	S	S	R
PRJNA413593	S	S	S	U
PRJNA413593	R	S	S	S
PRJNA413593	S	S	S	U
PRJNA413593	S	S	S	U
PRJNA413593	S	S	S	U
PRJNA413593	S	S	S	U
PRJNA413593	S	S	S	U
PRJNA413593	S	S	S	U
PRJNA413593	S	S	S	U
PRJNA413593	R	S	S	S
PRJNA413593	S	S	S	U
PRJNA413593	S	S	S	U
PRJNA413593	R	S	S	S
PRJNA413593	S	S	S	U
PRJNA413593	S	S	S	U
PRJNA413593	S	S	S	U
PRJNA413593	R	S	S	S
PRJNA413593	S	S	S	U
PRJNA413593	S	S	S	U
PRJNA413593	R	R	S	R
PRJNA413593	S	S	S	U
PRJNA413593	R	S	S	S
PRJNA413593	R	S	S	S
PRJNA413593	S	S	S	U
PRJNA413593	S	S	S	U
PRJNA413593	S	S	S	U
PRJNA413593	S	S	S	U
PRJNA413593	S	S	S	U
PRJNA413593	R	S	S	S
ERR2512949	S	S	S	S
ERR2513180	S	S	S	S
ERR2512606	R	S	S	S
ERR2513335	S	S	S	S
ERR2514712	S	S	S	S
ERR2514319	S	S	S	S
SAMN07659554	S	S	S	S

ERR2513495	S	S	S	S
ERR2514424	R	S	R	S
ERR2513064	S	S	S	S
SAMN07659036	R	S	S	S
ERR2514393	S	S	S	S
ERR2514509	S	S	S	S
ERR2513905	S	S	S	S
ERR2513169	S	S	S	S
ERR2513976	S	S	S	S
ERR2515035	S	S	U	U
ERR2512875	S	S	S	S
ERR2514348	S	S	S	S
ERR2514340	R	S	S	S
ERR2514657	S	S	S	S
ERR2513408	R	R	R	R
ERR2513488	R	S	S	S
ERR2514231	S	S	S	S
ERR2514053	R	S	S	S
ERR2512490	S	S	S	S
ERR2514685	S	S	S	S
ERR2514413	S	S	S	S
ERR2513459	S	S	S	S
ERR2513423	S	S	S	S
ERR2514112	S	S	S	S
ERR2512821	R	S	S	S
SAMN07658450	S	S	S	S
ERR2512700	R	R	R	R
ERR2512457	S	S	S	S
ERR2512441	S	S	S	S
SAMN07658683	S	S	S	S
ERR2512566	S	S	S	S
ERR2513349	S	S	S	S
ERR2512849	S	S	S	S
ERR2514174	S	S	S	S
ERR2514533	S	S	S	S
ERR2513713	S	S	S	S
ERR2512550	S	S	S	S
ERR2513311	S	S	S	S
ERR2513789	R	S	S	S
ERR2514902	S	S	S	S
ERR2514947	R	S	S	S
ERR2514097	S	S	S	S
ERR2514829	S	S	S	S
ERR2512890	S	S	S	S
ERR2513852	R	R	R	S
ERR2513082	S	S	S	S
ERR2512578	S	S	S	S
ERR2512465	S	S	S	S
ERR2514766	S	S	S	S
ERR2513873	S	S	S	S
ERR2512828	S	S	S	S
ERR2514181	S	S	S	S
SAMN07658160	S	S	S	S

SAMN07659677 S S S S
ERR2513951 S S S S
ERR2513084 S S S S
ERR2514855 S S S S
ERR2513942 S S S S
ERR2514756 S S S S
ERR2513734 S S S S
ERR2513910 S S S S
ERR2514233 S S S S
SAMN07659475 S S S S
ERR2513932 S S S S
ERR2512754 S S S S
SAMN07658236 S S S S
ERR2513059 S S S S
ERR2512866 S S S S
ERR2513508 S S S S
ERR2513699 S S S S
ERR2514043 S S S S
ERR2514173 S S S S
SAMN07660272 S S S S
ERR2513360 S S S S
ERR2513618 S S S S
ERR2513710 R S S S
ERR2513298 S S S S
ERR2513324 S S S S
ERR2514741 S S S S
ERR2514873 S S S S
ERR2515016 S S S R
ERR2514170 S S S S
ERR2513490 S S S S
SAMN07658172 S S S S
ERR2512714 S S S S
SAMN07658780 S S S S
ERR2513313 S S S S
ERR2514317 S S S S
ERR2513936 S S S S
ERR2512818 S S S S
ERR2512532 S S S S
ERR2514205 S S S S
ERR2513065 S S S S
ERR2512958 S S S S
ERR2514668 S S S S
ERR2514086 S S S S
ERR2513245 S S S S
ERR2513108 S S S S
ERR2513877 S S S S
ERR2514877 U U U S
ERR2514929 R S S S
ERR2513581 S S S S
ERR2513152 S S S S
ERR2513568 S S S S
ERR2513523 S S S S
ERR2512613 S S S S

ERR2513426	S	S	S	S
SAMN07660336	S	S	S	S
ERR2512862	S	S	S	S
ERR2514411	S	S	S	S
ERR2512674	S	S	S	S
SAMN07658831	S	S	S	S
ERR2512495	S	S	S	R
ERR2514652	S	S	S	S
ERR2513728	S	S	S	S
ERR2512594	S	S	S	S
SAMN07658534	S	S	U	S
ERR2514516	S	S	S	S
ERR2513907	S	S	S	S
ERR2512631	S	S	S	S
ERR2514111	S	S	S	S
ERR2514920	S	S	S	S
SAMN07659521	S	S	S	S
ERR2513451	S	S	S	S
ERR2514298	S	S	S	S
ERR2513600	S	S	S	S
ERR2513653	S	S	S	S
ERR2514091	S	S	S	S
ERR2514425	S	S	S	S
ERR2513450	S	S	S	S
ERR2513243	S	S	S	S
ERR2513428	S	S	S	R
ERR2514061	S	S	S	S
ERR2512449	S	S	S	S
ERR2513808	S	S	S	S
ERR2514711	S	S	S	S
ERR2514635	S	S	S	S
ERR2514024	S	S	S	S
SAMN07660374	S	S	S	S
ERR2513502	S	S	S	S
SAMN07658862	R	S	S	S
ERR2512838	S	S	S	R
ERR2514817	S	S	S	S
ERR2512599	S	S	S	S
ERR2514661	S	S	S	S
ERR2513496	S	S	S	S
ERR2514576	S	S	S	S
SAMN07658511	S	S	S	S
ERR2513200	S	S	S	S
ERR2515078	S	S	S	S
SAMN07659532	S	S	S	S
ERR2514724	S	S	S	S
ERR2512544	R	S	S	S
ERR2514477	S	S	S	S
ERR2514565	S	S	S	S
ERR2514474	S	S	S	S
ERR2513056	R	S	R	S
ERR2512661	S	S	S	S
ERR2513868	S	S	S	S

ERR2512867	S	S	S	S
ERR2513242	S	S	S	S
ERR2513520	S	S	S	S
ERR2513462	S	S	S	S
SAMN07658301	S	S	S	S
ERR2513630	S	S	S	S
ERR2512757	S	S	S	S
ERR2513975	S	S	S	S
ERR2513032	S	S	S	S
ERR2513254	S	S	S	S
ERR2514241	S	S	S	S
SAMN07658467	S	S	S	S
ERR2514619	S	S	S	S
ERR2514013	S	S	S	S
SAMN07659844	S	S	S	S
SAMN07658342	S	S	S	S
SAMN07659715	S	S	S	S
SAMN07660146	S	S	S	S
SAMN07658363	S	S	S	S
SAMN07658667	S	S	S	S
SAMN07659803	S	S	S	S
SAMN07660208	S	S	S	S
ERR2514327	R	S	S	S
SAMN07658969	S	S	S	S
SAMN07659815	S	S	S	S
SAMN07658845	S	S	S	S
SAMN07660234	S	S	S	S
SAMN07659809	S	S	S	S
SAMN07658645	S	S	S	S
SAMN07658153	S	S	S	S
SAMN07659620	S	S	S	R
SAMN07660455	S	S	S	S
SAMN07658821	S	S	S	S
SAMN07659680	S	S	S	S
SAMN07659362	S	S	S	S
SAMN07658690	S	S	S	S
SAMN07658586	S	S	S	S
SAMN07659116	S	S	S	S
SAMN07659995	S	S	S	S
SAMN07658936	S	S	S	U
SAMN07659943	R	R	R	U
SAMN07659075	S	S	S	S
SAMN07658733	S	S	S	S
SAMN07658539	S	S	S	S
SAMN07660461	S	S	S	S
SAMN07660147	S	S	S	S
SAMN07660426	S	S	S	S
SAMN07659676	S	S	S	S
SAMN07660082	S	S	S	S
SAMN07660017	S	S	S	S
SAMN07660206	S	S	S	S
SAMN07659105	S	S	S	S
SAMN07658809	S	S	S	S

SAMN07658424	R	S	S	S
SAMN07658558	S	S	S	S
SAMN07658568	S	S	S	S
SAMN07658510	R	R	S	S
SAMN07659964	S	S	S	S
SAMN07659610	S	S	S	S
SAMN07658775	S	S	S	S
SAMN07659838	S	S	S	S
SAMN07660454	S	S	S	S
SAMN07659329	S	S	S	S
SAMN07658958	S	S	S	S
SAMN07658912	S	S	S	S
SAMN07659808	R	S	S	S
SAMN07658489	S	S	S	S
SAMN07659434	S	S	S	S
SAMN07660190	S	S	S	S
SAMN07658795	S	S	S	S
SAMN07658349	S	S	S	S
SAMN07660178	S	S	S	S
SAMN07658404	S	S	S	U
SAMN07659642	S	S	S	S
SAMN07660069	S	S	S	S
SAMN07660435	S	S	S	S
SAMN07658614	S	S	S	S
SAMN07658208	R	S	S	S
SAMN07659734	S	S	S	S
SAMN07660305	R	R	S	S
SAMN07660157	U	S	S	S
SAMN07658158	S	S	S	S
SAMN07659695	R	S	S	S
SAMN07658848	R	S	S	S
SAMN07658253	S	S	S	S
SAMN07658804	S	S	S	U
SAMN07659787	U	U	U	S
SAMN07659443	S	S	S	S
SAMN07658688	S	S	S	S
SAMN07658901	S	S	S	S
SAMN07660270	S	S	S	S
SAMN07660123	S	S	S	S
ERR2514943	S	S	S	S
SAMN07658471	S	S	S	S
ERR2513812	S	S	S	S
ERR2515059	S	S	S	S
ERR2514925	S	S	S	S
ERR2514288	R	S	R	S
ERR2514275	U	U	U	S
ERR2513836	S	S	S	S
ERR2513682	S	S	S	S
ERR2514191	S	S	S	R
ERR2514454	S	S	S	S
ERR2514381	R	S	S	S
ERR2514063	R	S	S	U
ERR2513364	S	S	S	S

SAMN07660286	S	S	S	S
ERR2513005	S	S	S	S
SAMN07660300	R	R	S	S
ERR2513631	R	R	R	U
ERR2514550	R	R	S	S
ERR2514107	S	S	S	S
ERR2513603	S	S	S	S
SAMN07660217	S	S	S	S
ERR2512931	S	S	S	R
ERR2514105	S	S	S	S
ERR2513419	S	S	S	S
ERR2513138	S	S	S	R
ERR2513779	S	S	S	S
ERR2513923	S	S	S	S
ERR2512504	S	S	S	S
ERR2514961	S	S	S	S
ERR2514896	S	S	S	S
SAMN07659506	S	S	S	S
SAMN07660216	S	S	S	S
ERR2514364	S	S	S	S
SAMN07660425	S	S	S	S
SAMN07659632	S	S	S	S
SAMN07660463	S	S	S	S
ERR2514690	S	S	S	S
SAMN07660210	S	S	S	S
SAMN07658566	S	S	S	S
SAMN07659451	S	S	S	S
SAMN07659990	S	S	S	S
SAMN07659690	S	S	S	S
SAMN07659761	S	S	S	S
SAMN07660044	S	S	S	S
SAMN07658355	S	S	S	S
SAMN07658237	S	S	S	S
SAMN07659798	R	U	S	S
SAMN07660115	R	U	U	R
SAMN07659972	S	S	S	S
SAMN07659436	R	U	S	S
SAMN07660153	S	S	S	S
SAMN07659952	S	S	S	S
SAMN07658849	S	S	S	S
SAMN07660385	R	R	S	U
SAMN07660055	S	S	S	S
SAMN07658695	S	S	S	S
SAMN07660096	S	S	S	S
SAMN07659458	S	S	S	S
SAMN07659392	S	S	S	S
SAMN07658304	S	S	S	S
SAMN07658397	S	S	S	S
SAMN07659891	S	S	S	S
SAMN07660345	R	S	S	U
SAMN07658395	S	S	S	S
SAMN07659820	S	S	S	S
SAMN07658245	S	S	S	S

SAMN07659775	S	S	S	S
ERR2513126	R	R	S	R
SAMN07658244	R	S	S	S
SAMN07660011	S	S	S	S
SAMN07658541	S	S	S	S
SAMN07658156	S	S	S	S
SAMN07659009	S	S	S	S
SAMN07658270	S	S	S	S
SAMN07658486	S	S	S	S
SAMN07658573	S	S	S	S
SAMN07659823	S	S	S	S
SAMN07660103	S	S	S	S
SAMN07658923	S	S	S	S
SAMN07658942	S	S	S	S
SAMN07660440	S	S	S	S
SAMN07660111	S	S	S	U
SAMN07658300	S	S	S	S
SAMN07659988	S	S	S	S
SAMN07658803	S	S	S	S
SAMN07658144	R	S	S	S
SAMN07660074	S	S	S	S
SAMN07659879	S	S	S	S
SAMN07660375	S	S	S	S
SAMN07660373	U	U	S	U
SAMN07658993	S	S	S	S
SAMN07659717	R	S	S	S
SAMN07660326	S	S	S	S
ERR2514069	S	S	S	S
SAMN07659826	U	S	S	S
SAMN07659430	S	S	S	S
SAMN07658148	S	S	S	S
SAMN07659706	S	S	S	S
SAMN07658720	S	S	S	S
SAMN07659412	S	S	S	S
SAMN07658167	S	S	S	S
SAMN07658735	S	S	S	S
SAMN07658872	S	S	S	U
SAMN07658368	S	S	S	S
SAMN07659885	R	R	S	R
SAMN07659502	S	S	S	S
SAMN07658155	S	S	S	S
SAMN07658547	S	S	S	S
SAMN07659756	S	S	S	S
SAMN07659387	R	S	S	S
SAMN07658320	S	S	S	S
SAMN07659772	S	S	S	S
SAMN07658878	S	S	S	S
SAMN07660263	S	S	S	S
SAMN07660316	R	S	S	S
SAMN07660358	S	S	S	S
SAMN07659793	R	S	S	S
SAMN07659824	S	S	S	S
SAMN07659100	S	S	S	S

SAMN07660466	S	S	S	S
SAMN07658585	S	S	S	S
SAMN07658618	S	S	S	S
SAMN07659937	S	S	S	S
SAMN07659851	S	S	S	S
SAMN07658723	S	S	S	S
SAMN07658464	S	S	S	S
SAMN07659575	S	S	S	S
ERR2514821	S	S	S	S
SAMN07660436	S	S	S	S
ERR2514547	S	S	S	S
SAMN07659485	S	S	S	S
SAMN07660108	S	S	S	S
SAMN07659407	S	S	S	S
SAMN07658376	S	S	S	S
SAMN07658771	S	S	S	S
SAMN07659432	S	S	S	S
SAMN07658452	S	S	S	S
SAMN07658506	S	S	S	S
SAMN07660218	S	S	S	S
SAMN07658485	S	S	S	S
SAMN07659660	S	S	S	S
SAMN07658147	S	S	S	S
SAMN07658927	S	S	S	S
SAMN07660202	S	S	S	S
SAMN07659631	S	S	S	S
SAMN07658830	S	S	S	S
SAMN07659096	S	S	S	S
SAMN07659052	S	S	S	S
SAMN07659691	S	S	S	S
SAMN07659077	S	S	S	S
SAMN07658546	S	S	S	S
SAMN07658328	S	S	S	S
SAMN07658459	S	S	S	S
SAMN07659862	S	S	S	S
ERR2514346	S	S	S	S
SAMN07658392	R	S	S	S
SAMN07658341	S	S	S	S
SAMN07660224	S	S	S	S
SAMN07660346	S	S	S	S
SAMN07658352	S	S	S	S
SAMN07658271	R	S	S	S
SAMN07660056	S	S	S	U
SAMN07658886	U	U	U	S
SAMN07658992	S	S	S	S
SAMN07658827	S	S	S	R
SAMN07660160	S	S	S	S
SAMN07659693	U	S	S	U
SAMN07659718	S	S	S	S
SAMN07659044	S	S	S	S
SAMN07658575	U	S	S	U
SAMN07658483	S	S	S	S
SAMN07658518	S	S	S	S

SAMN07659456	S	S	S	S
SAMN07660078	S	S	S	S
SAMN07660170	U	U	U	S
SAMN07660453	U	S	S	U
SAMN07658902	S	S	S	S
SAMN07659986	S	S	S	S
SAMN07660005	S	S	S	S
SAMN07658514	S	S	S	S
SAMN07660213	S	S	S	S
SAMN07658399	S	S	S	S
SAMN07660022	S	S	S	S
SAMN07659054	R	S	S	S
SAMN07659477	S	S	S	S
SAMN07659769	R	S	S	S
SAMN07660070	S	S	S	S
SAMN07660057	S	S	S	S
SAMN07658995	S	S	S	S
SAMN07658929	S	S	S	S
SAMN07659473	S	S	S	S
SAMN07658193	S	S	S	S
SAMN07658538	S	S	S	S
SAMN07660009	S	S	S	S
SAMN07659714	S	S	S	S
SAMN07659060	S	S	S	S
SAMN07659759	S	S	S	S
SAMN07658161	S	S	S	S
SAMN07658621	R	R	S	U
SAMN07659805	S	S	S	S
SAMN07659917	S	S	S	S
SAMN07659652	S	S	S	S
SAMN07659097	S	S	S	S
SAMN07658764	S	S	S	S
SAMN07658820	S	S	S	S
SAMN07658777	S	S	S	S
SAMN07658807	S	S	S	S
SAMN07659508	S	S	S	S
SAMN07658994	S	S	S	S
SAMN07658781	S	S	S	S
SAMN07659108	R	R	U	R
SAMN07658719	S	S	S	S
SAMN07658503	S	S	S	S
SAMN07659380	S	S	S	S
SAMN07658408	S	S	S	U
SAMN07659419	S	S	S	S
SAMN07659604	S	S	S	S
SAMN07660352	S	S	S	S
SAMN07659490	S	S	S	S
SAMN07658378	R	S	S	S
ERR2514757	R	R	S	S
SAMN07658249	R	S	S	S
SAMN07659513	S	S	S	S
SAMN07659454	S	S	S	S
SAMN07658195	S	S	S	S

SAMN07659382	R	S	S	S
SAMN07659933	R	S	S	S
SAMN07659771	S	R	S	S
SAMN07659969	S	S	S	U
SAMN07659414	S	S	S	S
SAMN07658604	S	S	S	S
SAMN07659540	S	S	S	U
SAMN07659388	R	R	U	U
SAMN07658715	S	S	S	S
SAMN07658641	S	S	S	S
SAMN07660229	S	S	S	S
SAMN07660470	S	S	S	S
SAMN07658229	S	S	S	S
SAMN07658353	S	S	S	S
SAMN07659115	S	S	S	S
SAMN07659525	S	S	S	S
SAMN07658591	S	S	S	S
SAMN07660378	R	R	S	S
SAMN07659555	S	S	S	S
ERR2513680	S	S	S	S
SAMN07658519	S	S	S	S
SAMN07660012	S	S	S	S
SAMN07659394	R	S	S	S
ERR2514968	S	S	S	S
SAMN07659910	S	S	S	S
SAMN07659627	S	S	S	U
SAMN07659860	S	S	S	S
SAMN07660283	S	S	S	S
SAMN07658786	S	S	S	S
SAMN07659989	S	S	S	S
SAMN07659732	S	S	S	S
SAMN07659033	S	S	S	S
SAMN07659594	S	S	S	S
SAMN07659905	S	S	S	S
SAMN07658949	S	S	S	S
SAMN07658892	S	S	S	S
ERR2514240	R	R	R	R
ERR2513954	R	R	S	S
ERR2514412	S	R	S	S
ERR2512882	R	R	R	R
ERR2513872	R	R	S	S
ERR2515040	S	R	S	S
ERR2513050	S	S	S	S
ERR2514887	R	R	R	R
ERR2514014	S	S	S	S
ERR2515042	R	R	R	R
ERR2514422	R	R	R	R
ERR2513656	R	R	R	R
ERR2513221	R	R	S	R
ERR2512467	R	R	S	R
ERR2514395	R	R	S	R
ERR2512975	R	R	R	R
ERR2513831	R	R	R	R

ERR2514633	R	R	R	R
ERR2514487	R	R	R	R
SAMN07659031	S	S	S	S
SAMN07658260	R	S	S	S
SAMN07660198	S	S	S	S
SAMN07658582	S	S	S	S
SAMN07659982	S	S	S	S
SAMN07659859	S	S	S	U
SAMN07658551	S	S	S	S
SAMN07658203	U	U	S	U
SAMN07659828	S	S	S	S
SAMN07659017	S	S	S	S
SAMN07659614	S	S	S	S
SAMN07658868	S	S	S	S
SAMN07660377	R	R	U	R
SAMN07658284	S	S	S	S
SAMN07659008	S	S	S	S
SAMN07658313	S	S	S	S
SAMN07659599	S	S	S	S
SAMN07659619	S	S	S	S
SAMN07658836	S	S	S	S
SAMN07658134	S	S	S	S
SAMN07658293	S	S	S	S
SAMN07660302	S	S	S	S
SAMN07659455	S	S	S	S
SAMN07660323	S	S	S	S
SAMN07658925	S	S	S	S
SAMN07658753	S	S	S	S
SAMN07659951	S	S	S	S
ERR2513133	S	S	S	S
SAMN07659006	R	S	S	S
SAMN07658396	S	S	S	S
SAMN07658640	S	S	S	S
SAMN07659745	S	S	S	S
SAMN07659341	S	S	S	S
SAMN07658567	S	S	S	U
SAMN07659661	S	S	S	S
SAMN07658675	S	S	S	S
SAMN07658263	S	S	S	S
SAMN07659941	S	S	S	S
SAMN07658840	S	S	S	S
SAMN07658393	S	S	S	S
SAMN07659117	R	R	R	U
SAMN07660311	S	S	S	S
SAMN07660342	S	S	S	S
SAMN07660186	S	S	S	S
SAMN07658639	S	S	S	S
SAMN07659929	S	S	S	S
ERR2512728	S	S	S	S
SAMN07658142	S	S	S	S
SAMN07659425	S	S	S	R
SAMN07659770	S	S	S	S
SAMN07659656	S	S	S	S

SAMN07659020	S	S	S	S
SAMN07659702	S	S	S	S
SAMN07659603	S	S	S	S
SAMN07659835	S	S	S	S
SAMN07660114	S	S	S	S
SAMN07658885	S	S	S	S
SAMN07659679	S	S	S	U
SAMN07658891	S	S	S	S
SAMN07660313	S	S	S	S
SAMN07659997	S	S	S	S
SAMN07660248	S	S	S	S
SAMN07660104	S	S	S	S
SAMN07659328	S	S	S	S
SAMN07658746	S	S	S	U
SAMN07659790	S	S	S	S
SAMN07659968	S	S	S	S
SAMN07660028	S	S	S	S
SAMN07660120	S	S	S	S
SAMN07659602	S	S	S	S
SAMN07658422	U	S	S	S
SAMN07659584	S	S	S	S
SAMN07660049	S	S	S	S
SAMN07658894	S	S	S	S
SAMN07658769	S	S	S	S
SAMN07659829	S	S	S	S
SAMN07659574	S	S	S	S
SAMN07658851	R	R	S	U
SAMN07658441	S	S	S	S
SAMN07660398	R	R	S	U
SAMN07658959	S	S	S	S
SAMN07658947	S	S	S	S
ERR2514667	S	S	S	S
SAMN07660478	S	S	S	S
SAMN07659353	S	S	S	S
SAMN07658344	S	S	S	S
SAMN07658478	S	S	S	S
SAMN07659114	S	S	S	S
SAMN07659383	S	S	S	S
SAMN07658372	S	S	S	S
SAMN07660020	S	S	S	S
SAMN07658985	R	S	S	S
SAMN07659055	R	S	S	S
SAMN07659670	S	S	S	S
SAMN07658672	S	S	S	S
SAMN07660167	S	S	S	S
SAMN07658906	S	S	U	S
SAMN07659069	S	S	S	U
SAMN07659509	S	S	S	S
SAMN07659650	R	R	S	S
SAMN07658329	S	S	S	S
SAMN07660137	S	S	S	S
SAMN07658571	S	S	S	S
SAMN07659468	R	R	R	U

SAMN07659981	R	R	S	U
SAMN07658941	S	S	S	S
SAMN07659590	S	S	S	U
SAMN07660359	S	S	S	S
SAMN07659956	S	R	S	S
SAMN07658523	S	S	S	S
SAMN07658637	S	S	S	S
SAMN07660226	S	S	S	S
SAMN07658797	S	S	S	S
SAMN07658655	S	S	S	S
SAMN07659557	S	S	S	S
SAMN07660158	S	S	S	S
SAMN07660116	S	S	S	S
SAMN07660441	R	R	R	U
SAMN07659912	S	S	S	S
SAMN07659686	S	S	S	S
SAMN07659595	S	S	S	S
SAMN07658197	S	S	S	S
SAMN07658340	S	S	S	S
SAMN07658135	S	R	S	S
SAMN07658133	R	U	S	S
SAMN07660101	S	S	S	S
SAMN07658867	S	S	S	S
SAMN07658765	S	S	S	S
SAMN07658250	S	S	S	S
SAMN07659016	S	S	S	S
SAMN07658516	S	S	S	S
SAMN07660002	S	S	S	S
SAMN07660067	S	S	S	S
SAMN07659562	S	S	S	S
SAMN07659048	S	S	S	S
SAMN07658168	S	S	S	S
SAMN07658613	S	S	S	S
SAMN07659064	S	S	S	S
SAMN07658281	S	S	S	S
ERR2514175	S	S	S	S
SAMN07658873	S	S	S	S
SAMN07660014	S	S	S	S
SAMN07658658	S	S	S	S
SAMN07658211	S	S	S	S
SAMN07660155	S	S	S	S
SAMN07659378	S	S	S	S
SAMN07659858	U	U	U	S
SAMN07659834	S	S	S	S
SAMN07658457	R	S	S	S
SAMN07658243	S	S	S	S
SAMN07659025	S	S	S	S
SAMN07659371	R	S	S	S
SAMN07660077	S	S	S	S
SAMN07658632	S	S	S	S
SAMN07659027	S	S	S	S
SAMN07659109	S	S	S	S
SAMN07658574	U	U	U	S

SAMN07659367 S S S S
SAMN07658759 S S S S
SAMN07658692 R R U S
SAMN07659326 S S S S
SAMN07658779 S S S S
SAMN07660086 S S S S
SAMN07658668 S S S U
SAMN07658437 S S S S
SAMN07658595 R S S S
SAMN07658875 S S S S
SAMN07660131 S S S S
SAMN07658487 S S S U
SAMN07659119 S S S S
SAMN07660000 S S S S
SAMN07658863 S S S S
SAMN07658282 S S S S
SAMN07658544 S S S S
SAMN07660043 S S S S
SAMN07660026 S S S S
ERR2513470 S S S S
SAMN07658321 S S S S
SAMN07658905 S S S S
SAMN07659874 S S S S
SAMN07658317 S S S S
SAMN07659654 S S S S
ERR2513806 S S S S
SAMN07658987 S S S S
SAMN07658762 S S S S
SAMN07658218 S S S U
SAMN07659882 S S S S
SAMN07659057 S S S S
SAMN07658679 S S S S
SAMN07660227 S S S U
SAMN07660221 S S S S
SAMN07660097 S S S S
SAMN07658858 S S S S
SAMN07658223 S S S S
SAMN07659965 S S S S
SAMN07660465 S S S S
SAMN07658877 S S S S
SAMN07658859 S S S S
SAMN07659342 S S S S
SAMN07660370 S S S S
SAMN07658384 S S S S
SAMN07658988 S S S S
SAMN07659059 S S S S
ERR2513589 S S S S
SAMN07660141 R S S S
ERR2515060 S S S S
SAMN07659675 S S S S
SAMN07660172 S S S S
ERR2513342 S S S S
SAMN07660397 S S S S

SAMN07660085 S S S S
SAMN07659517 S S S S
SAMN07659618 S S S S
ERR2513966 S S S S
SAMN07658185 S S S S
ERR2514529 S S S S
SAMN07658978 S S S S
SAMN07660236 S S S U
SAMN07659681 S S S S
ERR2513724 S S S S
SAMN07658816 S S S S
SAMN07659384 R S S S
ERR2513578 S S S S
SAMN07658322 S S S S
SAMN07660255 S S S S
SAMN07658200 S S S S
SAMN07658616 S S S S
SAMN07660314 S S S S
SAMN07658420 S S S S
SAMN07658512 S S S S
SAMN07659942 S S S S
SAMN07659930 S S S S
SAMN07658130 R S S S
SAMN07659089 S S S S
SAMN07658183 S S S S
SAMN07659344 S S S S
SAMN07660040 S S S S
SAMN07659526 S S S S
SAMN07658935 S S S S
SAMN07660451 S S S S
SAMN07660266 S S S S
SAMN07658962 S S S S
SAMN07660419 S S S S
SAMN07658521 S S S S
SAMN07660031 S S S S
SAMN07658684 S S S S
SAMN07660296 R S S U
ERR2513871 S S S S
SAMN07658357 S S S U
SAMN07659960 R R R R
SAMN07659337 S S S S
SAMN07659081 S S S S
SAMN07660371 S S S S
SAMN07659621 S S S S
SAMN07658508 S S S S
SAMN07660289 S S S S
ERR2514749 S S S S
SAMN07659499 S S S S
SAMN07659722 S S S S
SAMN07658682 S S S S
SAMN07658846 R R R U
ERR553081 ERR553082 S S S S
ERR137238 S S S S

ERR137239 S S S S
ERR117462 R R S S
ERR227990 R S S S
ERR117463 R R S S
ERR551693 ERR551694 S S S S
ERR227998 S S S S
ERR137240 R S R S
ERR137241 S S S S
ERR552092 S S S S
ERR137242 R S S S
ERR550797 S S S S
ERR137243 R R R R
ERR117464 R R R R
ERR108449 S R R U
ERR2510137 S R U U
ERR2510171 R R U U
ERR2510202 R R U U
ERR2510197 S R U U
ERR2199830 S S S S
ERR108450 R R R R
ERR228006 R R S R
ERR228014 R S S S
ERR551271 S S S S
ERR108451 S S S S
ERR2199831 S S S S
ERR108452 S S S S
ERR117465 R R S S
ERR137244 R S S S
ERR137245 S S S S
ERR137246 R R R R
ERR553336 S S S S
ERR117466 R S S S
ERR2199832 S S S S
ERR067714 R R R R
ERR067715 R R R S
ERR552975 S S S S
ERR117467 S S S S
ERR551859 S S S S
ERR551394 S S S S
ERR551814 S S S S
ERR137247 R R R R
ERR137248 R R S R
ERR137249 S S S S
ERR108432 S S S S
ERR108433 S S S S
ERR108434 S S S S
ERR108435 R R S R
ERR108436 R R R S
ERS458062 S S S S
ERS456778 R R R R
ERR2516192 R R S S
ERR2516389 R R S S
SAMN07658256 S S S S

SAMN07658458	S	S	S	S
SAMN07658926	S	S	S	S
SAMN07660450	S	S	S	S
SAMN07658879	S	S	S	S
ERR2515063	S	S	S	S
SAMN07658856	S	S	S	S
SAMN07658656	S	S	S	S
ERR2514150	S	S	S	S
SAMN07658646	S	S	S	S
SAMN07660262	S	S	S	S
SAMN07659967	S	S	S	S
ERR2514567	S	S	S	S
ERR2513434	S	S	S	S
SAMN07660318	S	S	S	S
SAMN07660063	S	S	S	S
SAMN07660156	S	S	S	S
SAMN07658466	S	S	S	S
SAMN07660228	S	S	S	S
SAMN07659991	S	S	S	S
SAMN07659576	S	S	S	S
SAMN07660068	S	S	S	U
SAMN07658297	S	S	S	S
SAMN07658131	S	S	S	S
SAMN07658623	R	S	S	S
SAMN07658557	S	S	S	S
SAMN07659066	S	S	S	S
SAMN07658283	U	S	S	U
SAMN07659781	S	S	S	S
ERR2514326	S	S	S	S
SAMN07660462	S	S	S	S
SAMN07659596	S	S	S	U
SAMN07658239	S	S	S	S
SAMN07658964	S	S	S	S
SAMN07658290	R	S	S	S
SAMN07660386	R	S	S	S
SAMN07658453	S	S	S	S
SAMN07659343	U	U	U	S
SAMN07658527	S	S	S	S
SAMN07660188	S	S	S	S
SAMN07658916	R	U	R	R
SAMN07659449	S	S	S	S
SAMN07659410	S	S	S	S
SAMN07658176	S	S	S	S
SAMN07658140	S	S	S	S
SAMN07660303	S	S	S	S
SAMN07658402	S	S	S	S
SAMN07658310	S	S	S	S
SAMN07660165	S	S	S	S
SAMN07659007	S	S	S	S
SAMN07658562	S	S	S	S
SAMN07660464	S	S	S	S
SAMN07659832	S	S	S	S
SAMN07658951	S	S	S	S

SAMN07659042	S	S	S	S
SAMN07658389	S	S	S	S
SAMN07658898	R	S	S	S
SAMN07658414	S	S	S	S
SAMN07658505	R	S	S	S
SAMN07658635	S	S	S	S
SAMN07658127	S	S	S	S
SAMN07659437	S	S	S	S
SAMN07658257	S	S	S	S
ERR2512929	S	S	S	S
SAMN07658278	R	S	S	S
SAMN07660073	S	S	S	U
SAMN07659689	S	S	S	S
ERR2514974	R	S	S	S
SAMN07659427	S	S	S	S
SAMN07659385	S	S	S	S
SAMN07658744	R	S	S	S
SAMN07659376	S	S	S	S
SAMN07658219	S	S	S	S
SAMN07658184	S	S	S	S
SAMN07659524	S	S	S	S
ERR2515071	S	S	S	S
SAMN07658570	R	S	S	S
SAMN07658388	S	S	S	S
ERR2512748	S	S	S	S
SAMN07659351	S	S	S	S
SAMN07659112	S	S	S	S
SAMN07658580	S	S	S	S
ERR2513533	S	S	S	S
ERR2513843	S	S	S	S
SAMN07659735	S	S	S	U
SAMN07658713	S	S	S	R
SAMN07658238	S	S	S	S
SAMN07658738	S	S	S	S
SAMN07658592	S	S	S	S
SAMN07659418	S	S	S	U
SAMN07659366	S	S	S	S
SAMN07659415	S	S	S	S
ERR2514342	S	S	S	S
SAMN07660307	S	S	S	S
SAMN07659519	R	S	S	S
SAMN07659673	S	S	S	S
SAMN07659966	S	S	S	S
SAMN07659045	S	S	S	S
SAMN07659999	S	S	S	S
SAMN07658681	S	S	S	S
SAMN07658714	R	S	S	S
SAMN07660133	S	S	S	R
SAMN07660223	S	S	S	S
SAMN07658446	R	S	U	S
SAMN07658625	S	S	S	S
SAMN07660080	S	S	S	S
SAMN07659708	S	S	S	S

SAMN07658983	U	U	U	S
SAMN07658706	S	S	S	S
SAMN07659904	R	R	S	S
SAMN07658864	S	S	S	S
ERR2513716	S	S	S	S
ERR2514118	S	S	S	S
SAMN07660015	S	S	S	S
SAMN07658374	S	S	S	S
SAMN07658755	R	S	S	S
SAMN07659939	S	S	S	S
SAMN07658178	S	S	S	S
SAMN07660100	S	S	S	S
SAMN07660112	S	S	S	S
SAMN07658461	S	S	S	S
SAMN07658530	S	S	S	S
SAMN07658922	S	S	S	S
SAMN07659709	S	S	S	S
SAMN07658347	S	S	S	S
ERR2514745	S	S	S	S
SAMN07659634	S	S	S	S
ERR2514087	S	S	S	R
ERR2513193	S	S	S	S
ERR2513371	S	S	S	S
ERR2513820	S	S	S	U
ERR2512750	S	S	S	U
ERR2514076	S	S	S	S
ERR2514451	R	S	R	R
ERR2512780	R	S	S	S
ERR2514012	S	S	S	S
ERR2512653	S	S	S	S
ERR2513683	S	S	S	S
ERR2512682	S	S	S	S
ERR2514913	S	S	S	S
ERR2512775	S	S	S	S
ERR2514369	S	S	S	S
ERR2514400	S	S	S	S
ERR2513516	S	S	S	S
ERR2512433	S	S	S	S
ERR2514848	R	S	S	S
ERR2512497	S	S	S	S
ERR2513688	S	S	S	S
ERR2513571	S	S	S	S
ERR2514728	S	S	S	S
ERR2514290	S	S	S	S
ERR2514548	S	S	S	U
ERR2513597	S	S	S	S
ERR2514811	S	S	S	S
ERR2514481	S	S	S	S
ERR2512525	S	S	S	U
ERR2513645	S	S	S	S
ERR2514643	S	S	S	S
ERR2514259	S	S	S	S
ERR2513970	S	S	S	S

ERR2513001	S	S	S	S
ERR2514078	S	S	S	S
ERR2513398	S	S	S	S
ERR2513106	S	S	S	S
ERR2512990	S	S	S	S
ERR2513172	S	S	S	S
ERR2514797	S	S	S	S
ERR2513796	S	S	S	S
ERR2515055	S	S	S	S
ERR2514377	S	S	S	S
ERR2514869	S	S	S	S
ERR2512636	S	S	S	S
ERR2512837	S	S	S	S
ERR2513742	R	R	R	U
ERR2513723	S	S	S	S
ERR2514088	R	S	R	S
ERR2513991	S	S	S	S
ERR2514528	S	S	S	S
ERR2514559	U	S	S	U
ERR2513297	S	S	S	S
ERR2512872	S	S	S	S
ERR2513354	S	S	S	S
ERR2514157	R	S	S	S
ERR2513406	S	S	S	S
ERR2514165	S	S	S	S
ERR2512694	S	S	S	S
ERR2514435	S	S	S	S
ERR2513183	S	S	S	S
ERR2513941	S	S	S	S
ERR2514120	R	S	S	S
ERR2512437	S	S	S	S
ERR2514692	S	S	S	U
ERR2512553	S	S	S	S
ERR2514055	S	S	S	S
ERR2514092	S	S	S	U
ERR2513283	S	S	S	S
ERR2512954	S	S	S	R
ERR2514659	S	S	S	S
ERR2514394	S	S	S	S
ERR2512492	S	S	S	S
ERR2513070	S	S	S	S
ERR2513146	S	S	S	S
ERR2514854	S	S	S	S
ERR2514263	S	S	S	S
ERR2513782	S	S	S	S
ERR2513704	S	S	S	S
ERR2514045	S	S	S	S
ERR2512667	S	S	S	S
ERR2514502	S	S	S	S
ERR2512964	S	S	S	S
ERR2514708	S	S	S	U
ERR2513413	S	S	S	S
ERR2513121	S	S	S	S

ERR2512590	S	S	S	S
ERR2513240	R	R	R	S
ERR2513995	S	S	S	S
ERR2512826	U	U	U	S
ERR2513026	S	S	S	U
ERR2514228	S	S	S	S
ERR2513573	S	S	S	S
ERR2514941	S	S	S	S
ERR2513440	S	S	S	S
ERR2513971	S	S	S	S
ERR2513436	R	R	R	U
ERR2513412	S	S	S	S
ERR2512577	S	S	S	S
ERR2512897	S	S	S	S
ERR2514793	S	S	S	S
ERR2514303	S	S	S	S
ERR2514851	S	S	S	S
ERR2512877	S	S	S	S
ERR2513746	S	S	S	S
ERR2514859	S	S	S	U
ERR2513092	S	S	S	S
ERR2514885	S	S	S	S
ERR2514938	S	S	S	S
ERR2514580	R	S	S	S
ERR2512735	S	S	S	S
ERR2514744	S	S	S	S
ERR2513588	S	S	S	S
ERR2513318	S	S	S	S
ERR2513302	S	S	S	R
ERR2513539	S	S	S	S
ERR2513270	S	S	S	S
ERR2513744	S	S	S	S
ERR2514623	U	U	U	S
ERR2514343	S	S	S	S
ERR2514345	S	S	S	S
ERR2513430	S	S	S	S
ERR2514923	S	S	S	S
ERR2515032	S	S	S	S
ERR2513593	S	S	S	S
ERR2512657	S	S	S	S
ERR2513590	S	S	S	S
SAMN07659655	S	S	S	S
SAMN07658438	S	S	S	S
SAMN07659021	S	S	S	S
ERR2514234	S	S	S	
SAMN07660443	S	S	S	S
SAMN07659330	S	S	U	S
SAMN07658326	S	S	S	S
SAMN07659606	S	S	S	S
SAMN07658817	S	S	S	S
SAMN07659586	S	S	S	S
ERR2514183	S	S	S	
SAMN07658721	S	S	S	S

ERR2513633	S	S	S	S
ERR2513848	S	S	S	S
SAMN07660401	S	S	S	S
ERR2512858	S	S	S	S
ERR2514366	S	S	S	S
ERR2514952	S	S	S	U
SAMN07658843	S	S	S	S
SAMN07658507	S	S	S	S
SAMN07660349	S	S	S	S
ERR2514217	S	S	S	S
ERR2512483	S	S	S	S
ERR2512991	S	S	S	S
ERR2513155	S	S	S	S
ERR2514310	S	S	S	S
ERR2513655	S	S	S	U
ERR2514455	S	S	S	S
ERR2513576	S	S	S	S
ERR2515082	S	S	S	S
ERR2512841	R	S	R	U
ERR2513830	S	S	S	S
ERR2514881	S	S	S	S
ERR2513345	S	S	S	S
ERR2512558	S	S	S	S
ERR2514552	S	S	S	S
ERR2514788	S	S	S	S
ERR2514116	S	S	S	S
ERR2513137	S	S	S	S
ERR2513989	S	S	S	S
ERR2514306	S	S	S	S
ERR2512570	S	S	S	R
ERR2513467	S	S	S	S
ERR2514575	S	S	S	S
ERR2514710	S	S	S	S
ERR2512921	S	S	S	S
ERR2512548	S	S	S	S
ERR2514646	R	R	S	U
ERR2514765	S	S	S	S
ERR2513972	S	S	S	S
ERR2514880	S	S	S	S
ERR2512633	S	S	S	S
ERR2515102	S	S	S	S
ERR2514207	S	S	S	S
ERR2512638	S	S	S	S
ERR2515036	S	S	S	R
ERR2514739	S	S	S	S
ERR2514070	S	S	S	S
ERR2513273	S	S	S	S
ERR2514928	S	S	S	S
ERR2513986	S	S	S	S
ERR2514658	S	S	S	S
ERR2513559	S	S	S	S
ERR2513743	S	S	S	S
ERR2514216	S	S	S	S

ERR2513089	S	S	S	S
ERR2514098	S	S	S	S
ERR2514119	S	S	S	S
ERR2513443	S	S	S	S
ERR2512955	S	S	S	S
ERR2512724	S	S	S	S
ERR2514688	S	S	U	U
ERR2514148	S	S	S	S
ERR2512656	S	S	S	S
ERR2513840	S	S	S	S
ERR2514796	S	S	S	S
ERR2513044	S	S	S	S
ERR2512923	S	S	S	U
ERR2512811	S	S	S	S
ERR2512450	S	S	S	S
ERR2514006	U	S	S	S
ERR2514230	S	S	S	U
ERR2512715	S	S	S	S
ERR2514735	S	S	S	U
ERR2512487	S	S	S	S
ERR2515093	S	S	S	S
ERR2514815	S	S	S	U
ERR2512625	S	S	S	S
ERR2512871	S	S	S	S
ERR2514361	S	S	S	S
ERR2514403	S	S	S	S
ERR2514126	S	S	S	S
ERR2514418	S	S	S	S
ERR2514354	U	S	U	S
ERR2512443	S	S	S	S
ERR2513904	S	S	S	S
ERR2513048	S	S	S	S
ERR2514277	S	S	S	S
ERR2513663	U	U	U	S
ERR2514686	S	S	S	S
ERR2514408	S	S	S	S
ERR2514904	S	S	S	S
ERR2512753	S	S	S	S
ERR2513343	R	S	S	S
ERR2514410	S	S	S	S
ERR2514908	S	S	S	S
ERR2513767	S	S	S	S
ERR2512541	R	S	S	S
ERR2514048	S	S	S	S
ERR2512496	S	S	S	S
ERR2512769	S	S	S	U
ERR2514822	U	U	U	S
ERR2514398	R	R	U	S
ERR2513705	S	S	S	S
ERR2513159	S	S	S	S
ERR2512658	S	S	S	S
ERR2512976	S	S	S	S
ERR2514840	S	S	S	S

ERR2513504	S	S	S	S
ERR2513669	S	S	S	S
ERR2512829	S	S	S	S
ERR2512912	S	S	S	S
ERR2513841	S	S	S	S
ERR2514677	S	S	S	S
ERR2514389	S	S	S	S
ERR2514681	S	S	S	S
ERR2512961	S	S	S	S
ERR2514546	S	S	S	S
ERR2512630	S	S	S	S
ERR2513883	S	S	S	S
ERR2513384	S	S	S	S
ERR2514476	S	S	S	S
ERR2514949	S	S	S	S
ERR2514898	S	S	S	S
ERR2514743	S	S	S	S
ERR2512693	S	S	S	S
ERR2513088	S	S	S	S
ERR2513338	S	S	S	S
ERR2514067	S	S	S	S
ERR2512608	S	S	S	S
ERR2514998	S	S	S	S
ERR2513535	S	S	S	S
ERR2513626	S	S	S	S
ERR2514057	S	S	S	S
ERR2514335	S	S	S	S
ERR2513025	S	S	S	S
ERR2513052	S	S	S	S
ERR2513999	R	S	S	S
ERR2515083	S	S	S	S
ERR2513842	S	S	S	S
ERR2514246	S	S	S	S
ERR2513348	S	S	S	S
ERR2514649	S	S	S	S
ERR2514778	S	S	S	S
ERR2514551	S	S	S	S
ERR2512643	S	S	S	S
ERR2512444	S	S	S	S
ERR2513884	S	S	S	S
ERR2513538	S	S	S	S
ERR2513895	S	S	S	S
ERR2512848	S	S	S	S
ERR2514210	S	S	S	U
ERR2512847	S	S	S	S
ERR2514142	R	S	S	S
ERR2513878	S	S	S	S
ERR2512948	S	S	S	S
ERR2513929	S	S	S	S
ERR2513961	R	S	S	S
ERR2514357	S	S	S	S
ERR2514789	S	S	S	S
ERR2513125	S	S	S	S

ERR2513803	S	S	S	S
ERR2513924	S	S	S	S
ERR2514740	U	S	S	U
ERR2512937	S	S	S	S
ERR2513866	S	S	S	S
ERR2513045	S	S	S	S
ERR2515027	S	S	S	S
ERR2513652	S	S	S	S
ERR2513016	S	S	S	S
ERR2514590	S	S	S	S
ERR2512484	S	S	S	S
ERR2514752	S	S	S	S
ERR2514109	S	S	S	S
ERR2514841	S	S	S	S
ERR2512972	S	S	S	U
ERR2513642	S	S	S	S
ERR2514167	S	S	S	S
ERR2515069	S	S	S	S
ERR2512655	S	S	S	S
ERR2514059	S	S	S	S
ERR2513416	S	S	S	S
ERR2514276	S	S	S	S
ERR2513130	S	S	S	S
ERR2513478	S	S	S	S
ERR2513834	S	S	S	S
ERR2512846	S	S	S	S
ERR2514738	S	S	S	S
ERR2513229	R	R	R	U
ERR2512511	S	S	S	S
ERR2513332	U	S	S	S
ERR2512774	S	S	S	S
ERR2514515	S	S	S	S
ERR2512967	S	S	S	S
ERR2512520	S	S	S	S
ERR2513837	S	S	S	S
ERR2513717	S	S	S	S
ERR2514305	S	S	S	S
ERR2514281	S	S	S	S
ERR2513399	S	S	S	S
ERR2515026	S	S	S	S
ERR2514272	S	S	S	S
ERR2514767	S	S	S	U
ERR2514578	S	S	S	S
ERR2512922	S	S	S	S
ERR2514479	S	S	S	S
ERR2514301	S	S	S	S
ERR2514256	S	S	S	S
ERR2514660	S	S	S	S
ERR2513291	S	S	S	S
ERR2513953	S	S	S	S
ERR2514038	S	S	S	S
ERR2512770	S	S	S	S
ERR2513665	S	S	S	S

ERR2513950	R	R	R	S
ERR2513817	S	S	S	S
ERR2515007	S	S	S	S
ERR2514491	S	S	S	S
ERR2514427	S	S	S	S
ERR2514274	S	S	S	S
ERR2513248	S	S	S	S
ERR2514084	S	S	S	S
ERR2514806	S	S	S	U
ERR2514557	U	U	U	S
ERR2513563	S	S	S	S
ERR2512436	S	S	S	S
ERR2512460	S	S	S	U
ERR2512503	S	S	S	S
ERR2514714	S	S	S	S
ERR2513592	S	S	S	S
ERR2515041	S	S	S	S
ERR2513795	S	S	S	S
ERR2514703	S	S	S	S
ERR2513061	S	S	S	S
ERR2512442	S	S	S	S
ERR2514772	S	S	S	S
ERR2514891	S	S	S	S
ERR2513257	S	S	S	S
ERR2515079	S	S	S	S
ERR2513063	S	S	S	S
ERR2513447	S	S	S	S
ERR2513415	S	S	S	S
ERR2514475	S	S	S	S
ERR2514926	S	S	S	S
ERR2512660	S	S	S	S
ERR2512855	S	S	S	S
ERR2513766	S	S	S	S
ERR2513054	S	S	S	S
ERR2514075	R	R	S	S
ERR2514864	S	S	S	U
ERR2514090	S	S	S	S
ERR2512598	S	S	S	S
ERR2514017	S	S	S	S
ERR2513801	S	S	S	S
ERR2514837	S	S	S	R
ERR2512934	S	S	S	S
ERR2515013	S	S	S	S
ERR2512624	S	S	S	S
ERR2512904	S	S	S	S
ERR2514137	U	U	S	S
ERR2513969	S	S	S	S
ERR2514899	S	S	S	S
ERR2514866	S	S	S	S
ERR2513758	S	S	S	S
ERR2513912	S	S	S	S
ERR2514124	S	S	S	S
ERR2512422	S	S	S	U

ERR2513438	S	S	S	S
ERR2514689	S	S	S	U
ERR2513483	S	S	S	S
ERR2514125	S	S	S	S
ERR2513417	S	S	S	S
ERR2514954	S	S	S	S
ERR2512524	S	S	S	S
ERR2513431	S	S	S	S
ERR2513397	S	S	S	S
ERR2512994	S	S	S	S
ERR2514748	S	S	S	S
ERR2514193	S	S	S	S
ERR2513356	S	S	S	S
ERR2514733	S	S	S	U
ERR2513210	S	S	S	S
ERR2512536	S	S	S	S
ERR2513177	S	S	S	S
ERR2513850	R	R	U	U
ERR2513233	S	S	S	S
ERR2512572	S	S	S	S
ERR2513223	S	S	S	S
ERR2514722	S	S	S	S
ERR2512523	S	S	S	S
ERR2512493	S	S	S	S
ERR2514592	S	S	S	S
ERR2513755	S	S	S	S
ERR2512528	S	S	S	S
ERR2515001	S	S	S	S
ERR2513718	S	S	S	S
ERR2512725	S	S	S	U
ERR2514073	S	S	S	S
ERR2512797	U	S	S	S
ERR2514907	S	S	S	S
ERR2514962	S	S	S	U
ERR2514558	R	S	S	S
ERR2513258	S	S	S	S
ERR2513328	S	S	S	S
ERR2512642	S	S	S	U
ERR2513251	S	S	S	S
ERR2513619	S	S	S	S
ERR2513773	S	S	S	S
ERR2514540	S	S	S	S
ERR2514977	S	S	S	S
ERR2512737	S	S	S	S
ERR2515087	U	U	U	S
ERR2513748	S	S	S	S
ERR2513107	S	S	S	S
ERR2514717	S	S	S	S
ERR2514409	S	S	S	S
ERR2513500	S	S	S	S
ERR2514750	S	S	S	S
ERR2512732	S	S	S	R
ERR2512947	S	S	S	S

ERR2514823	S	S	S	S
ERR2513204	S	S	S	S
ERR2512960	S	S	S	S
ERR2513071	S	S	S	U
ERR2514022	S	S	S	U
ERR2512817	S	S	S	S
ERR2513383	S	S	S	S
ERR2513641	S	S	S	U
ERR2513448	S	S	S	S
ERR2513955	S	S	S	S
ERR2513237	S	S	S	S
ERR2514862	S	S	S	S
ERR2512716	U	S	S	S
ERR2514328	S	S	S	S
ERR2512604	S	S	S	S
ERR2514291	R	S	S	S
ERR2513546	S	S	S	S
ERR2515039	S	S	S	S
ERR2513900	S	S	S	S
ERR2512900	S	S	S	S
ERR2513004	S	S	S	S
ERR2513903	S	S	S	S
ERR2514836	S	S	S	S
ERR2514138	S	S	S	S
ERR2513709	S	S	S	S
ERR2512416	S	S	S	S
ERR2514555	S	S	S	S
ERR2512783	S	S	S	S
ERR2514617	S	S	S	S
ERR2513187	S	S	S	S
ERR2512911	S	S	S	S
ERR2514858	S	S	S	S
ERR2513149	S	S	S	S
ERR2515062	S	S	S	S
ERR2514352	S	S	S	R
ERR2515022	S	S	S	S
ERR2515077	S	S	S	S
ERR2513501	S	S	S	S
ERR2512825	S	S	S	S
ERR2513737	S	S	S	S
ERR2514727	S	S	S	S
ERR2512689	S	S	S	S
ERR2513621	S	S	S	S
ERR2512592	S	S	S	S
ERR2513897	S	S	S	S
ERR2513067	U	U	U	S
ERR2512804	S	S	S	S
ERR2514011	S	S	S	S
ERR2513686	S	S	S	S
ERR2512429	S	S	S	S
ERR2513262	S	S	S	U
ERR2513098	S	S	S	S
ERR2514002	S	S	S	S

ERR2512677	S	S	S	S
ERR2514315	S	S	S	S
ERR2513611	S	S	S	S
ERR2512615	S	S	S	S
ERR2512767	S	S	S	U
ERR2512733	S	S	S	S
ERR2514813	S	S	S	S
ERR2513672	U	S	S	S
ERR2513902	S	S	S	S
ERR2514665	S	S	S	S
ERR2513043	U	U	U	S
ERR2514485	S	S	S	S
ERR2514760	R	R	R	S
ERR2513160	R	S	U	U
ERR2514503	R	R	R	S
ERR2513168	S	S	S	S
ERR2514973	S	S	S	S
ERR2512880	S	S	S	S
ERR2513334	S	S	S	S
ERR2514200	R	S	S	S
ERR2514852	S	S	S	S
ERR2514673	S	S	S	S
ERR2514178	U	U	U	S
ERR2515089	S	S	S	S
ERR2513181	S	S	S	U
ERR2514655	S	S	S	S
ERR2512864	S	S	S	S
ERR2514215	S	S	S	S
ERR2514794	S	S	S	S
ERR2514161	S	S	S	S
ERR2513491	S	S	S	S
ERR2513144	S	S	S	S
ERR2514261	S	S	S	S
ERR2513994	R	R	R	R
ERR2513670	S	S	S	S
ERR2513628	S	S	S	S
ERR2514442	S	S	S	S
ERR2514106	R	R	S	S
ERR2514535	U	S	S	S
ERR2513875	S	S	S	S
ERR2514325	S	S	S	S
ERR2513542	S	S	S	S
ERR2513018	S	S	S	S
ERR2513664	S	S	S	S
ERR2513925	S	S	S	S
ERR2512494	S	S	S	S
ERR2512526	S	S	S	S
ERR2513700	S	S	S	S
ERR2512743	S	S	S	S
ERR2513497	S	S	S	S
ERR2512699	S	S	S	S
ERR2513738	S	S	S	S
ERR2514953	S	S	S	S

ERR2513217	S	S	S	S
ERR2512741	S	S	S	S
ERR2513140	S	S	S	S
ERR2513605	U	U	U	S
ERR2514030	S	S	S	S
ERR2513509	S	S	U	U
ERR2512812	R	S	S	S
ERR2514638	S	S	S	S
ERR2513948	S	S	S	S
ERR2512601	S	S	S	U
ERR2512987	S	S	S	S
ERR2512557	S	S	S	S
ERR2512970	S	S	S	S
ERR2513658	S	S	S	U
ERR2514705	S	S	S	S
ERR2514803	S	S	S	S
ERR2514702	S	S	S	S
ERR2513632	S	S	S	S
ERR2513101	S	S	S	S
ERR2513165	R	R	S	S
ERR2514018	S	S	S	S
ERR2514390	S	S	S	U
ERR2514083	S	S	S	S
ERR2515017	S	S	S	S
ERR2513561	S	S	S	S
ERR2513617	S	S	S	S
ERR2512453	S	S	S	S
ERR2514415	S	S	S	S
ERR2514316	S	S	S	S
ERR2514250	S	S	S	S
ERR2512698	S	S	S	S
ERR2512892	S	S	S	S
ERR2514446	S	S	S	S
ERR2513231	S	S	S	S
ERR2514832	S	S	S	R
ERR2514218	S	S	S	S
ERR2513317	S	S	S	S
ERR2513109	S	S	S	S
ERR2513117	S	S	S	S
ERR2514628	S	S	S	S
ERR2513255	S	S	S	S
ERR2514270	S	S	S	S
ERR2513179	U	U	U	S
ERR2513361	S	S	S	S
ERR2512549	S	S	S	R
ERR2512556	S	S	S	S
ERR2514513	S	S	S	S
ERR2513790	S	S	S	S
ERR2513078	S	S	S	S
ERR2513011	U	U	U	S
ERR2512568	S	S	S	S
ERR2512756	S	S	S	S
ERR2513565	S	S	S	U

ERR2513861 S S S S
ERR2512943 S S S S
ERR2512546 S S S S
ERR2513661 S S S S
ERR2512485 S S S S
ERR2514146 S S S S
ERR2513264 S S S S
ERR2513964 U S S S
ERR2514995 S S S S
ERR2512434 S S S S
ERR2514706 S S S S
ERR2513911 S S S S
ERR2512621 S S S S
ERR2513708 S S S S
ERR2513396 U S U U
ERR2514488 U S S S
ERR2513420 S S U S
ERR2513657 S S U S
ERR2513275 S S S S
ERR2513006 S S S S
ERR2513512 S S S S
ERR2514308 S S S S
ERR2512883 U U U S
ERR2514525 S S S S
ERR2513750 S S S U
ERR2514350 U S S U
ERR552626,ERR552625 R R R S
ERR551780,ERR551781 R R R R
ERR108437 S S S S
ERR550786,ERR550787 R R R S
ERR551645,ERR551644 R R S R
ERR067716 R S S S
ERR067717 R R R S
ERR067680 S S S S
ERR498360 ERR498361 ERR498362 S S S S
ERR067682 S S S S
ERR228038 R R R S
ERR067683 R S S S
ERR552214 S S S S
ERR552601 S S S S
ERR067684 S S S S
ERR067685 S R S S
ERR553043 S S S S
ERR067686 R S S S
ERR067687 R R R R
ERR067688 S S S S
ERR067689 S S S S
ERR067690 S S S R
ERR067691 R R S S
ERR137251 S S S S
ERR498350 ERR498351 ERR498352 S S S S
ERR550986 S S S S
ERR108505 S S S S

ERR137252	S	S	S	S
ERR108506	S	S	S	R
ERR137253	S	S	S	S
ERR552175	S	S	S	S
ERR137254	R	R	R	S
ERR137255	R	R	S	R
ERR108507	R	R	R	S
ERR108508	S	S	S	S
ERR108509	S	S	S	S
ERR108510	R	R	S	R
ERR108511	R	R	S	S
ERR137256	S	S	S	S
ERR137257	R	R	S	S
ERR552755	S	S	S	S
ERR137258	S	S	S	S
ERR137260	R	R	S	S
ERR137261	S	S	S	S
ERR2514068	S	S	S	S
ERR2512850	S	S	S	S
ERR2514857	S	S	S	S
ERR2513296	S	S	S	S
ERR2513536	S	S	S	S
ERR2514599	S	S	S	U
ERR2514695	U	S	U	S
ERR2514771	S	S	S	S
ERR2513473	S	S	S	S
ERR2514723	S	S	S	S
ERR2514579	S	S	S	S
ERR2513124	S	S	S	S
ERR2514460	S	S	S	S
ERR2513009	U	S	S	S
ERR2513977	S	S	U	S
ERR2513662	U	U	U	S
ERR2513794	S	S	S	U
ERR2513015	S	S	S	S
ERR2512842	S	S	S	S
ERR2514108	S	S	S	S
ERR2514521	S	S	S	S
ERR2514679	R	S	S	S
ERR2514981	U	S	S	S
ERR2514514	S	S	S	U
ERR2515070	S	S	S	S
ERR2513764	S	S	S	S
ERR2514351	S	S	S	S
ERR2513735	U	U	U	S
ERR2513959	S	S	S	U
ERR2513762	S	S	S	S
ERR2512773	S	S	S	S
ERR2512941	S	S	S	U
ERR137262	R	R	R	S
ERR137263	R	R	R	R
ERR137264	R	R	S	S
ERR137266	S	S	S	S

ERR137267	R	S	S	S
ERR137268	S	S	S	S
ERR117468	R	R	S	S
ERR2199833	S	S	S	S
ERR2199834	S	S	S	U
ERR137269	R	R	S	S
ERR553075	S	S	S	S
ERR137270	S	S	S	S
ERR550710	S	S	S	S
ERR137271	R	R	R	R
ERR137273	R	R	S	S
ERR137274	R	R	S	S
ERR137275	R	R	R	R
ERR137277	R	R	R	S
ERR137278	S	S	S	S
ERR137279	S	S	S	S
ERR137280	S	S	S	S
ERR2199835	S	S	S	S
ERR2199836 ERR2199837	R	R	R	R
ERR137281	R	R	S	S
ERR2199838	S	S	S	S
ERR137282	R	R	S	S
ERR2199839	S	S	S	S
ERR2199840	S	S	S	S
ERR2199841	R	S	S	S
ERR551349	S	S	S	S
ERR137283	R	R	S	S
ERR137284	R	R	R	R
ERR137285	S	S	S	S
ERR144542	R	R	S	R
ERR2199842	S	S	S	U
ERR144543	R	R	S	S
ERR552268	S	S	S	S
ERR144544	S	S	S	S
ERR144545	R	S	S	R
ERR144546	S	R	S	S
ERS458469	S	S	S	S
ERR552689 ERR552690	S	S	S	S
ERS457423	S	S	S	S
ERR144547	S	S	S	S
ERR144548	R	R	S	S
ERS457826	S	S	S	S
ERR551470 ERR551471	S	S	S	S
ERR144549	R	R	S	S
ERR144550	S	S	R	S
ERR144551	R	R	R	S
ERR144552	S	S	S	S
ERR144555	S	S	S	S
ERR144556	R	R	S	S
ERR144557	S	S	S	S
ERR144558	R	S	S	S
ERR144559	R	S	S	S
ERR144560	R	R	R	R

ERR144561	R	R	S	S
ERR144562	R	R	S	S
ERR144563	R	R	R	S
ERR552233	S	S	S	S
ERR144564	R	S	S	S
ERR144565	R	S	R	S
ERR552054	S	S	S	S
ERR144566	R	R	R	S
ERR144567	R	R	R	R
ERR551783	S	S	S	S
ERR2199843 ERR2199844 ERR2199845	S	S	S	S
ERR550617	S	S	S	S
ERR144569	S	S	S	S
ERR553278	S	S	S	S
ERR144570	R	R	S	S
ERR144571	R	R	R	R
ERR144573	R	R	R	S
ERR2199846	S	S	S	S
ERR2199847	S	R	S	S
ERR144574	R	R	R	S
ERR144575	R	R	S	S
ERR144576	R	S	S	S
ERR144577	S	S	S	S
ERR144578	S	S	S	S
ERR144579	R	R	R	S
ERR144580	R	S	S	S
ERR144581	R	R	S	R
ERR144582	S	S	S	S
ERR551238	R	R	R	S
ERR144585	S	S	S	S
ERR552027	S	S	S	S
ERR144587	R	R	R	S
ERR144588	R	S	S	S
ERR144590	R	R	S	S
ERR2199848	S	S	S	S
ERR144592	S	S	S	S
ERR144593	R	R	S	S
ERR144594	R	S	S	S
ERR144595	R	R	R	R
ERR551837	S	S	S	S
ERR2199849	S	S	S	S
ERR144596	R	S	R	R
ERR144597	R	R	S	S
ERR144598	R	S	S	S
ERR144599	R	S	S	S
ERR144600	R	R	R	S
ERR550948	S	S	S	S
ERR551977 ERR551978	S	S	S	S
ERR552948	S	S	S	S
ERR144602	S	S	S	S
ERR144603	S	S	S	S
ERR108453	R	R	R	R
ERR2199850	S	S	S	S

ERR144604	R	S	S	S
ERR551632	S	S	S	S
ERR553219	S	S	S	S
ERR551732	S	S	S	S
ERR551243	S	S	S	S
ERR552183	S	S	S	S
ERR550840	S	S	S	S
ERR553346	S	S	S	S
ERR144605	R	S	S	S
ERR144606	R	R	R	R
ERR144607	R	R	R	R
ERR144608	R	S	R	R
ERR144609	R	S	S	S
ERR551598	S	S	S	S
ERR144610	S	S	S	S
ERR144611	R	R	S	S
ERR144613	S	S	S	S
ERR144614	S	S	S	S
ERR144616	S	S	S	S
ERR144618	R	S	S	S
ERR144619	R	R	R	S
ERR553160	R	S	S	S
ERR144620	R	R	R	R
ERR144621	S	S	S	R
ERR144622	R	R	R	R
ERR552904	S	S	S	S
ERR144623	R	R	S	S
ERR144624	R	R	R	S
ERR551050	S	S	S	S
ERR553235	S	S	S	S
ERR2199851	S	S	S	S
ERR144625	R	R	S	S
ERR144626	S	S	S	S
ERR144627	R	S	R	S
ERR144628	R	R	R	S
ERR228046	R	R	S	S
ERR2199852	S	S	S	S
ERR553255	S	S	S	S
ERR2199853 ERR2199854	R	R	S	S
ERR144629	S	S	S	S
ERR144630	S	S	S	S
ERR144631	R	R	R	S
ERR551533	R	S	R	S
ERR144632	R	R	R	S
ERR144633	R	R	R	S
ERR551631	S	S	S	S
ERR144634	R	R	R	S
ERR144635	R	R	R	R
ERR144636	R	R	R	R
ERR158569	S	R	S	S
ERR158570	R	R	S	S
ERR158571	S	S	S	S
ERR158572	S	S	R	S

ERR552581 S S S S
ERR552665 S S S S
ERR158575 S S S S
ERR158576 R R R S
ERR2199855 ERR2199856 S S S S
ERR158577 S S S S
ERR2199857 S S S S
ERR158579 R R S R
ERR551426 S S S S
ERR553293 S S S S
ERR158580 R R S R
ERR158581 R R R S
ERR551483 S S S S
ERR552499,ERR552498 R R R R
ERR551714 R S S S
ERR551420 S S S S
ERR551867 S S S S
ERR2199858 S S S S
ERR158582 S S S S
ERR551793,ERR551795,ERR551794 R R R S
ERR550908,ERR550906,ERR550907 R R R R
ERR551499,ERR551500,ERR551501 R R S S
ERR552164,ERR552165 R R S R
ERR158585 R R R S
ERR552711,ERR552712 R R R S
ERR158587 R R R R
ERR550926 S S S S
ERR158588 R R R R
ERR551741 S S S S
ERR158589 R S R S
ERR158590 R R R S
ERR158591 S S S R
ERR158592 R R S S
ERR158593 R R R R
ERR158595 R R S S
ERR551912 S S S S
ERR551357 S S S S
ERR158596 R R S S
ERR158598 R R S S
ERR552535 S S S S
ERR228062 R R R R
ERR227975 R R S S
ERR227983 S R R S
ERR227991 S S S S
ERR227999 S S S S
ERR551779 S S S S
ERR228007 S S S S
ERR228015 S S S S
ERR228023 S S S S
ERR228031 S S S S
ERR551633 S S S S
ERR228039 R R R R
ERR228047 S S S S

ERR2199859	R	S	S	S
ERR228055	S	R	S	S
ERR228063	S	S	S	S
ERR227976	R	R	S	S
ERR227984	S	S	S	S
ERR227992	S	S	S	S
ERR228000	S	S	S	S
ERR228008	S	S	S	S
ERR228016	R	R	R	S
ERR228024	R	R	R	R
ERR2199860	S	S	S	S
ERR228032	R	R	S	S
ERR228040	R	S	S	S
ERR551552	S	S	S	S
ERR551580	S	S	S	S
ERR228048	R	S	S	S
ERR228056	R	R	R	R
ERR228064	R	R	S	S
ERR227977	R	R	R	S
ERR227985	R	R	R	R
ERR228001	R	R	S	R
ERR228009	R	R	S	S
ERR228017	R	R	S	S
ERR228025	R	R	S	S
ERR228033	S	S	S	S
ERR228049	R	R	R	R
ERR228057	S	S	S	S
ERR228065	S	S	S	S
ERR553301	S	S	S	S
ERR227994	R	R	R	S
ERR228002	S	S	S	S
ERR228010	S	S	S	S
ERR228018	S	S	S	S
ERR228026	R	S	S	S
ERR228034	S	S	S	S
ERR228042	R	R	S	R
ERR552465	S	S	S	S
ERR228058	R	S	S	S
ERR550875	S	S	S	S
ERR227979	S	S	S	S
ERR552130	S	S	S	S
ERR227987	R	R	S	S
ERR228003	R	R	S	S
ERR228011	S	S	S	S
ERR228019	R	R	S	S
ERR228027	S	S	S	S
ERR552186	S	S	S	S
ERR228035	S	S	S	S
ERR228043	R	R	S	R
ERR552897,ERR552896	R	R	R	R
ERR552099,ERR552098	R	R	R	R
ERR228059	R	R	R	S
ERR551435	S	S	S	S

ERR228067 S S S S
ERR552142,ERR552144,ERR552143 R R R R
ERR551380,ERR551381 R R S S
ERR553384 ERR553385 S S S S
ERR227980 R S R S
ERR551295,ERR551296 R R R R
ERR227988 S S S S
ERR551244 S S S S
ERR227996 R R R S
ERR228004 S S S S
ERR228012 R R R S
ERR228028 R R R R
ERR228036 R R R R
ERR228044 R S S S
ERR551690 S S S S
ERR228060 S S S S
ERR552449 S S S S
ERR228068 S S S S
ERR552251 ERR552252 S S S S
ERR227981 R R S R
ERR552881 S S S S
ERR552697 S S S S
ERR227997 R S S S
ERR228005 S S S S
ERR551562 S S S S
ERR228013 R R R S
ERR228021 S S S R
ERR228029 R R S S
ERR228037 R R S S
ERR228045 S S S S
ERR228053 S S S S
ERR228061 R R R S
ERR228069 S S S S
ERR229915 R R R S
ERR229923 R R R S
ERR229931 R R R R
ERR229939 S S S S
ERR229947 R R S S
ERR229955 R R R S
ERR229971 R R R R
ERR229979 S S S S
ERR229987 S S S S
ERR229995 R R S S
ERR230003 R R S S
ERR229924 R R R S
ERR229932 R R R S
ERR229940 R R S S
ERR229948 S S S S
ERR229956 S S S S
ERR229964 R S S S
ERR229972 S S S S
ERR229988 R R S S
ERR230004 S S S S

ERR229917 S S S S
ERR552920 S S S S
ERR229925 R S S S
ERR229941 S S S S
ERR229949 R R R R
ERR229957 R S S S
ERR229965 S R R S
ERR229973 S S S S
ERR229981 S S S S
ERR229989 R S S S
ERR551811,ERR551809,ERR551810 R R R R
ERR553340 S S S S
ERR552816,ERR552814,ERR552815 R R R R
ERR229997 R R S S
ERR230005 R R R R
ERR552106,ERR552105 R R S R
ERR552609 S S S S
ERR552970,ERR552971 R R R R
ERR229918 R R R S
ERR551925,ERR551926 R R S S
ERR229926 R R R S
ERR498357 ERR498358 ERR498359 S S S S
ERR553137 S S S S
ERR551106 S S S S
ERR229934 S S S S
ERR551558,ERR551556,ERR551557 R R R S
ERR229942 S S S S
ERR552447,ERR552446 R R R R
ERR552607,ERR552606 R R R R
ERR229950 S S S S
ERR551877,ERR551878 R R R S
ERR229958 S S S S
ERR552950,ERR552951 R R S S
ERR229966 R R R S
ERR552215,ERR552217,ERR552216 R R R R
ERR229982 R R R R
ERR552123,ERR552122 R R S S
ERR229990 S R R S
ERR551986,ERR551987 R R R S
ERR552347,ERR552346 R R R R
ERR551429,ERR551428 R R R R
ERR551767,ERR551768 R R R R
ERR229998 R R R S
ERR2199861 S S S S
ERR230006 R R R S
ERR229919 S S S S
ERR229927 R R S R
ERR550642 S S S S
ERR229935 R S S S
ERR229943 S S S S
ERR229959 S S S R
ERR551884 S S S S
ERR229967 R S S S

ERR552985	S	S	S	S
ERR229975	S	S	S	S
ERR229983	R	S	S	S
ERR552511	S	S	S	S
ERR553112	S	S	S	S
ERR551486	S	S	S	S
ERR229991	S	S	S	S
ERR229999	R	S	R	S
ERR230007	R	R	R	S
ERR229920	S	S	S	R
ERR553079	S	S	S	S
ERR229928	S	S	S	R
ERR229936	R	R	R	R
ERR229944	S	S	S	S
ERR229960	R	R	S	S
ERR229968	R	R	R	S
ERR229976	R	R	R	S
ERR229984	R	R	R	S
ERR229992	S	S	S	S
ERR230008	R	R	S	S
ERR552567	S	S	S	S
ERR229921	R	R	R	S
ERR229929	R	R	R	R
ERR229937	S	S	S	S
ERR229945	S	S	S	S
ERR229953	R	R	S	S
ERR229961	R	S	R	S
ERR229969	R	R	S	R
ERR551433	S	S	S	S
ERR229977	S	S	S	S
ERR229985	S	S	S	S
ERR552059	S	S	S	S
ERR229993	R	R	R	R
ERR230001	S	S	S	S
ERR229922	R	R	R	S
ERR229938	S	S	S	R
ERR229946	R	R	R	S
ERR229954	S	S	S	S
ERR229962	R	R	R	S
ERR229970	S	S	S	S
ERR229978	R	R	R	S
ERR229986	S	S	S	S
ERR229994	R	R	S	S
ERR230002	S	S	S	S
ERR230010	S	S	S	S
ERR234652	S	S	S	S
ERR234653	R	R	S	S
ERR234654	R	R	R	S
ERR234655	R	S	S	S
ERR234657	R	R	R	R
ERR2199862 ERR2199863	S	S	S	S
ERR234658	R	R	R	R
ERR552280	S	S	S	S

ERR234660 R R R S
ERR234661 R R R R
ERR234662 R S R S
ERR234663 S S S S
ERR234664 S S S S
ERR2199864 S S S S
ERR234666 R R S S
ERR2199865 S S S S
ERR234667 S S S S
ERR552715 S S S S
ERR551072 S S S S
ERR234668 R R R R
ERR234669 R R S R
ERR552976 S S S S
ERR234671 R R S R
ERR2199866 ERR2199867 S S S S
ERR234672 R R R R
ERR2199868 S S S S
ERR234674 R S S S
ERR2199869 ERR2199870 S S S S
ERR234683 R S S S
ERR234684 S S S S
ERR234685 S S S S
ERR2199871 S S S S
ERR234686 S S S S
ERR551742 S S S S
ERR234687 S S S S
ERR551778 S S S S
ERR234688 R S R R
ERR234689 S S S S
ERR552884 S S S S
ERR234690 S S S S
ERR234691 S S S S
ERR234692 R R S S
ERR552630 S S S S
ERR2199872 ERR2199873 S S S S
ERR2199874 S S S S
ERR551149 S S S S
ERR552863 S S S S
ERR234693 R S S S
ERR234694 S S S S
ERR234695 S S S S
ERR234696 R S S R
ERR234697 S S S S
ERR552353 S S S S
ERR551881 ERR551882 S S S S
ERR2199875 S S S S
ERR234698 R R S S
ERR234699 R S S R
ERR553174 S S S S
ERR551116 S S S S
ERR550871 S S S S
ERR498391,ERR498392 S S S S

ERR551022 S S S S
ERR552578 R S S S
ERR2199876 ERR2199877 S S S S
ERR2199878 S S S S
ERR551350 S S S S
ERR550835 S S S S
ERR553359 S S S S
ERR553062 S S S S
ERR551358 S S S S
ERR551080 S S S S
ERR2199879 S S S S
ERR552326 R R S S
ERR550974 S S S S
ERR2199880 ERR2199881 S S S S
ERR552315 S S S S
ERR2199882 ERR2199883 S S S S
ERR552582 R S S S
ERR552960 S S S S
ERR551695 S S S S
ERR551911 S S S S
ERR552006 S S S S
ERR551662 S S S S
ERR552372 S S S S
ERR551389 S S S S
ERR2199884 S S S S
ERR552686 S S S S
ERR553016 S S S S
ERR551406 S S S S
ERR2199885 ERR2199886 S S S S
ERR2199887 S S S S
ERR551738,ERR551737 R R R R
ERR552413,ERR552412 R R S S
ERR551735,ERR551736 R R R R
ERR553179,ERR553178 R R R S
ERR551285,ERR551284 R R R R
ERR551952,ERR551951 R R S S
ERR552301,ERR552302 R R R S
ERR551466,ERR551467 R R R S
ERR552918 S S S S
ERR551990 S S S S
ERR550704 S S S S
ERR552066 S S S S
ERR2199888 S S S S
ERR553225 S S S S
ERR552722 S S S S
ERR551862,ERR551861 R R S R
ERR550830,ERR550829 R R R R
ERR552545,ERR552546 R R R R
ERR550838,ERR550837 R R R R
ERR552398,ERR552399 R R S R
ERR551787 R S S S
ERR552602 S S S S
ERR552007,ERR552008 R R R R

ERR2199889	S	S	S	S
ERR2199890	S	S	S	S
ERR2199891	S	S	S	S
ERR552269	S	S	S	S
ERR2199892	S	S	S	S
ERR2199893	R	S	S	S
ERR551375	S	S	S	S
ERS458259	S	S	S	S
ERS458497	R	S	S	S
ERS458130	S	S	S	S
ERS457929	S	S	S	S
ERR553084	S	S	S	S
ERR551236	R	R	R	R
ERR2199894	S	S	S	S
ERR553136	S	S	S	S
ERR550936	R	S	S	S
ERR550709	S	S	S	S
ERR552827	S	S	S	S
ERR551902	S	S	S	S
ERR552155	S	S	S	S
ERR551041	S	S	S	S
ERR552639	S	S	S	S
ERR552402	S	S	S	S
ERR2199895	S	S	S	S
ERR2199896	S	S	S	S
ERR551989	S	S	S	S
ERR551282	S	S	S	S
ERR550998	S	S	S	S
ERR551117	S	S	S	S
ERR551065	S	S	S	S
ERR551110	S	S	S	S
ERR552528	S	S	S	S
ERR552871	S	S	S	S
ERR552207	S	S	S	S
ERR553280	S	S	S	S
ERR553287	S	S	S	S
ERR552691	S	S	S	S
ERR551893	S	S	S	S
ERR552759	S	S	S	S
ERR552657	S	S	S	S
ERR552804	S	S	S	S
ERR2199897	R	S	S	S
ERR551663	R	S	S	S
ERR2199898	S	S	S	S
ERR552835	S	S	S	S
ERR551267	S	S	S	S
ERR553159	S	S	S	S
ERR553048	S	S	S	S
ERR551225	S	S	S	S
ERR552290	S	S	S	S
ERR552178	S	S	S	S
ERR550957	S	S	S	S
ERR551657	S	S	S	S

ERR550700 S S S S
ERS458315 S S S S
ERR551763 S S S S
ERS457433 S S S S
ERS458341 S S S S
ERR552564 S S S S
ERS458107 S S S S
ERR551868 S S S S
ERR552680 S S S S
ERR550678 S S S S
ERR552783 ERR552784 S S S S
ERR552856 S S S S
ERR2199899 ERR2199900 S S S S
ERR2199901 ERR2199902 S S S S
ERR551761 S S S S
ERR552866 S S S S
ERR552757 S S S S
ERR553271 S S S S
ERR551505 S S S S
ERR552335 S S S S
ERR2199903 S S S S
ERR550938 S S S S
ERR553055 S S S S
ERR550924 S S S S
ERR551273 S S S S
ERR552126 S S S S
ERR551933 S S S S
ERR551374 S S S S
ERR552058 S S S S
ERR553202 S S S S
ERR552761 S S S S
ERR552085 S S S S
ERR551602 S S S S
ERR550773,ERR550772 R R R R
ERR552978,ERR552977 R R R S
ERR552931,ERR552932 R R S R
ERR2199904 S S S S
ERR551528,ERR551527 R R S R
ERR552973,ERR552974 R R R S
ERR552244,ERR552245 R R R R
ERR551016,ERR551015 R R R R
ERR551087 S S S S
ERR551413 S S S S
ERR550630 S S S S
ERR550714 S S S S
ERR552820 S S S S
ERR551993 S S S S
ERR551840 S S S S
ERR553199 S S S S
ERR551013 S S S S
ERR551188 S S S S
ERS458746 S S S S
ERS457436 R S S R

ERR550755 R S S R
ERR551434 S S S S
ERR2199905 S S S S
ERR551849 S S S S
ERR551463 S S S S
ERR550745 S S S S
ERR551974,ERR551973 R R R R
ERR2199906 S S S S
ERR552322,ERR552321 R R R R
ERR551360,ERR551361,ERR551362 R R R R
ERR552683 S S S S
ERR551351 S S S S
ERR551301 S S S S
ERR2199907 ERR2199908 S S S S
ERR2199909 S S S S
ERS457167 S S S S
ERS457639 S S S S
ERR550811 S S S S
ERS457170 S S S S
ERR551490 S S S S
ERR552313 ERR552314 S S S S
ERR2199910 ERR2199911 S S S S
ERR2199912 S S S S
ERR550865 S S S S
ERR552168 S S S S
ERR2041696 R R S S
ERR2041698 R R R R
ERR2041699 ERR2041700 R R R R
ERR2041701 ERR2041702 R R R R
ERR2041703 ERR2041704 R R S R
ERR2041705 ERR2041706 R R S R
ERR2041707 ERR2041708 R R S R
ERR2041709 R R R R
ERR2041710 ERR2041711 R R S R
ERR2041712 R R R R
ERR2041713 ERR2041714 R R R R
ERR2199913 S S S S
ERR2041715 ERR2041716 R R S R
ERR2199914 R R R R
ERR2041717 R R R R
ERR552212 S S S S
ERR551842 S S S S
ERR551572 S S S S
ERR551388 S S S S
ERR552177 R S S S
ERR552391 R R S S
ERR553119 S S S S
ERR551077 S S S S
ERR550846 S S S S
ERR551277 S S S S
ERR551415 S S S S
ERR551078 S S S S
ERR552355 S S S S

ERR551097 S S S S
ERR552272 S S S S
ERR551458 S S S S
ERR2199915 S S S S
ERR552385 S S S S
ERR550628 S S S S
ERR553231 S S S S
ERR552943 S S S S
ERS457076 R S S R
ERS457803 S S S S
ERR550791 S S S S
ERS457122 R R R R
ERR498287 ERR498288 ERR498289 S S S S
ERR553374 S S S S
ERR2199916 S S S S
ERR553158 S S S S
ERS456829 S S S S
ERR2199917 S S S S
ERR553277 S S S S
ERR551441 S S S S
ERS457914 S S S S
ERR553239 S S S S
ERR550921 S S S S
ERR2199918 S S S S
ERR2199919 S S S S
ERR552193 R R R R
ERR2199920 S S S U
ERR552727 S S S S
ERR2199921 S S S U
ERR550619 R R R R
ERR2199922 S S S U
ERR2199923 S S S U
ERR2199924 S S S U
ERR2199925 S S S U
ERR2199926 S S S U
ERR2199927 S S S U
ERR2199928 S S S U
ERR2199929 S S S U
ERR2199930 S S S U
ERR2199931 S S S U
ERR2199932 S S S U
ERR2199933 S S S U
ERR2199934 S S S U
ERR2199935 S S S U
ERR2199936 S S S U
ERR2199937 S S S U
ERR553324 S S S U
ERR2199938 S S S U
ERR551045 R R S R
ERR551941 ERR551942 S S S U
ERR2199939 S S S U
ERR619080 S S S U
ERR553058 R R R S

ERR552352 R S S R
ERR2199940 S S S U
ERR2199941 S S S U
ERR2199942 S S S U
ERR2199943 S S S U
ERR2199944 S S S U
ERR551854 ERR551855 S S S U
ERR2199945 S S S U
ERR551194 ERR551195 R U R R
ERR2199946 S S S U
ERR2199947 S S S U
ERR552491 R R R R
ERR551627 S S S S
ERR2199948 S S S S
ERR2199949 S S S U
ERR552754 R R R R
ERR552456 R R S R
ERR2199950 S S S U
ERR552108 ERR552109 S S S U
ERR2199951 S S S U
ERR552710 R R S R
ERR550660 S S S S
ERR2199952 S S S U
ERR2199953 ERR2199954 S S S U
ERR552013 S S S S
ERR2199955 S S S U
ERR551608 S S S S
ERR2199956 S S S U
ERR551025 S S S S
ERR551229 S S S S
ERR551170 S S S S
ERR2199957 S S S S
ERR551018 S S S S
ERR553357 R R S R
ERR551196 R R R R
ERR551612 S S S S
ERR550969 R S S S
ERR552379 R S S S
ERR551029 R S S S
ERR2199958 S S S S
ERR550947,ERR550946 R S S S
ERR2199959 ERR2199960 S S S S
ERR552271 S S S S
ERR552477 S S S S
ERR552828 S S S S
ERR552706 S S S S
ERR552583 S S S S
ERR551520 S S S S
ERR2199961 S S S S
ERR552882,ERR552883 R R R R
ERR2199962 S S S S
ERR2199963 S S S S
ERR2199964 S S S S

ERS458588 R S S S
ERS457550 S S S S
ERS457476 R S R R
ERS457695 S S S S
ERR551091 R S S S
ERR553332 R S S S
ERR552832 S S S S
ERR551105,ERR551104 R R R R
ERR551063,ERR551062 R R R S
ERR550895,ERR550894 R R S S
ERR552226,ERR552225 R R S R
ERR550980,ERR550981 R R R S
ERR551582,ERR551581 R R R R
ERR550713 S S S S
ERR550784,ERR550785 R R R S
ERR551232 S S S S
ERR553098,ERR553097 R R S S
ERR551910,ERR551909 R R S R
ERR552638 S S S S
ERR2199965 S S S S
ERR2199966 S S S S
ERR550761 S S S S
ERR2199967 S S S S
ERR551908 S S S S
ERR550796 S S S S
ERR552137 S S S S
ERR552957 S S S S
ERR551252 S S S R
ERR550699 S S S S
ERR552227 S S S S
ERR552436 S S S S
ERR552145 S S S R
ERR550746 S S S S
ERR551344 S S S S
ERR2199968 ERR552695 S S S S
ERR551079 S S S U
ERR552811 S S S S
ERR2199969 S S S U
ERR2199970 S S S U
ERR2199971 S S S U
ERR2199972 S S S U
ERR2199973 S S S U
ERR551609 R U R R
ERR553387 S S S S
ERR551071 S S S U
ERR2199974 S S S S
ERR550741 R R R R
ERR2199975 S S S U
ERS457092 S S S S
ERR552518 R R R R
ERR553105 R R R U
ERR2199976 S S S U
ERS458656 S S S S

ERR551733 S S S S
ERR553230 R R R R
ERR2199977 S S S U
ERR552083 R U R R
ERR552821 ERR552822 R U R R
ERR551988 S S S S
ERR552637 R U R R
ERR2199978 S S S S
ERR2199979 S S S U
ERR2199980 S S S S
ERS457325 R R R S
ERR2199981 S S S U
ERR552396 ERR552397 S S S U
ERR551825 R S S R
ERR2199982 S S S U
ERR551594 S S S S
ERR552464 S S S S
ERR551030 R S S S
ERR551968 S S S S
ERR550747 S S S S
ERS458403 S S S S
ERS457053 R S S S
ERS456997 S S S S
ERS457811 S S S S
ERR552861 S S S S
ERR550935 S S S S
ERR2199983 S S S S
ERR551047 S S S S
ERR552892 S S S S
ERR552758 S S S S
ERR552945 S S S S
ERR552378 S S S S
ERR2199984 ERR2199985 S S S S
ERR552263 S S S S
ERR551784 S S S S
ERR553088 S S S S
ERR552923 R S S S
ERR552880 S S S S
ERR553150 S S S S
ERR551674 ERR551675 S S S S
ERR552264 S S S S
ERR553120 R S S S
ERR2199986 S S S S
ERR552055 S S S S
ERR552988 S S S S
ERR551275 S S S S
ERR551886 S S S S
ERR552543 S S S S
ERR550927 S S S S
ERR552382 S S S S
ERR552703 S S S S
ERR550839 S S S S
ERR553077 S S S S

ERR553227	S	S	S	S
ERR2199987	S	S	S	S
ERR551661	S	S	S	S
ERR553203	R	S	S	S
ERR552146	S	S	S	S
ERR552128	S	S	S	S
ERR551286	S	S	S	S
ERR553003	S	S	S	S
ERR553033	S	S	S	S
ERR553310	S	S	S	S
ERR551774	S	S	S	S
ERR553005	S	S	S	S
ERR2199989	S	S	S	U
ERR552394	S	S	S	S
ERR2199990	S	S	S	U
ERR2199991	S	S	S	U
ERR551614	S	S	S	S
ERR2199992	S	S	S	U
ERR2199993	S	S	S	U
ERR2199995	S	S	S	U
ERR2199996	S	S	S	U
ERR2200003	S	S	S	S
ERR2199998	S	S	S	U
ERR2199999	S	S	S	U
ERR2200000	S	S	S	U
ERR2200001	S	S	S	U
ERR2200002	S	S	S	U
ERR551545	R	R	R	R
ERR2200004	S	S	S	U
ERR2200005	S	S	S	U
ERR550867 ERR550868	S	S	S	U
ERR2200006	S	S	S	U
ERR2200007	S	S	S	U
ERR2200008	S	S	S	U
ERR2200009	S	S	S	U
ERR550632	S	S	S	S
ERR2200010	S	S	S	U
ERR2200011 ERR552806	R	S	S	U
ERR552316	S	S	S	S
ERR2200012	S	S	S	S
ERR552278	S	S	S	S
ERR552547	S	S	S	S
ERR551395	S	S	S	S
ERR552862	S	S	S	S
ERR2200013	S	S	S	U
ERR2200014	S	S	S	U
ERR551679	S	S	S	S
ERS457742	S	S	S	S
ERR552512	S	S	S	S
ERR2200015	S	S	S	U
ERR551160	S	S	S	S
ERR551120	S	S	S	S
ERS457645	S	S	S	S

ERR2200016 S S S S
ERR2200017 S S S U
ERR2200018 S S S U
ERR2200019 S S S U
ERR2200020 S S S U
ERR2200021 S S S U
ERR2200022 ERR553221 R R R R
ERR2200023 S S S U
ERR551382 S S S S
ERR552381 S S S S
ERR550629 S S S S
ERR2200024 S S S U
ERR2200025 S S S U
ERR552857 S S S S
ERR2200026 ERR2200027 S S S S
ERR553283 S S S S
ERR552721 S S S S
ERR550661 S S S S
ERR552749 S S S S
ERR551607 S S S S
ERR552235 S S S S
ERR552912 S S S S
ERR551459 S S S S
ERR553069 S S S S
ERR2200028 S S S S
ERR551497 S S S S
ERR551718 S S S S
ERR551026 S S S S
ERR551606 S S S S
ERR551776,ERR551775,ERR551777 R R R R
ERR551646 S S S S
ERR553089 S S S S
ERS456906 S S S S
ERS458757 S S S S
ERR2200029 S S S S
ERR552460 S S S S
ERR552096 S S S S
ERR553155 S S S S
ERR550718 R R S S
ERR551125 S S S S
ERR551183 S S S S
ERR552624 S S S S
ERR2200030 ERR2200031 ERR551204 R S S S
ERR2200032 S S S U
ERR2200033 S S S U
ERR2200034 S S S U
ERR2200035 S S S U
ERR2200036 S S S S
ERR2200037 S S S U
ERR2200038 S S S U
ERR551899 R R R R
ERR553294 S S S S
ERR553156 ERR553157 S S S U

ERR2200039 S S S U
ERR551828,ERR551829 R R S S
ERR551750,ERR551749,ERR551748 R R S S
ERR552764 ERR552765 R R R R
ERR2200040 S S S U
ERR551670 R S S S
ERR553039 S S S S
ERR552020 R S S S
ERR552717 S S S S
ERR553090 S S S S
ERR551573 S S S S
ERR553254 S S S S
ERR550795 S S S S
ERR552025 S S S S
ERR551752 S S S S
ERS458414 S R S S
ERS458258 S S S S
ERR552645,ERR552646 R R S R
ERR550978 R S S S
ERR553373 S S S S
ERR2200041 S S S S
ERR2200042 S S S S
ERR2200043 S S S U
ERR550711 S S S S
ERR2200044 S S S U
ERR552688 S S S S
ERR2200045 S S S U
ERR551086 S S S S
ERR2200048 ERR552420 R S S R
ERR552914 R S S S
ERR2200049 S S S U
ERR552484 R R R R
ERR2200050 S S S S
ERR2200051 S S S U
ERR552057 S S S S
ERR2200052 S S S U
ERR2200053 S S S S
ERR2200054 S S S S
ERR550739 S S S S
ERR550774 S S S S
ERR552018 S S S S
ERR2200055 S S S S
ERR552461 S S S S
ERR551481 S S S S
ERR551672 S S S S
ERS457344 S S S S
ERS457104 S S S S
ERR550944 S S S S
ERR2200056 S S S S
ERR2200057 S S S S
ERR552359 S S S S
ERR551274 S S S S
ERR552788 R S S S

ERR552154 S S S S
ERR553256 S S S S
ERR551393 R R R U
ERR2200058 ERR552487 R R R R
ERR553078 S S S S
ERR553223 S S S S
ERR551396 S S S S
ERR551098 S S S S
ERR551487 S S S S
ERR2200059 S S S S
ERR2200060 ERR2200061 ERR553328 R R R R
ERR551712 S S S S
ERR553266 S S S S
ERR551813 R S S S
ERR2200062 S S S S
ERR2200063 S S S S
ERR551720 S S S S
ERR2200064 ERR553204 R R R R
ERR551272 S S S S
ERR2200065 S S S U
ERR2200066 ERR551621 R R S R
ERR552544 S S S S
ERR2200067 S S S U
ERR2200068 ERR2200069 S S S U
ERR551322 S S S S
ERR2041718 R R R S
ERR2041719 R R S S
ERR2041720 R R R R
ERR2041721 R R R R
ERR2041722 R R S S
ERR551096 S S S S
ERR2041723 R R R S
ERR2041724 R R S S
ERR2041727 R R R R
ERR2041728 R R S R
ERR2041729 R R S S
ERR2041730 R R R S
ERR2041731 R R R S
ERR2041732 R R S R
ERR551157 S S S S
ERR2041733 R R R S
ERR2041734 R R R R
ERR2041735 R R R S
ERR2041736 R R R S
ERR2041737 ERR2041738 R R R R
ERR2041739 R R R S
ERR2200070 R R R R
ERR552587 S S S S
ERR550696 S S S S
ERR2200071 R S S S
ERR2200072 S S S R
ERR2200073 S S S S
ERR552850 S S S S

ERR553392 S S S S
ERR551754 S S S S
ERR551959,ERR551960 R R S S
ERR551948,ERR551949 R R R S
ERR552704 S S S S
ERR2200074 S S S U
ERR552294 S S S S
ERR552730 S S S S
ERR2200076 S S S U
ERR551579 S S S S
ERR2200077 S S S U
ERR551937 ERR551938 R R R R
ERR2200078 S S S U
ERR2200079 S S S U
ERR552743 S S S U
ERR552071 R S S S
ERR552993 ERR552994 R R R R
ERR2200080 S S S U
ERR553216 R R R R
ERR551390 S S S S
ERR551901 S S S S
ERR551655 S S S S
ERR552980 S S S S
ERR551495,ERR551496 R R R R
ERR552042,ERR552041 R R R S
ERR553051,ERR553050 R R R R
ERR552791,ERR552790 R R R R
ERR551121 R R R R
ERR2200081 S S S S
ERR2200082 S S S S
ERR552300 S S S S
ERR551084 S S S S
ERR551303 S S S S
ERR552043 S S S S
ERR2200083 ERR2200084 S S S S
ERR552737 S S S S
ERR551883 S S S S
ERR551574 S S S S
ERS457854 S S S S
ERS457331 S S S S
ERR553362 S S S S
ERR551186 S S S S
ERR552858 R S S S
ERR551541 S S S S
ERR550825 S S S S
ERS456952 S S S S
ERR552060 S S S S
ERR551346 S S S S
ERR551753 S S S S
ERR550892,ERR550893 R R R S
ERR552611,ERR552610 R R S S
ERR2200085 S S S S
ERR550730 S S S S

ERR552490 S S S S
ERR552375 S S S S
ERR551356 S S S S
ERR552577 S S S S
ERR552812 S S S S
ERR553023,ERR553022 R R S S
ERR550633 S S S S
ERR551539,ERR551540 R R R R
ERR551719 S S S S
ERR553036 S S S S
ERR552345 S S S S
ERR498363 ERR498364 ERR498365 S S S S
ERS457810 S S S S
ERS458448 R R R S
ERS458196 S S S S
ERR2200087 S S S U
ERR552073 R R R R
ERR2200088 S S S U
ERR552276 S S S S
ERR552597 S S S S
ERR551012 S S S S
ERR550767 S S S S
ERR550685,ERR550684 R R R S
ERR550848,ERR550849 R R R R
ERR551715 S S S S
ERR551460,ERR551461 R R R R
ERR552458,ERR552457 R R R R
ERR550914,ERR550915 R R R S
ERR552510,ERR552509 R R R S
ERR551478,ERR551477 R R U R
ERR552248 S S S S
ERR552724 S S S S
ERR2200089 S S S S
ERR550890,ERR550891 R R R S
ERR552047 R R R R
ERR2200090 S S S S
ERR551802 S S S S
ERR552367 S S S S
ERR498376,ERR498375 S S S S
ERR550806,ERR550807 R R R R
ERS456981 S S S S
ERR552080,ERR552079 R R S S
ERS456816 S S S S
ERR552410,ERR552409 R R S S
ERR551159 R R S R
ERR551684 S S S S
ERR550828 S S S S
ERR550802 S S S S
ERR551874 S S S S
ERR550983 S S S S
ERR551114 S S S S
ERR552076 S S S S
ERR551064 S S S S

ERR551345 S S S S
ERR552091 S S S S
ERR550818 S S S S
ERR552568 S S S S
ERR553068 R R R R
ERR552559,ERR552561,ERR552560 R R S R
ERR551957,ERR551956 R R R R
ERR552940,ERR552939 R R S R
ERR2200091 S S S S
ERR551894,ERR551895 R R S R
ERR552065,ERR552064 R R R S
ERR2041740 ERR2041741 R R S S
ERR2041742 R R S R
ERR550928,ERR550929 R R S R
ERR552926,ERR552927 R R R R
ERR2041743 R R R S
ERR2041744 R R S R
ERR2041745 R R S S
ERR2041746 R R R R
ERR2041747 R R R S
ERR2041748 R R R R
ERR2041749 R R R R
ERR2041750 R R R R
ERR2041751 R R S S
ERS457689 S S S S
ERR2041752 R R R S
ERR2041753 R R R S
ERR2041754 R R R R
ERR550880 S S S S
ERR551586 S S S S
ERR2041755 R R R S
ERR2200092 R R R S
ERS457924 S S S S
ERR2041756 R R R S
ERR2041757 R R R R
ERR2041758 R R S S
ERR552653,ERR552652 R R R R
ERR552693 S S S S
ERR2041759 R R R R
ERR2200093 R R R S
ERR2041760 R R R R
ERR2041761 R R R S
ERR2041762 R R R R
ERR2041763 R R R R
ERR2041764 R R R R
ERR2041765 R R S R
ERR2041766 R R R S
ERR551827,ERR551826 R R S R
ERR2041767 R R S S
ERR2041768 R R R S
ERR2041769 R R R S
ERR2041770 R R S S
ERR552519,ERR552520 R R R R

ERR551964 S S S S
ERR552924 S S S S
ERR552504,ERR552503 R R R R
ERR552687 S S S S
ERR2200096 S S S U
ERR551757 ERR551758 R R R R
ERR551000,ERR550999 R R R R
ERR551397 S S S S
ERR553019 S S S S
ERR552572 ERR552573 R R R R
ERR550821 S S S S
ERR553180,ERR553181 R R S S
ERR552293 S S S S
ERR2200097 S S S S
ERR552274 R R R S
ERR550960,ERR550961 R R R S
ERR551947,ERR551946 R R R S
ERR552681,ERR552682 R R S S
ERR552966 S S S S
ERS458484 S S S S
ERR551251,ERR551250 R R S S
ERR551816,ERR551815 R R S S
ERR552834,ERR552833 R R R R
ERR552628 S S S S
ERR552014 S S S S
ERR552953 S S S S
ERR551455 S S S S
ERR551596,ERR551595 R R S S
ERR553038 S S S S
ERR551723 S S S S
ERR552028 S S S S
ERR551616 S S S S
ERR551771,ERR551770 R R S S
ERR551841 S S S S
ERR2200098 ERR2200099 S S S S
ERR2200100 S S S S
ERR551279 S S S S
ERR552868 S S S S
ERR552428 S S S S
ERR551504 S S S S
ERR2200101 S S S S
ERR551822,ERR551821 R R R S
ERR552781 S S S S
ERR2200102 S S S S
ERR552418,ERR552417 R R R S
ERR550620 S S S S
ERR552819,ERR552818 R R S R
ERR552770 S S S S
ERS458489 R R R S
ERR550950,ERR550949 R R R S
ERR551259,ERR551258 R R R S
ERR551526 S S S S
ERR553264 R R R U

ERR2200103 S S S U
ERR553342 ERR553343 R R R U
ERR552612 R R R R
ERR552361 R R R U
ERR552495 R R R R
ERR551245 S S S S
ERR553358 R R R R
ERR552124 R R R R
ERR553182 R S S R
ERR551979 ERR551980 ERR551981 ERR551982 S S S U
ERR551578 R R S S
ERR553171 S S S S
ERR553066 S S S S
ERR2200104 S S S S
ERR553049 S S S S
ERR551340 S S S S
ERR550618 S S S S
ERR551103 S S S S
ERR551898 S S S S
ERR551931 S S S S
ERR552656 S S S S
ERR553276 S S S S
ERR552508 S S S S
ERR552705 S S S S
ERR551143 S S S S
ERR550768 S S S S
ERR552513,ERR552514 R R R R
ERR553262 S S S S
ERR553381 S S S S
ERR551442 S S S S
ERR551522 S S S S
ERR550856 S S S S
ERR2041771 R R S S
ERR2041772 R R S R
ERR2041773 R R S S
ERR2041774 R R R R
ERR2041775 R R R R
ERR2041776 R R S S
ERR2041777 R R R R
ERR2041780 R R R R
ERR2041781 R R R S
ERR2041782 R R R R
ERR2041783 R R R R
ERR2041784 R R R R
ERR2041785 R R R R
ERR2041786 R R R R
ERR2041787 R R R R
ERR2041788 R R S R
ERR551300 S S S S
ERR552373,ERR552374 R R S R
ERR552299 S S S S
ERR550805 S S S S
ERR550759 S S S S

ERR551518 S S S S
ERR550676,ERR550675 R R R S
ERR551099,ERR551100 R R S S
ERR552805 S S S S
ERS457155 S S S S
ERS458034 S S S S
ERS457942 S S S S
ERR552663 S S S S
ERR553149 S S S S
ERR550951 S S S S
ERR552166 S S S S
ERR552218 S S S S
ERR552350 S S S S
ERR551291 S S S S
SAMN05276441 S S S S
SAMN05276333 S S S S
SAMN05276332 S S S S
SAMN05276370 S S S S
SAMN05276411 S S S S
ERR551213 S S S S
SAMN05276430 S S S S
SAMN05276500 S S S S
SAMN05276409 S S S S
SAMN05276346 S S S S
SAMN05276382 R R R S
SAMN05276344 S S S S
SAMN05276559 S S S S
SAMN05276389 S S S S
SAMN05276503 S S S S
SAMN05276366 S S S S
SAMN05276508 S S S S
SAMN05276356 R R R S
SAMN05276542 S S S S
SAMN05276336 S S S S
SAMN05276493 R R R S
SAMN05276338 S S S S
SAMN05276502 S S S S
SAMN05276460 S S S S
SAMN05276399 S S S S
SAMN05276501 S S S S
SAMN05276319 S S S S
SAMN05276438 S S S S
SAMN05276349 S S S S
SAMN05276367 S S S S
SAMN05276379 S S S S
SAMN05276355 S S S S
ERR550695 S S S S
ERR551936 S S S S
ERR550731 S S S S
ERR498353 ERR498354 ERR498355 S S S S
ERR550733,ERR550732 R R S S
ERR551417,ERR551418 R R R R
ERR2200105 S S S U

ERR552009 S S S S
ERR552745 R R R R
ERR551834 S S S S
ERR552149 ERR552150 R R R R
ERR552151 R R S R
ERR552780 R R R U
ERR551689 R S S S
ERR551650 R R R S
ERR553185 ERR553186 R R R U
ERR2200106 S S S S
ERR551940 S S S S
ERR552100 S S S S
ERR552944 R S S S
ERR551696 S S S S
ERR550776 S S S S
ERR551260 S S S S
ERR550621 S S S S
ERR552969 S S S S
ERR552443,ERR552442 R R S S
ERR552600 S S S S
ERR551094 S S S S
ERR2200107 S S S S
ERR551806,ERR551805,ERR551804 R R R R
ERR551510,ERR551509 R R R U
ERR552734,ERR552733 R R R S
ERR550897,ERR550896 R R S S
ERR552354 S S S S
ERR553394,ERR553393 R R R R
ERR551824 S S S S
ERR551132,ERR551133 R R R R
ERR2200108 S S S S
ERR551624,ERR551623 R R R S
ERR552839 S S S S
ERR550624 S S S S
ERR552209 S S S S
ERR550737 S S S S
ERR551747 S S S S
ERR551158 S S S S
ERR2200109 S S S S
ERR552291 S S S S
ERR550862 S S S S
ERR550845 S S S S
ERR553265 S S S S
ERR552615 S S S S
ERR498293 ERR498294 ERR498295 S S S S
ERS458727 R S S S
ERR552286,ERR552287 R R R S
ERR551962 R S S S
ERR551985,ERR551984 R R S S
ERR552642 S S S S
ERR1768638 R R R R
ERR2200110 S S S S
ERR552538 S S S S

ERR553061 S S S R
ERR552771,ERR552772 R R R U
ERR552603,ERR552604 R R S R
ERR551888,ERR551887 R R R R
ERR551918,ERR551919 R R R R
ERR551600,ERR551599 R R R S
ERR552433,ERR552434 R R S S
ERR552719,ERR552718 R R R S
ERR551667,ERR551668 R R S S
ERR551643,ERR551642 R R S S
ERR551731,ERR551730 R R S S
ERR552677 R R S S
ERR552148,ERR552147 R R R R
ERR551844,ERR551845 R R R S
ERR550656 S S S S
ERR550692 S S S S
ERR551546 S S S S
ERR551007 S S S S
ERR2200111 S S S S
ERR552107 S S S S
ERR2200112 S S S S
ERR551326 S S S S
ERR551152 S S S S
ERR551576 S S S S
ERR552324 S S S S
ERR895346 S S S S
ERR550995 S S S S
ERR551372,ERR551373 R R R S
ERR550688 S S S S
ERR550813 R R R U
ERR553134 R R R R
ERR553116 S R S S
ERR553143 S S S S
ERR552336 S S S S
ERR551999 S S S S
ERR2200113 S S S S
ERR2200114 S S S S
ERR551115 S S S S
ERR552989 ERR552990 ERR552991 ERR552992 R R S S
ERR2200115 S S S U
ERR551240 ERR551241 R R R U
ERR553232 R S S S
ERR551564 S S S S
ERR553299 S S S S
ERR498383 ERR498384 S S S S
ERR552699 S S S S
ERR552661,ERR552660 R R R R
ERR551191 S S S R
ERR552342,ERR552341 R R S S
ERR552793,ERR552794 R R R R
ERR551321,ERR551320 R R R R
ERR550743,ERR550744 R R R R
ERR550667,ERR550669,ERR550668 R R R R

ERR551211,ERR551210 R R R R
ERR2200116 S S S S
ERR550778,ERR550777,ERR550779 R R R R
ERR550989,ERR550988,ERR550990 R R R R
ERR552531,ERR552532,ERR552530 R R R R
ERR551971 S S S S
ERR550635 S S S S
ERR552946 S S S S
ERR550853 R S S S
ERR553175 S S S S
ERR550817 S S S S
ERR551312 S S S S
ERR551119 S S S S
ERR553282 R R R R
ERR2200117 S S S U
ERR552463 R R S R
ERR552789 R R S R
ERR550708 S S S S
ERR2200118 S S S U
ERR551873 S S S S
ERR552181 ERR552182 R R R U
ERR2200119 S S S U
ERR551871 S S S S
ERR552851 S S S S
ERR551798,ERR551797 R R S S
ERR550748,ERR550749 R R S R
ERR550841,ERR550842 R R R S
ERR551437,ERR551436 R R S S
ERR551377,ERR551379,ERR551378 R R S S
ERR550649,ERR550648 R R R S
ERR553192,ERR553191 R R S S
ERR551532 S S S S
ERR551648,ERR551649 R R S S
ERR552631,ERR552632 R R R S
ERR553086 S S S S
ERR552033,ERR552032 R R S S
ERR553184,ERR553183 R R R R
ERR551996,ERR551994,ERR551995 R R R R
ERR550706,ERR550707 R R R S
ERR551431,ERR551432 R R S S
ERR551108,ERR551109 R R S S
ERR551745,ERR551744,ERR551743 R R S S
ERR552068,ERR552067 R R S S
ERR552190 S S S S
ERR551385,ERR551384 R R S S
ERR551020,ERR551019 R R R S
ERR551464,ERR551465 R R S R
ERR552842,ERR552841 R R R S
ERR550770,ERR550771 R R S R
ERR552595 S S S S
ERR551198 S S S S
ERR2200120 S S S S
ERR2200121 S S S S

ERR551061 S S S S
ERR2200122 ERR551111 ERR551112 S S S S
ERR2200123 S S S S
ERR551009 S S S R
ERR551058 S S S S
ERR551796 S S S S
ERR552153 S S S S
ERR552916 ERR552917 R R R U
ERR552070 R R R R
ERR553020 ERR553021 R R R R
ERR552809 R R R R
ERR550657 R S S S
ERR2200124 S S S S
ERR550943 S S S S
ERR552156 S S S S
ERR553327 S S S S
ERR553214 ERR553215 S S S S
ERS458461 R R R S
ERS457115 R S S S
ERS458043 R S S R
ERR551708,ERR551707 R R R S
ERR551128,ERR551129 R R R S
ERR2200125 S S S S
ERR551052,ERR551051 R R R S
ERR551264 ERR551265 S S S S
ERR552750 S S S S
ERR552674 S S S S
ERR552229 S S S S
ERR551165 S S S S
ERR553167 S S S S
ERR553034 S S S S
ERR550801 S S S S
ERR551860 R S S S
ERR551348 S S S S
ERR553309 S S S S
ERR552826 S S S S
ERR552072 S S S S
ERR551315 ERR551316 S S S S
ERR550672 S S S S
ERR552999 S S S S
ERR552401,ERR552400 R R R R
ERR552358 R R R S
ERR2200126 S S S S
ERR550664,ERR550663 R R R R
ERR551945,ERR551943,ERR551944 R R R R
ERR550682,ERR550681,ERR550683 R R R R
ERR552738,ERR552739 R R S S
ERR2200127 S S S S
ERR551352,ERR551353 R R R R
ERR550722 S S S S
ERR552258,ERR552257 R R R R
ERR553198,ERR553197 R R R S
ERR551536 R R R R

ERR552986 ERR552987 R R R R
ERR551298 ERR551299 R R S S
ERR550763,ERR550762 R R R S
ERR551447 S S S S
ERR553135 S S S S
ERR2200128 ERR2200129 S S S S
ERR2200130 S S S S
ERR552644 S S S S
ERR552756 S S S S
ERR551366 R R S S
ERR552649 S S S S
ERR553267 S S S S
ERR551762 S S S S
ERR2200131 S S S S
ERR552713 S S S S
ERR552539 R S S S
ERR552180 S S S S
ERR551864 S S S S
ERR553259 S S S S
ERR552744 S S S S
ERR553390 S S S S
ERR550673 S S S S
ERR551503 S S S S
ERR552716 S S S S
ERR551658 S S S S
ERR550810 S S S S
ERR552762 S S S S
ERR550844 S S S S
ERR550662 S S S S
ERR2200132 S S S S
ERR550965 S S S S
ERR2200133 S S S S
ERR552837 S S S S
ERR550991 S S S S
ERR552541 S S S S
ERR551482 S S S S
ERR553091 S S S S
ERR551818 S S S S
ERR552205 S S S S
ERR551297 S S S S
ERR551865 ERR551866 S S S S
ERR551565 S S S S
ERR552803 S S S S
ERR551872 S S S S
ERR552476 S S S S
ERR550639 ERR550640 S S S S
ERR2200134 S S S S
ERR553102 S S S S
ERR552298,ERR552297 R R S S
ERR552741,ERR552740 R R R S
ERR552112,ERR552111 R R R S
ERR551992 S S S S
ERR552390,ERR552389 R R R R

ERR552955,ERR552956 R R R R
ERR2200135 ERR2200136 S S S S
ERR550863 ERR550864 R R R R
ERR2200137 ERR2200138 R R R R
ERR553001 ERR553002 R R R R
ERR2200139 S S S U
ERR553311 S S S S
ERR550910 S S S S
ERR552203 S S S S
ERR552524 S S S S
ERR552995 S S S S
ERR2200140 S S S S
ERR551042 S S S S
ERR553193 S S S S
ERR552388 S S S S
ERR552270 S S S S
ERR551220,ERR551221 R R R S
ERR553246,ERR553247 R R S S
ERR551142,ERR551141 R R S R
ERR551592,ERR551591 R R R R
ERR551515,ERR551514,ERR551516 R R S S
ERR552580 R R S S
ERR550809,ERR550808 R R R S
ERR551634,ERR551635 R R S S
ERR550956 S S S S
ERR552810 S S S S
ERR551559 S S S S
ERR2200141 S S S S
ERR551127 S S S S
ERR2200142 ERR2200143 S S S S
ERR550717 S S S S
ERR2200144 S S S S
ERR552860 S S S S
ERR2516559 R R R U
ERR2516478 R R R R
ERR2516482 R R R R
ERR551603,ERR551604 R R R S
ERR551365 S S S S
ERR552102,ERR552101 R R R S
ERR551293 R S S S
ERR553308 S S S S
ERR2200145 S S S S
ERR551889 S S S S
ERR2200146 ERR2200147 S S S S
ERR553113 R S S S
ERR552584 S S S S
ERR553339 S S S S
ERR551954 S S S S
ERR551224 S S S S
ERR2516527 R R R R
ERR2516483 R U R U
ERR2516614 R R R S
ERR2516713 R R R U

ERR2516499	R	R	R	R
ERR2516543	R	R	R	R
ERR2516590	R	R	R	S
ERR2516727	R	R	R	R
ERR2516460	R	R	R	S
ERR2516696	R	R	R	R
ERR2516663	R	R	R	U
ERR553388	S	S	S	S
ERR553249	S	S	S	S
ERR2041789	R	R	R	R
ERR2041790 ERR2041791	R	R	R	R
ERR2041792 ERR2041793	R	R	S	S
ERR2041794	R	R	S	S
ERR2041795	R	R	R	S
ERR2041796	R	R	R	R
ERR2041797 ERR2041798	R	R	R	S
ERR2041799 ERR2041800	R	R	S	R
ERR2041801 ERR2041802	R	R	R	R
ERR2200148	R	R	S	R
ERR2041803	R	R	R	S
ERR2041804	R	R	S	S
ERR2041805	R	R	S	S
ERR2200149	S	S	S	S
ERR552202	S	S	S	S
ERR552110	S	S	S	S
ERR551290	R	R	R	R
ERR552419	S	S	S	S
ERR552441	R	R	R	R
ERR550812	R	R	R	R
ERR551701	R	R	R	R
ERR552933	R	R	R	R
ERR552937	R	R	R	S
ERR552517	R	R	R	R
ERR551611,ERR551610	R	R	R	R
ERR552774	R	R	S	R
ERR553333	S	S	S	S
ERR2200150 ERR2200151	S	S	S	S
ERR551145	R	R	R	R
ERR551430	R	R	R	R
ERR552483	S	S	S	S
PRJNA413593	S	S	S	U
PRJNA413593	S	S	S	U
PRJNA413593	S	S	S	U
PRJNA413593	S	S	S	U
PRJNA413593	R	R	S	U
PRJNA413593	S	S	S	U
PRJNA413593	S	S	S	U
PRJNA413593	S	S	S	U
PRJNA413593	S	S	S	U
PRJNA413593	S	S	S	U
PRJNA413593	S	S	S	U
PRJNA413593	S	S	S	U
PRJNA413593	S	S	S	U

PRJNA413593	S	S	S	U
PRJNA413593	S	S	S	U
PRJNA413593	R	S	S	U
PRJNA413593	S	S	S	U
PRJNA413593	S	S	S	U
PRJNA413593	R	S	S	U
PRJNA413593	S	S	S	U
PRJNA413593	R	S	S	U
PRJNA413593	S	S	S	U
PRJNA413593	S	S	S	U
PRJNA413593	S	S	S	U
PRJNA413593	S	S	S	U
PRJNA413593	S	S	S	U
PRJNA413593	S	S	S	U
PRJNA413593	R	R	R	R
PRJNA413593	S	S	S	U
PRJNA413593	S	S	S	U
PRJNA413593	S	S	S	U
PRJNA413593	S	S	S	U
PRJNA413593	S	S	U	U
PRJNA413593	S	S	S	U
PRJNA413593	S	S	S	U
PRJNA413593	U	S	S	S
PRJNA413593	S	S	S	U
PRJNA413593	S	S	S	U
PRJNA413593	S	S	S	U
PRJNA413593	S	S	S	U
PRJNA413593	S	S	S	U
PRJNA413593	S	S	S	U
PRJNA413593	S	S	S	U
PRJNA413593	S	S	S	U
PRJNA413593	S	S	S	U
PRJNA413593	S	S	S	U
PRJNA413593	S	S	S	U
PRJNA413593	S	S	S	U
PRJNA413593	S	S	S	U
PRJNA413593	S	S	S	U
PRJNA413593	S	S	S	U
PRJNA413593	S	S	S	U
PRJNA413593	S	S	S	U
PRJNA413593	S	S	S	U
PRJNA413593	S	S	S	U
PRJNA413593	S	S	S	U
PRJNA413593	S	S	S	U
PRJNA413593	S	S	S	U
PRJNA413593	S	S	S	U
PRJNA413593	S	S	S	U
PRJNA413593	S	S	S	S
PRJNA413593	R	S	S	S
PRJNA413593	S	S	S	U
PRJNA413593	S	S	S	U
PRJNA413593	R	R	R	R
PRJNA413593	S	S	R	U

PRJNA413593	S	S	S	U
PRJNA413593	S	S	S	U
PRJNA413593	R	S	S	S
PRJNA413593	R	S	S	S
PRJNA413593	S	S	S	U
PRJNA413593	R	S	S	S
PRJNA413593	S	S	S	U
PRJNA413593	S	S	S	U
PRJNA413593	S	S	S	U
PRJNA413593	S	S	S	U
PRJNA413593	S	S	S	U
PRJNA413593	S	S	S	U
PRJNA413593	S	S	S	U
PRJNA413593	S	S	S	U
PRJNA413593	S	S	S	U
PRJNA413593	S	S	S	U
PRJNA413593	R	S	S	S
PRJNA413593	S	S	S	U
PRJNA413593	S	S	S	U
PRJNA413593	S	S	S	U
PRJNA413593	S	S	S	U
PRJNA413593	S	S	S	U
PRJNA413593	S	S	S	U
PRJNA413593	S	S	S	U
PRJNA413593	S	S	S	U
PRJNA413593	S	S	S	U
PRJNA413593	S	S	S	U
PRJNA413593	S	S	S	U
PRJNA413593	S	S	S	U
PRJNA413593	S	S	S	U
PRJNA413593	S	S	S	U
PRJNA413593	S	S	S	U
PRJNA413593	S	S	S	U
PRJNA413593	S	S	S	U
PRJNA413593	S	S	S	U
PRJNA413593	R	R	S	R
PRJNA413593	S	S	S	U
PRJNA413593	R	S	S	S
PRJNA413593	S	S	S	U
PRJNA413593	S	S	S	U
PRJNA413593	S	S	S	U
PRJNA413593	S	S	S	U
PRJNA413593	S	S	S	U
PRJNA413593	S	S	S	U
PRJNA413593	S	S	S	U
PRJNA413593	S	S	S	U
PRJNA413593	S	S	S	U
PRJNA413593	S	S	S	U
PRJNA413593	R	S	S	S
PRJNA413593	S	S	S	U
PRJNA413593	R	S	S	S
PRJNA413593	S	S	R	U
PRJNA413593	R	S	R	S

PRJNA413593	S	S	S	U
PRJNA413593	S	S	S	U
PRJNA413593	S	S	S	U
PRJNA413593	S	S	S	U
PRJNA413593	R	S	S	S
PRJNA413593	S	S	S	U
PRJNA413593	R	S	S	S
PRJNA413593	R	R	R	S
PRJNA413593	S	S	S	U
PRJNA413593	S	S	S	U
PRJNA413593	S	S	S	U
PRJNA413593	S	S	S	U
PRJNA413593	S	S	S	U
PRJNA413593	S	S	S	U
PRJNA413593	S	S	S	U
PRJNA413593	S	S	S	U
PRJNA413593	S	S	S	U
PRJNA413593	S	S	S	U
PRJNA413593	S	S	S	U
PRJNA413593	S	S	S	U
PRJNA413593	S	S	S	U
PRJNA413593	S	S	S	U
PRJNA413593	S	S	S	U
PRJNA413593	S	S	S	U
PRJNA413593	S	S	S	U
PRJNA413593	S	S	S	U
PRJNA413593	S	S	S	U
PRJNA413593	S	S	S	U
PRJNA413593	S	S	S	U
PRJNA413593	S	R	S	S
PRJNA413593	R	S	S	S
PRJNA413593	S	S	S	U
PRJNA413593	S	S	S	U
PRJNA413593	S	S	S	U
PRJNA413593	S	S	S	U
PRJNA413593	S	S	S	U
PRJNA413593	S	S	S	U
PRJNA413593	S	S	S	U
PRJNA413593	S	S	S	U
PRJNA413593	S	S	S	U
PRJNA413593	R	R	R	R
PRJNA413593	S	S	S	U
PRJNA413593	S	S	S	U
PRJNA413593	S	R	S	S
PRJNA413593	S	S	S	U
PRJNA413593	S	S	S	U
PRJNA413593	S	S	S	U
PRJNA413593	S	S	S	U
PRJNA413593	S	R	S	S
PRJNA413593	S	S	S	U
PRJNA413593	S	S	S	U
PRJNA413593	S	S	S	U
PRJNA413593	S	S	S	U

PRJNA413593	S	S	S	U
PRJNA413593	S	S	S	U
PRJNA413593	S	S	S	U
PRJNA413593	S	S	S	U
PRJNA413593	S	S	R	U
PRJNA413593	R	S	S	S
PRJNA413593	S	S	U	U
PRJNA413593	S	R	S	S
PRJNA413593	S	R	S	S
PRJNA413593	R	S	S	S
PRJNA413593	S	S	S	U
PRJNA413593	S	S	S	U
PRJNA413593	S	S	S	U
PRJNA413593	R	R	R	R
PRJNA413593	S	S	S	U
PRJNA413593	S	S	S	U
PRJNA413593	S	S	S	U
PRJNA413593	S	S	S	U
PRJNA413593	S	S	S	U
PRJNA413593	S	S	S	U
PRJNA413593	S	S	S	U
PRJNA413593	S	S	S	U
PRJNA413593	S	S	S	U
PRJNA413593	S	S	S	U
PRJNA413593	S	S	S	U
PRJNA413593	S	S	R	U
PRJNA413593	S	S	S	U
PRJNA413593	S	S	S	U
PRJNA413593	S	S	S	U
PRJNA413593	S	S	S	U
PRJNA413593	R	S	S	S
PRJNA413593	S	S	S	U
PRJNA413593	S	S	S	U
PRJNA413593	S	S	S	U
PRJNA413593	S	S	S	U
PRJNA413593	S	S	S	U
PRJNA413593	S	S	S	U
PRJNA413593	S	S	S	U
PRJNA413593	S	S	S	U
PRJNA413593	S	S	S	U
PRJNA413593	S	R	S	U
PRJNA413593	S	S	S	U
PRJNA413593	S	S	S	U
PRJNA413593	S	S	S	U
PRJNA413593	S	S	S	U
PRJNA413593	S	S	S	U
PRJNA413593	S	S	S	U
PRJNA413593	S	S	S	U
PRJNA413593	S	R	S	U
PRJNA413593	S	S	S	U
PRJNA413593	S	S	S	U
PRJNA413593	S	S	S	U
PRJNA413593	R	S	S	U

PRJNA413593	S	S	S	U
PRJNA413593	S	S	S	U
PRJNA413593	S	S	S	U
PRJNA413593	S	S	S	U
PRJNA413593	R	S	S	U
PRJNA413593	S	S	S	U
PRJNA413593	S	S	S	U
PRJNA413593	S	S	S	U
PRJNA413593	S	S	S	U
PRJNA413593	R	S	S	U
PRJNA413593	S	S	S	U
PRJNA413593	S	S	S	U
PRJNA413593	S	S	S	U
PRJNA413593	S	S	S	U
PRJNA413593	S	S	S	U
PRJNA413593	S	S	S	U
PRJNA413593	S	S	S	U
PRJNA413593	S	S	S	U
PRJNA413593	S	S	S	U
PRJNA413593	S	S	S	U
PRJNA413593	S	S	S	U
PRJNA413593	S	R	S	U
PRJNA413593	S	S	S	U
PRJNA413593	S	S	S	U
PRJNA413593	S	S	S	U
PRJNA413593	S	S	S	U
PRJNA413593	S	S	S	U
PRJNA413593	S	S	S	U
PRJNA413593	S	S	S	U
PRJNA413593	S	S	S	U
PRJNA413593	S	S	S	U
PRJNA413593	S	S	S	U
PRJNA413593	S	S	S	U
PRJNA413593	S	S	S	U
PRJNA413593	R	S	S	U
PRJNA413593	S	S	S	U
PRJNA413593	S	S	S	U
PRJNA413593	R	R	S	S
PRJNA413593	S	S	S	U
PRJNA413593	S	S	S	U
PRJNA413593	S	S	S	U
PRJNA413593	S	S	S	U
PRJNA413593	S	S	S	U
PRJNA413593	S	S	S	U
PRJNA413593	S	S	S	U
PRJNA413593	S	S	S	U
PRJNA413593	S	S	S	U
PRJNA413593	S	S	S	U
PRJNA413593	S	S	S	U
PRJNA413593	S	S	S	U
PRJNA413593	S	S	S	U
PRJNA413593	S	S	S	U
PRJNA413593	S	S	S	U

PRJNA413593	S	S	S	U
PRJNA413593	S	S	S	U
PRJNA413593	S	S	S	U
PRJNA413593	S	S	S	U
PRJNA413593	S	S	S	U
PRJNA413593	S	S	S	U
PRJNA413593	S	S	S	U
PRJNA413593	S	S	S	U
PRJNA413593	S	S	S	U
PRJNA413593	S	S	S	U
PRJNA413593	S	S	S	U
PRJNA413593	S	S	S	U
PRJNA413593	S	S	S	U
PRJNA413593	S	S	S	U
PRJNA413593	S	S	S	U
PRJNA413593	S	S	S	U
PRJNA413593	S	S	S	U
PRJNA413593	R	S	R	S
PRJNA413593	S	S	S	U
PRJNA413593	S	S	S	U
PRJNA413593	S	S	S	U
PRJNA413593	S	S	S	S
PRJNA413593	S	S	S	U
PRJNA413593	S	S	S	U
PRJNA413593	S	S	S	U
PRJNA413593	S	S	S	U
PRJNA413593	S	S	S	U
PRJNA413593	S	S	S	U
PRJNA413593	S	S	S	U
PRJNA413593	S	S	S	U
PRJNA413593	S	S	S	U
PRJNA413593	S	S	S	U
PRJNA413593	S	S	S	U
PRJNA413593	R	R	R	R
PRJNA413593	S	S	S	U
PRJNA413593	R	S	S	U
PRJNA413593	S	S	S	U
PRJNA413593	S	S	S	U
PRJNA413593	S	S	S	U
PRJNA413593	S	S	S	U
PRJNA413593	R	S	S	U
PRJNA413593	S	S	S	U
PRJNA413593	S	S	S	U
PRJNA413593	S	S	S	U
PRJNA413593	S	S	S	U
PRJNA413593	S	S	S	U
PRJNA413593	S	S	S	U
PRJNA413593	S	S	S	U
PRJNA413593	S	S	S	U
PRJNA413593	S	S	S	U
PRJNA413593	S	S	S	U
PRJNA413593	S	S	S	U
PRJNA413593	S	S	S	U

PRJNA413593	S	S	R	U
PRJNA413593	S	S	S	U
PRJNA413593	R	S	R	U
PRJNA413593	S	S	S	U
PRJNA413593	S	S	S	U
PRJNA413593	S	S	S	U
PRJNA413593	S	S	S	U
PRJNA413593	S	S	S	U
PRJNA413593	S	S	S	U
PRJNA413593	S	S	S	U
PRJNA413593	S	S	S	U
PRJNA413593	S	S	S	U
PRJNA413593	S	S	S	U
PRJNA413593	S	S	S	U
PRJNA413593	S	S	S	U
PRJNA413593	S	S	S	U
PRJNA413593	S	S	S	U
PRJNA413593	S	S	S	U
PRJNA413593	S	S	S	U
PRJNA413593	S	S	S	U
PRJNA413593	S	S	S	U
PRJNA413593	S	S	S	U
PRJNA413593	S	S	S	U
PRJNA413593	S	S	S	U
PRJNA413593	S	S	S	U
PRJNA413593	S	S	S	U
PRJNA413593	S	S	S	U
PRJNA413593	S	S	S	U
PRJNA413593	S	S	S	U
PRJNA413593	S	S	S	U
PRJNA413593	S	S	S	U
PRJNA413593	S	S	S	U
PRJNA413593	S	S	S	U
PRJNA413593	S	S	S	U
PRJNA413593	R	S	S	U
PRJNA413593	S	S	S	U
PRJNA413593	S	S	S	U
PRJNA413593	S	S	S	U
PRJNA413593	S	S	S	U
PRJNA413593	S	S	S	U
PRJNA413593	S	S	S	U
PRJNA413593	R	R	R	R
PRJNA413593	S	S	S	U
PRJNA413593	S	S	S	U
PRJNA413593	S	S	S	U
PRJNA413593	S	S	S	U
PRJNA413593	S	S	S	U
PRJNA413593	S	S	S	U
PRJNA413593	S	S	S	U
PRJNA413593	S	S	S	U
PRJNA413593	U	S	S	S
PRJNA413593	S	S	S	U
PRJNA413593	S	S	S	U
PRJNA413593	S	S	S	U

PRJNA413593	S	S	S	U
PRJNA413593	S	S	S	U
PRJNA413593	S	S	S	U
PRJNA413593	S	S	S	U
PRJNA413593	S	S	S	U
PRJNA413593	S	S	S	U
PRJNA413593	S	S	S	U
PRJNA413593	R	S	S	U
PRJNA413593	S	S	S	U
PRJNA413593	S	S	S	U
PRJNA413593	S	S	S	U
PRJNA413593	R	S	S	U
PRJNA413593	S	S	S	U
PRJNA413593	S	S	S	U
PRJNA413593	S	S	S	U
PRJNA413593	S	S	S	U
PRJNA413593	S	S	S	U
PRJNA413593	S	S	S	U
PRJNA413593	S	S	S	U
PRJNA413593	S	S	S	U
PRJNA413593	S	S	S	U
PRJNA413593	S	S	S	U
PRJNA413593	S	S	S	U
PRJNA413593	R	S	S	U
PRJNA413593	R	R	R	R
PRJNA413593	S	S	S	U
PRJNA413593	S	S	S	U
PRJNA413593	S	S	S	U
PRJNA413593	S	S	S	U
PRJNA413593	S	S	S	U
PRJNA413593	S	S	S	U
PRJNA413593	S	S	S	U
PRJNA413593	S	S	S	U
PRJNA413593	S	S	S	U
PRJNA413593	S	S	S	U
PRJNA413593	R	R	R	R
PRJNA413593	S	S	S	U
PRJNA413593	S	S	S	U
PRJNA413593	S	S	S	U
PRJNA413593	S	S	S	U
PRJNA413593	S	S	S	U
PRJNA413593	S	S	S	U
PRJNA413593	S	S	R	U
PRJNA413593	R	S	S	U
PRJNA413593	S	S	S	U
PRJNA413593	S	S	S	U
PRJNA413593	S	R	S	U
PRJNA413593	R	S	S	S
PRJNA413593	R	S	S	U
PRJNA413593	S	S	S	U
PRJNA413593	S	S	S	U
PRJNA413593	S	S	S	U
PRJNA413593	S	S	S	U

PRJNA413593	S	S	S	U
PRJNA413593	S	S	S	U
PRJNA413593	S	S	S	U
PRJNA413593	S	S	S	U
PRJNA413593	R	S	S	S
PRJNA413593	S	S	S	U
PRJNA413593	S	S	S	U
PRJNA413593	S	S	S	U
PRJNA413593	S	S	S	U
PRJNA413593	S	S	S	U
PRJNA413593	R	S	S	S
PRJNA413593	R	R	S	S
PRJNA413593	S	S	S	U
PRJNA413593	S	S	S	U
PRJNA413593	S	S	S	U
PRJNA413593	S	S	S	U
PRJNA413593	R	S	S	U
PRJNA413593	S	S	S	U
PRJNA413593	R	S	S	U
PRJNA413593	S	S	S	U
PRJNA413593	S	S	S	U
PRJNA413593	S	S	S	U
PRJNA413593	S	S	S	U
PRJNA413593	S	S	S	U
PRJNA413593	S	S	S	U
PRJNA413593	S	S	S	U
PRJNA413593	S	S	S	U
PRJNA413593	S	S	S	U
PRJNA413593	S	S	S	U
PRJNA413593	S	S	S	U
PRJNA413593	S	S	S	U
PRJNA413593	S	S	S	U
PRJNA413593	S	S	S	U
PRJNA413593	S	S	S	U
PRJNA413593	S	S	S	U
PRJNA413593	S	S	S	U
PRJNA413593	S	S	S	U
PRJNA413593	S	S	S	U
PRJNA413593	S	S	S	U
PRJNA413593	S	S	S	U
PRJNA413593	S	S	S	U
PRJNA413593	S	S	S	U
PRJNA413593	S	S	S	U
PRJNA413593	R	S	S	U
PRJNA413593	S	S	S	U
PRJNA413593	S	S	S	U
PRJNA413593	S	S	S	U
PRJNA413593	R	S	S	U
PRJNA413593	S	S	S	U
PRJNA413593	S	S	S	U
PRJNA413593	S	S	S	U
PRJNA413593	S	S	S	U
PRJNA413593	S	S	S	U

PRJNA413593	S	S	S	U
PRJNA413593	S	S	S	U
PRJNA413593	S	S	S	U
PRJNA413593	S	S	S	U
PRJNA413593	S	S	S	U
PRJNA413593	S	S	S	U
PRJNA413593	S	S	S	U
PRJNA413593	S	S	S	U
PRJNA413593	S	S	S	U
PRJNA413593	S	S	S	U
PRJNA413593	S	S	S	U
PRJNA413593	S	S	S	U
PRJNA413593	S	S	S	U
PRJNA413593	S	S	S	U
PRJNA413593	S	S	S	U
PRJNA413593	S	S	S	U
PRJNA413593	S	S	S	U
PRJNA413593	S	S	S	U
PRJNA413593	R	S	S	U
PRJNA413593	R	S	S	U
PRJNA413593	S	S	S	U
PRJNA413593	S	S	S	U
PRJNA413593	S	S	S	U
PRJNA413593	R	S	S	U
PRJNA413593	S	S	S	U
PRJNA413593	S	S	S	U
PRJNA413593	S	S	S	U
PRJNA413593	S	S	S	U
PRJNA413593	R	S	S	U
PRJNA413593	S	S	S	U
PRJNA413593	S	S	S	U
PRJNA413593	S	R	S	U
PRJNA413593	S	S	S	U
PRJNA413593	S	S	S	U
PRJNA413593	S	S	S	U
PRJNA413593	S	S	S	U
PRJNA413593	S	S	S	U
PRJNA413593	S	S	S	U
PRJNA413593	S	S	S	U
PRJNA413593	S	S	S	U
PRJNA413593	S	S	S	U
PRJNA413593	S	S	S	U
PRJNA413593	S	S	S	U
PRJNA413593	S	S	S	U
PRJNA413593	S	S	S	U
PRJNA413593	S	S	S	U
PRJNA413593	S	S	S	U
PRJNA413593	S	S	S	U
PRJNA413593	S	S	S	U
PRJNA413593	S	S	S	U
PRJNA413593	R	S	S	S
PRJNA413593	S	S	S	U
PRJNA413593	R	R	R	R

PRJNA413593	S	S	S	U
PRJNA413593	S	S	S	U
PRJNA413593	R	S	S	U
PRJNA413593	S	S	S	U
PRJNA413593	S	S	S	U
PRJNA413593	S	S	S	U
PRJNA413593	S	S	S	U
PRJNA413593	S	S	S	U
PRJNA413593	S	S	S	U
PRJNA413593	S	S	S	U
PRJNA413593	S	S	S	U
PRJNA413593	S	S	S	U
PRJNA413593	S	S	S	U
PRJNA413593	R	S	S	U
PRJNA413593	S	S	S	U
PRJNA413593	S	S	S	U
PRJNA413593	S	S	S	U
PRJNA413593	S	S	S	U
PRJNA413593	S	S	S	U
PRJNA413593	S	S	S	U
PRJNA413593	S	S	S	U
PRJNA413593	S	S	S	U
PRJNA413593	S	S	S	U
PRJNA413593	S	S	S	U
PRJNA413593	S	S	S	U
PRJNA413593	S	S	S	U
PRJNA413593	S	S	S	U
PRJNA413593	S	S	S	U
PRJNA413593	R	S	S	U
PRJNA413593	S	S	S	U
PRJNA413593	S	S	S	U
PRJNA413593	S	S	S	U
PRJNA413593	S	S	S	U
PRJNA413593	S	S	S	U
PRJNA413593	S	S	S	U
PRJNA413593	S	S	S	U
PRJNA413593	S	S	S	U
PRJNA413593	S	S	S	U
PRJNA413593	S	S	S	U
PRJNA413593	S	S	S	U
PRJNA413593	R	S	R	U
PRJNA413593	S	S	S	U
PRJNA413593	S	S	S	U
PRJNA413593	R	U	R	U
PRJNA413593	S	S	S	U
PRJNA413593	S	S	S	U
PRJNA413593	S	S	S	U
PRJNA413593	S	S	S	U
PRJNA413593	S	S	S	U
PRJNA413593	S	S	S	U
PRJNA413593	S	S	S	U
PRJNA413593	S	S	S	U
PRJNA413593	R	S	S	U

PRJNA413593	S	S	S	U
PRJNA413593	S	S	S	U
PRJNA413593	S	S	S	U
PRJNA413593	S	S	S	U
PRJNA413593	S	S	S	U
PRJNA413593	S	S	S	U
PRJNA413593	S	S	S	U
PRJNA413593	S	S	S	U
PRJNA413593	S	S	S	U
PRJNA413593	S	S	S	U
PRJNA413593	S	S	S	U
PRJNA413593	S	S	S	U
PRJNA413593	S	S	S	U
PRJNA413593	S	S	S	U
PRJNA413593	S	S	S	U
PRJNA413593	S	S	S	U
PRJNA413593	S	S	S	U
PRJNA413593	S	S	S	U
PRJNA413593	S	S	S	U
PRJNA413593	R	S	S	U
PRJNA413593	S	S	S	U
PRJNA413593	S	S	S	U
PRJNA413593	S	S	S	U
PRJNA413593	S	S	S	U
PRJNA413593	S	S	S	U
PRJNA413593	S	S	S	U
PRJNA413593	R	S	S	U
PRJNA413593	S	S	S	U
PRJNA413593	S	S	S	U
PRJNA413593	R	S	S	U
PRJNA413593	S	S	S	U
PRJNA413593	S	S	S	U
PRJNA413593	S	S	S	U
PRJNA413593	S	S	S	U
PRJNA413593	S	S	S	U
PRJNA413593	S	S	S	U
PRJNA413593	S	S	S	U
PRJNA413593	S	S	S	U
PRJNA413593	S	S	S	U
PRJNA413593	S	S	S	U
PRJNA413593	S	S	S	U
PRJNA413593	R	S	S	U
PRJNA413593	S	S	S	U
PRJNA413593	S	S	S	U
PRJNA413593	S	S	S	U
PRJNA413593	S	S	S	U
PRJNA413593	S	S	S	U
PRJNA413593	S	S	S	U
PRJNA413593	S	S	S	U
PRJNA413593	S	S	S	U
PRJNA413593	S	S	S	U
PRJNA413593	R	S	S	U
PRJNA413593	S	S	S	U

PRJNA413593	S	S	S	U
PRJNA413593	S	S	S	U
PRJNA413593	S	S	S	U
PRJNA413593	S	S	S	U
PRJNA413593	S	S	S	U
PRJNA413593	S	S	S	U
PRJNA413593	S	S	S	U
PRJNA413593	S	S	S	U
PRJNA413593	S	S	S	U
PRJNA413593	S	S	S	U
PRJNA413593	S	S	S	U
PRJNA413593	S	S	S	U
PRJNA413593	R	S	S	U
PRJNA413593	S	S	S	U
PRJNA413593	S	S	S	U
PRJNA413593	S	S	S	U
PRJNA413593	S	S	S	U
PRJNA413593	R	S	S	S
PRJNA413593	S	S	S	U
PRJNA413593	S	S	S	U
PRJNA413593	S	S	S	U
PRJNA413593	S	S	S	U
PRJNA413593	S	S	S	U
PRJNA413593	S	S	S	U
PRJNA413593	S	S	S	U
PRJNA413593	S	S	S	U
PRJNA413593	S	S	S	U
PRJNA413593	S	S	S	U
PRJNA413593	S	S	S	U
PRJNA413593	S	S	S	U
PRJNA413593	R	S	S	S
PRJNA413593	S	S	S	U
PRJNA413593	S	S	S	U
PRJNA413593	S	S	S	U
PRJNA413593	S	R	S	U
PRJNA413593	R	S	S	S
PRJNA413593	R	S	S	S
PRJNA413593	S	S	S	U
PRJNA413593	R	S	S	S
PRJNA413593	S	S	S	U
PRJNA413593	S	S	S	U
PRJNA413593	S	S	S	U
PRJNA413593	S	S	S	U
PRJNA413593	R	S	S	U
PRJNA413593	S	S	S	U
PRJNA413593	S	S	S	U
PRJNA413593	S	S	S	U
PRJNA413593	S	S	S	U
PRJNA413593	S	S	S	U
PRJNA413593	S	S	S	U
PRJNA413593	R	S	S	S
PRJNA413593	S	S	S	U
PRJNA413593	S	S	S	U

PRJNA413593	S	S	S	U
PRJNA413593	S	S	S	U
PRJNA413593	R	S	S	S
PRJNA413593	R	S	S	S
PRJNA413593	S	S	S	U
PRJNA413593	S	S	S	U
PRJNA413593	S	S	S	U
ERR2516053	S	S	S	S
ERR2515930	S	S	S	S
ERR2515940	S	S	S	S
ERR2515330	S	S	S	S
ERR2515219	S	S	S	S
ERR2516016	S	S	S	S
ERR2515272	S	S	S	S
ERR2515795	S	S	S	S
ERR2515229	S	S	S	S
ERR2515888	S	S	S	S
ERR2515534	S	S	S	S
ERR2515490	S	S	S	S
ERR2515771	S	S	S	S
ERR2515524	S	S	S	S
ERR2515855	S	S	S	S
ERR2515935	S	S	S	S
ERR2515848	S	S	S	S
ERR2515556	S	S	S	S
ERR2516054	S	S	S	S
ERR2515757	S	S	S	S
ERR2515657	S	S	S	S
ERR2515631	S	S	S	S
ERR2515704	S	S	U	S
ERR2516079	S	S	S	U
ERR2515941	S	S	S	S
ERR2515146	S	S	S	S
ERR2515942	S	S	S	S
ERR2515401	S	S	S	S
ERR2515170	S	S	S	S
ERR2516046	S	S	S	S
ERR2516011	R	S	S	S
ERR2515364	S	S	S	S
ERR2515925	S	S	S	S
ERR2515581	S	S	S	S
ERR2515408	R	S	S	S
ERR2515765	S	S	S	S
ERR2515716	S	S	S	S
ERR2515957	S	S	S	R
ERR2515955	S	S	S	S
ERR2515334	S	S	S	S
ERR2515604	S	S	S	S
ERR2515944	S	S	S	S
ERR2515886	S	S	S	S
ERR2515477	S	S	S	S
ERR2515195	S	R	S	S
ERR2515741	S	S	S	S

ERR2515416	S	S	S	S
ERR2515177	S	S	S	S
ERR2516072	S	S	S	S
ERR2515662	S	S	S	R
ERR2515649	S	S	S	S
ERR2516015	S	S	S	S
ERR2515607	S	S	S	S
ERR2515678	S	S	S	S
ERR2516083	S	S	S	S
ERR2515830	S	S	S	S
ERR2515939	S	S	S	S
ERR2515630	S	S	S	S
ERR2515216	S	S	S	S
ERR2515418	S	S	S	S
ERR2515762	S	S	S	S
ERR2515242	R	R	R	S
ERR2515695	S	S	S	S
ERR2515586	S	S	S	S
ERR2515484	S	S	S	S
ERR2515807	S	S	S	S
ERR2515269	S	S	S	S
ERR2515800	S	S	S	S
ERR2515452	S	S	S	S
ERR2515374	S	S	S	S
ERR2515321	S	S	S	S
ERR2515896	S	S	S	S
ERR2515675	S	S	S	S
ERR2515998	S	S	S	S
ERR2515644	S	S	S	S
ERR2515619	S	S	S	S
ERR2515959	S	S	S	S
ERR2515721	S	S	S	S
ERR2515766	S	S	S	S
ERR2515328	S	S	S	S
ERR2515460	S	S	S	S
ERR2515788	S	S	S	S
ERR2515923	S	S	S	S
ERR2516043	R	R	S	R
ERR2515863	S	S	S	S
ERR2515927	S	S	S	S
ERR2515282	S	S	S	S
ERR2516078	S	R	S	S
ERR2516027	R	S	S	R
ERR2515406	S	S	S	S
ERR2515887	S	S	S	S
ERR2515474	R	R	R	R
ERR2515145	S	S	S	S
ERR2516098	S	S	S	S
ERR2515387	S	R	S	S
ERR2515427	R	S	S	U
ERR2515179	S	S	S	S
ERR2515548	S	S	S	S
ERR2515694	S	S	S	S

ERR2515921	S	S	S	S
ERR2516013	S	S	S	S
ERR2515753	S	S	S	S
ERR2515799	S	S	S	S
ERR2515922	S	S	S	S
ERR2516009	S	S	S	S
ERR2515549	S	S	S	S
ERR2515947	S	S	S	S
ERR2515495	S	S	S	S
ERR2515281	S	S	S	S
ERR2515760	S	S	S	S
ERR2515850	S	S	S	S
ERR2516033	S	S	S	S
ERR2515234	S	S	S	S
ERR2515398	S	S	S	S
ERR2515239	S	S	S	S
ERR2515150	S	S	S	S
ERR2515557	S	S	S	S
ERR2515285	S	S	S	S
ERR2515531	S	S	S	S
ERR2515876	S	S	S	S
ERR2515892	S	S	S	S
ERR2515292	R	S	S	S
ERR2516093	S	S	S	S
ERR2515824	S	S	S	S
ERR2515601	S	S	S	S
ERR2515472	S	S	S	S
ERR2516092	S	S	S	S
ERR2515465	R	R	R	R
ERR2515203	S	S	S	S
ERR2515750	S	S	S	S
ERR2515987	S	S	S	S
ERR2515994	S	S	S	S
ERR2515279	S	S	S	S
ERR2516038	R	S	S	S
ERR2515844	S	S	S	S
ERR2515825	S	S	S	S
ERR2515637	R	R	S	S
ERR2515500	S	S	S	S
ERR2515700	S	S	S	S
ERR2516036	S	S	S	S
ERR2515526	S	S	S	S
ERR2515113	S	S	S	S
ERR2515355	S	S	S	S
ERR2515230	S	S	S	S
ERR2516070	R	R	S	R
ERR2515273	S	S	S	S
ERR2515470	S	S	S	S
ERR2515318	S	S	S	S
ERR2515108	S	S	S	S
ERR2515814	S	S	S	S
ERR2515690	S	S	S	S
ERR2516044	S	S	S	S

ERR2515908	S	S	S	S
ERR2515274	S	S	S	S
ERR2515308	S	S	S	S
ERR2515772	S	S	S	S
ERR2515287	S	S	S	S
ERR2515533	S	S	S	S
ERR2515943	S	S	S	U
ERR2515672	S	S	S	S
ERR2515629	S	S	S	S
ERR2515288	S	S	S	S
ERR2515817	S	S	S	S
ERR2515142	S	S	U	S
ERR2515986	S	S	S	S
ERR2515599	S	S	S	S
ERR2515671	S	S	S	S
ERR2515999	S	S	S	S
ERR2515641	S	S	S	U
SRR671775	S	S	S	U
SRR671722	R	R	S	U
SRR671721	R	R	R	U
SRR671868	R	R	R	U
SRR671869	R	R	S	U
SRR671867	R	R	S	U
SRR671865	R	R	R	U
SRR671724	R	R	R	U
SRR671720	R	R	R	U
SRR671866	R	R	R	U
SRR671780	R	R	S	U
SRR671779	R	R	S	U
SRR671776	S	S	S	U
SRR671777	S	S	S	U
SRR671778	S	S	S	U
SRR671719	R	R	R	U
SRR671727	R	R	S	U
SRR671723	S	S	S	U
SRR671858	S	S	S	U
SRR671859	S	S	S	U
SRR671860	S	S	S	U
SRR671728	R	R	R	U
SRR671729	S	S	S	U
SRR671730	S	S	S	U
SRR671731	R	R	S	U
SRR671762	S	S	S	U
SRR671748	R	R	R	U
SRR671749	R	R	S	U
SRR671750	R	R	S	U
SRR671751	R	R	R	U
SRR671752	R	R	S	U
SRR671841	R	R	R	U
SRR671753	R	R	S	U
SRR671758	R	R	S	U
SRR671757	R	R	S	U
SRR671759	R	R	R	U

SRR671763	S	S	S	U
SRR671832	S	S	S	U
SRR671764	S	S	S	U
SRR671760	R	R	S	U
SRR671755	R	R	S	U
SRR671842	R	R	R	U
SRR671756	R	R	R	U
SRR671754	R	R	R	U
SRR671761	R	R	R	U
SRR671732	R	R	R	U
SRR671833	S	S	S	U
SRR671843	R	R	S	U
SRR671834	R	R	R	U
SRR671835	S	S	S	U
SRR671769	R	R	R	U
SRR671772	R	R	R	U
SRR671770	R	R	R	U
SRR671767	R	R	R	U
SRR671765	R	R	R	U
SRR671844	R	R	R	U
SRR671771	R	R	R	U
SRR671774	R	R	S	U
SRR671766	R	R	R	U
SRR671768	R	R	S	U
SRR671773	S	S	S	U
SRR671845	R	R	R	U
SRR671836	R	R	S	U
SRR671837	R	R	R	U
SRR671846	S	S	S	U
SRR671847	R	R	S	U
SRR671863	R	R	R	U
SRR671861	R	R	S	U
SRR671864	R	R	R	U
SRR671862	R	R	R	U
SRR671789	R	R	S	U
SRR671783	R	R	R	U
SRR671790	R	R	R	U
SRR671782	R	R	R	U
SRR671725	R	R	R	U
SRR671726	R	R	R	U
SRR671733	R	R	S	U
SRR671857	R	R	R	U
SRR671856	S	S	S	U
SRR671734	R	R	S	U
SRR671735	R	R	S	U
SRR671736	R	R	S	U
SRR671784	R	R	S	U
SRR671788	S	S	S	U
SRR671855	R	R	R	U
SRR671781	R	R	R	U
SRR671786	S	S	S	U
SRR671787	R	R	R	U
SRR671785	R	R	R	U

SRR671794	R	R	R	U
SRR671792	S	S	S	U
SRR671800	S	S	S	U
SRR671797	R	R	R	U
SRR671796	R	R	R	U
SRR671829	R	R	S	U
SRR671799	R	R	R	U
SRR671801	R	R	S	U
SRR671791	R	R	S	U
SRR671854	R	R	R	U
SRR671737	R	R	R	U
SRR671739	R	R	R	U
SRR671879	S	S	S	U
SRR671870	S	S	S	U
SRR671875	S	S	S	U
SRR671877	S	S	S	U
SRR671876	S	S	S	U
SRR671740	R	R	S	U
SRR671741	S	S	S	U
SRR671742	R	R	S	U
SRR671743	R	R	S	U
SRR671744	R	R	S	U
SRR671804	R	R	R	U
SRR671827	S	S	S	U
SRR671810	R	R	R	U
SRR671811	R	R	R	U
SRR671805	S	S	S	U
SRR671806	R	R	R	U
SRR671807	R	R	R	U
SRR671808	S	S	S	U
SRR671809	R	R	R	U
SRR671812	R	R	S	U
SRR671813	R	R	R	U
SRR671814	S	S	S	U
SRR671815	R	R	R	U
SRR671816	R	R	S	U
SRR671817	R	R	S	U
SRR671818	R	R	R	U
SRR671819	R	R	S	U
SRR671820	R	R	S	U
SRR671821	R	R	R	U
SRR671822	R	R	R	U
SRR671823	S	S	S	U
SRR671824	R	R	S	U
SRR671825	R	R	R	U
SRR671826	R	R	R	U
SRR671828	R	R	S	U
SRR671853	R	R	R	U
SRR671852	R	R	S	U
SRR671795	R	R	S	U
SRR671830	S	S	S	U
SRR671831	R	R	S	U
SRR671802	S	S	S	U

SRR671793	S	S	S	U
SRR671798	R	R	S	U
SRR671803	S	S	S	U
SRR671745	R	R	S	U
SRR671746	R	R	S	U
SRR671738	R	R	S	U
SRR671871	S	S	S	U
SRR671873	S	S	S	U
SRR671878	S	S	S	U
SRR671872	S	S	S	U
SRR671874	S	S	S	U
SRR671848	R	R	S	U
SRR671838	R	R	S	U
SRR671849	R	R	R	U
SRR671839	R	R	R	U
SRR671850	R	R	R	U
SRR671851	R	R	R	U
SRR671840	R	R	R	U
SRR671747	S	S	S	U
ERR2515879	S	S	S	S
ERR2515953	S	S	S	S
ERR2515595	S	S	S	S
ERR2515976	R	S	S	S
ERR2515912	S	S	S	S
ERR2515713	R	R	S	R
ERR2515989	S	S	S	S
ERR2515313	S	S	S	S
ERR2516006	S	R	S	S
ERR2516099	S	S	S	S
ERR2515873	S	S	S	S
ERR2516065	S	S	S	S
ERR2515489	S	S	S	S
ERR2515491	R	S	S	S
ERR2515208	S	S	S	S
ERR2515610	S	S	S	S
ERR2515569	S	S	S	S
ERR2515685	S	S	S	S
ERR2516047	R	R	R	R
ERR2515169	R	S	S	S
ERR2515598	S	S	S	S
ERR2515958	S	S	S	S
ERR2515784	S	S	S	S
ERR2515875	S	S	S	S
ERR2515854	S	S	S	S
ERR2515305	S	S	S	S
ERR2515136	S	S	S	S
ERR2515587	S	S	S	S
ERR2515600	S	S	S	S
ERR2515525	S	S	S	S
ERR2515532	R	R	S	S
ERR2515180	S	S	S	S
ERR2515728	S	S	S	S
ERR2515324	S	S	S	S

ERR2515906	S	S	S	S
ERR2515385	R	S	S	S
ERR2515501	S	S	S	S
ERR2515632	S	S	S	S
ERR2515449	R	S	S	S
ERR2515158	S	S	S	S
ERR2515960	S	S	S	S
ERR2515326	S	S	S	S
ERR2515834	R	U	S	U
ERR2515327	S	S	S	S
ERR2515397	S	S	S	S
ERR2515654	S	S	S	S
ERR2515403	S	S	S	S
ERR2515734	S	S	S	S
ERR2515505	S	S	S	S
ERR2515787	S	S	S	S
ERR2515457	S	S	S	S
ERR2515579	S	S	S	S
ERR2515919	S	S	S	S
ERR2515212	S	S	S	S
ERR2515639	S	S	S	S
ERR2515106	S	S	S	S
ERR2515602	S	S	S	S
ERR2515847	S	S	S	S
ERR2515414	S	S	S	S
ERR2516101	S	S	R	S
ERR2515992	S	S	S	S
ERR2515871	S	S	S	S
ERR2515275	S	S	S	S
ERR2515946	S	S	S	S
ERR2515235	S	S	S	S
ERR2515232	S	S	S	S
ERR2516008	R	R	S	R
ERR2515110	S	S	S	S
ERR2515139	S	S	S	S
ERR2516095	S	S	S	S
ERR2515699	S	S	S	S
ERR2515894	S	S	S	S
ERR2515151	S	S	S	S
ERR2515149	S	S	S	S
ERR2516087	S	S	S	S
ERR2515412	S	S	S	S
ERR2515214	S	S	S	S
ERR2515422	S	S	S	S
ERR2515669	S	U	S	S
ERR2515167	S	S	S	S
ERR2515431	S	S	S	S
ERR2515260	S	S	S	S
ERR2515566	R	S	S	S
ERR2515717	R	S	S	S
ERR2515432	S	S	S	S
ERR2516035	S	S	S	S
ERR2515140	S	S	S	S

ERR2515837	R	S	S	U
ERR2515884	S	S	S	S
ERR2515698	S	S	S	S
ERR2515570	S	S	S	S
ERR2515849	S	S	S	S
ERR2515733	S	S	S	S
ERR2515295	S	S	S	S
ERR2515122	S	S	S	S
ERR2515877	S	S	S	S
ERR2515443	S	S	S	S
ERR2515383	S	S	S	S
ERR2515529	S	S	S	S
ERR2515974	S	S	S	R
ERR2516100	S	S	S	S
ERR2515782	S	S	S	S
ERR2515592	S	S	S	S
ERR2515222	S	S	S	S
ERR2515975	S	S	S	S
ERR2516075	S	S	S	S
ERR2515399	S	S	S	S
ERR2515265	R	R	R	R
ERR2515499	S	S	S	S
ERR2515823	S	S	S	S
ERR2515625	S	S	S	S
ERR2515521	S	S	S	S
ERR2515200	S	S	S	S
ERR2515655	S	S	S	S
ERR2516096	S	S	S	S
ERR2516002	S	S	S	S
ERR2515993	R	S	S	S
ERR2515420	S	S	S	S
ERR2515240	S	S	S	S
ERR2515502	S	S	S	S
ERR2516022	S	S	S	S
ERR2515967	S	S	S	S
ERR2516058	S	S	S	S
ERR2515881	R	R	S	S
ERR2515271	S	S	S	U
ERR2515786	S	S	S	S
ERR2516089	S	S	S	S
ERR2515808	S	S	S	S
ERR2515578	S	S	S	S
ERR2515341	S	S	S	S
ERR2515832	S	S	S	S
ERR2515676	S	S	S	S
ERR2515440	S	S	S	S
ERR2515846	S	S	S	S
ERR2515297	S	S	S	S
ERR2515977	S	S	S	S
ERR2515861	S	S	S	S
ERR2515897	S	S	S	S
ERR2515206	S	S	S	S
ERR2515365	S	S	S	S

ERR2515157	S	S	S	S
ERR2515481	S	S	S	S
ERR2515712	S	S	S	S
ERR2515315	S	S	S	S
ERR2515447	S	S	S	S
ERR2516061	S	S	S	S
ERR2515309	S	S	S	S
ERR2515197	S	S	S	S
ERR2515253	S	S	S	S
ERR2515538	S	S	S	S
ERR2515236	S	S	S	S
ERR2515635	S	S	S	S
ERR2515805	S	S	S	S
ERR2515726	S	S	S	S
ERR2515840	S	S	S	S
ERR2515605	S	S	S	S
ERR2516021	S	S	S	S
ERR2515444	S	S	S	S
ERR2515132	S	S	S	S
ERR2515613	S	S	S	S
ERR2515880	S	S	S	S
ERR2515300	S	S	S	S
SAMN03647097	S	S	S	S
SAMN03647098	S	S	S	S
SAMN03647099	S	S	S	S
SAMN03647100	S	S	S	S
SAMN03647101	S	S	S	S
SAMN03647102	S	S	S	S
SAMN03647103	S	S	S	S
SAMN03647104	S	S	S	S
SAMN03647105	S	S	S	S
SAMN03647106	S	S	S	S
SAMN03647107	S	S	S	S
SAMN03647108	S	S	S	S
SAMN03647109	S	S	S	S
SAMN03647110	S	S	S	S
SAMN03647111	S	S	S	S
SAMN03647112	S	S	S	S
SAMN03647113	S	S	S	S
SAMN03647114	S	S	S	S
SAMN03647115	S	S	S	S
SAMN03647116	S	S	S	S
SAMN03647117	S	S	S	S
SAMN03647118	S	S	S	S
SAMN03647119	S	S	S	S
SAMN03647120	S	S	S	S
SAMN03647121	S	S	S	S
SAMN03647122	R	R	S	S
SAMN03647123	S	S	S	S
SAMN03647124	S	S	S	S
SAMN03647125	S	S	S	S
SAMN03647126	S	S	S	S
SAMN03647127	S	S	S	S

SAMN03647128	S	S	S	
SAMN03647129	R	R	S	S
SAMN03647130	S	S	S	S
SAMN03647131	R	S	S	S
SAMN03647132	R	S	S	S
SAMN03647133	R	S	S	S
SAMN03647134	R	S	S	S
SAMN03647135	S	S	S	
SAMN03647136	R	S	S	S
SAMN03647137	R	S	S	S
SAMN03647138	R	S	S	S
SAMN03647139	R	S	S	S
SAMN03647140	R	S	S	S
SAMN03647141	R	S	S	S
SAMN03647142	R	R	R	R
SAMN03647143	R	S	S	S
SAMN03647144	R	S	S	S
SAMN03647145	S	R	R	
SAMN03647146	R	S	S	S
SAMN03647147	R	R	R	S
SAMN03647148	R	S	S	S
SAMN03647149	R	R	S	S
SAMN03647150	R	S	S	S
SAMN03647151	R	R	S	S
SAMN03647152	R	S	S	S
SAMN03647153	R	S	S	S
SAMN03647154	R	S	S	S
SAMN03647155	R	R	S	S
SAMN03647156	R	S	S	S
SAMN03647157	R	S	S	S
SAMN03647158	R	S	S	S
SAMN03647159	R	S	S	S
SAMN03647160	R	S	S	S
SAMN03647161	R	S	S	S
SAMN03647162	R	S	S	S
SAMN03647163	S	S	S	S
SAMN03647164	S	S	S	S
SAMN03647165	S	S	S	S
SAMN03647166	S	S	S	S
SAMN03647167	S	S	S	S
SAMN03647168	S	S	S	S
SAMN03647169	S	S	S	S
SAMN03647170	S	S	S	S
SAMN03647171	S	S	S	S
SAMN03647172	S	S	S	S
SAMN03647173	S	S	S	S
SAMN03647174	S	S	S	S
SAMN03647175	S	S	S	S
SAMN03647176	S	S	S	S
SAMN03647177	S	S	S	S
SAMN03647178	S	S	S	S
SAMN03647179	R	S	S	S
SAMN03647180	S	S	S	S

SAMN03647181	S	S	S	S
SAMN03647182	S	S	S	S
SAMN03647183	S	S	S	S
SAMN03647184	S	S	S	S
SAMN03647185	S	S	S	S
SAMN03647186	S	S	S	S
SAMN03647187	S	S	S	S
SAMN03647188	R	S	S	S
SAMN03647189	S	S	S	S
SAMN03647190	S	S	S	S
SAMN03647191	S	S	S	S
SAMN03647192	S	S	S	S
SAMN03647193	S	S	S	S
SAMN03647194	R	R	S	S
SAMN03647195	S	S	S	S
SAMN03647196	S	S	S	S
SAMN03647197	S	S	S	S
SAMN03647198	S	S	S	S
SAMN03647199	R	R	S	S
SAMN03647200	S	S	S	S
SAMN03647201	S	S	S	S
SAMN03647202	S	S	S	S
SAMN03647203	S	S	S	S
SAMN03647204	R	S	S	S
SAMN03647205	R	S	S	S
SAMN03647206	S	S	S	S
SAMN03647207	S	S	S	S
SAMN03647208	S	S	S	S
SAMN03647209	S	S	S	S
SAMN03647210	S	S	S	S
SAMN03647211	S	S	S	S
SAMN03647212	S	S	S	S
SAMN03647213	S	S	S	S
SAMN03647214	S	S	S	S
SAMN03647215	S	S	S	S
SAMN03647216	S	S	S	S
SAMN03647217	S	S	S	S
SAMN03647218	S	S	S	S
SAMN03647219	S	S	S	S
SAMN03647220	S	S	S	S
SAMN03647221	S	S	S	S
SAMN03647222	S	S	S	S
SAMN03647223	S	S	S	S
SAMN03647224	S	S	S	S
SAMN03647225	S	S	S	S
SAMN03647226	S	S	S	S
SAMN03647227	S	S	S	S
SAMN03647228	S	S	S	S
SAMN03647229	S	S	S	S
SAMN03647230	S	S	S	S
SAMN03647231	R	S	S	S
SAMN03647232	S	S	S	S
SAMN03647233	S	S	S	S

SAMN03647234	S	S	S	S
SAMN03647235	S	S	S	S
SAMN03647236	S	S	S	S
SAMN03647237	S	S	S	S
SAMN03647238	R	S	S	S
ERR025833	S	S	S	S
ERR025834	S	S	S	S
ERR025835	S	S	S	S
ERR025836	S	S	S	S
ERR025837	S	S	S	S
ERR025838	S	S	S	S
ERR025839	S	S	S	S
ERR025842	S	S	S	S
ERR025843	S	S	S	S
ERR025844	S	S	S	S
ERR025846	S	S	S	S
ERR025847	S	S	S	S
ERR025848	S	S	S	S
ERR038254	S	S	S	S
ERR038255	S	S	S	S
ERR038256	S	S	S	S
ERR038257	S	S	S	S
ERR038258	S	S	S	S
ERR038259	S	S	S	S
ERR038260	S	S	S	S
ERR038261	S	S	S	S
ERR038262	S	S	S	S
ERR038263	S	S	S	S
ERR038264	S	S	S	S
ERR038265	S	S	S	S
ERR038266	S	S	S	S
SAMN03647239	S	S	S	S
ERR038269	S	S	S	S
ERR038270	S	S	S	S
ERR038271	S	S	S	S
ERR038272	S	S	S	S
ERR038273	S	S	S	S
ERR038274	S	S	S	S
ERR038275	S	S	S	S
ERR038276	S	S	S	S
ERR038277	S	S	S	S
ERR038278	S	S	S	S
ERR038279	S	S	S	S
ERR038280	S	S	S	S
ERR038281	S	S	S	S
ERR038282	S	S	S	S
ERR038283	S	S	S	S
ERR038284	S	S	S	S
ERR038285	S	S	S	S
ERR038286	S	S	S	S
ERR038287	S	S	S	S
ERR038288	S	S	S	S
SAMN03647240	S	S	S	S

ERR038290	S	S	S	S
ERR038291	S	S	S	S
ERR038292	S	S	S	S
ERR038293	S	S	S	S
ERR038294	S	S	S	S
ERR038295	S	S	S	S
ERR038296	S	S	S	S
ERR038298	S	S	S	S
ERR038299	S	S	S	S
ERR038300	S	S	S	S
ERR039323	S	S	S	S
ERR039324	S	S	S	S
ERR039325	S	S	S	S
ERR039326	S	S	S	S
ERR039327	S	S	S	S
ERR039328	S	S	S	S
ERR039329	S	S	S	S
ERR039330	S	S	S	S
ERR039331	S	S	S	S
ERR039332	S	S	S	S
ERR039333	S	S	S	S
ERR039334	S	S	S	S
ERR039335	S	S	S	S
ERR039336	S	S	S	S
ERR039337	S	S	S	S
ERR039338	S	S	S	S
ERR039339	S	S	S	S
ERR039340	S	S	S	S
ERR039341	S	S	S	S
ERR039342	S	S	S	S
ERR039343	S	S	S	S
ERR039344	S	S	S	S
ERR039345	S	S	S	S
ERR039346	S	S	S	S
ERR040086	S	S	S	S
ERR040087	S	S	S	S
ERR040088	S	S	S	S
ERR040089	S	S	S	S
ERR040090	S	S	S	S
ERR040091	S	S	S	S
SAMN03647241	S	S	S	S
ERR040093	S	S	S	S
ERR040094	S	S	S	S
ERR040095	S	S	S	S
ERR040096	S	S	S	S
ERR040097	S	S	S	S
ERR040098	S	S	S	S
ERR040099	S	S	S	S
ERR040100	S	S	S	S
ERR040101	S	S	S	S
ERR040102	S	S	S	S
ERR040103	S	S	S	S
ERR040104	S	S	S	S

ERR040105	R	S	S	S
ERR040106	S	S	S	S
ERR040107	S	S	S	S
ERR040108	S	S	S	S
ERR040109	S	S	S	S
ERR046729	S	S	S	S
ERR046730	R	S	S	S
SAMN03647242	S	S	S	S
ERR046732	S	S	S	S
ERR046733	S	S	S	S
ERR046734	S	S	S	S
ERR046735	S	S	S	S
ERR046736	S	S	S	S
ERR046737	S	S	S	S
ERR046738	S	S	S	S
ERR046739	S	S	S	S
SAMN03647243	S	S	S	S
ERR046741	S	S	S	S
SAMN03647244	S	S	S	S
ERR046743	S	S	S	S
ERR046744	S	S	S	S
ERR046745	S	S	S	S
ERR046746	S	S	S	S
ERR046747	S	S	S	R
ERR046748	S	S	S	R
ERR046749	S	S	S	R
SAMN03647245	S	S	S	R
ERR046751	S	S	S	S
ERR046752	S	S	S	S
ERR046753	S	S	S	S
ERR046754	S	S	S	S
ERR046755	S	S	S	S
ERR046756	S	S	S	S
SAMN03647246	S	S	S	S
ERR046758	S	S	S	S
ERR046759	S	S	S	S
ERR046760	S	S	S	S
ERR046761	S	S	S	S
ERR046762	S	S	S	S
ERR046763	S	S	S	S
ERR046764	S	S	S	S
ERR046765	S	S	S	S
ERR046766	S	S	S	S
ERR046767	S	S	S	S
ERR046768	S	S	S	S
ERR046769	S	S	S	S
ERR046770	S	S	S	S
ERR046771	S	S	S	S
ERR046772	S	S	S	S
ERR046773	S	S	S	S
SAMN03647247	S	S	S	R
ERR046775	S	S	S	S
ERR046776	S	S	S	S

ERR046777	S	S	S	S
ERR046778	R	S	S	S
ERR046779	S	S	S	S
ERR046780	R	S	S	S
ERR046781	S	S	S	S
ERR046782	S	S	S	S
ERR046783	S	S	S	S
ERR046784	R	S	R	S
ERR046785	S	S	S	S
ERR046786	R	S	S	S
ERR046788	R	S	S	S
ERR046789	R	S	S	S
ERR046791	S	S	S	S
ERR046792	S	S	R	S
ERR046794	S	S	S	S
ERR046795	S	S	S	S
ERR046796	R	R	R	R
ERR046797	S	S	S	S
ERR046798	S	S	S	S
ERR046799	S	S	S	S
ERR046800	S	S	S	S
SAMN03647248	S	S	S	S
SAMN03647249	S	S	S	S
SAMN03647250	S	S	S	S
SAMN03647251	S	S	S	S
SAMN03647252	S	S	S	S
SAMN03647253	S	S	S	S
SAMN03647254	S	S	S	S
SAMN03647255	S	S	S	S
SAMN03647256	S	S	S	S
SAMN03647257	S	S	S	S
SAMN03647258	S	S	S	S
SAMN03647259	S	S	S	S
SAMN03647260	S	S	S	S
ERR046819	S	S	S	S
ERR046820	S	S	S	S
ERR046821	R	R	S	U
ERR046822	S	S	S	S
ERR046823	S	S	S	S
ERR046824	S	S	S	S
ERR046825	S	R	S	S
SAMN03647261	S	S	S	S
SAMN03647262	S	S	S	S
SAMN03647263	S	S	S	S
SAMN03647264	S	S	S	S
ERR046831	S	S	S	S
ERR046832	S	S	S	S
ERR046834	R	S	S	S
ERR046837	S	S	S	S
ERR046838	S	S	S	S
ERR046839	S	S	S	S
ERR046840	S	S	S	S
ERR046841	S	S	S	S

ERR046842	S	S	S	S
ERR046843	S	S	S	S
ERR046844	S	S	S	S
ERR046845	S	S	S	S
ERR046846	S	S	S	S
ERR046847	S	S	S	S
ERR046849	S	S	S	S
ERR046850	S	S	S	S
ERR046851	S	S	S	S
ERR046852	S	S	S	S
ERR046853	S	S	S	S
ERR046854	S	S	S	S
ERR046855	R	R	S	S
ERR046857	S	S	S	S
ERR046858	S	S	S	S
ERR046859	S	S	S	S
ERR046860	S	S	S	S
ERR046861	S	S	S	S
ERR046862	S	S	S	S
ERR046863	S	S	S	S
ERR046864	S	S	S	S
ERR046865	S	S	S	S
ERR046866	S	S	S	S
ERR046867	S	S	S	S
ERR046869	S	S	S	S
ERR046871	S	S	S	S
ERR046872	S	S	S	S
ERR046873	S	S	S	S
ERR046874	S	S	S	S
ERR046875	S	S	S	S
ERR046876	S	S	S	S
ERR046877	S	S	S	S
ERR046878	S	S	S	S
ERR046879	S	S	S	S
ERR046880	S	S	S	S
ERR046881	S	S	S	S
ERR046882	S	S	S	S
ERR046883	S	S	S	S
ERR046884	S	S	S	S
ERR046885	S	S	S	S
ERR046898	S	S	S	S
ERR046887	S	S	S	S
ERR046888	S	S	S	S
ERR046889	S	S	S	S
ERR046890	S	S	S	S
ERR046891	S	S	S	S
ERR046893	S	S	S	S
ERR046894	S	S	S	S
ERR046895	S	S	S	S
ERR046897	S	S	S	S
ERR046833	S	S	S	S
SAMN03647265	S	S	S	S
ERR046901	S	S	S	S

ERR046903	R	R	R	R
ERR046904	S	S	S	S
ERR046905	S	S	S	S
ERR046907	S	S	S	S
ERR046908	S	S	S	S
ERR046910	S	S	S	S
ERR046911	S	S	R	S
ERR046912	S	S	S	S
ERR046913	S	S	R	S
ERR046914	S	S	S	S
ERR046915	S	S	S	S
ERR046916	S	S	S	S
ERR046917	R	R	R	R
ERR046918	S	S	S	S
ERR046919	S	S	S	S
ERR046920	S	S	S	S
ERR046921	S	S	S	S
ERR046922	S	S	S	S
ERR046923	S	S	S	S
ERR046924	S	S	S	S
ERR046925	S	S	S	S
ERR046926	S	S	S	S
ERR046928	S	S	S	S
ERR046929	S	S	S	S
ERR046930	S	S	S	S
SAMN03647266	S	S	S	S
ERR046932	S	S	S	S
ERR046933	R	U	U	S
ERR046936	S	S	S	S
ERR046937	U	U	S	U
ERR046938	S	S	S	S
ERR046939	S	S	S	S
ERR046940	S	S	S	S
ERR046941	S	S	S	S
ERR046942	S	S	S	S
ERR046943	R	R	S	S
ERR046945	S	S	S	S
ERR046946	S	S	S	S
ERR046947	S	S	S	S
ERR046948	S	S	S	S
ERR046949	S	S	S	S
ERR046950	S	S	S	S
ERR046951	S	S	S	S
ERR046952	S	S	S	S
ERR046953	S	S	S	S
ERR046954	S	S	S	R
SAMN03647267	S	S	S	S
ERR046957	S	S	S	S
ERR046958	S	S	S	S
ERR046959	S	S	S	S
ERR046960	S	S	S	S
ERR046961	S	S	S	R
ERR046963	S	S	S	S

ERR046964	S	S	S	S
ERR046965	S	S	S	S
ERR046966	S	S	S	S
ERR046967	S	S	S	S
ERR046968	S	S	S	S
ERR046969	S	S	S	S
ERR046970	S	S	S	S
ERR046971	S	S	S	S
ERR046972	S	S	S	S
SAMN03647268	S	S	S	S
ERR046974	S	S	S	S
ERR046975	S	S	S	S
SAMN03647269	S	S	S	S
SAMN03647270	R	S	S	R
SAMN03647271	S	S	S	S
SAMN03647272	S	S	S	S
ERR046980	S	S	S	S
ERR046981	R	S	S	S
ERR046982	S	S	S	S
ERR046983	S	S	S	S
SAMN03647273	S	S	S	S
SAMN03647274	R	R	S	S
ERR046986	R	S	S	S
SAMN03647275	S	S	S	S
ERR046988	S	S	S	S
ERR046989	S	S	S	R
ERR046990	S	S	S	S
ERR046991	S	S	S	S
ERR046992	S	S	S	S
ERR046993	S	S	S	S
ERR046994	S	S	S	S
ERR046995	S	S	S	S
ERR046996	S	S	S	S
ERR046997	S	S	S	S
ERR046998	S	S	S	S
ERR046999	S	S	S	S
ERR047000	S	S	S	S
ERR047002	R	S	S	S
ERR047003	S	S	S	S
ERR047004	S	S	S	S
ERR047005	S	S	S	S
ERR047006	S	S	S	S
ERR047007	S	S	S	S
ERR047008	S	S	S	S
ERR047009	S	S	S	S
ERR047010	S	S	S	S
ERR047011	S	S	S	S
ERR047012	S	S	S	S
ERR047013	S	S	S	S
ERR047014	S	S	S	S
SAMN03647276	S	S	S	S
ERR047016	S	S	S	S
ERR072089	S	S	S	S

SAMN03647277	S	S	S	S
SAMN03647278	S	S	S	S
ERR072019	S	S	S	S
ERR072020	S	S	S	S
ERR072021	S	S	S	S
ERR072022	S	S	S	S
ERR072023	S	S	S	S
ERR072024	S	S	S	S
ERR072025	S	S	S	S
ERR072026	S	S	S	S
ERR072027	S	S	S	S
ERR072028	S	S	S	S
ERR072029	S	S	S	S
ERR072030	S	S	S	S
ERR072031	S	S	S	S
ERR072032	S	S	S	S
ERR072034	S	S	S	S
ERR072035	S	S	S	S
ERR072036	S	S	S	S
ERR072037	S	S	S	U
ERR072038	S	S	S	S
ERR072039	S	S	S	S
ERR072040	S	S	S	S
ERR072041	S	S	S	S
ERR072042	S	S	S	S
SAMN03647279	S	S	S	S
ERR072044	S	S	S	S
ERR072045	S	S	S	S
ERR072046	S	S	S	S
ERR072047	S	S	S	S
ERR072048	S	S	S	S
SAMN03647280	S	S	S	S
ERR072050	S	S	S	S
ERR072051	S	S	S	S
SAMN03647281	R	S	S	S
SAMN03647282	S	S	S	S
SAMN03647283	S	S	S	S
SAMN03647284	S	S	S	S
SAMN03647285	S	S	S	S
SAMN03647286	S	S	S	S
SAMN03647287	S	S	S	S
SAMN03647288	S	S	S	S
SAMN03647289	S	S	S	S
SAMN03647290	S	S	S	S
ERR072065	S	S	S	S
SAMN03647291	S	S	S	S
SAMN03647292	S	S	S	S
SAMN03647293	S	S	S	S
SAMN03647294	S	S	S	S
SAMN03647295	S	S	S	S
SAMN03647296	S	S	S	S
ERR072072	S	S	S	S
SAMN03647297	S	S	S	S

SAMN03647298	S	S	S	S
SAMN03647299	S	S	S	S
SAMN03647300	S	S	S	S
ERR072077	S	S	S	S
SAMN03647301	S	S	S	S
SAMN03647302	S	S	S	S
ERR072080	S	S	S	S
SAMN03647303	S	S	S	S
SAMN03647304	R	S	S	R
SAMN03647305	R	R	R	R
SAMN03647306	S	S	S	S
SAMN03647307	S	S	S	S
ERR072087	S	S	S	S
SAMN03647308	S	S	S	S
SAMN03647309	S	S	S	S
SAMN03647310	S	S	S	S
SAMN03647311	S	S	S	S
ERR072094	S	S	S	S
ERR072095	S	S	S	S
ERR072096	S	S	S	S
SAMN03647312	S	S	S	S
SAMN03647313	S	S	S	S
SAMN03647314	S	S	S	S
SAMN03647315	S	S	S	S
SAMN03647316	S	S	S	S
SAMN03647317	S	S	S	S
ERS389340	R	R	R	R
ERS389341	S	S	S	S
SAMN03647318	S	S	S	S
SAMN03647319	S	S	S	S
SAMN03647320	S	S	S	S
SAMN03647321	R	S	S	S
SAMN03647322	R	S	S	U
SAMN03647323	S	S	S	S
SAMN03647324	S	S	S	S
SAMN03647325	S	S	S	S
SAMN03647326	S	S	S	S
SAMN03647327	S	S	S	S
SAMN03647328	S	S	S	S
SAMN03647329	R	S	S	S
SAMN03647330	R	S	S	S
SAMN03647331	R	S	S	S
SAMN03647332	R	S	S	S
SAMN03647333	S	S	S	S
SAMN03647334	S	S	S	S
SAMN03647335	S	S	S	S
SAMN03647336	S	S	S	S
SAMN03647337	S	S	S	S
SAMN03647338	S	S	S	S
SAMN03647339	R	S	S	S
SAMN03647340	R	S	S	S
SAMN03647341	R	S	S	S
SAMN03647342	S	S	S	S

SAMN03647343	S	S	S	S
SAMN03647344	S	S	S	S
SAMN03647345	S	S	S	S
SAMN03647346	S	S	S	S
SAMN03647347	S	S	S	S
SAMN03647348	R	S	S	S
SAMN03647349	R	S	S	S
SAMN03647350	R	S	S	S
SAMN03647351	R	S	S	S
SAMN03647352	R	S	S	S
SAMN03647353	S	S	S	S
SAMN03647354	S	S	S	S
SAMN03647355	S	S	S	S
SAMN03647356	S	S	S	S
SAMN03647357	S	S	S	S
SAMN03647358	S	S	S	S
SAMN03647359	R	S	S	S
SAMN03647360	R	S	S	S
SAMN03647361	R	S	S	S
SAMN03647362	R	S	S	S
SAMN03647363	S	S	S	S
SAMN03647364	S	S	S	S
SAMN03647365	S	S	S	S
SAMN03647366	S	S	S	S
SAMN03647367	R	S	S	S
SAMN03647368	S	S	S	S
SAMN03647369	R	S	S	S
SAMN03647370	R	S	S	S
SAMN03647371	R	S	S	S
SAMN03647372	S	S	S	S
SAMN03647373	S	S	S	S
SAMN03647374	S	S	S	S
SAMN03647375	S	S	S	S
SAMN03647376	S	S	S	S
SAMN03647377	S	S	S	S
SAMN03647378	S	S	S	S
SAMN03647379	S	R	S	S
SAMN03647380	R	S	S	S
SAMN03647381	R	S	S	S
SAMN03647382	R	S	S	S
SAMN03647383	S	S	S	S
SAMN03647384	S	S	S	S
SAMN03647385	S	S	S	S
SAMN03647386	S	S	S	S
SAMN03647387	S	S	S	S
SAMN03647388	R	S	S	S
SAMN03647389	R	S	S	S
SAMN03647390	R	R	S	S
SAMN03647391	R	S	S	S
SAMN03647392	R	S	S	S
SAMN03647393	R	S	S	S
SAMN03647394	S	S	S	S
SAMN03647395	S	S	S	S

SAMN03647396	S	S	S	S
SAMN03647397	S	S	S	S
SAMN03647398	R	S	S	S
SAMN03647399	S	R	S	S
SAMN03647400	R	R	R	S
SAMN03647401	R	S	R	S
SAMN03647402	R	S	S	S
SAMN03647403	S	S	S	S
SAMN03647404	R	R	R	S
SAMN03647405	S	S	S	S
SAMN03647406	S	S	S	S
SAMN03647407	R	R	R	S
SAMN03647408	S	S	S	S
SAMN03647409	S	S	S	S
SAMN03647410	S	S	S	S
SAMN03647411	R	R	S	R
SAMN03647412	S	R	R	
SAMN03647413	R	S	S	S
SAMN03647414	U	U	U	S
SAMN03647415	S	S	S	S
SAMN03647416	S	S	S	S
SAMN03647417	S	S	S	S
SAMN03647418	R	S	S	S
SAMN03647419	R	R	S	S
SAMN03647420	S	S	S	S
SAMN03647421	R	S	S	S
SAMN03647422	R	S	S	S
SAMN03647423	R	R	S	S
SAMN03647424	R	S	S	S
SAMN03647425	S	S	S	S
SAMN03647426	S	S	S	S
SAMN03647427	S	S	S	S
SAMN03647428	S	S	S	S
SAMN03647429	S	S	S	S
SAMN03647430	R	R	R	S
SAMN03647431	R	S	S	S
SAMN03647432	R	S	S	S
SAMN03647433	R	S	S	S
SAMN03647434	R	S	S	S
SAMN03647435	S	S	S	S
SAMN03647436	S	S	S	S
SAMN03647437	S	S	S	S
SAMN03647438	S	S	S	S
SAMN03647439	S	S	S	S
SAMN03647440	R	R	R	S
SAMN03647441	S	S	S	S
SAMN03647442	S	S	S	S
SAMN03647443	R	S	S	S
SAMN03647444	R	S	S	S
SAMN03647445	R	S	S	S
SAMN03647446	S	S	S	S
SAMN03647447	S	S	S	S
SAMN03647448	S	S	S	S

SAMN03647449	S	S	S	S
SAMN03647450	S	S	S	S
SAMN03647451	R	S	S	S
SAMN03647452	S	S	S	S
SAMN03647453	S	S	S	S
SAMN03647454	S	S	S	S
SAMN03647455	R	R	S	S
SAMN03647456	R	S	S	S
SAMN03647457	S	S	S	S
SAMN03647458	S	S	S	S
SAMN03647459	S	S	S	S
SAMN03647460	S	S	S	S
SAMN03647461	R	S	S	R
SAMN03647462	R	S	S	S
SAMN03647463	R	S	S	S
SAMN03647464	S	S	S	S
SAMN03647465	S	S	S	S
SAMN03647466	S	S	S	S
SAMN03647467	R	R	S	R
SAMN03647468	R	S	S	S
SAMN03647469	S	S	S	S
SAMN03647470	S	S	S	S
SAMN03647471	S	S	S	S
SAMN03647472	S	S	S	S
SAMN03647473	R	S	S	S
SAMN03647474	R	R	S	S
SAMN03647475	S	S	S	S
SAMN03647476	S	S	S	S
SAMN03647477	S	S	S	S
SAMN03647478	S	S	S	S
SAMN03647479	R	R	R	R
SAMN03647480	S	S	S	S
SAMN03647481	S	S	S	S
SAMN03647482	S	S	S	S
SAMN03647483	S	S	S	S
SAMN03647484	S	S	S	S
SAMN03647485	R	S	S	S
SAMN03647486	S	S	S	S
SAMN03647487	S	S	S	S
SAMN03647488	S	S	S	S
SAMN03647489	S	S	S	S
SAMN03647490	S	S	S	S
SAMN03647491	S	S	S	S
SAMN03647492	S	S	S	S
SAMN03647493	R	S	S	S
SAMN03647494	R	S	S	S
SAMN03647495	S	S	S	S
SAMN03647496	R	S	S	S
SAMN03647497	R	R	S	S
SAMN03647498	R	S	S	S
SAMN03647499	S	S	S	S
SAMN03647500	S	S	S	S
SAMN03647501	S	S	S	S

SAMN03647502	S	S	S	S
SAMN03647503	S	S	S	S
SAMN03647504	R	R	S	R
SAMN03647505	R	S	S	S
SAMN03647506	S	S	S	S
SAMN03647507	S	S	S	S
SAMN03647508	R	R	R	S
SAMN03647509	S	S	S	S
SAMN03647510	S	S	S	S
SAMN03647511	S	S	S	S
SAMN03647512	S	S	S	S
SAMN03647513	R	S	S	S
SAMN03647514	R	S	S	S
SAMN03647515	R	S	S	S
SAMN03647516	R	S	S	S
SAMN03647517	R	S	S	S
SAMN03647518	S	S	S	S
SAMN03647519	S	S	S	S
SAMN03647520	S	S	S	S
SAMN03647521	S	S	S	S
SAMN03647522	R	R	S	S
SAMN03647523	R	S	S	S
SAMN03647524	R	S	S	S
SAMN03647525	R	R	R	R
SAMN03647526	R	S	S	S
SAMN03647527	S	S	S	U
SAMN03647528	S	S	S	S
SAMN03647529	S	S	S	S
SAMN03647530	S	S	S	S
SAMN03647531	S	S	S	S
SAMN03647532	R	S	S	S
SAMN03647533	S	S	S	S
SAMN03647534	S	S	S	S
SAMN03647535	S	S	S	S
SAMN03647536	S	S	S	S
SAMN03647537	S	S	S	S
SAMN03647538	S	S	S	S
SAMN03647539	S	S	S	S
SAMN03647540	R	R	S	S
SAMN03647541	R	R	S	S
SAMN03647542	R	S	S	S
SAMN03647543	R	S	S	S
SAMN03647544	S	S	S	S
SAMN03647545	S	S	S	S
SAMN03647546	S	S	S	S
SAMN03647547	R	S	S	S
SAMN03647548	S	S	S	S
SAMN03647549	R	R	S	R
SAMN03647550	R	S	S	S
SAMN03647551	S	S	S	S
SAMN03647552	S	S	S	S
SAMN03647553	S	S	S	S
SAMN03647554	S	S	S	S

SAMN03647555 S S S S
SAMN03647556 R S S S
SAMN03647557 S S S S
SAMN03647558 R R R R
SAMN03647559 R S S S
SAMN03647560 R R R
SAMN03647561 R R S S
SAMN03647562 S R R R
SAMN03647563 R R U S
SAMN03647564 R S S S
SAMN03647565 R R S R
SAMN03647566 R S S S
SAMN03647567 R S S S
SAMN03647568 R S S S
SAMN03647569 S S S
SAMN03647570 R S S S
SAMN03647571 S R S S
SAMN03647572 R S S S
SAMN03647573 R R R R
ERS389344 S S S R
ERS389345 S S S S
SAMN03647574 S S S S
ERS389347 S S S S
ERS389348 S S S S
ERS389349 S S S S
SAMN03647575 R R S S
SAMN03647576 S S S S
ERS389350 S S S S
SAMN03647577 S S S S
ERS389351 S S S S
ERS389352 S S S S
ERS389353 S S S S
ERS389354 R S S S
ERS389355 S S S S
ERS389356 S S S S
ERS389357 R S S S
ERS389358 S S S S
ERS389359 S S S S
ERS389360 S S S S
ERS389361 S S S S
ERS389362 S S S S
ERS389363 S S S S
ERS389364 S S S S
ERS389365 S S S S
ERS389366 S S S S
ERS389367 S S S S
SAMN03647578 S S S S
ERS389369 S S S S
SAMN03647579 S S S S
ERS389371 S S S S
ERS389372 S S S S
SAMN03647580 S S S S
ERS389373 S S S S

ERS389374	S	S	S	S
ERS389375	S	S	S	S
ERS389376	S	S	S	S
SAMN03647581	S	S	S	S
ERS389377	S	S	S	S
SAMN03647582	S	S	S	S
ERS389379	S	S	S	S
ERS389380	R	S	S	S
ERS389381	S	S	S	S
ERS389382	S	S	S	S
ERS389383	S	S	S	S
SAMN03647583	S	S	S	S
ERS389384	S	S	S	S
ERS389385	S	S	S	S
SAMN03647584	S	S	S	S
ERS389387	S	S	S	S
ERS389388	S	S	S	S
ERS389389	S	S	R	S
ERS389390	S	S	S	S
ERS389391	S	S	S	S
ERS389392	S	S	S	S
ERS389393	S	S	S	S
SAMN03647585	S	S	S	S
ERS389394	S	S	S	S
SAMN03647586	S	S	S	S
ERS389395	S	S	S	S
SAMN03647587	S	S	S	S
ERS389397	S	S	S	S
ERS389398	S	S	S	S
ERS389399	S	S	S	S
ERS389400	S	S	S	S
ERS389401	S	S	S	S
ERS389402	S	S	S	S
SAMN03647588	S	S	S	S
SAMN03647589	S	S	S	S
ERS389403	S	S	S	S
SAMN03647590	R	S	S	S
ERS389404	S	S	S	S
ERS389405	S	S	S	S
ERS389406	S	S	S	R
ERS389407	S	S	S	S
SAMN03647591	S	S	S	S
ERS389408	S	S	S	S
SAMN03647592	R	R	R	R
SAMN03647593	S	S	S	S
SAMN03647594	S	S	S	S
SAMN03647595	S	S	S	S
SAMN03647596	S	S	S	S
ERS389409	S	S	S	S
ERS389410	S	S	S	S
ERS389411	S	S	S	S
ERS389412	S	S	S	S
SAMN03647597	S	S	S	S

SAMN03647598	S	S	S	S
ERS389413	S	S	S	S
SAMN03647599	S	S	S	S
ERS389415	S	S	S	S
ERS389416	S	S	S	S
ERS389417	S	S	S	S
ERS389418	S	S	S	R
ERS389419	S	S	S	S
ERS389420	S	S	S	S
SAMN03647600	S	S	S	S
ERS389421	S	S	S	S
SAMN03647601	S	S	S	S
ERS389422	S	S	S	S
ERS389423	S	S	S	S
ERS389424	S	S	S	S
ERS389425	S	S	S	S
SAMN03647602	R	R	R	S
SAMN03647603	S	S	S	S
ERS389426	S	S	S	S
SAMN03647604	S	S	S	S
ERS389427	S	R	S	S
ERS389428	S	S	S	S
SAMN03647605	R	R	S	R
ERS389429	S	S	S	S
ERS389430	S	S	S	S
ERS389431	S	S	S	S
ERS389432	S	S	S	S
ERS389433	S	S	S	S
ERS389434	S	S	S	S
SAMN03647606	S	S	S	S
ERS389435	S	S	S	S
ERS389436	S	S	S	S
SAMN03647607	S	S	S	S
ERS389437	S	S	S	S
ERS389438	S	S	S	S
SAMN03647608	S	S	S	S
SAMN03647609	S	S	S	S
ERS389439	S	S	S	S
SAMN03647610	S	S	S	S
SAMN03647611	S	S	S	S
SAMN03647612	S	S	S	S
ERS389441	S	S	S	S
SAMN03647613	S	S	S	S
SAMN03647614	S	S	S	S
SAMN03647615	S	S	S	S
ERS389445	S	S	S	S
ERS389447	S	S	S	S
ERS389448	S	S	S	S
SAMN03647616	S	S	S	S
SAMN03647617	S	S	S	S
ERS389449	S	S	S	S
SAMN03647618	S	S	S	S
SAMN03647619	R	S	S	S

ERS389450 S S S S
SAMN03647620 S S S S
ERS389451 S S S S
ERS389452 S S S S
SAMN03647621 S S S S
SAMN03647622 S S S S
ERS389455 S S S S
ERS389456 S S S S
SAMN03647623 S S S S
ERS389458 S S S S
ERS389462 R S S R
ERS389463 S S S S
SAMN03647624 S S S S
ERS389464 S S S S
ERS389465 S S S S
ERS389467 S S S S
ERS389468 S S S S
SAMN03647625 S S S S
SAMN03647626 R S S S
ERS389469 S S S S
ERS389470 S S S S
SAMN03647627 S S S S
ERS389471 S S S S
SAMN03647628 S S S S
ERS389472 S S S S
ERS389473 S S S S
ERS389474 S S S S
SAMN03647629 S S S S
ERS389475 R R S
ERS389476 S S S S
ERS389477 S S S S
ERS389478 S S S S
ERS389479 S S S S
ERS389480 S S S S
ERS389481 S S S S
ERS389482 S S S R
ERS389483 S S S S
SAMN03647630 S S S S
ERS389484 S S S S
ERS389485 S S S S
ERS389486 S S S S
ERS389487 S S S S
SAMN03647631 S S S S
ERS389488 S S S S
ERS389489 S S S S
ERS389490 S S S S
SAMN03647632 R R R S
ERS389491 S S S S
ERS389492 S S S S
ERS389493 S S S S
SAMN03647633 S S S S
SAMN03647634 S S S S
ERS389494 S S S S

ERS389495	S	S	S	S
SAMN03647635	S	S	S	S
SAMN03647636	S	S	S	S
SAMN03647637	S	S	S	S
ERS389498	S	S	S	S
ERS389499	S	S	S	S
ERS389500	S	S	S	S
ERS389501	R	S	S	S
SAMN03647638	S	U	U	U
ERS389503	S	S	S	S
ERS389504	S	S	S	S
ERS389505	S	S	S	S
ERS389507	S	S	S	S
ERS389508	S	S	S	S
ERS389509	S	S	S	S
ERS389510	S	S	S	S
ERS389511	R	R	R	R
ERS389512	S	S	S	S
ERS389513	S	S	S	S
SAMN03647639	S	S	S	S
ERS389514	S	S	S	S
ERS389515	S	S	S	S
ERS389516	S	S	S	S
SAMN03647640	S	S	S	S
SAMN03647641	S	S	S	S
SAMN03647642	S	S	S	S
SAMN03647643	S	S	S	S
SAMN03647644	S	S	S	S
SAMN03647645	S	S	S	S
SAMN03647646	S	S	S	S
SAMN03647647	S	S	S	S
SAMN03647648	S	S	S	S
SAMN03647649	S	S	S	S
SAMN03647650	S	S	S	S
SAMN03647651	S	S	S	S
SAMN03647652	S	S	S	S
SAMN03647653	S	S	S	S
SAMN03647654	S	S	S	S
SAMN03647655	S	S	S	S
SAMN03647656	S	S	S	S
SAMN03647657	S	S	S	S
SAMN03647658	S	S	S	S
SAMN03647659	S	S	S	S
SAMN03647660	S	S	S	S
SAMN03647661	S	S	S	S
SAMN03647662	S	S	S	S
SAMN03647663	S	S	S	S
SAMN03647664	S	S	S	S
SAMN03647665	S	S	S	S
SAMN03647666	S	S	S	S
SAMN03647667	S	S	S	S
SAMN03647668	S	S	S	S
SAMN03647669	S	S	S	S

SAMN03647670	U	U	U	S
SAMN03647671	S	S	S	S
SAMN03647672	R	S	S	S
SAMN03647673	S	S	S	S
SAMN03647674	S	S	S	S
SAMN03647675	S	S	S	S
SAMN03647676	S	S	S	S
SAMN03647677	S	S	S	S
SAMN03647678	S	S	S	S
SAMN03647679	S	S	S	S
SAMN03647680	S	S	S	S
SAMN03647681	S	S	S	S
SAMN03647682	S	S	S	S
SAMN03647683	S	S	S	S
SAMN03647684	S	S	S	S
SAMN03647685	S	S	S	S
SAMN03647686	S	S	S	S
SAMN03647687	S	S	S	S
SAMN03647688	S	S	S	S
SAMN03647689	S	S	S	S
SAMN03647690	S	S	S	S
SAMN03647691	S	S	S	S
SAMN03647692	S	S	S	S
SAMN03647693	S	S	S	S
SAMN03647694	S	S	S	S
SAMN03647695	S	S	S	S
SAMN03647696	S	S	S	S
SAMN03647697	S	S	S	S
SAMN03647698	S	S	S	S
SAMN03647699	S	S	S	S
SAMN03647700	S	S	S	S
SAMN03647701	S	S	S	S
SAMN03647702	S	S	S	S
SAMN03647703	S	S	S	S
SAMN03647704	S	S	S	S
SAMN03647705	S	S	S	S
SAMN03647706	S	S	S	S
SAMN03647707	S	S	S	S
SAMN03647708	S	S	S	S
SAMN03647709	S	S	S	S
SAMN03647710	S	S	S	S
SAMN03647711	S	S	S	S
SAMN03647712	S	S	S	S
SAMN03647713	S	S	S	S
SAMN03647714	S	S	S	S
SAMN03647715	S	S	S	S
SAMN03647716	S	S	S	S
SAMN03647717	S	S	S	S
SAMN03647718	S	S	S	S
SAMN03647719	S	S	S	S
SAMN03647720	S	S	S	S
SAMN03647721	S	S	S	S
SAMN03647722	U	U	U	S

SAMN03647723	S	S	S	S
SAMN03647724	S	S	S	S
SAMN03647725	S	S	S	S
SAMN03647726	S	S	S	S
SAMN03647727	S	S	S	S
SAMN03647728	S	S	S	S
SAMN03647729	S	S	S	S
SAMN03647730	S	S	S	S
SAMN03647731	S	S	S	S
SAMN03647732	S	S	S	S
SAMN03647733	S	S	S	S
SAMN03647734	S	S	S	S
SAMN03647735	S	S	S	S
SAMN03647736	S	S	S	S
SAMN03647737	S	S	S	S
SAMN03647738	S	S	S	S
SAMN03647739	S	S	S	S
SAMN03647740	S	S	S	S
SAMN03647741	S	S	S	S
SAMN03647742	S	S	S	S
SAMN03647743	S	S	S	S
SAMN03647744	S	S	S	S
SAMN03647745	S	S	S	S
SAMN03647746	S	S	S	S
SAMN03647747	S	S	S	S
SAMN03647748	S	S	S	S
SAMN03647749	S	S	S	S
SAMN03647750	S	S	S	S
SAMN03647751	S	S	S	S
SAMN03647752	S	S	S	S
SAMN03647753	S	S	S	S
SAMN03647754	S	S	S	S
SAMN03647755	S	S	S	S
SAMN03647756	S	S	S	S
SAMN03647757	S	S	S	S
SAMN03647758	S	S	S	S
SAMN03647759	S	S	S	S
SAMN03647760	S	S	S	S
SAMN03647761	S	S	S	S
SAMN03647762	S	S	S	S
SAMN03647763	S	S	S	S
SAMN03647764	S	S	S	S
SAMN03647765	S	S	S	S
SAMN03647766	S	S	S	S
SAMN03647767	S	S	S	S
SAMN03647768	S	S	S	S
SAMN03647769	S	S	S	S
SAMN03647770	S	S	S	S
SAMN03647771	S	S	S	S
SAMN03647772	S	S	S	S
SAMN03647773	S	S	S	S
SAMN03647774	S	R	S	S
SAMN03647775	S	S	S	S

SAMN03647776	S	S	S	S
SAMN03647777	S	S	S	S
SAMN03647778	S	S	S	S
SAMN03647779	S	S	S	S
SAMN03647780	S	S	S	S
SAMN03647781	S	S	S	S
SAMN03647782	S	S	S	S
SAMN03647783	S	S	S	S
SAMN03647784	S	S	S	S
SAMN03647785	S	S	S	S
SAMN03647786	S	S	S	S
SAMN03647787	S	S	S	S
SAMN03647788	S	S	S	S
SAMN03647789	S	S	S	S
SAMN03647790	S	S	S	S
SAMN03647791	S	S	S	S
SAMN03647792	S	S	S	S
SAMN03647793	S	S	S	S
SAMN03647794	S	S	S	S
SAMN03647795	S	S	S	S
SAMN03647796	S	S	S	S
SAMN03647797	S	S	S	S
SAMN03647798	S	S	S	S
SAMN03647799	S	S	S	S
SAMN03647800	S	S	S	S
SAMN03647801	S	S	S	S
SAMN03647802	S	S	S	S
SAMN03647803	S	S	S	S
SAMN03647804	S	S	S	S
SAMN03647805	S	S	S	S
SAMN03647806	S	S	S	S
SAMN03647807	S	S	S	S
SAMN03647808	S	S	S	S
SAMN03647809	S	S	S	S
SAMN03647810	S	S	S	S
SAMN03647811	S	S	S	S
SAMN03647812	S	S	S	S
SAMN03647813	S	S	S	S
SAMN03647814	S	S	S	S
SAMN03647815	S	S	S	S
SAMN03647816	S	S	S	S
SAMN03647817	S	S	S	S
SAMN03647818	S	S	S	S
SAMN03647819	S	S	S	S
SAMN03647820	S	S	S	S
SAMN03647821	S	S	S	S
SAMN03647822	S	S	S	S
SAMN03647823	S	S	S	S
SAMN03647824	S	S	S	S
SAMN03647825	S	S	S	S
SAMN03647826	S	S	S	S
SAMN03647827	S	S	S	S
SAMN03647828	S	S	S	S

SAMN03647829	S	S	S	S
SAMN03647830	S	S	S	S
SAMN03647831	S	S	S	S
SAMN03647832	S	S	S	S
SAMN03647833	S	S	S	S
SAMN03647834	S	S	S	S
SAMN03647835	S	S	S	S
SAMN03647836	S	S	S	S
SAMN03647837	S	S	S	S
SAMN03647838	S	S	S	S
SAMN03647839	S	S	S	S
SAMN03647840	S	S	S	S
SAMN03647841	S	S	S	S
SAMN03647842	S	S	S	S
SAMN03647843	S	S	S	S
SAMN03647844	S	S	S	S
SAMN03647845	S	S	S	S
SAMN03647846	S	S	S	S
SAMN03647847	S	S	S	S
SAMN03647848	S	S	S	S
SAMN03647849	S	S	S	S
SAMN03647850	S	S	S	S
SAMN03647851	S	S	S	S
SAMN03647852	S	S	S	S
SAMN03647853	S	S	S	S
SAMN03647854	S	S	S	S
SAMN03647855	S	S	S	S
SAMN03647856	S	S	S	S
SAMN03647857	S	S	S	S
SAMN03647858	S	S	S	S
SAMN03647859	S	S	S	S
SAMN03647860	S	S	S	S
SAMN03647861	S	S	S	S
SAMN03647862	U	S	S	S
SAMN03647863	S	S	S	S
SAMN03647864	S	S	S	S
SAMN03647865	S	S	S	S
SAMN03647866	S	S	S	S
SAMN03647867	S	S	S	S
SAMN03647868	S	S	S	S
SAMN03647869	S	S	S	S
SAMN03647870	S	S	S	S
SAMN03647871	S	S	S	S
SAMN03647872	S	S	S	S
SAMN03647873	S	S	S	S
SAMN03647874	S	S	S	S
SAMN03647875	S	S	S	S
SAMN03647876	S	S	S	S
SAMN03647877	S	S	S	S
SAMN03647878	R	S	S	S
SAMN03647879	S	S	S	S
SAMN03647880	S	S	S	S
SAMN03647881	S	S	S	S

SAMN03647882	S	S	S	S
SAMN03647883	S	S	S	S
SAMN03647884	S	S	S	S
SAMN03647885	S	S	S	S
SAMN03647886	S	S	S	S
SAMN03647887	S	S	S	S
SAMN03647888	S	S	S	S
SAMN03647889	S	S	S	S
SAMN03647890	S	S	S	S
SAMN03647891	S	S	S	S
SAMN03647892	S	S	S	S
SAMN03647893	S	S	S	S
SAMN03647894	U	U	U	R
SAMN03647895	S	S	S	S
SAMN03647896	S	S	S	S
SAMN03647897	S	S	S	S
SAMN03647898	S	S	S	S
SAMN03647899	S	S	S	S
SAMN03647900	S	S	S	S
SAMN03647901	S	S	S	S
SAMN03647902	S	S	S	S
SAMN03647903	S	S	S	S
SAMN03647904	S	S	S	S
SAMN03647905	S	S	S	S
SAMN03647906	S	S	S	S
SAMN03647907	S	S	S	S
SAMN03647908	S	S	S	S
SAMN03647909	S	S	S	S
SAMN03647910	S	S	S	S
SAMN03647911	S	S	S	S
SAMN03647912	R	S	S	S
SAMN03647913	R	R	R	S
SAMN03647914	S	S	S	S
SAMN03647915	S	S	S	S
SAMN03647916	S	S	S	S
SAMN03647917	S	S	S	S
SAMN03647918	S	S	S	S
SAMN03647919	S	S	S	S
SAMN03647920	S	S	S	S
SAMN03647921	S	S	S	S
SAMN03647922	S	S	S	S
SAMN03647923	S	S	S	S
SAMN03647924	S	S	S	S
SAMN03647925	S	S	S	S
SAMN03647926	S	S	S	S
SAMN03647927	S	S	S	S
SAMN03647928	S	S	S	S
SAMN03647929	S	S	S	S
SAMN03647930	U	U	S	S
SAMN03647931	S	S	S	S
SAMN03647932	S	S	S	S
SAMN03647933	S	S	S	S
SAMN03647934	S	S	S	S

SAMN03647935	S	S	S	S
SAMN03647936	S	S	S	S
SAMN03647937	S	S	S	S
SAMN03647938	S	S	S	S
SAMN03647939	S	S	S	S
SAMN03647940	S	S	S	S
SAMN03647941	S	S	S	S
SAMN03647942	S	S	S	S
SAMN03647943	S	S	S	S
SAMN03647944	S	S	S	S
SAMN03647945	S	S	S	S
SAMN03647946	S	S	S	S
SAMN03647947	S	S	S	S
SAMN03647948	S	S	S	S
SAMN03647949	S	S	S	S
SAMN03647950	S	S	S	S
SAMN03647951	S	S	S	S
SAMN03647952	S	S	S	S
SAMN03647953	S	S	S	S
SAMN03647954	S	S	S	S
SAMN03647955	S	S	S	S
SAMN03647956	S	S	S	S
SAMN03647957	S	S	S	S
SAMN03647958	S	S	S	S
SAMN03647959	S	S	S	S
SAMN03647960	S	S	S	S
SAMN03647961	S	S	S	S
SAMN03647962	S	S	S	S
SAMN03647963	S	S	S	S
SAMN03647964	S	S	S	S
SAMN03647965	S	S	S	S
SAMN03647966	S	S	S	S
SAMN03647967	S	S	S	S
SAMN03647968	S	S	S	S
SAMN03647969	S	S	S	S
SAMN03647970	S	S	S	S
SAMN03647971	S	S	S	S
SAMN03647972	S	S	S	S
SAMN03647973	S	S	S	S
SAMN03647974	S	S	S	S
SAMN03647975	S	S	S	S
SAMN03647976	R	R	S	S
SAMN03647977	S	S	S	S
SAMN03647978	S	S	S	S
SAMN03647979	S	S	S	S
SAMN03647980	S	S	S	S
SAMN03647981	S	S	S	S
SAMN03647982	S	S	S	S
SAMN03647983	S	S	S	S
SAMN03647984	S	S	S	S
SAMN03647985	S	S	S	S
SAMN03647986	S	S	S	S
SAMN03647987	S	S	S	S

SAMN03647988	S	S	S	S
SAMN03647989	S	S	S	S
SAMN03647990	S	S	S	S
SAMN03647991	S	S	S	S
SAMN03647992	S	S	S	S
SAMN03647993	S	S	S	R
SAMN03647994	S	S	S	R
SAMN03647995	R	S	S	S
SAMN03647996	R	S	S	S
SAMN03647997	S	S	S	S
SAMN03647998	S	S	S	R
SAMN03647999	S	S	R	S
SAMN03648000	R	S	S	S
SAMN03648001	R	R	S	S
SAMN03648002	R	S	S	S
SAMN03648003	R	R	R	R
SAMN03648004	R	S	S	S
SAMN03648005	R	S	S	S
SAMN03648006	R	S	S	S
SAMN03648007	S	S	S	S
SAMN03648008	S	S	S	S
SAMN03648009	R	R	S	S
SAMN03648010	R	S	S	S
SAMN03648011	R	R	S	S
SAMN03648012	S	R	S	S
SAMN03648013	R	S	S	S
SAMN03648014	R	S	S	S
SAMN03648015	R	R	S	S
SAMN03648016	R	S	S	S
SAMN03648017	S	S	S	R
SAMN03648018	R	S	S	S
SAMN03648019	R	S	S	S
SAMN03648020	S	S	S	R
SAMN03648021	R	R	S	S
SAMN03648022	R	S	S	S
SAMN03648023	R	S	S	S
SAMN03648024	R	S	S	S
SAMN03648025	R	S	S	S
SAMN03648026	R	S	S	S
SAMN03648027	R	R	S	S
SAMN03648028	R	R	S	S
SAMN03648029	S	S	S	S
SAMN03648030	S	S	S	S
SAMN03648031	S	S	S	S
SAMN03648032	R	S	S	S
SAMN03648033	S	S	S	S
SAMN03648034	S	S	S	S
SAMN03648035	S	S	S	S
SAMN03648036	S	S	S	S
SAMN03648037	S	S	S	S
SAMN03648038	S	S	S	S
SAMN03648039	R	S	S	S
SAMN03648040	S	S	S	S

SAMN03648041	R	S	S	S
SAMN03648042	R	R	R	S
SAMN03648043	R	R	R	S
SAMN03648044	R	S	S	S
SAMN03648045	R	S	S	S
SAMN03648046	R	R	R	S
SAMN03648047	R	S	S	S
SAMN03648048	R	S	S	S
SAMN03648049	R	S	S	S
SAMN03648050	R	S	U	S
SAMN03648051	R	S	S	S
SAMN03648052	R	S	S	S
SAMN03648053	R	R	S	S
SAMN03648054	R	S	S	S
SAMN03648055	R	S	S	S
SAMN03648056	R	S	S	S
SAMN03648057	R	R	U	R
SAMN03648058	R	S	S	S
SAMN03648059	R	S	S	S
SAMN03648060	R	S	S	S
SAMN03648061	R	S	S	S
SAMN03648062	R	R	R	S
SAMN03648063	R	R	S	R
SAMN03648064	R	R	S	U
SAMN03648065	R	S	S	S
SAMN03648066	S	S	S	S
SAMN03648067	R	S	S	S
SAMN03648068	S	R	S	S
SAMN03648069	S	S	S	R
SAMN03648070	R	S	S	S
SAMN03648071	R	S	S	S
SAMN03648072	S	S	S	S
SAMN03648073	R	S	S	S
SAMN03648074	S	S	S	
SAMN03648075	R	S	S	S
SAMN03648076	R	S	S	S
SAMN03648077	R	R	S	S
SAMN03648078	R	S	S	S
SAMN03648079	R	R	S	S
SAMN03648080	R	S	S	U
SAMN03648081	R	S	S	S
SAMN03648082	R	R	R	R
SAMN03648083	S	S	S	
SAMN03648084	R	R	R	S
SAMN03648085	R	R	S	S
SAMN03648086	S	S	S	S
SAMN03648087	R	R	S	S
SAMN03648088	R	S	S	S
SAMN03648089	R	S	S	S
SAMN03648090	R	S	S	S
SAMN03648091	R	S	S	S
SAMN03648092	R	S	S	S
SAMN03648093	R	R	R	S

SAMN03648094	R	R	S	R
SAMN03648095	S	S	S	S
SAMN03648170	S	R	S	R
SAMN03648171	S	S	S	S
SAMN03648172	R	R	S	S
SAMN03648173	S	S	S	S
SAMN03648174	S	S	S	S
SAMN03648175	S	S	S	S
SAMN03648176	S	S	S	S
SAMN03648177	S	S	S	S
SAMN03648178	S	S	S	S
SAMN03648179	S	S	S	S
SAMN03648180	S	S	S	S
SAMN03648181	S	S	S	S
SAMN03648182	S	S	S	S
SAMN03648183	S	S	S	S
SAMN03648184	S	S	S	S
SAMN03648185	S	S	S	S
SAMN03648186	S	S	S	S
SAMN03648187	S	S	S	S
SAMN03648188	S	S	S	S
SAMN03648189	S	S	S	S
SAMN03648190	S	S	S	S
SAMN03648191	S	S	S	S
SAMN03648192	S	S	S	S
SAMN03648193	S	S	S	S
SAMN03648194	S	S	S	S
SAMN03648195	S	S	S	S
SAMN03648196	S	S	S	S
SAMN03648197	R	R	R	R
SAMN03648198	S	S	S	S
SAMN03648199	S	S	S	S
SAMN03648200	S	S	S	S
SAMN03648201	S	S	S	S
SAMN03648202	S	S	S	S
SAMN03648203	S	S	S	S
SAMN03648204	S	S	S	S
SAMN03648205	S	S	S	S
SAMN03648206	S	S	S	S
SAMN03648207	S	S	S	S
SAMN03648208	S	S	S	S
SAMN03648209	S	S	S	S
SAMN03648210	S	S	S	S
SAMN03648211	S	S	S	S
SAMN03648212	S	S	S	S
SAMN03648213	S	S	S	S
SAMN03648214	S	S	S	S
SAMN03648215	S	S	S	S
SAMN03648216	S	S	S	S
SAMN03648217	S	S	S	S
SAMN03648218	S	S	S	S
SAMN03648219	S	S	S	S
SAMN03648220	S	S	S	S

SAMN03648221	S	S	S	S
SAMN03648222	S	S	S	S
SAMN03648223	S	S	S	S
SAMN03648224	S	S	S	S
SAMN03648225	S	S	S	S
SAMN03648226	S	S	S	S
SAMN03648227	S	S	S	S
SAMN03648228	S	S	S	S
SAMN03648229	S	S	S	S
SAMN03648230	S	S	S	S
SAMN03648231	S	S	S	S
SAMN03648232	S	S	S	S
SAMN03648233	S	S	S	S
SAMN03648234	S	S	S	S
SAMN03648235	S	S	S	S
SAMN03648236	S	S	S	S
SAMN03648237	S	S	S	S
SAMN03648238	S	S	S	S
SAMN03648239	S	S	S	S
SAMN03648240	S	S	S	S
SAMN03648241	S	S	S	S
SAMN03648242	S	S	S	S
SAMN03648243	S	S	S	S
SAMN03648244	S	S	S	S
SAMN03648245	S	S	S	U
SAMN03648246	S	S	S	S
SAMN03648247	S	S	S	S
SAMN03648248	S	S	S	S
SAMN03648249	S	S	S	S
SAMN03648250	S	S	S	S
SAMN03648251	S	S	S	S
SAMN03648252	S	S	S	S
SAMN03648253	S	S	S	S
SAMN03648254	S	S	S	S
SAMN03648255	S	S	S	S
SAMN03648256	S	S	S	S
SAMN03648257	S	S	S	S
SAMN03648258	S	S	S	S
SAMN03648259	S	S	S	S
SAMN03648260	S	S	S	S
SAMN03648261	S	S	S	S
SAMN03648262	S	S	S	S
SAMN03648263	S	S	S	S
SAMN03648264	S	S	S	S
SAMN03648265	S	S	S	S
SAMN03648266	S	S	S	S
SAMN03648267	S	S	S	S
SAMN03648268	R	R	S	S
SAMN03648269	S	S	S	S
SAMN03648270	R	R	S	R
SAMN03648271	R	S	S	S
SAMN03648272	S	S	S	S
SAMN03648273	S	S	S	S

SAMN03648274	S	S	S	S
SAMN03648275	S	S	S	S
SAMN03648276	U	U	U	S
SAMN03648277	S	S	S	S
SAMN03648278	S	S	S	S
SAMN03648279	S	S	S	S
SAMN03648280	S	S	S	S
SAMN03648281	S	S	S	S
SAMN03648282	S	S	S	S
SAMN03648283	S	S	S	S
SAMN03648284	S	S	R	S
SAMN03648285	S	S	S	S
SAMN03648286	S	S	R	
SAMN03648287	S	S	S	S
SAMN03648288	S	S	S	S
SAMN03648289	S	S	S	S
SAMN03648290	S	S	S	S
SAMN03648291	S	S	S	S
SAMN03648292	S	S	S	S
SAMN03648293	S	S	S	S
SAMN03648294	S	S	S	S
SAMN03648295	S	S	S	S
SAMN03648296	S	S	S	S
SAMN03648297	S	S	S	S
SAMN03648298	S	S	S	S
SAMN03648299	S	S	S	S
SAMN03648300	R	R	U	R
SAMN03648301	S	S	S	S
SAMN03648302	R	R	R	R
SAMN03648303	S	S	S	S
SAMN03648304	S	S	S	S
SAMN03648305	S	S	S	S
SAMN03648306	S	S	S	S
SAMN03648307	S	S	S	S
SAMN03648308	S	S	S	S
SAMN03648309	S	S	S	S
ERS389518	S	S	S	S
ERS389519	S	S	S	S
ERS389520	S	S	S	S
SAMN03648310	R	U	S	S
ERS389522	S	S	S	S
ERS389523	S	S	S	S
ERS389524	S	S	S	S
ERS389525	S	S	S	S
ERS389527	S	S	S	S
ERS389528	S	S	S	S
ERS389529	S	S	S	S
ERS389530	S	S	S	S
SAMN03648311	S	S	S	S
SAMN03648312	R	S	S	S
ERS389535	S	S	S	S
ERS389536	S	S	S	S
ERS389538	S	S	S	S

SAMN03648313	S	U	U	U
ERS389539	S	S	S	S
ERS389540	S	U	U	U
ERS389541	S	S	S	S
ERS389542	R	S	S	S
ERS389543	S	S	S	S
ERS389544	S	S	S	S
SAMN03648314	S	S	S	S
ERS389545	S	S	S	S
ERS389548	S	S	S	S
ERS389549	S	S	S	S
ERS389550	S	S	S	S
ERS389551	S	U	U	U
ERS389552	S	S	S	S
ERS389553	R	U	U	U
ERS389554	S	S	S	S
SAMN03648315	S	S	S	S
SAMN03648316	S	S	S	S
SAMN03648317	S	S	S	S
SAMN03648318	S	S	S	S
SAMN03648319	S	S	S	S
SAMN03648320	S	S	S	S
SAMN03648321	S	S	S	S
SAMN03648322	S	S	S	S
SAMN03648323	S	S	S	S
SAMN03648324	R	S	S	S
SAMN03648325	S	S	S	S
SAMN03648326	S	S	S	S
SAMN03648327	S	S	S	S
SAMN03648328	S	S	S	S
SAMN03648329	S	S	S	S
SAMN03648330	S	S	S	S
SAMN03648331	S	S	S	S
ERS389558	S	S	S	S
ERS389559	S	S	S	S
ERS389562	S	S	S	S
ERS389563	S	S	S	S
ERS389564	R	S	S	S
ERS389565	S	S	S	S
ERS389568	S	S	S	S
ERS389569	S	S	S	S
SAMN03648332	S	S	S	S
ERS389575	S	S	S	S
ERS389579	S	S	S	S
ERS389580	S	S	S	S
ERS389581	S	S	S	S
SAMN03648333	S	S	S	S
SAMN03648334	S	S	S	U
SAMN03648335	S	S	S	S
SAMN03648336	S	S	S	S
SAMN03648337	S	S	S	S
SAMN03648338	S	S	S	S
SAMN03648339	S	S	S	S

SAMN03648340	S	S	S	S
SAMN03648341	S	S	S	S
SAMN03648342	S	S	S	S
SAMN03648343	S	S	S	S
SAMN03648344	S	S	S	S
SAMN03648345	S	S	S	S
SAMN03648346	S	S	S	S
SAMN03648347	S	S	S	S
SAMN03648348	S	S	S	S
SAMN03648349	S	S	S	S
SAMN03648350	S	S	S	S
SAMN03648351	S	S	S	S
SAMN03648352	S	S	S	S
SAMN03648353	S	S	S	S
SAMN03648354	S	S	S	S
SAMN03648355	S	S	S	S
SAMN03648356	S	S	S	S
SAMN03648357	S	S	S	S
SAMN03648358	S	S	S	S
SAMN03648359	S	S	S	S
SAMN03648360	S	S	S	S
SAMN03648361	S	S	S	S
SAMN03648362	S	S	S	S
SAMN03648363	S	S	S	S
SAMN03648364	S	S	S	S
SAMN03648365	S	S	S	S
SAMN03648366	S	S	S	S
SAMN03648367	S	S	S	S
SAMN03648368	S	S	S	S
SAMN03648369	S	S	S	S
SAMN03648370	S	S	S	S
SAMN03648371	S	S	S	S
SAMN03648372	S	S	S	S
SAMN03648373	S	S	S	S
SAMN03648374	S	S	S	S
SAMN03648375	S	S	S	S
SAMN03648376	S	S	S	S
SAMN03648377	S	S	S	S
SAMN03648378	S	S	S	S
SAMN03648379	S	S	S	S
SAMN03648380	S	S	S	S
SAMN03648381	S	S	S	S
SAMN03648382	S	S	S	S
SAMN03648383	S	S	S	S
SAMN03648384	S	S	S	S
SAMN03648385	S	S	S	S
SAMN03648386	S	S	S	S
SAMN03648387	S	S	S	S
SAMN03648388	S	S	S	S
SAMN03648389	S	S	S	S
SAMN03648390	S	S	S	S
SAMN03648391	S	S	S	S
SAMN03648392	S	S	S	S

SAMN03648393	S	S	S	S
SAMN03648394	S	S	S	S
SAMN03648395	S	S	S	S
SAMN03648396	S	S	S	S
SAMN03648397	S	S	S	S
SAMN03648398	S	S	S	S
SAMN03648399	S	S	S	S
SAMN03648400	S	S	S	S
SAMN03648401	S	S	S	S
SAMN03648402	S	S	S	S
SAMN03648403	S	S	S	S
SAMN03648404	S	S	S	S
SAMN03648405	S	S	S	S
SAMN03648406	S	S	S	S
SAMN03648407	S	S	S	S
SAMN03648408	S	S	S	S
SAMN03648409	S	S	S	S
SAMN03648410	S	S	S	S
SAMN03648411	S	S	S	S
SAMN03648412	S	S	S	S
SAMN03648413	S	S	S	S
SAMN03648414	S	S	S	S
SAMN03648415	S	S	S	S
SAMN03648416	S	S	S	S
SAMN03648417	S	S	S	S
SAMN03648418	S	S	S	S
SAMN03648419	S	S	S	S
SAMN03648420	S	S	S	S
SAMN03648421	S	S	S	S
SAMN03648422	S	S	S	S
SAMN03648423	S	S	S	S
SAMN03648424	S	S	S	S
SAMN03648425	S	S	S	S
SAMN03648426	S	S	S	S
SAMN03648427	S	S	S	S
SAMN03648428	S	S	S	S
SAMN03648429	S	S	S	S
SAMN03648430	S	S	S	S
SAMN03648431	S	S	S	S
SAMN03648432	S	S	S	S
SAMN03648433	S	S	S	S
SAMN03648434	S	S	S	S
SAMN03648435	S	S	S	S
SAMN03648436	S	S	S	S
SAMN03648437	S	S	S	S
SAMN03648438	S	S	S	S
SAMN03648439	S	S	S	S
SAMN03648440	S	S	S	S
SAMN03648441	S	S	S	S
SAMN03648442	S	S	S	S
SAMN03648443	S	S	S	S
SAMN03648444	S	S	S	S
SAMN03648445	S	S	S	S

SAMN03648446	S	S	S	S
SAMN03648447	S	S	S	S
SAMN03648448	S	S	S	S
SAMN03648449	S	S	S	S
SAMN03648450	S	S	S	S
SAMN03648451	S	S	S	S
SAMN03648452	S	S	S	S
SAMN03648453	S	S	S	S
SAMN03648454	S	S	S	S
SAMN03648455	S	S	S	S
SAMN03648456	S	S	S	S
SAMN03648457	S	S	S	S
SAMN03648458	S	S	S	S
SAMN03648459	S	S	S	S
SAMN03648460	S	S	S	S
SAMN03648461	S	S	S	S
SAMN03648462	S	S	S	S
SAMN03648463	S	S	S	S
SAMN03648464	S	S	S	S
SAMN03648465	S	S	S	S
SAMN03648466	S	S	S	S
SAMN03648467	S	S	S	S
SAMN03648468	S	S	S	S
SAMN03648469	S	S	S	S
SAMN03648470	S	S	S	S
SAMN03648471	S	S	S	S
SAMN03648472	S	S	S	S
SAMN03648473	S	S	S	S
SAMN03648474	S	S	S	S
SAMN03648475	S	S	S	S
SAMN03648476	S	S	S	S
SAMN03648477	S	S	S	S
SAMN03648478	S	S	S	S
SAMN03648479	S	S	S	S
SAMN03648480	S	S	S	S
SAMN03648481	S	S	S	S
SAMN03648482	S	S	S	U
SAMN03648483	S	S	S	S
SAMN03648484	S	S	S	S
SAMN03648485	S	S	S	S
SAMN03648486	S	S	S	S
SAMN03648487	R	S	S	S
SAMN03648488	S	S	S	S
SAMN03648489	S	S	S	S
SAMN03648490	S	S	S	S
SAMN03648491	S	S	S	S
SAMN03648492	S	S	S	S
SAMN03648493	S	S	S	S
SAMN03648494	S	S	S	S
SAMN03648495	S	S	S	S
SAMN03648496	S	S	S	S
SAMN03648497	S	S	S	S
SAMN03648498	S	S	S	S

SAMN03648499	S	S	S	S
SAMN03648500	S	S	S	S
SAMN03648501	S	S	S	S
SAMN03648502	S	S	S	S
SAMN03648503	S	S	S	S
SAMN03648504	S	S	S	S
SAMN03648505	R	S	S	S
SAMN03648506	R	S	S	S
SAMN03648507	S	S	S	S
SAMN03648508	S	R	R	R
SAMN03648509	S	S	S	R
SAMN03648510	R	S	S	S
SAMN03648511	S	S	S	S
SAMN03648512	S	S	S	S
SAMN03648513	S	S	S	S
SAMN03648514	S	S	S	S
SAMN03648515	S	S	S	S
SAMN03648516	S	S	S	S
SAMN03648517	S	S	S	S
SAMN03648518	S	S	S	U
SAMN03648519	S	S	S	S
SAMN03648520	S	S	S	S
SAMN03648521	S	S	S	S
SAMN03648522	S	S	S	S
SAMN03648523	S	S	S	S
SAMN03648524	S	S	S	S
ERS389586	S	S	S	S
SAMN03648525	S	S	S	S
ERS389587	S	S	S	S
ERS389588	S	S	S	S
ERS389589	S	S	S	S
ERS389590	S	U	U	U
ERS389591	S	S	S	S
ERS389592	S	S	S	S
ERR2515372	S	S	S	S
ERR2515493	S	S	S	S
ERR2515455	S	S	S	R
ERR2515111	S	S	S	S
ERR2515819	S	S	S	S
ERR2516028	S	S	S	S
ERR2515626	S	S	S	S
ERR2515790	S	S	S	S
ERR2515437	S	S	S	S
ERR2515268	S	S	S	S
ERR2515402	S	S	S	S
ERR2515980	S	S	S	S
ERR2515138	S	S	S	S
ERR2515547	S	S	S	S
ERR2515770	S	S	S	S
ERR2515293	R	R	R	R
ERR2515740	R	R	R	R
ERR2516085	S	S	S	S
ERR2515172	S	S	S	S

ERR2515467	S	S	S	S
ERR2516059	S	S	S	U
ERR2515243	S	S	S	S
ERR2516037	R	S	S	S
ERR2515469	S	S	S	S
ERR2515290	S	S	S	S
ERR2515562	S	S	S	S
ERR2515901	S	S	S	S
ERR2515702	S	S	S	S
ERR2515192	S	S	S	S
ERR2515693	S	S	S	S
ERR2515114	R	S	S	S
ERR2515118	S	S	S	S
ERR2515190	S	S	S	S
ERR2515954	S	S	S	S
ERR2515809	S	S	S	S
ERR2515727	S	R	S	S
ERR2515349	S	S	S	S
ERR2515377	S	S	S	S
ERR2515353	R	R	R	S
ERR2515811	S	S	S	S
ERR2515124	S	S	S	S
ERR2515818	S	S	S	S
ERR2515652	S	S	S	S
ERR2515433	S	S	S	S
ERR2515751	R	S	S	R
ERR2515673	S	S	S	S
ERR2515116	S	S	S	S
ERR2515622	R	R	R	R
ERR2515302	S	S	S	S
ERR2515375	R	S	S	S
ERR2515845	R	S	S	S
ERR2515537	S	S	S	S
ERR2516048	S	S	S	S
ERR2515580	S	S	S	S
ERR2515656	S	S	S	S
ERR2515291	S	S	S	S
ERR2515368	S	S	S	S
ERR2515446	S	S	S	S
ERR2515129	S	S	S	S
ERR2515191	S	S	S	U
ERR2515226	S	S	S	S
ERR2515646	S	S	S	S
ERR2515156	S	S	S	S
ERR2515966	S	S	S	S
ERR2515462	S	S	S	S
ERR2515689	S	S	S	S
ERR2515540	R	S	S	S
ERR2515464	S	S	S	S
ERR2515270	S	S	S	S
ERR2515779	S	S	S	S
ERR2515435	S	S	S	S
ERR2515553	S	S	S	S

ERR2516076	S	S	S	S
ERR2515237	S	S	S	S
ERR2515522	S	S	S	S
ERR2515916	S	S	S	S
ERR2515314	S	S	S	S
ERR2515257	S	S	S	S
ERR2515572	S	S	S	S
ERR2515461	S	S	S	R
ERR2515964	S	S	S	S
ERR2516081	S	S	S	S
ERR2515653	S	S	S	S
ERR2515483	S	S	S	S
ERR2515134	S	S	S	S
ERR2516088	S	S	S	S
ERR2515828	S	S	S	S
ERR2515391	S	S	S	S
ERR2515905	S	S	S	S
ERR2515278	S	S	S	S
ERR2515841	S	S	S	S
ERR2516056	S	S	S	S
ERR2515617	S	S	S	S
ERR2515742	S	S	S	S
ERR2515298	S	S	S	S
ERR2515596	S	S	S	S
ERR2515128	S	S	S	S
ERR2516040	S	S	S	S
ERR2515803	S	S	S	S
ERR2515866	S	S	S	S
ERR2515821	S	S	S	S
ERR2515320	S	S	S	S
ERR2515853	S	S	S	S
ERR2515681	S	S	S	S
ERR2516094	S	S	S	S
ERR2515550	S	S	S	S
ERR2515869	S	S	S	S
ERR2515487	S	S	R	S
ERR2515453	S	S	S	S
ERR2515608	S	S	S	S
ERR2515627	S	S	S	S
ERR2515793	R	S	S	S
ERR2515722	S	S	S	S
ERR2515706	S	S	S	S
ERR2515829	S	S	S	S
ERR2515885	S	S	S	S
ERR2515827	S	S	S	S
ERR2515254	S	S	S	S
ERR2515312	S	S	S	S
ERR2515160	S	S	S	R
ERR2516062	S	S	S	S
ERR2515105	S	S	S	S
ERR2516026	S	S	S	R
ERR2515148	S	S	S	S
ERR2515651	S	S	S	S

ERR2515929	S	S	S	S
ERR2515184	S	S	S	S
ERR2515233	S	S	S	S
ERR2515201	S	S	S	S
ERR2515729	S	S	S	S
ERR2515161	S	S	S	S
ERR2515454	S	S	S	S
ERR2515723	S	S	S	S
ERR2515842	R	S	S	S
ERR2515778	S	S	S	S
ERR2515338	S	S	S	S
ERR2515545	S	S	S	S
ERR2515515	S	S	S	S
ERR2515198	S	S	S	S
ERR2515188	S	S	S	S
ERR2515783	S	S	S	S
ERR2515972	S	S	S	S
ERR2515527	S	S	S	S
ERR2515945	S	S	R	S
ERR2516080	S	S	S	S
ERR2515902	S	S	S	S
ERR2515125	S	S	S	S
ERR2515503	S	S	S	S
ERR2515289	S	S	S	S
ERR2515194	S	S	S	S
ERR2515256	S	S	S	S
ERR2515261	R	S	S	R
ERR2515316	S	S	S	S
ERR2515245	S	R	R	S
ERR2515715	S	S	S	S
ERR2516050	R	S	S	R
ERR2515154	S	S	S	S
ERR2515317	S	S	S	S
ERR2515642	S	S	S	S
ERR2515558	S	S	S	S
ERR2515181	S	S	S	S
ERR2515731	S	S	S	R
ERR2515395	S	S	S	S
ERR2515351	S	S	S	S
ERR2515752	S	S	S	S
ERR2515891	S	S	S	S
ERR2515838	S	S	S	S
ERR2515899	S	S	S	S
ERR2515951	S	S	S	S
ERR2515303	S	S	S	S
ERR2515858	R	S	S	S
ERR2515220	S	S	S	S
ERR2516082	S	S	S	S
ERR2515932	S	S	S	S
ERR2515714	R	S	S	S
ERR2515187	S	S	S	S
ERR2515985	S	S	S	S
ERR2515831	S	S	S	S

ERR2515504	R	S	S	S
ERR2515582	S	S	S	S
ERR2515381	S	S	S	S
ERR2516073	R	S	S	S
ERR2515276	S	S	S	S
ERR2515780	S	S	S	S
ERR2515468	S	S	S	S
ERR2515677	R	R	S	S
ERR2515893	R	S	S	S
ERR2515862	S	S	S	S
ERR2515141	S	S	S	S
ERR2515767	S	S	S	S
ERR2515434	S	S	S	S
ERR2515429	S	S	S	S
ERR2515255	R	R	S	S
ERR2516052	S	S	S	S
ERR2515264	R	R	R	R
ERR2515380	S	S	S	S
ERR2515379	S	S	S	S
ERR2516074	S	R	S	S
ERR2515730	S	S	S	S
ERR2515168	S	S	S	S
ERR2515638	S	S	S	S
ERR2515480	S	S	S	S
ERR2515459	S	S	S	S
ERR2515430	S	S	S	U
ERR2515796	S	S	S	S
ERR2515137	S	S	S	S
ERR2515843	S	S	S	S
ERR2515296	S	S	S	U
ERR2516042	S	S	S	S
ERR2515456	S	S	S	S
ERR2515792	S	S	S	S
ERR2515176	S	S	S	S
ERR2515120	S	S	S	S
ERR2515339	S	S	S	S
ERR2515668	S	S	S	S
ERR2515563	S	S	S	S
ERR2515683	S	S	S	S
ERR2515207	S	S	S	S
ERR2515215	S	S	S	S
ERR2515183	S	S	S	S
ERR2515378	S	S	S	S
ERR2516032	S	S	S	S
ERR2515421	S	S	S	S
ERR2515769	S	S	S	S
ERR2515813	S	S	S	S
ERR2515388	S	S	S	S
ERR2515680	S	S	S	S
ERR2515359	S	S	S	S
ERR2515439	R	S	S	S
ERR2515688	S	S	S	S
ERR2515551	S	S	S	S

ERR2515336	S	S	S	S
ERR2515692	S	S	S	S
ERR2515574	S	S	S	S
ERR2515185	R	S	S	R
ERR2515665	S	S	S	S
ERR2515127	S	S	S	S
ERR2515889	S	S	S	S
ERR2515366	S	S	S	S
ERR2515392	S	S	S	S
ERR2515299	S	S	S	S
ERR2515307	S	S	S	S
ERR2515816	S	S	S	S
ERR2515984	R	S	S	S
ERR2515404	S	S	S	S
ERR2515973	S	S	S	S
ERR2515536	S	S	S	S
ERR2515492	S	S	S	S
ERR2515968	S	S	S	S
ERR2515393	S	S	S	S
ERR2515561	S	S	S	S
ERR2516071	S	S	S	S
ERR2515593	S	S	S	S
ERR2515707	S	S	S	S
ERR2515620	S	S	S	S
ERR2515496	S	S	S	S
ERR2515394	S	S	S	S
ERR2515516	S	S	S	S
ERR2515621	S	S	U	S
ERR2515981	S	S	S	S
ERR2515746	S	S	S	U
ERR2515913	S	S	S	S
ERR2515450	S	S	S	S
ERR2515244	S	S	S	S
ERR2515259	S	S	S	S
ERR2515426	S	S	R	S
ERR2515370	S	S	R	S
ERR2515852	R	S	S	S
ERR2515736	S	S	S	S
SAMN03648526	S	S	S	S
ERR2515535	S	S	S	S
ERR2515196	S	S	S	S
ERR2515340	S	S	S	S
ERR2515517	S	S	S	S
ERR2515424	S	S	S	S
ERR2515914	S	S	S	S
ERR2515659	S	S	S	S
ERR2515251	S	S	S	S
ERR2516039	S	S	S	S
ERR2515775	S	S	S	S
ERR2515357	S	S	S	S
ERR2515956	R	R	S	S
ERR2515241	S	S	S	S
ERR2515883	S	S	S	S

ERR2515428	S	S	S	S
ERR2515400	R	S	R	R
ERR2515471	S	S	S	S
ERR2515785	S	S	S	S
ERR2515476	S	S	S	S
ERR2515773	S	S	S	S
ERR2515616	S	S	S	S
ERR2516084	S	S	S	S
ERR2515564	S	S	S	S
ERR2516000	S	S	S	S
ERR2515555	S	S	S	S
SAMN03648527	S	S	S	S
SAMN03648528	S	S	S	S
ERR2509680	S	S	S	S
SAMN03648529	S	S	S	S
ERR2509681	S	S	S	S
ERR2515193	S	S	S	S
ERR2515367	S	S	S	S
ERR2515624	S	S	S	S
ERR2515171	S	S	S	S
ERR2515687	S	S	S	R
ERR2515164	S	S	S	S
ERR2515991	S	S	S	S
ERR2515409	R	R	S	R
ERR2515478	S	S	S	S
ERR2515696	S	S	S	S
ERR2515262	R	R	R	S
ERR2515836	S	S	S	S
ERR2515204	S	S	S	S
ERR2515162	S	S	S	S
ERR2515804	S	S	S	S
ERR2516152	R	R	S	S
ERR2516124	S	S	S	S
ERR2516160	R	R	S	S
ERR2516145	S	S	S	S
ERR2516143	R	R	S	S
ERR2516111	S	R	S	S
ERR2516125	R	R	S	S
ERR2516138	R	R	R	R
ERR2516147	S	S	S	S
ERR2516158	R	S	S	S
ERR2516135	S	S	S	R
ERR2516136	R	R	R	R
ERR2516161	R	R	R	R
ERR2516151	R	S	R	S
ERR2516122	S	S	S	S
ERR2516132	R	S	S	S
ERR2516103	S	S	S	S
ERR2516155	S	S	S	S
ERR2516123	S	R	S	S
ERR2516153	R	S	S	S
ERR2516141	S	S	S	U
ERR2516144	S	S	S	S

ERR2516121	S	S	S	S
ERR2516117	S	S	S	S
ERR2516102	S	S	S	S
ERR2516105	S	S	S	S
ERR2516126	S	S	S	S
ERR2516110	S	S	S	S
ERR2516130	S	S	S	S
ERR2516164	S	S	S	S
ERR2516146	S	S	S	U
ERR2516148	S	S	S	S
ERR2516133	S	S	S	S
ERR2516142	S	S	S	S
ERR2516157	S	S	S	S
ERR2516119	S	S	S	S
ERR2516140	S	S	S	S
ERR2516163	S	S	S	S
ERR2516159	S	S	S	S
ERR2516113	S	S	S	S
ERR2516128	S	S	S	S
ERR2516112	S	S	S	S
ERR2516137	S	S	S	S
ERR2516150	R	S	S	S
ERR2516118	S	S	S	S
SAMN03648530	S	S	S	S
ERR2516139	S	S	S	S
ERR2516109	S	S	S	S
ERR2516149	S	S	S	S
ERR2516165	S	S	S	S
ERR2516116	S	S	S	S
ERR2516115	S	S	S	S
ERR2516114	S	S	S	S
ERR2516104	S	S	S	S
ERR2516120	S	S	S	U
ERR2516129	R	R	R	S
ERR2516131	S	S	S	S
ERR2516156	S	S	S	S
ERR2516106	S	S	S	S
ERR2516107	S	S	S	S
ERR2516162	S	S	S	S
ERR2516127	S	S	S	S
ERR2516134	S	S	S	S
SAMN03648531	S	S	S	S
SAMN03648532	S	S	S	S
ERR2516108	S	S	S	S
ERR2516154	S	S	S	S
ERR2515363	S	S	S	S
ERR2516051	S	S	S	S
ERR2515907	S	R	S	S
ERR2515822	S	S	S	S
ERR2515718	S	S	S	S
ERR2515865	S	S	S	S
ERR2515280	S	S	S	S
ERR2515895	S	S	S	S

ERR2515413	S	S	S	S
ERR2515618	S	S	S	S
ERR2515494	S	S	S	S
ERR2515820	S	S	S	S
ERR2515306	S	S	S	S
ERR2515904	S	S	S	S
ERR2515928	S	S	S	S
ERR2515343	S	S	S	S
ERR2517022	R	R	S	R
ERR2516989	R	R	U	U
ERR2517021	R	R	S	S
ERR2516751	R	R	U	S
ERR2517068	R	R	S	S
ERR2517120	R	R	S	S
ERR2517097	R	R	U	R
ERR2516763	R	R	U	S
ERR2516743	R	R	U	U
ERR2516935	R	R	R	R
ERR2516990	R	R	U	R
ERR2516808	R	R	S	S
ERR2517017	R	R	U	S
ERR2516790	R	R	U	U
ERR2516881	R	R	U	S
ERR2516929	R	R	R	R
ERR2517066	R	R	R	S
ERR2516901	R	R	U	R
ERR2516752	R	R	U	R
ERR2517062	R	R	U	S
ERR2516775	R	R	U	R
ERR2517077	R	R	U	U
ERR2516861	R	R	U	S
ERR2516945	R	R	U	S
ERR2516848	R	R	U	S
ERR2516917	R	R	U	R
ERR2516773	R	R	U	U
ERR2517118	R	R	R	R
ERR2516758	R	R	S	S
ERR2517038	R	R	U	S
ERR2516883	R	R	U	U
ERR2516832	R	R	S	S
ERR2516833	R	R	U	S
ERR2516845	R	R	S	R
ERR2517063	R	R	S	R
ERR2516804	R	R	S	S
ERR2516754	R	R	S	S
ERR2516844	R	R	R	S
ERR2517019	R	R	R	R
ERR2516912	R	R	S	S
ERR2517089	R	R	R	S
ERR2516978	R	R	U	R
ERR2517078	R	R	S	S
ERR2516810	R	R	S	R
ERR2516939	R	R	R	R

ERR2516891	R	R	R	R
ERR2517045	R	R	U	R
ERR2516966	S	S	S	S
ERR2516849	R	R	U	S
ERR2516968	R	R	R	R
ERR2516982	R	R	R	R
ERR2517044	R	R	S	U
ERR2517084	R	R	U	S
ERR2516974	R	R	U	S
ERR2517101	R	R	U	S
ERR2516777	R	R	U	R
ERR2517074	R	R	S	R
ERR2516862	R	R	U	S
ERR2516952	S	S	S	S
ERR2516780	R	R	U	S
SAMN03648533	S	S	S	S
ERR2516957	S	U	S	S
ERR2516769	R	R	U	R
ERR2517029	R	R	S	S
ERR2516828	U	R	R	S
ERR2516867	R	R	U	R
ERR2516805	R	R	R	R
ERR2517093	R	R	U	S
ERR2516794	R	U	S	S
ERR2517110	R	R	S	S
ERR2516960	R	R	R	R
ERR2517079	R	R	S	S
ERR2516829	R	R	R	R
ERR2517061	S	S	S	S
ERR2516843	R	R	R	S
ERR2517010	R	R	U	R
ERR2517030	S	S	S	S
ERR2517086	R	R	U	U
ERR2517067	R	R	U	R
ERR2517056	R	R	U	S
ERR2516802	R	R	U	R
ERR2516847	R	R	U	S
ERR2516913	R	R	S	S
ERR2516949	R	R	U	R
ERR2516850	R	R	U	U
ERR2517057	R	R	U	S
ERR2516834	R	R	S	S
ERR2516823	R	R	S	S
ERR2516899	R	R	R	R
ERR2516796	S	S	S	S
ERR2516814	R	R	S	S
ERR2516782	R	R	S	S
ERR2517004	R	R	R	S
ERR2516930	S	S	S	S
ERR2516817	R	R	S	R
ERR2516915	R	R	U	U
ERR2517103	R	R	S	S
ERR2516824	R	R	R	R

ERR2517085	R	R	S	S
ERR2517109	S	S	S	S
ERR2516909	R	R	S	S
ERR2516839	R	R	U	S
ERR2516764	R	R	U	S
ERR2516871	R	R	U	S
ERR2516880	R	R	U	R
ERR2517033	R	R	U	R
ERR2516994	R	R	R	R
ERR2516905	R	R	U	S
ERR2517042	R	R	S	S
ERR2517123	R	R	S	R
ERR2516852	R	R	U	U
ERR2517076	R	R	U	S
ERR2516886	R	R	R	S
ERR2516928	R	R	S	S
ERR2516911	R	R	R	R
ERR2517047	R	R	R	R
ERR2516811	R	R	R	S
ERR2517090	R	R	R	S
ERR2516981	R	R	R	S
ERR2516988	R	R	S	S
ERR2516742	R	R	R	S
ERR2516950	R	R	R	S
ERR2516941	R	R	S	S
ERR2517054	U	R	S	R
ERR2516979	R	R	U	R
ERR2516778	R	R	U	S
ERR2516942	R	R	U	S
ERR2516841	R	R	R	R
ERR2516894	R	R	U	S
ERR2516910	R	R	R	R
ERR2516898	R	R	R	R
ERR2517114	R	R	U	U
ERR2517125	R	R	U	R
ERR2516815	R	R	U	U
ERR2517112	R	R	U	R
ERR2517041	R	R	U	R
ERR2517082	R	R	R	S
ERR2516992	S	S	S	S
ERR2516767	R	R	R	S
ERR2516934	R	R	S	R
ERR2516946	R	R	U	S
ERR2517075	R	R	U	S
ERR2516784	R	R	R	R
ERR2516846	R	R	R	R
ERR2516781	R	R	R	S
ERR2516920	R	R	R	U
ERR2516937	R	R	S	S
ERR2516947	R	R	U	S
ERR2516801	R	R	R	R
ERR2517055	R	R	U	R
ERR2516933	R	R	S	S

ERR2516772	R	R	U	R
ERR2516984	R	R	U	S
ERR2517087	R	U	R	R
ERR2516756	R	R	U	U
ERR2516922	R	R	U	U
ERR2517000	R	R	R	R
ERR2516771	R	R	U	U
ERR2517013	R	R	R	R
ERR2517106	R	R	U	U
ERR2516925	R	R	S	S
ERR2517107	R	R	R	S
ERR2517018	R	R	R	R
ERR2516825	R	R	R	R
ERR2517064	R	R	U	S
ERR2516821	R	R	R	S
ERR2516806	R	R	U	U
ERR2516750	R	R	S	S
ERR2516889	S	U	S	S
ERR2516926	R	R	R	R
ERR2517121	R	R	S	S
ERR2516908	R	R	U	R
ERR2516840	R	R	U	R
ERR2516969	R	R	U	S
ERR2516932	R	R	U	S
ERR2517111	R	R	S	S
ERR2516863	R	R	R	S
ERR2516783	R	R	S	R
ERR2516793	R	R	R	S
ERR2516830	S	S	S	S
ERR2516744	R	U	S	S
ERR2516859	R	R	S	S
ERR2516762	S	U	S	S
ERR2516819	R	R	R	S
ERR2516809	R	R	R	S
ERR2516803	R	R	U	U
ERR2517016	R	R	R	R
ERR2517026	R	R	S	S
ERR2517116	R	R	U	U
ERR2516807	R	R	U	S
ERR2517073	U	R	S	S
ERR2517094	R	R	S	S
ERR2517007	R	R	S	S
ERR2516944	R	R	S	S
ERR2516868	R	R	R	R
ERR2516897	R	R	U	U
ERR2516962	R	R	S	S
ERR2517060	R	R	S	S
ERR2517037	R	R	S	S
ERR2516980	R	R	R	S
ERR2516993	R	R	R	R
ERR2516768	R	R	U	U
ERR2516858	R	R	U	U
ERR2516975	R	R	R	S

ERR2517050	S	S	S	S
ERR2516875	R	R	R	R
ERR2517005	R	R	R	R
ERR2516786	R	R	S	S
ERR2517052	R	R	U	S
ERR2516951	R	R	U	S
SAMN03648534	S	S	S	S
ERR2516902	R	R	U	S
ERR2516826	R	R	U	S
ERR2516878	R	R	S	S
ERR2517081	R	R	U	S
ERR2516791	R	R	U	S
ERR2516885	R	R	U	S
ERR2517058	S	S	S	S
ERR2516836	R	R	U	U
ERR2516877	R	R	U	U
ERR2516749	R	R	U	S
ERR2516943	R	R	R	S
ERR2516940	R	R	U	R
ERR2516961	R	R	U	S
ERR2517002	R	R	U	S
ERR2516866	R	R	U	S
ERR2516874	R	R	U	S
ERR2516755	R	S	S	S
ERR2516774	R	R	R	R
ERR2516956	R	R	U	S
ERR2517069	R	R	S	R
ERR2516964	R	R	R	S
ERR2516740	R	R	S	S
ERR2516921	R	R	U	U
ERR2517059	R	R	R	S
ERR2516746	R	R	S	S
ERR2517115	R	R	S	S
ERR2516985	R	R	R	R
ERR2516904	R	R	S	S
SAMN03648535	S	S	S	S
ERR2517031	R	R	R	S
ERR2516792	R	R	R	S
ERR2517104	R	R	S	S
ERR2517065	R	U	S	S
ERR2517124	R	R	S	S
ERR2516973	R	R	R	R
ERR2516813	R	R	U	S
ERR2516983	R	R	U	R
ERR2516914	R	R	S	R
ERR2517011	R	R	R	R
ERR2516976	R	R	S	S
ERR2517092	R	R	R	R
ERR2517100	R	R	U	R
ERR2516967	R	R	U	S
ERR2516870	R	R	R	R
ERR2516795	R	R	R	S
ERR2516895	R	R	U	R

ERR2517117	R	R	U	S
ERR2517113	R	R	U	S
ERR2516842	R	R	U	R
ERR2517119	R	R	U	S
ERR2516873	S	S	S	S
ERR2517046	R	R	U	R
ERR2516818	R	R	S	S
ERR2517051	S	S	S	S
ERR2516931	R	R	U	S
ERR2516797	R	U	U	R
ERR2517039	R	R	S	S
ERR2516853	R	R	U	U
ERR2517096	R	R	U	U
ERR2516959	R	R	R	R
ERR2517088	R	R	S	S
ERR2517001	R	R	U	U
ERR2517095	R	R	U	S
ERR2516977	R	R	U	R
ERR2516800	R	R	U	U
ERR2516820	R	R	S	S
ERR2516765	R	R	U	U
ERR2516888	R	R	R	S
ERR2516872	R	R	R	R
ERR2516997	R	R	U	S
ERR2516953	R	R	S	S
ERR2516759	R	R	U	R
ERR2517083	R	R	U	R
ERR2517071	R	R	U	R
ERR2516865	R	R	U	S
ERR2516986	R	R	U	S
ERR2516788	R	R	R	R
ERR2517032	R	R	S	R
ERR2516882	R	R	U	R
ERR2516938	R	R	U	U
ERR2516963	R	R	R	S
ERR2517098	R	R	U	S
ERR2516745	R	R	S	S
ERR2517070	R	R	R	S
ERR2517049	R	R	U	S
ERR2517080	R	R	U	S
ERR2517122	R	R	R	R
ERR2516958	R	R	U	U
ERR2516766	R	R	U	S
ERR2516907	R	R	U	S
ERR2516770	R	R	U	S
ERR2516893	R	R	U	R
ERR2516999	R	U	S	S
ERR2516927	R	R	U	R
ERR2516869	S	S	S	S
ERR2517015	R	R	U	R
ERR2517108	R	R	R	R
ERR2516987	R	R	S	S
ERR2517008	R	R	R	R

ERR2517025	R	R	S	S
ERR2516747	R	R	U	S
ERR2517012	R	R	R	S
ERR2516856	R	R	R	S
ERR2516879	R	R	R	S
ERR2517003	R	R	U	R
ERR2517009	R	R	U	R
ERR2516996	R	R	U	R
ERR2516757	R	R	R	R
ERR2516837	R	U	S	S
ERR2516864	R	U	S	S
ERR2516831	R	R	U	R
ERR2516906	R	R	U	S
ERR2516753	R	R	S	S
ERR2517028	R	R	S	S
ERR2516936	R	U	U	S
ERR2516954	R	R	U	S
ERR2516857	R	R	U	S
ERR2516798	R	R	R	R
ERR2516779	R	R	R	S
ERR2517105	S	S	S	S
ERR2516785	U	R	S	S
ERR2516776	R	S	S	S
ERR2516787	R	R	R	R
ERR2516919	R	S	S	S
ERR2516998	R	R	R	R
ERR2517020	R	R	U	S
ERR2516741	S	S	S	S
ERR2516835	R	R	U	S
ERR2516760	R	R	S	U
ERR2516972	R	R	S	S
ERR2517036	R	R	S	S
ERR2516748	R	R	R	S
ERR2517102	S	S	S	S
ERR2516887	U	R	S	S
ERR2517027	R	R	S	S
ERR2516851	R	R	U	R
ERR2516799	R	R	R	S
ERR2517072	S	S	S	S
ERR2517048	U	S	S	S
ERR2516827	R	R	S	R
ERR2517023	R	R	R	S
ERR2517014	R	R	R	R
SAMN03648536	S	S	S	S
ERR2517091	R	R	S	S
ERR2516789	R	R	S	S
ERR2516890	R	R	U	R
ERR2516971	R	R	S	R
ERR2516918	R	R	S	S
ERR2516838	R	R	U	S
ERR2516860	R	S	S	S
ERR2516916	R	R	R	S
ERR2517035	R	R	S	S

ERR2516812	R	R	R	R
ERR2516923	R	R	S	S
ERR2516854	R	R	R	R
ERR2517053	R	R	R	R
ERR2517099	R	R	U	R
ERR2516855	R	R	S	S
ERR2516816	U	U	U	S
ERR2516892	R	R	U	S
ERR2517006	R	R	U	S
ERR2516955	R	R	U	R
ERR2516991	R	R	R	S
ERR2516903	R	R	S	S
ERR2516900	R	R	R	S
ERR2517043	R	R	S	S
ERR2516948	R	R	S	S
ERR2516822	R	R	R	R
ERR2516761	R	R	U	S
ERR2516970	R	R	R	S
ERR2516965	R	R	S	R
ERR2516896	R	R	S	S
ERR2516924	R	R	R	S
ERR2516876	R	R	U	S
ERR2517034	R	R	S	S
ERR2517024	R	R	U	S
ERR2516995	R	R	U	R
ERR2516884	R	R	R	S
ERR2517040	R	R	U	S
SAMN03648537	S	S	S	R
SAMN03648538	S	S	S	S
SAMN03648539	S	S	S	S
SAMN03648540	S	S	S	S
SAMN03648541	S	S	S	S
SAMN03648542	S	S	S	S
ERR2509674	S	S	S	S
ERR2509675	S	S	S	S
SAMN03648543	S	S	S	S
SAMN03648544	S	S	S	S
ERR2509682	S	S	S	S
ERR2509880	S	S	S	S
ERR2509881	S	S	S	S
ERR2509882	S	S	S	S
ERR2509883	S	S	S	S
SAMN03648545	S	S	S	S
SAMN03648546	S	S	S	S
SAMN03648547	S	S	S	S
SAMN03648548	S	S	S	S
SAMN03648549	S	S	S	S
SAMN03648550	S	S	S	S
SAMN03648551	S	S	S	R
SAMN03648552	S	S	S	S
SAMN03648553	R	S	S	S
SAMN03648554	R	R	S	R
SAMN03648555	S	S	S	S

SAMN03648556	R	S	S	S
SAMN03648557	S	S	S	S
SAMN03648558	R	S	S	S
SAMN03648559	R	S	S	S
SAMN03648560	S	S	S	S
SAMN03648561	S	S	S	S
SAMN03648562	S	S	S	S
SAMN03648563	R	S	S	R
ERR2510838	R	R	R	U
ERR2510832	R	R	R	R
ERR2510674	R	R	R	R
ERR2510675	R	R	S	S
ERR2510676	R	R	R	R
ERR2510677	R	R	S	S
ERR2510833	R	R	R	S
ERR2510695	R	R	R	R
ERR2510678	R	R	R	R
ERR2510834	R	R	R	S
ERR2510715	R	R	R	R
ERR2510839	R	R	R	S
ERR2510655	R	R	U	R
ERR2510841	R	R	S	R
ERR2510843	R	R	R	S
ERR2510845	R	R	R	R
ERR2510329	R	R	R	R
ERR2510840	R	R	R	S
ERR2510848	R	R	R	R
ERR2510842	R	R	R	S
ERR2510827	R	R	S	R
ERR2510844	R	R	R	R
ERR2510690	R	R	R	R
ERR2510702	R	R	R	R
ERR2510847	R	R	S	S
ERR2510703	R	R	R	R
ERR2510849	R	R	R	S
ERR2510653	R	R	S	R
ERR2510846	R	R	R	R
ERR2510852	R	R	R	R
ERR2510853	R	R	R	R
ERR2510854	R	R	S	S
ERR2510855	R	R	S	R
ERR2510856	R	R	R	S
ERR2510857	R	R	R	S
ERR2510858	R	R	S	S
ERR2510709	R	R	R	R
ERR2510723	R	R	R	R
ERR2510682	R	R	R	R
ERR2510862	R	R	R	R
ERR2510863	R	R	R	R
ERR2510864	R	R	R	R
ERR2510865	R	R	S	R
ERR2510866	R	R	S	S
ERR2510867	R	R	R	R

ERR2510868	R	R	S	S
ERR2510869	R	R	R	U
ERR2510696	R	R	S	R
ERR2510851	R	R	R	R
ERR2510859	R	R	R	S
ERR2510872	R	R	R	R
ERR2510824	R	R	U	U
ERR2510874	R	R	U	U
ERR2510875	R	R	U	U
ERR2510876	R	R	S	R
ERR2510877	R	R	U	U
ERR2510878	R	R	R	R
ERR2510701	R	R	U	U
ERR2510686	R	R	R	R
ERR2510666	R	R	R	R
ERR2510667	U	U	U	R
ERR2510669	R	R	R	R
ERR2510730	R	R	R	R
ERR2510731	R	R	S	S
ERR2510732	R	R	S	S
ERR2510734	R	R	R	R
ERR2510850	R	R	R	R
ERR2510880	R	R	S	R
ERR2510735	R	R	R	R
ERR2510656	R	R	U	U
ERR2510657	R	R	U	R
ERR2510881	R	R	R	S
ERR2510704	R	R	R	R
ERR2510705	R	R	R	S
ERR2510706	R	R	S	R
ERR2510707	R	R	R	R
ERR2510708	R	R	S	R
ERR2510658	R	R	S	R
ERR2510659	R	R	S	S
ERR2510660	R	R	R	R
ERR2510661	R	R	S	S
ERR2510459	R	R	R	R
ERR2510662	R	R	S	S
ERR2510663	R	R	R	R
ERR2510664	R	R	S	S
ERR2510665	R	R	S	S
ERR2510668	R	R	R	R
ERR2510710	R	R	R	R
ERR2510670	R	R	R	R
ERR2510671	R	R	R	R
ERR2510673	R	R	R	R
ERR2510679	R	R	S	S
ERR2510882	R	R	R	R
ERR2510711	R	R	R	R
ERR2510712	R	R	S	R
ERR2510713	R	R	R	R
ERR2510714	R	R	R	R
ERR2510716	R	R	R	R

ERR2510717	R	R	R	R
ERR2510718	R	R	S	S
ERR2510719	R	R	R	R
ERR2510720	R	R	S	R
ERR2510721	R	S	R	S
ERR2510722	R	R	U	S
ERR2510680	R	R	S	R
ERR2510681	R	S	S	S
ERR2510724	R	R	U	U
ERR2510683	R	R	R	S
ERR2510254	R	R	R	R
ERR2510279	R	R	R	R
ERR2510684	R	R	R	S
ERR2510685	R	R	R	R
ERR2510725	R	R	R	S
ERR2510510	R	R	R	R
ERR2510726	R	R	R	R
ERR2510687	R	R	S	S
ERR2510688	R	R	S	S
ERR2510689	R	R	R	R
ERR2510733	R	R	R	R
ERR2510691	R	R	S	S
ERR2510692	R	R	R	R
ERR2510693	R	R	R	S
ERR2510694	R	R	R	S
ERR2510727	R	R	U	R
ERR2510728	R	R	S	S
ERR2510360	R	R	R	R
ERR2510729	R	R	R	R
ERR2510654	R	R	R	R
ERR2510823	R	R	S	S
ERR2510826	R	R	S	R
ERR2510672	R	R	S	S
ERR2510736	S	S	S	S
ERR2510835	S	S	S	S
ERR2510818	S	S	S	S
ERR2510738	S	S	S	S
ERR2510737	S	S	S	S
ERR2510739	S	S	S	S
ERR2510810	S	S	S	S
ERR2510829	S	S	S	S
ERR2510836	S	S	S	S
ERR2510819	S	S	S	S
ERR2510831	S	S	S	S
ERR2510813	S	S	S	S
ERR2510812	S	S	S	S
ERR2510820	S	S	S	S
ERR2510808	S	S	S	S
ERR2510830	S	S	S	S
ERR2510837	S	S	S	S
ERR2510821	S	S	S	S
ERR2510807	S	S	S	S
ERR2510816	S	S	S	S

ERR2510814	S	S	S	S
ERR2510811	S	S	S	S
ERR2510809	S	S	S	S
ERR2510740	S	S	S	S
ERR2510741	S	S	S	S
ERR2510742	S	S	S	R
ERR2510743	S	S	S	S
ERR2510744	S	S	S	S
ERR2510745	S	S	S	S
ERR2510746	S	S	S	S
ERR2510747	S	S	S	S
ERR2510748	S	S	S	S
ERR2510749	S	S	S	S
ERR2510750	S	S	S	S
ERR2510751	S	S	S	S
ERR2510752	S	S	S	S
ERR2510753	S	S	S	S
ERR2510754	S	S	S	S
ERR2510755	S	S	S	S
ERR2510756	S	S	S	S
ERR2510758	S	S	S	S
ERR2510759	S	S	S	S
ERR2510760	S	S	S	S
ERR2510761	S	S	S	S
ERR2510762	S	S	S	U
ERR2510763	S	S	S	S
ERR2510764	S	S	S	S
ERR2510765	S	S	S	S
ERR2510766	S	S	S	S
ERR2510767	S	S	S	S
ERR2510768	S	S	S	S
ERR2510769	S	S	S	S
ERR2510770	S	S	S	S
ERR2510771	S	S	S	S
ERR2510772	S	S	S	S
ERR2510773	S	S	S	S
ERR2510774	S	S	S	S
ERR2510775	S	S	S	S
ERR2510776	S	S	S	S
ERR2510777	S	S	S	S
ERR2510778	S	S	S	S
ERR2510779	S	S	S	S
ERR2510780	S	S	S	S
ERR2510781	S	S	S	S
ERR2510782	S	S	S	S
ERR2510783	S	S	S	S
ERR2510784	S	S	S	S
ERR2510785	S	S	S	S
ERR2510786	S	S	S	S
ERR2510787	S	S	S	S
ERR2510788	S	S	S	S
ERR2510789	S	S	S	S
ERR2510790	S	S	S	S

ERR2510791	S	S	S	S
ERR2510792	S	S	S	S
ERR2510793	S	S	S	S
ERR2510794	S	S	S	S
ERR2510795	S	S	S	S
ERR2510796	S	S	S	S
ERR2510797	S	S	S	S
ERR2510798	S	S	S	S
ERR2510799	S	S	S	S
ERR2510800	S	S	S	S
ERR2510801	S	S	S	S
ERR2510802	S	S	S	S
ERR2510803	S	S	S	S
ERR2510804	S	S	S	S
ERR2510805	S	S	S	S
ERR2510806	S	S	S	S
ERR2510817	R	S	S	S
ERR2510822	S	S	S	S
ERR2510828	S	S	S	S
ERR2515436	S	R	R	S
ERR2515202	R	R	S	R
ERR2515764	S	S	S	S
ERR2515585	S	R	S	S
ERR2516030	S	S	S	S
SAMN03648564	S	S	S	S
SAMN03648565	S	S	S	S
SAMN03648566	S	S	S	S
ERR2515763	S	S	S	S
ERR2515485	S	S	S	S
ERR2515543	S	S	S	S
ERR2515969	S	R	S	S
ERR2515691	R	R	S	S
ERR2515451	S	S	S	S
ERR2515950	R	R	R	S
ERR2515748	R	R	S	S
ERR2515130	R	R	S	R
ERR2515247	R	R	R	R
ERR2515679	S	S	S	S
ERR2515870	S	S	S	S
ERR2515332	S	S	S	S
ERR2515323	S	R	S	S
ERR2515352	S	S	S	S
ERR2515900	S	S	S	S
ERR2515701	S	R	S	S
ERR2515438	S	R	S	S
ERR2515205	S	R	S	R
ERR2515661	S	R	S	S
SAMN03648567	S	S	S	S
SAMN03648568	S	S	S	S
ERR2509684	R	S	S	S
ERR2509884	R	R	U	S
ERR2509885	S	S	S	S
ERR2509886	S	S	S	S

ERR2509887	R	S	S	S
ERR2509888	S	S	S	S
ERR2509889	R	S	S	S
SAMN03648569	S	S	S	S
SAMN03648570	S	S	S	S
ERR2509677	S	S	S	S
ERR2509890	S	S	S	S
ERR2509891	S	S	S	S
ERR2509892	R	S	S	S
SAMN03648571	S	S	S	S
SAMN03648572	S	S	S	S
SAMN03648573	S	S	S	S
SAMN03648574	S	S	S	S
SAMN03648575	S	S	S	S
SAMN03648576	S	S	S	S
SAMN03648577	S	S	S	S
SAMN03648578	S	S	S	S
SAMN03648579	S	S	S	S
SAMN03648580	S	S	S	S
ERR2516086	S	S	S	S
ERR2515603	R	R	S	S
ERR2515738	S	R	S	S
ERR2515611	S	R	S	S
ERR2517126	S	S	S	S
ERR2517127	S	S	S	S
ERR2517128	S	S	S	S
ERR2517129	S	S	S	S
ERR2517130	S	S	S	U
ERR2517131	S	S	S	S
ERR2517132	S	S	S	S
ERR2517133	S	S	S	S
ERR2517134	R	S	S	S
ERR2517135	S	S	S	S
ERR2517136	S	S	S	S
ERR2517137	R	S	S	S
ERR2517138	S	S	S	S
ERR2517139	S	S	S	S
ERR2517140	S	S	S	S
ERR2517141	S	S	S	S
ERR2517142	S	S	S	S
ERR2517143	S	S	S	S
ERR2517144	S	S	S	S
ERR2517145	S	S	S	S
ERR2517146	S	S	S	S
ERR2517147	S	S	S	U
ERR2517148	S	S	S	S
ERR2517149	S	S	S	S
ERR2517150	S	S	S	S
ERR2517151	S	S	S	R
ERR2517152	S	S	S	S
ERR2517153	S	S	S	S
ERR2517154	S	S	S	S
ERR2517155	S	S	S	S

ERR2517156	S	S	S	S
ERR2517157	R	S	S	S
ERR2517158	S	S	S	S
ERR2517159	S	S	S	S
ERR2517160	S	S	S	S
ERR2517161	S	S	S	S
ERR2517162	S	S	S	S
ERR2517163	S	S	S	S
ERR2517164	S	S	S	S
ERR2517165	S	S	S	S
ERR2517166	S	S	S	R
ERR2517167	S	S	S	S
ERR2517168	S	S	S	S
ERR2517169	S	S	S	S
ERR2517170	S	S	S	S
ERR2517171	S	S	S	S
ERR2517172	S	S	S	S
ERR2517173	S	S	S	S
ERR2517174	S	S	S	S
ERR2517175	S	S	S	S
ERR2517176	S	S	S	S
ERR2517177	S	S	S	U
ERR2517178	S	S	S	S
ERR2517179	S	S	S	R
ERR2517180	S	S	S	S
ERR2517181	S	S	S	S
ERR2517182	S	S	S	S
ERR2517183	S	S	S	S
ERR2517184	S	S	S	S
ERR2517185	S	S	S	S
ERR2517186	S	S	S	S
ERR2517187	S	S	S	S
ERR2517188	R	R	U	S
ERR2517189	S	S	S	S
ERR2517190	S	S	S	S
ERR2517191	S	S	S	U
ERR2517192	S	S	S	S
ERR2517193	R	R	U	S
ERR2517194	S	S	S	S
ERR2517195	S	S	S	R
ERR2517196	S	S	S	S
ERR2517197	S	S	S	S
ERR2517198	S	S	S	S
ERR2517199	S	S	S	S
ERR2517200	S	S	S	S
ERR2517201	S	S	S	S
ERR2517202	S	S	S	S
ERR2517203	S	S	S	U
ERR2517204	S	S	S	U
ERR2517205	S	S	S	S
ERR2517206	S	S	S	S
ERR2517207	S	S	S	S
ERR2517208	S	S	S	S

ERR2517209	S	S	S	S
ERR2517210	S	S	S	S
ERR2517211	S	S	S	S
ERR2517212	S	S	S	S
ERR2517213	S	S	S	S
ERR2517214	S	S	S	S
ERR2517215	S	S	S	S
ERR2517216	S	S	S	S
ERR2517217	S	S	S	S
ERR2517218	S	S	S	S
ERR2517219	S	S	S	U
ERR2517220	R	S	S	S
ERR2517221	S	S	S	S
ERR2517222	S	S	S	S
ERR2517223	S	S	S	S
ERR2517224	S	S	S	S
ERR2517225	S	S	S	S
ERR2517226	S	S	S	S
ERR2517227	S	S	S	R
ERR2517228	S	S	S	S
ERR2517229	S	S	S	U
ERR2517230	S	S	S	S
ERR2517231	S	S	S	S
ERR2517232	R	S	S	S
ERR2517233	S	S	S	R
ERR2517234	S	S	S	S
ERR2517235	R	S	S	S
ERR2517236	S	S	S	S
ERR2517237	S	S	S	S
ERR2517238	S	S	S	S
ERR2517239	S	S	S	R
ERR2517240	S	S	S	S
ERR2517241	S	S	S	S
ERR2517242	S	S	S	S
ERR2517243	S	S	S	U
ERR2517244	U	S	S	S
ERR2517245	S	S	S	S
ERR2517246	S	S	S	S
ERR2517247	S	S	S	S
ERR2517248	S	S	S	S
ERR2517249	S	S	S	S
ERR2517250	R	S	S	S
ERR2517251	S	S	S	S
ERR2517252	S	S	S	S
ERR2517253	S	S	S	S
ERR2517254	U	S	S	S
ERR2517255	S	S	S	S
ERR2517256	S	S	S	S
ERR2517257	S	S	S	S
ERR2517258	S	S	S	S
ERR2517259	R	R	S	S
ERR2517260	S	R	S	S
ERR2517261	S	S	S	S

ERR2517262	U	S	S	S
ERR2517263	S	S	S	S
ERR2517264	S	S	S	S
ERR2517265	S	S	S	U
ERR2517266	S	S	S	U
ERR2517267	S	S	S	S
ERR2517268	S	S	S	U
ERR2517269	S	S	S	S
ERR2517270	S	S	S	S
ERR2517271	S	S	S	S
ERR2517272	S	S	S	S
ERR2517273	S	S	S	S
ERR2517274	R	S	S	S
ERR2517275	U	S	S	S
ERR2517276	S	S	S	R
ERR2517277	S	S	S	S
ERR2517278	S	S	S	S
ERR2517279	S	S	S	S
ERR2517280	S	S	S	S
ERR2517281	R	S	S	S
ERR2517282	S	S	S	S
ERR2517283	S	S	S	S
ERR2517284	S	S	S	U
ERR2517285	S	S	S	S
ERR2517286	S	S	S	S
ERR2517287	S	S	S	S
ERR2517288	S	S	S	S
ERR2517289	R	S	S	S
ERR2517290	S	S	S	S
ERR2517291	S	S	S	S
ERR2517292	S	S	S	S
ERR2517293	S	S	S	S
ERR2517294	S	S	S	S
ERR2517295	S	S	S	S
ERR2517296	S	S	S	S
ERR2517297	S	S	S	S
ERR2517298	S	S	S	U
ERR2517299	S	S	S	S
ERR2517300	S	S	S	S
ERR2517301	R	S	S	S
ERR2517302	S	S	S	S
ERR2517303	S	S	S	S
ERR2517304	S	S	S	R
ERR2517305	R	S	S	S
ERR2517306	S	S	S	S
ERR2517307	R	R	R	R
ERR2517308	S	S	S	U
ERR2517309	R	S	S	S
ERR2517310	S	S	S	U
ERR2517311	S	S	S	S
ERR2517312	S	S	S	S
ERR2517313	S	S	S	S
ERR2517314	S	S	S	S

ERR2517315	S	S	S	U
ERR2517316	S	S	S	S
ERR2517317	S	S	S	S
ERR2517318	R	S	S	S
ERR2517319	S	S	S	S
ERR2517320	U	S	S	S
ERR2517321	S	S	S	S
ERR2517322	S	S	S	S
ERR2517323	S	S	S	U
ERR2517324	S	S	S	S
ERR2517325	S	S	S	S
ERR2517326	S	S	S	S
ERR2517327	S	S	S	S
ERR2517328	S	S	S	S
ERR2517329	S	S	S	S
ERR2517330	S	S	S	U
ERR2517331	S	S	S	S
ERR2517332	S	S	S	S
ERR2517333	R	R	S	R
ERR2517334	S	S	S	S
ERR2517335	R	S	S	S
ERR2517336	S	S	S	S
ERR2517337	S	S	S	S
ERR2517338	S	S	S	S
ERR2517339	S	S	S	S
ERR2517340	S	S	S	S
ERR2517341	S	S	S	S
ERR2517342	R	R	S	S
ERR2517343	S	S	S	S
ERR2517344	S	S	S	S
ERR2517345	S	S	S	S
ERR2517346	S	S	S	U
ERR2517347	S	S	S	S
ERR2517348	S	S	S	S
ERR2517349	S	S	S	R
ERR2517350	S	S	S	S
ERR2517351	S	S	S	S
ERR2517352	S	S	S	S
ERR2517353	S	S	S	S
ERR2517354	S	S	S	S
ERR2517355	S	S	S	S
ERR2517356	S	S	S	U
ERR2517357	S	S	S	R
ERR2517358	S	S	S	S
ERR2517359	S	S	S	S
ERR2517360	S	S	S	S
ERR2517361	S	S	S	S
ERR2517362	S	S	S	S
ERR2517363	R	S	S	S
ERR2517364	S	S	S	S
ERR2517365	S	S	S	S
ERR2517366	S	S	S	S
ERR2517367	S	S	S	S

ERR2517368	S	S	S	R
ERR2517369	S	S	S	S
ERR2517370	S	S	S	S
ERR2517371	R	R	U	R
ERR2517372	S	S	S	S
ERR2517373	S	S	S	S
ERR2517374	S	S	S	S
ERR2517375	S	S	S	S
ERR2517376	S	S	S	S
ERR2517377	S	S	S	S
ERR2517378	S	S	S	S
ERR2517379	R	R	S	S
ERR2517380	S	S	S	S
ERR2517381	S	S	S	S
ERR2517382	S	S	S	S
ERR2517383	S	S	S	U
ERR2517384	S	S	S	S
ERR2517385	S	S	S	U
ERR2517386	S	S	S	S
ERR2517387	S	S	S	S
ERR2517388	S	S	S	S
ERR2517389	S	S	S	S
ERR2517390	S	S	S	U
ERR2517391	S	S	S	U
ERR2517392	S	S	S	S
ERR2517393	S	S	S	S
ERR2517394	S	S	S	S
ERR2517395	S	S	S	S
ERR2517396	S	S	S	S
ERR2517397	S	S	S	S
ERR2517398	S	S	S	U
ERR2517399	S	S	S	S
ERR2517400	S	S	S	S
ERR2517401	S	S	S	S
ERR2517402	S	S	S	S
ERR2517403	S	S	S	S
ERR2517404	S	S	S	S
ERR2517405	S	S	S	S
ERR2517406	S	S	S	S
ERR2517407	S	S	S	S
ERR2517408	S	S	S	R
ERR2517409	S	S	S	S
ERR2517410	R	S	S	S
ERR2517411	S	S	S	S
ERR2517412	S	S	S	S
ERR2517413	S	S	S	S
ERR2517414	S	S	S	S
ERR2517415	S	S	S	S
ERR2517416	S	S	S	S
ERR2517417	S	S	S	S
ERR2517418	S	S	S	S
ERR2517419	S	S	S	S
ERR2517420	S	S	S	S

ERR2517421	S	S	S	S
ERR2517422	S	S	S	S
ERR2517423	S	S	S	S
ERR2517424	S	S	S	S
ERR2517425	S	S	S	R
ERR2517426	R	S	S	S
ERR2517427	S	S	S	U
ERR2517428	S	S	S	S
ERR2517429	S	S	S	S
ERR2517430	S	S	S	S
ERR2517431	S	S	S	S
ERR2517432	S	S	S	S
ERR2517433	R	S	S	S
ERR2517434	S	S	S	S
ERR2517435	S	S	S	U
ERR2517436	R	R	U	R
ERR2517437	S	S	S	S
ERR2517438	S	S	S	S
ERR2517439	S	S	S	S
ERR2517440	R	S	S	S
ERR2517441	S	S	S	S
ERR2517442	S	S	S	S
ERR2517443	S	S	S	R
ERR2517444	S	S	S	U
ERR2517445	S	S	S	S
ERR2517446	S	S	S	S
ERR2517447	S	S	S	S
ERR2517448	S	S	S	S
ERR2517449	S	S	S	S
ERR2517450	S	S	S	S
ERR2517451	S	S	S	S
ERR2517452	S	S	S	S
ERR2517453	S	S	S	S
ERR2517454	S	S	S	S
ERR2517455	S	S	S	S
ERR2517456	S	S	S	S
ERR2517457	S	S	S	S
ERR2517458	S	S	S	U
ERR2517459	S	S	S	S
ERR2517460	S	S	S	S
ERR2517461	S	S	S	S
ERR2517462	S	S	S	S
ERR2517463	S	S	S	S
ERR2517464	S	S	S	S
ERR2517465	S	S	S	U
ERR2517466	S	S	S	S
ERR2517467	S	S	S	S
ERR2517468	S	S	S	S
ERR2517469	S	S	S	S
ERR2517470	S	S	S	S
ERR2517471	S	S	S	S
ERR2517472	S	S	S	S
ERR2517473	S	S	S	S

ERR2517474	S	S	S	S
ERR2517475	S	S	S	S
ERR2517476	S	S	S	S
ERR2517477	S	S	S	S
ERR2517478	S	S	S	S
ERR2517479	S	S	S	S
ERR2517480	S	S	S	S
ERR2517481	S	S	S	S
ERR2517482	S	S	S	S
ERR2517483	S	S	S	S
ERR2517484	U	R	S	S
ERR2517485	S	S	S	S
ERR2517486	R	S	S	S
ERR2517487	S	S	S	S
ERR2517488	S	S	S	S
ERR2517489	S	S	S	S
ERR2517490	S	S	S	S
ERR2517491	S	S	S	S
ERR2517492	S	S	S	S
ERR2517493	S	S	S	S
ERR2517494	S	S	S	S
ERR2517495	S	S	S	R
ERR2517496	S	S	S	S
ERR2517497	R	S	S	S
ERR2517498	S	S	S	S
ERR2517499	S	S	S	S
ERR2517500	S	S	S	S
ERR2517501	S	S	S	U
ERR2517502	S	S	S	S
ERR2517503	S	S	S	S
ERR2517504	S	S	S	S
ERR2517505	S	S	S	S
ERR2517506	S	S	S	S
ERR2517507	S	S	S	S
ERR2517508	S	S	S	S
ERR2517509	S	S	S	S
ERR2517510	S	S	S	S
ERR2517511	S	S	S	S
ERR2517512	S	S	S	S
ERR2517513	S	S	S	S
ERR2517514	S	S	S	S
ERR2517515	S	S	S	U
ERR2517516	S	S	S	S
ERR2517517	S	S	S	S
ERR2517518	S	S	S	R
ERR2517519	S	S	S	S
ERR2517520	S	S	S	S
ERR2517521	S	S	S	S
ERR2517522	S	S	S	S
ERR2517523	S	S	S	R
ERR2517524	S	S	S	S
ERR2517525	S	S	S	S
ERR2517526	S	S	S	S

ERR2517527	S	S	S	S
ERR2517528	S	S	S	S
ERR2517529	S	S	S	S
ERR2517530	S	S	S	S
ERR2517531	S	S	S	S
ERR2517532	S	S	S	S
ERR2517533	S	S	S	S
ERR2517534	S	S	S	S
ERR2517535	S	S	S	S
ERR2517536	S	S	S	S
ERR2517537	S	S	S	S
ERR2517538	S	S	S	S
ERR2517539	S	S	S	S
ERR2517540	S	S	S	S
ERR2517541	S	S	S	S
ERR2517542	S	S	S	S
ERR2517543	S	S	S	S
ERR2517544	S	S	S	S
ERR2517545	S	S	S	S
ERR2517546	S	S	S	S
ERR2517547	U	R	R	S
ERR2517548	R	R	U	R
ERR2517549	S	S	S	S
ERR2517550	S	S	S	S
ERR2517551	S	S	S	S
ERR2517552	S	S	S	S
ERR2517553	S	S	S	S
ERR2517554	S	S	S	S
ERR2517555	S	S	S	S
ERR2517556	S	S	S	S
ERR2517557	S	S	S	S
ERR2517558	S	S	S	S
ERR2517559	S	S	S	S
ERR2517560	S	S	S	U
ERR2517561	S	S	S	S
ERR2517562	S	S	S	S
ERR2517563	S	S	S	U
ERR2517564	S	S	S	S
ERR2517565	S	S	S	S
ERR2517566	S	S	S	S
ERR2517567	U	S	S	U
ERR2517568	S	S	S	S
ERR2517569	R	R	U	R
ERR2517570	S	S	S	S
ERR2517571	S	S	S	S
ERR2517572	S	S	S	U
ERR2517573	S	S	S	S
ERR2517574	S	S	S	S
ERR2517575	S	S	S	S
ERR2517576	S	S	S	S
ERR2517577	S	S	S	S
ERR2517578	S	S	S	S
ERR2517579	S	S	S	S

ERR2517580	S	S	S	S
ERR2517581	S	S	S	S
ERR2517582	S	S	S	S
ERR2517583	S	S	S	S
ERR2517584	S	S	S	S
ERR2517585	S	S	S	S
ERR2517586	S	S	S	S
ERR2517587	S	S	S	S
ERR2517588	S	S	S	S
ERR2517589	S	S	S	S
ERR2517590	S	S	S	S
ERR2517591	S	S	S	S
ERR2517592	S	S	S	S
ERR2517593	S	S	S	U
ERR2517594	S	S	S	S
ERR2517595	S	S	S	S
ERR2517596	S	S	S	U
ERR2517597	S	S	S	S
ERR2517598	S	S	S	S
ERR2517599	S	S	S	S
ERR2517600	S	S	S	S
ERR2517601	S	S	S	S
ERR2517602	S	S	S	U
ERR2517603	S	S	S	S
ERR2517604	S	S	S	S
ERR2517605	S	S	S	S
ERR2517606	S	S	S	S
ERR2517607	S	S	S	S
ERR2517608	S	S	S	U
ERR2517609	S	S	S	S
ERR2517610	S	S	S	S
ERR2517611	S	S	S	S
ERR2517612	S	S	S	S
ERR2517613	S	S	S	S
ERR2517614	S	S	S	S
ERR2517615	S	S	S	S
ERR2517616	S	S	S	R
ERR2517617	S	S	S	R
ERR2517618	S	S	S	S
ERR2517619	S	S	S	S
ERR2517620	S	S	S	S
ERR2517621	S	S	S	S
ERR2517622	S	S	S	S
ERR2517623	R	S	S	S
ERR2517624	U	S	S	S
ERR2517625	S	S	S	S
ERR2517626	S	S	S	S
ERR2517627	S	S	S	U
ERR2517628	S	S	S	S
ERR2517629	S	S	S	S
ERR2517630	S	S	S	S
ERR2517631	S	S	S	S
ERR2517632	R	U	S	S

ERR2517633	S	S	S	S
ERR2517634	S	S	S	S
ERR2517635	S	S	S	S
ERR2517636	S	S	S	S
ERR2517637	S	S	S	S
ERR2517638	S	S	S	S
ERR2517639	S	S	S	S
ERR2517640	S	S	S	S
ERR2517641	S	S	S	S
ERR2517642	S	S	S	S
ERR2517643	S	S	S	S
ERR2517644	S	S	S	S
ERR2517645	S	S	S	S
ERR2517646	S	S	S	S
ERR2517647	S	S	S	S
ERR2517648	R	S	S	S
ERR2517649	S	S	S	U
ERR2517650	S	S	S	S
ERR2517651	S	S	S	S
ERR2517652	S	S	S	S
ERR2517653	S	S	S	S
ERR2516537	R	R	R	S
ERR2516670	R	R	S	S
ERR2516594	R	R	S	S
ERR2516609	R	R	S	S
ERR2516419	R	R	S	S
ERR2516622	R	R	S	S
ERR2516454	R	R	S	U
ERR2516726	R	R	S	U
ERR2516626	R	R	R	U
ERR2516575	R	R	R	U
ERR2516617	R	R	S	U
ERR2516435	R	R	R	U
ERR2516684	R	R	R	U
ERR2516449	R	R	R	S
ERR2516674	R	R	S	S
ERR2516484	R	R	S	S
ERR2516550	R	R	S	U
ERR2516495	R	R	R	R
ERR2516542	R	R	S	S
ERR2516735	R	R	S	S
ERR2516498	R	R	S	S
ERR2516561	R	R	S	S
ERR2516574	R	R	S	S
ERR2516580	R	R	S	U
ERR2516475	R	R	S	S
ERR2516468	R	R	R	S
ERR2516416	R	R	R	U
ERR2516720	R	R	R	U
ERR2516651	R	R	S	U
ERR2516584	R	R	R	S
ERR2516411	R	R	R	S
ERR2516536	R	R	R	S

ERR2516601	R	R	S	S
ERR2516474	R	R	R	S
ERR2516522	R	R	R	R
ERR2516680	R	R	S	S
ERR2516583	R	R	R	R
ERR2516582	R	R	R	U
ERR2516715	R	R	R	S
ERR2516642	R	R	S	S
ERR2516442	R	R	R	S
ERR2516714	R	R	R	S
ERR2516700	R	R	S	S
ERR2516634	R	R	S	S
ERR2516657	R	R	R	S
ERR2516545	R	R	R	R
ERR2516403	R	R	R	S
ERR2516426	R	R	S	S
ERR2516629	R	R	S	S
ERR2516535	R	R	S	S
ERR2516577	R	R	S	S
ERR2516711	R	R	S	S
ERR2516473	R	R	R	R
ERR2516421	R	R	S	S
ERR2516510	R	R	S	U
ERR2516661	R	R	S	S
ERR2516718	R	R	S	S
ERR2516516	R	R	S	U
ERR2516539	R	R	R	S
ERR2516643	R	R	S	S
ERR2516606	R	R	S	U
ERR2516596	R	R	R	R
ERR2516423	R	R	S	S
ERR2516627	R	R	R	S
ERR2516676	R	R	R	S
ERR2516703	R	R	R	S
ERR2516524	R	R	R	R
ERR2516719	R	R	R	U
ERR2516549	R	R	R	S
ERR2516586	R	R	S	S
ERR2516604	R	R	S	S
ERR2516621	R	R	R	U
ERR2516417	R	R	R	R
ERR2516655	R	R	S	S
ERR2516459	R	R	R	R
ERR2516736	R	R	U	U
ERR2516438	R	R	U	U
ERR2516652	R	R	U	U
ERR2516563	R	R	U	U
ERR2516659	R	R	U	U
ERR2516725	S	S	U	U
ERR2516466	R	R	S	U
ERR2516489	R	R	S	S
ERR2516521	R	R	R	U
ERR2516633	R	R	R	U

ERR2516401	R	R	S	U
ERR2516406	R	R	S	U
ERR2516613	R	R	S	U
ERR2516415	R	R	S	U
ERR2516579	R	R	R	U
ERR2516571	R	R	R	U
ERR2516689	R	R	R	S
ERR2516555	R	R	R	U
ERR2516569	R	R	S	U
ERR2516453	R	R	R	U
ERR2516553	R	R	S	U
ERR2516444	R	R	S	U
ERR2516640	R	R	S	U
ERR2516679	R	R	S	U
ERR2516566	R	R	S	S
ERR2516470	R	R	S	U
ERR2516556	R	R	S	S
ERR2516402	R	R	S	U
ERR2516432	R	R	R	U
ERR2516506	R	R	R	S
ERR2516671	R	R	S	R
ERR2516461	R	R	S	S
ERR2516694	R	R	R	S
ERR2516567	R	R	S	S
ERR2516712	R	R	R	U
ERR2516603	R	R	S	S
ERR2516695	R	R	S	S
ERR2516519	R	R	S	S
ERR2516471	R	R	S	U
ERR2516445	R	R	S	S
ERR2516638	R	R	S	R
ERR2516448	R	R	S	S
ERR2516477	R	R	S	R
ERR2516729	R	R	R	S
ERR2516607	R	R	R	R
ERR2516562	R	R	R	S
ERR2516494	R	R	S	R
ERR2516588	R	R	R	U
ERR2516672	R	R	S	S
ERR2516660	R	R	R	R
ERR2516692	R	R	S	S
ERR2516641	R	R	S	R
ERR2516447	R	R	R	R
ERR2516730	R	R	R	R
ERR2516593	R	R	R	R
ERR2516407	R	R	S	R
ERR2516413	R	R	R	S
ERR2516656	R	R	S	R
ERR2516653	R	R	R	S
ERR2516439	R	R	R	U
ERR2516410	R	R	S	S
ERR2516722	R	R	R	S
ERR2516551	R	R	S	S

ERR2516505	R	R	S	S
ERR2515445	R	R	S	R
ERR2515745	R	R	S	S
ERR2515961	S	S	S	S
ERR2515674	S	R	S	S
ERR2515329	S	R	S	S
ERR2515382	S	R	S	S
ERR2515645	S	R	S	S
ERR2515663	R	R	S	S
SAMN03648581	S	S	S	S
ERR2515697	S	R	S	S
ERR2509678	S	S	S	S
ERR2509679	S	S	S	S
ERR2509676	S	S	S	S
ERR2515777	R	R	R	R
ERR2515369	S	R	S	S
ERR2515686	S	R	S	S
ERR2515936	R	R	R	S
ERR2515648	R	R	R	S
ERR2515732	S	R	S	U
ERR2515155	R	R	S	S
ERR2515506	R	R	S	R
ERR2515266	S	R	S	S
ERR2515348	R	R	R	S
ERR2515982	R	R	R	R
ERR2515497	S	S	S	S
SAMN03648582	S	S	S	S
SAMN03648583	S	S	S	S
ERR2515384	R	R	S	S
ERR2515407	R	R	S	R
ERR2515131	R	R	R	R
ERR2515917	R	R	R	S
ERR2516003	R	R	S	S
ERR2515962	R	R	R	S
ERR2515119	S	R	S	S
ERR2515643	R	R	R	R
ERR2515851	R	R	S	S
ERR2515552	R	R	S	R
ERR2516067	R	R	R	S
ERR2515342	R	R	R	R
ERR2515815	S	S	S	S
ERR2515211	S	S	S	S
ERR2515856	S	R	S	S
ERR2515542	R	R	R	R
ERR2515311	S	S	S	S
ERR2515970	S	S	S	S
ERR2515859	R	R	S	S
SAMN03648584	S	S	S	S
SAMN03648585	S	S	S	S
SAMN03648586	S	S	S	S
ERR2515376	R	R	R	S
ERR2515223	S	R	S	U
ERR2515498	S	R	S	S

ERR2515224 R R S S
ERR2515225 R R S S
ERR2515666 R R S S
ERR2515868 R R S S
ERR2515670 S R S S
ERR2515277 S R S S
ERR2515801 R R S S
ERR2515231 S R S S
ERR2515347 R R S S
ERR2515258 R R S S
ERR2515948 R R R R
SAMN03648587 S S S S
SAMN03648588 S S S S
SAMN03648589 R S S S
SAMN03648590 S S S S
SAMN03648591 R R S R
ERR2515442 S R S S
ERR2515791 S S S S
SRR6367399 PRJNA393378 R R R R
SRR5817475 PRJNA393378 R R S S
SRR6339662 PRJNA393378 R R S S
SRR6339642 PRJNA393378 R R R R
SRR6367401 PRJNA393378 R R R R
SRR5817464 PRJNA393378 R R R R
SRR2333215 PRJNA270697┬á R R S R
SRR5817469 PRJNA393378 R R R R
SRR6339643 PRJNA393378 R R R R
SRR5817467 PRJNA393378 R R S S
SRR5817471 PRJNA393378 R R S R
SRR5817478 PRJNA393378 R R S R
SRR6339640 PRJNA393378 R R S R
SRR6367396 PRJNA393378 R R S S
SRR6339641 PRJNA393378 R R S S
SRR5817479 PRJNA393378 R R S R
SRR6339637 PRJNA393378 R R S S
SRR6339648 PRJNA393378 R R S S
SRR6339651 PRJNA393378 R R R S
SRR6339645 PRJNA393378 R R S S
SRR6339653 PRJNA393378 R R R R
SRR6369876 PRJNA393378 R R S R
SRR6339661 PRJNA393378 R R S S
SRR6339667 PRJNA393378 R S R S
SRR5817481 PRJNA393378 R R R S
SRR5817480 PRJNA393378 R R S S
SRR5817470 PRJNA393378 R R R R
SRR6339649 PRJNA393378 R R R S
SRR5817466 PRJNA393378 R R R R
SRR6339664 PRJNA393378 R R S S
SRR5817473 PRJNA393378 R R S R
SRR5817472 PRJNA393378 R R R R
SRR6369875 PRJNA393378 R R S R
SRR6367398 PRJNA393378 R R S R
SRR6339665 PRJNA393378 R S S R

SRR2328057 PRJNA270697┬á R S S R
SRR6369877 PRJNA393378 R S R S
SRR6367400 PRJNA393378 R R R S
SRR6369878 PRJNA393378 R R S R
SRR5817463 PRJNA393378 R R S S
SRR6339666 PRJNA393378 R R S R
SRR6339644 PRJNA393378 R R R R
SRR6367395 PRJNA393378 S S S S
SRR6339663 PRJNA393378 S S S S
ERR2509673 S S S S
ERR2509893 S S S S
SAMN03648592 S S S S
SAMN03648593 S S S S
SAMN03648594 S S S S
SAMN03648595 S S S S
SAMN03648596 R S S S
SAMN03648597 S S S S
SAMN03648598 R S S R
ERR2515523 R R S S
SAMN03648599 S S S S
SAMN03648600 S S S S
SAMN03648601 S S S S
SAMN03648602 S U S U
SAMN03648603 S S S S
SAMN03648604 S S S R
SAMN03648605 S S S S
SAMN03648606 S S S S
SAMN03648607 R R R S
SAMN03648608 S S S S
SAMN03648609 S S S S
SAMN03648610 S S S S
SAMN03648611 S S S S
SAMN03648612 S S S S
SAMN03648613 S R R S
SAMN03648614 S S S S
SAMN03648615 R S S S
SAMN03648616 S S S S
SAMN03648617 S S S S
SAMN03648618 S S S S
SAMN03648619 S S S S
SAMN03648620 S R S S
SAMN03648621 S S S S
SAMN03648622 S S S S
SAMN03648623 S S S U
SAMN03648624 S S S S
SAMN03648625 S U S U
SAMN03648626 S U S U
SAMN03648627 S S S S
SAMN03648628 S S S S
SAMN03648629 S S S S
SAMN03648630 S U S U
SAMN03648631 R R R R
SAMN03648632 S S S S

SAMN03648633	S	U	S	U
SAMN03648634	S	S	S	S
SAMN03648635	S	S	S	S
SAMN03648636	S	S	S	U
SAMN03648637	S	U	S	U
SAMN03648638	S	S	S	S
SAMN03648639	R	S	S	S
SAMN03648640	S	S	S	S
SAMN03648641	S	S	S	S
SAMN03648642	S	U	S	U
SAMN03648643	S	S	S	S
SAMN03648644	S	S	S	S
SAMN03648645	S	S	S	S
SAMN03648646	S	S	S	S
SAMN03648647	S	S	S	S
SAMN03648648	S	S	S	S
SAMN03648649	S	S	S	S
SAMN03648650	S	U	S	U
SAMN03648651	S	S	S	S
SAMN03648652	R	R	S	S
SAMN03648653	S	S	S	S
SAMN03648654	S	S	S	U
SAMN03648655	S	S	S	S
SAMN03648656	S	S	S	S
SAMN03648657	S	S	S	S
SAMN03648658	S	U	S	U
SAMN03648659	R	U	S	U
SAMN03648660	S	U	S	U
SAMN03648661	S	S	S	S
SAMN03648662	R	S	S	S
SAMN03648663	S	U	U	U
SAMN03648664	S	U	S	U
SAMN03648665	S	S	S	S
SAMN03648666	S	S	S	S
SAMN03648667	S	U	S	U
SAMN03648668	S	S	S	S
SAMN03648669	S	S	S	S
SAMN03648670	S	S	S	S
SAMN03648671	S	S	S	U
SAMN03648672	S	S	S	S
SAMN03648673	S	U	S	U
SAMN03648674	S	R	S	S
SAMN03648675	S	S	S	S
SAMN03648676	S	S	S	S
SAMN03648677	S	U	U	U
SAMN03648678	S	S	S	S
SAMN03648679	S	S	S	S
SAMN03648680	S	S	S	S
ERR2515209	R	R	S	S
SAMN03648681	S	S	S	S
SAMN03648682	S	S	S	S
SAMN03648683	S	S	S	S
SAMN03648684	S	S	S	S

SAMN03648685	S	S	S	U
SAMN03648686	S	S	S	S
SAMN03648687	S	S	S	S
SAMN03648688	S	S	S	S
SAMN03648689	S	S	S	S
SAMN03648690	R	S	S	S
SAMN03648691	S	S	S	S
SAMN03648692	S	S	S	S
SAMN03648693	S	S	S	S
SAMN03648694	S	S	S	S
SAMN03648695	S	S	S	S
SAMN03648696	S	S	S	S
SAMN03648697	S	S	S	S
SAMN03648698	S	S	S	U
SAMN03648699	S	U	S	U
SAMN03648700	S	S	S	S
SAMN03648701	R	S	S	U
SAMN03648702	S	S	S	S
SAMN03648703	S	S	S	S
SAMN03648704	S	S	S	R
SAMN03648705	S	S	S	S
SAMN03648706	S	S	S	S
SAMN03648707	S	R	S	S
SAMN03648708	S	S	S	S
SAMN03648709	S	S	S	S
SAMN03648710	S	S	S	S
SAMN03648711	S	S	S	S
SAMN03648712	S	S	S	S
SAMN03648713	S	S	S	S
SAMN03648714	R	S	R	R
SAMN03648715	S	S	S	S
SAMN03648716	R	S	S	S
SAMN03648717	R	R	S	S
SAMN03648718	S	S	S	S
SAMN03648719	S	S	S	S
SAMN03648720	S	U	S	U
SAMN03648721	S	S	S	S
SAMN03648722	S	S	S	S
SAMN03648723	S	S	S	S
SAMN03648724	S	S	S	S
SAMN03648725	S	S	S	S
SAMN03648726	S	S	S	S
ERR2515573	R	R	R	S
SAMN03648727	S	S	S	S
SAMN03648728	S	S	S	S
SAMN03648729	R	R	R	R
ERR2515301	R	R	S	R
SAMN03648730	S	S	S	S
SAMN03648731	S	S	S	S
SAMN03648732	S	S	S	S
SAMN03648733	S	S	S	S
SAMN03648734	S	S	S	S
ERR2516025	R	R	R	R

ERR2515623	S	R	S	S
SAMN03648735	S	S	S	S
SAMN03648736	S	S	S	S
SAMN03648737	S	S	S	S
SAMN03648738	S	S	S	S
SAMN03648739	S	S	S	S
SAMN03648740	R	S	S	R
SAMN03648741	S	S	S	S
SAMN03648742	S	R	S	S
SAMN03648743	S	S	S	S
SAMN03648744	S	S	S	S
SAMN03648745	S	S	S	S
SAMN03648746	S	U	S	U
SAMN03648747	S	S	S	S
SAMN03648748	S	S	S	S
SAMN03648749	S	S	S	S
SAMN03648750	S	S	S	S
SAMN03648751	S	S	S	S
SAMN03648752	R	R	S	S
SAMN03648753	S	S	S	U
SAMN03648754	S	S	S	S
SAMN03648755	S	S	S	S
SAMN03648756	S	S	S	S
SAMN03648757	S	S	S	S
SAMN03648758	S	S	S	R
SAMN03648759	S	S	S	S
SAMN03648760	S	S	S	S
SAMN03648761	S	U	S	U
SAMN03648762	S	S	S	S
SAMN03648763	R	S	S	S
SAMN03648764	S	S	S	S
SAMN03648765	S	S	S	S
SAMN03648766	R	S	S	R
SAMN03648767	S	S	S	S
SAMN03648768	S	S	S	S
SAMN03648769	S	S	S	S
SAMN03648770	S	S	S	S
SAMN03648771	S	S	S	R
SAMN03648772	S	S	S	S
SAMN03648773	S	S	S	S
SAMN03648774	S	S	S	S
SAMN03648775	S	S	S	S
SAMN03648776	S	S	S	S
SAMN03648777	R	S	S	S
SAMN03648778	S	S	S	S
SAMN03648779	S	S	S	S
SAMN03648780	S	S	S	S
SAMN03648781	S	S	S	S
SAMN03648782	S	U	S	U
SAMN03648783	S	S	S	U
SAMN03648784	S	S	S	S
SAMN03648785	S	S	S	S
SAMN03648786	R	S	R	R

SAMN03648787	S	S	S	S
SAMN03648788	R	S	S	S
SAMN03648789	S	S	S	S
SAMN03648790	S	U	U	U
SAMN03648791	S	S	S	S
SAMN03648792	S	S	S	S
SAMN03648793	S	S	S	U
SAMN03648794	S	S	S	S
SAMN03648795	S	S	S	S
SAMN03648796	S	S	S	U
SAMN03648797	S	S	S	S
SAMN03648798	S	U	S	U
SAMN03648799	S	S	S	S
SAMN03648800	S	S	S	S
SAMN03648801	S	S	S	S
SAMN03648802	R	R	S	R
SAMN03648803	S	S	S	S
ERR2515995	R	R	R	R
SAMN03648804	S	S	S	S
SAMN03648805	S	U	S	U
SAMN03648806	S	S	S	S
SAMN03648807	S	S	S	S
SAMN03648808	S	S	S	S
SAMN03648809	S	R	S	S
SAMN03648810	S	U	S	U
SAMN03648811	S	U	U	U
SAMN03648812	S	S	S	S
SAMN03648813	S	U	S	U
SAMN03648814	S	S	S	S
SAMN03648815	S	S	S	S
SAMN03648816	S	S	S	S
SAMN03648817	S	S	S	S
SAMN03648818	S	S	S	S
SAMN03648819	S	R	S	S
SAMN03648820	S	U	S	U
SAMN03648821	R	R	R	R
SAMN03648822	S	S	S	S
SAMN03648823	S	S	S	S
SAMN03648824	S	S	S	S
SAMN03648825	S	S	S	S
SAMN03648826	R	S	S	S
SAMN03648827	S	S	S	U
SAMN03648828	S	S	S	R
SAMN03648829	S	S	S	U
SAMN03648830	S	S	S	S
SAMN03648831	S	S	S	S
SAMN03648832	S	S	S	R
ERR2515774	S	R	S	S
SAMN03648833	S	U	S	U
SAMN03648834	S	U	S	S
SAMN03648835	S	S	S	S
ERR2515482	R	R	S	S
SAMN03648836	S	U	S	U

SAMN03648837	S	R	S	S
SAMN03648838	S	S	S	S
ERR2515283	R	R	S	S
ERR2515356	S	R	S	R
SAMN03648839	S	S	S	S
SAMN03648840	S	S	S	S
SAMN03648841	S	S	S	S
SAMN03648842	S	R	S	S
SAMN03648843	S	S	S	S
ERR2516090	S	R	S	S
SAMN03648844	R	R	R	R
SAMN03648845	R	R	S	R
ERR2515166	R	R	S	S
SAMN03648846	S	S	S	S
SAMN03648847	S	S	S	S
SAMN03648848	S	S	S	S
SAMN03648849	S	S	S	S
SAMN03648850	S	S	S	S
SAMN03648851	S	S	S	S
SAMN03648852	R	S	S	S
SAMN03648853	S	S	S	S
SAMN03648854	R	S	S	U
SAMN03648855	S	S	S	S
ERR2515267	S	R	S	S
SAMN03648856	S	S	S	S
SAMN03648857	S	S	S	S
SAMN03648858	S	S	S	S
SAMN03648859	S	S	S	S
SAMN03648860	S	S	S	S
SAMN03648861	R	S	S	S
SAMN03648862	S	S	S	S
SAMN03648863	S	S	S	S
SAMN03648864	S	S	S	S
SAMN03648865	S	S	S	S
SAMN03648866	S	S	S	S
SAMN03648867	S	S	S	S
ERR2515463	S	R	S	S
SAMN03648868	S	S	S	S
ERR2515246	R	R	S	R
SAMN03648869	S	S	S	S
SAMN03648870	S	R	S	S
SAMN03648871	S	S	S	S
SAMN03648872	S	S	S	S
SAMN03648873	S	S	S	S
SAMN03648874	S	S	S	S
SAMN03648875	S	S	S	S
SAMN03648876	R	S	S	S
SAMN03648877	S	S	S	S
SAMN03648878	S	S	S	S
SAMN03648879	S	S	S	S
SAMN03648880	S	S	S	S
SAMN03648881	R	S	S	S
SAMN03648882	R	S	S	S

SAMN03648883	S	S	S	S
SAMN03648884	R	S	S	S
SAMN03648885	S	S	S	S
SAMN03648886	R	S	S	S
SAMN03648887	S	S	S	S
SAMN03648888	S	R	S	S
SAMN03648889	S	S	S	S
SAMN03648890	S	S	S	S
SAMN03648891	S	S	S	S
SAMN03648892	S	S	S	S
SAMN03648893	S	S	S	S
ERR2515703	R	S	S	S
SAMN03648894	S	S	S	S
SAMN03648895	S	S	S	S
SAMN03648896	S	S	S	S
ERR2515937	R	R	S	S
SAMN03648897	R	S	S	S
SAMN03648898	S	S	S	S
SAMN03648899	S	S	S	S
ERR2515576	R	R	S	S
SAMN03648900	R	S	S	S
SAMN03648901	S	S	S	S
SAMN03648902	S	S	S	S
SAMN03648903	S	S	S	R
SAMN03648904	S	S	S	S
SAMN03648905	S	S	S	S
SAMN03648906	S	S	S	S
SAMN03648907	S	S	S	S
SAMN03648908	S	S	S	S
SAMN03648909	S	S	S	S
SAMN03648910	S	S	S	S
SAMN03648911	S	S	S	R
SAMN03648912	S	S	S	S
SAMN03648913	S	S	S	S
ERR2515857	S	R	S	S
SAMN03648914	S	S	S	S
ERR2515761	R	S	S	S
SAMN03648915	S	S	S	S
SAMN03648916	S	S	S	S
SAMN03648917	S	S	S	S
SAMN03648918	S	S	S	S
SAMN03648919	S	S	S	S
SAMN03648920	S	S	S	S
ERR2515709	S	S	S	S
SAMN03648921	S	S	S	S
SAMN03648922	R	R	S	S
SAMN03648923	S	S	S	S
SAMN03648924	S	S	S	S
SAMN03648925	S	S	S	S
SAMN03648926	S	S	S	S
SAMN03648927	R	S	S	S
SAMN03648928	S	S	S	S
SAMN03648929	S	S	S	S

SAMN03648930	R	S	S	S
SAMN03648931	S	S	S	S
ERR2515882	S	R	S	S
ERR2515121	R	R	S	S
SAMN03648932	S	S	S	S
SAMN03648933	R	S	S	S
ERR2515737	R	R	S	S
SAMN03648934	S	S	S	S
SAMN03648935	S	S	S	S
ERR2515890	S	R	S	S
SAMN03648936	S	S	S	S
SAMN03648937	R	R	S	S
SAMN03648938	S	S	S	S
SAMN03648939	S	S	S	S
SAMN03648940	S	S	S	S
SAMN03648941	S	S	S	S
SAMN03648942	S	S	S	S
SAMN03648943	S	S	S	S
SAMN03648944	S	S	S	S
ERR2515612	R	R	S	R
SAMN03648945	S	S	S	S
SAMN03648946	S	S	S	S
SAMN03648947	S	S	S	S
SAMN03648948	R	S	S	S
ERR2515417	S	R	S	S
SAMN03648949	S	S	S	S
SAMN03648950	S	S	S	S
SAMN03648951	S	S	S	S
SAMN03648952	S	S	S	S
SAMN03648953	S	S	S	S
SAMN03648954	S	S	S	S
SAMN03648955	S	R	S	S
ERR2515294	R	R	S	R
SAMN03648956	S	S	S	S
SAMN03648957	S	S	S	S
SAMN03648958	S	S	S	S
SAMN03648959	S	S	S	R
ERR2515123	R	R	S	S
SAMN03648960	S	S	S	S
SAMN03648961	S	S	S	S
ERR2515371	R	R	S	R
ERR2515571	S	R	S	S
SAMN03648962	S	S	S	S
SAMN03648963	S	S	S	S
SAMN03648964	S	S	S	S
SAMN03648965	S	S	S	S
SAMN03648966	S	S	S	S
SAMN03648967	S	S	S	S
SAMN03648968	S	S	S	S
SAMN03648969	S	S	S	S
SAMN03648970	S	S	S	S
SAMN03648971	S	S	S	S
SAMN03648972	S	S	S	S

SAMN03648973	S	S	S	S
SAMN03648974	S	S	S	S
SAMN03648975	S	S	S	S
SAMN03648976	S	S	S	S
SAMN03648977	S	S	S	S
SAMN03648978	R	R	S	R
SAMN03648979	S	S	R	S
ERR2515684	R	R	R	R
SAMN03648980	S	S	S	R
SAMN03648981	S	S	S	S
SAMN03648982	S	S	S	S
SAMN03648983	S	S	S	S
SAMN03648984	S	S	S	S
SAMN03648985	S	S	S	S
SAMN03648986	S	S	S	S
ERR2515539	S	R	S	S
SAMN03648987	S	S	S	S
SAMN03648988	R	S	S	S
SAMN03648989	S	S	S	S
SAMN03648990	R	S	S	S
SAMN03648991	S	S	S	S
SAMN03648992	S	S	S	S
SAMN03648993	S	S	S	S
SAMN03648994	S	S	S	S
SAMN03648995	S	S	S	S
SAMN03648996	S	S	S	S
SAMN03648997	S	S	S	S
SAMN03648998	S	S	S	S
SAMN03648999	S	S	S	S
SAMN03649000	R	R	S	S
SAMN03649001	S	R	S	S
SAMN03649002	S	S	S	S
SAMN03649003	S	S	S	S
SAMN03649004	R	R	R	R
SAMN03649005	S	S	S	S
SAMN03649006	S	S	S	S
SAMN03649007	S	S	S	S
SAMN03649008	R	R	S	U
SAMN03649009	S	S	S	S
SAMN03649010	R	R	S	R
SAMN03649011	S	S	S	S
SAMN03649012	S	S	S	S
SAMN03649013	S	S	S	S
SAMN03649014	S	S	S	S
SAMN03649015	S	S	S	S
SAMN03649016	S	S	S	S
SAMN03649017	S	S	S	S
SAMN03649018	R	S	S	S
SAMN03649019	S	S	S	S
SAMN03649020	S	S	S	S
SAMN03649021	S	S	S	S
ERR2515228	R	R	R	R
SAMN03649022	R	R	S	S

SAMN03649023	S	S	S	S
SAMN03649024	S	S	S	S
SAMN03649025	S	S	S	S
SAMN03649026	S	S	S	S
SAMN03649027	S	S	S	S
SAMN03649028	S	S	S	S
SAMN03649029	S	S	S	S
SAMN03649030	S	S	S	S
SAMN03649031	S	S	S	S
SAMN03649032	S	S	S	S
SAMN03649033	S	S	S	S
SAMN03649034	S	S	S	S
SAMN03649035	S	S	S	S
SAMN03649036	S	S	S	S
SAMN03649037	S	S	S	S
SAMN03649038	S	S	S	S
SAMN03649039	S	S	S	S
SAMN03649040	S	S	S	S
SAMN03649041	S	S	S	S
SAMN03649042	S	S	S	S
SAMN03649043	S	S	S	S
SAMN03649044	S	S	S	S
SAMN03649045	R	S	S	S
SAMN03649046	S	S	S	S
ERR2515174	S	S	S	S
SAMN03649047	S	S	S	S
SAMN03649048	S	S	S	S
SAMN03649049	S	S	S	S
ERR2515979	S	S	S	S
SAMN03649050	S	S	S	S
ERR2515186	S	S	S	S
SAMN03649051	S	S	S	S
SAMN03649052	S	S	S	S
SAMN03649053	R	R	R	R
SAMN03649054	S	S	S	S
SAMN03649055	S	S	S	S
SAMN03649056	S	S	S	S
SAMN03649057	S	S	S	S
SAMN03649058	S	S	S	S
ERR2515112	R	R	R	R
ERR2515143	S	R	S	S
ERR2515983	S	R	S	S
ERR2515583	R	R	U	R
SAMN03649059	S	S	S	S
SAMN03649060	S	S	S	R
ERR2515389	R	R	S	S
ERR2515567	R	R	R	R
ERR2516029	S	R	S	S
ERR2515304	R	R	R	S
SAMN03649061	S	S	S	S
ERR2515362	S	R	S	S
ERR2516024	R	R	R	S
ERR2515518	S	R	S	S

ERR2515396 S R S S
SAMN03649062 S S S S
ERR2515559 R R S S
ERR2515252 R R R R
SAMN03649063 S S S S
ERR2515711 R R S S
ERR2515217 S S S S
ERR2515488 R R R R
ERR2516007 S R S S
ERR2515606 R R S R
ERR2515345 R R S R
ERR2516020 R R S S
ERR2515874 R R S S
ERR2515963 S S S S
ERR2515781 S R S S
ERR2515165 R R R U
ERR2515664 R R S R
ERR2516049 R R R U
ERR2516001 R R S S
ERR2515978 R R R S
ERR2515949 R R R S
ERR2515650 R R R U
ERR2515390 R R R R
ERR2516031 R R R R
ERR2515117 S S S S
ERR2515360 S S S S
ERR2515755 S S S S
ERR2515938 R R S S
ERR2515903 R R S R
ERR2515126 S R S S
ERR2515189 R R S S
ERR2515565 S R S S
ERR2515952 R R S R
ERR2515423 R R S R
ERR2515218 S R S S
ERR2516069 R R R R
ERR2515147 R R S R
ERR2515682 R R R R
ERR2515107 S R S S
ERR2515997 R R R R
ERR2515934 R R R S
ERR2515759 R R S R
ERR2515221 S R S S
ERR2515178 S R S S
ERR2515628 S R S S
ERR2515486 R R R R
ERR2515310 R R R R
ERR2515284 R R S R
ERR2515614 R R R R
ERR2516004 R R S S
ERR2515634 R R S S
ERR2515615 R R S R
ERR2515724 R R R R

ERR2516010	R	R	S	S
ERR2515441	S	R	S	S
ERR2515249	R	R	R	R
ERR2515163	R	R	R	R
ERR2516034	S	R	S	S
ERR2515346	R	R	R	S
ERR2515554	R	R	R	R
ERR2515933	R	R	R	R
ERR2515705	R	R	S	R
ERR2515633	R	R	S	S
ERR2516060	R	R	S	S
ERR2515350	S	R	S	S
ERR2515227	R	R	S	R
ERR2515749	R	R	R	S
ERR2515530	R	R	S	S
ERR2515109	R	R	R	S
ERR2515410	R	R	R	R
ERR2515915	S	R	S	S
ERR2515798	R	R	R	R
ERR2516057	R	R	R	R
ERR2515210	R	R	S	R
ERR2516063	U	R	R	U
ERR2515708	U	R	S	U
ERR2515182	R	R	R	R
ERR2515754	U	R	R	U
ERR2515591	U	R	R	U
ERR2515213	U	R	R	U
ERR2515802	U	R	R	U
ERR2515361	U	R	R	U
ERR2515990	U	R	R	U
ERR2515810	U	R	R	U
ERR2515541	U	R	R	U
ERR2515358	U	R	S	U
ERR2515590	U	R	R	U
ERR2515173	U	R	R	U
ERR2515864	U	R	R	U
ERR2515513	U	R	S	U
ERR2515544	U	R	R	U
ERR2515920	U	R	R	U
ERR2515794	U	R	R	U
ERR2515660	U	R	R	U
ERR2515996	U	R	R	U
ERR2515528	U	R	R	U
ERR2515776	U	R	R	U
ERR2515833	U	R	R	U
ERR2515710	U	S	S	U
ERR2515910	U	R	R	U
ERR2515546	U	R	R	U
ERR2516017	U	R	R	U
ERR2515588	U	R	R	U
ERR2516005	U	R	R	U
ERR2515797	U	R	R	U
ERR2515867	U	R	R	U

ERR2515658	U	R	R	U
ERR2515286	U	R	R	U
ERR2515335	U	R	S	U
ERR2515839	U	R	R	U
ERR2515475	U	R	R	U
ERR2515248	U	R	S	U
ERR2515667	U	R	R	U
ERR2515479	U	R	R	U
ERR2516023	U	R	R	U
ERR2515135	U	R	R	U
ERR2515636	U	R	R	U
ERR2515924	U	R	R	U
ERR2516045	U	R	R	U
ERR2515519	U	R	R	U
ERR2515597	U	R	R	U
ERR2515826	U	R	R	U
ERR2516064	U	R	R	U
ERR2515419	U	R	R	U
ERR2515199	U	R	R	U
ERR2515520	U	R	S	U
ERR2515560	U	R	R	U
ERR2515965	U	R	R	U
ERR2515344	U	R	R	U
ERR2516012	U	R	R	U
ERR2515756	U	R	R	U
ERR2516041	U	S	S	U
ERR2515860	U	R	S	U
ERR2515159	U	R	R	U
ERR2515322	U	R	R	U
ERR2515589	U	R	R	U
ERR2516055	U	R	R	U
ERR2515575	S	S	S	U
ERR2515640	U	R	R	U
ERR2515739	U	R	R	U
ERR2515812	U	R	S	U
ERR2515594	U	R	R	U
ERR2515609	U	R	R	U
ERR2515319	U	R	R	U
ERR2515473	U	R	R	U
ERR2515898	U	R	R	U
ERR2515411	U	R	R	U
ERR2515743	U	R	R	U
ERR2515331	U	R	R	U
ERR2515926	U	R	R	U
ERR2515584	U	R	R	U
ERR2515806	S	S	S	U
ERR2515835	U	R	R	U
ERR2515405	U	R	R	U
ERR2515725	U	R	R	U
ERR2516066	U	R	R	U
ERR2515719	U	R	R	U
ERR2515386	U	R	R	U
ERR2515152	U	R	R	U

ERR2515514	U	R	R	U
ERR2515747	U	R	S	U
ERR2516019	U	R	R	U
ERR2515872	U	S	R	U
ERR2515425	U	R	R	U
ERR2515325	U	R	S	U
ERR2515175	U	R	R	U
ERR2515789	U	R	S	U
ERR2515878	U	S	S	U
ERR2515918	U	S	S	U
ERR2515415	U	S	S	U
ERR2515373	U	R	R	U
ERR2515238	U	R	R	U
ERR2515971	U	R	R	U
ERR2515263	U	R	R	U
ERR2515568	U	R	R	U
ERR2515735	R	R	R	R
ERR2515458	U	R	R	U
ERR2516014	U	R	R	U
ERR2515133	U	R	R	U
ERR2516077	U	R	S	U
ERR2515115	R	R	R	U
ERR2515911	S	R	S	U
ERR2515931	R	R	S	U
ERR2515768	U	R	R	U
ERR2515250	U	R	R	U
ERR2515988	U	R	R	U
ERR2516068	U	R	R	U
ERR2515144	U	R	R	U
ERR2515337	U	R	R	U
ERR2516018	R	R	R	U
ERR2515153	U	R	R	U
ERR2515333	U	R	S	U
ERR2515647	U	R	S	U
ERR2515354	S	R	S	U
ERR2515758	R	R	R	R
ERR2516097	S	R	S	S
ERR2515448	R	R	S	S
ERR2515744	S	S	S	S
ERR2515577	R	R	R	R
ERR2516091	R	R	R	R
ERR2515466	R	R	R	R
ERR2515720	R	R	S	S
SAMN03649064	S	S	S	S
ERR2509894	S	S	S	S
SAMN03649065	S	S	S	S
SAMN03649066	S	S	S	U
SAMN03649067	S	S	S	S
SAMN03649068	S	S	S	S
ERR2509683	S	S	S	S
SAMN03649069	S	S	S	S
ERR2509895	S	S	S	S
ERR2509685	S	S	S	S

ERR2509686	S	S	S	S
ERR2509896	S	S	S	S
ERR2509897	R	R	R	S
ERR2509879	S	S	S	S
SAMN03649070	S	S	S	S
SAMN03649071	S	S	S	S
ERR2515909	S	S	S	S
SAMN03649072	S	S	S	S
SAMN03649073	S	S	S	S
SAMN03649074	S	S	S	S
SAMN03649075	S	S	S	S
