# Supplementary material for: Multi-Label Random Forest Model for Tuberculosis Drug Resistance Classification and Mutation Ranking
Source: Front Microbiol. 2020 Apr 22;11:667. doi: 10.3389/fmicb.2020.00667 (PMC7188832; doi:10.3389/fmicb.2020.00667)
Supplement: Data Sheet 1 — SRA/ENA accession numbers and phenotypes to four first-line drugs. [file Data_Sheet_2.pdf]

Multi-Label Random Forest Model for Tuberculosis Drug Resistance Classification and Mutation Ranking

SUPPLEMENTARY A

These 23 genes were previously identified to be associated with resistance of examined anti-TB drugs in the literature. The purpose of selecting these 23 genes is to make our results comparable with the baseline model, direct association. The next step as the future work will be to analyse the whole genome sequences including positions outside these genes. Including all genetic variants will increase the feature space to 200k-300k. RF models usually cannot deal with very high dimensional data in presence of dependencies as in the TB data.

Table S1. A list of 23 candidate genes and their associated drugs.

Genes	Relevant drug
ahpC, fabG1, inhA, katG, ndh	INH
rpoB	RIF
embA, embB, embC, embR, iniA, iniC, manB, rmlD	EMB
pncA, rpsA	PZA
gyrA, gyrB	OFX, MOX, CIP
rpsL, gidB, rrs, tlyA	SM
gidB, rrs, tlyA	AK and CAP
gidB, rrs, tlyA, eis	KAN

SUPPLEMENTARY B

In each node of Figure S1, value has four rows indicating the resistant/susceptible numbers for EMB, INH, RIF and PZA respectively while it only has one row in Figure S2, showing the resistant/susceptible split for EMB.

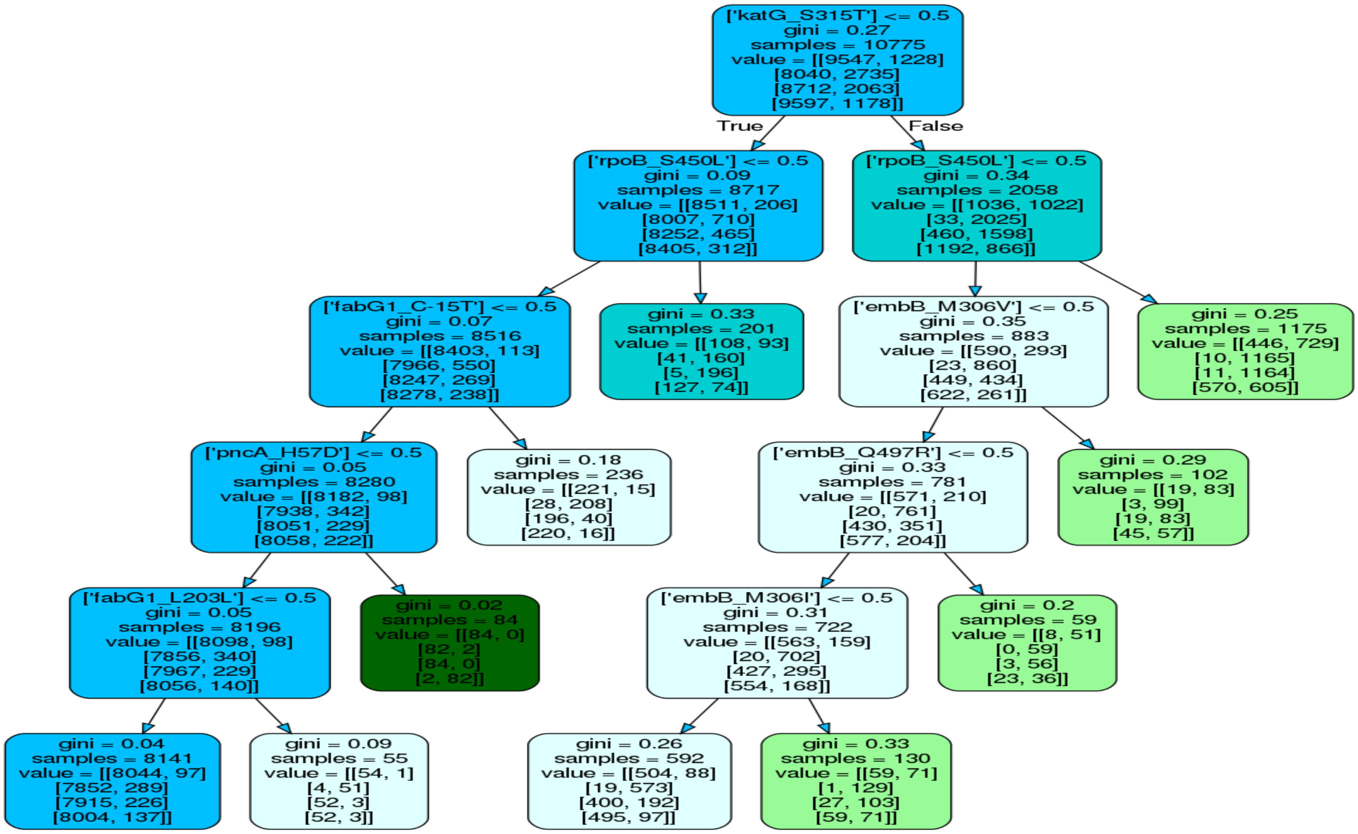

Figure S1. A decision tree from the forest trained considering MLRF + F3. Only 10 leaf nodes presented here. sample means the total number of isolates meeting the criteria indicated at each node and value denotes the size of two classes at this node. For example, samples = 883 with a value of [{590, 293},...] associated to *embB*_M306V shows that from the total 883 samples, 590 are susceptible and 293 are resistant to EMB. value has four rows indicating the resistant/susceptible numbers for EMB, INH, RIF and PZA, respectively.

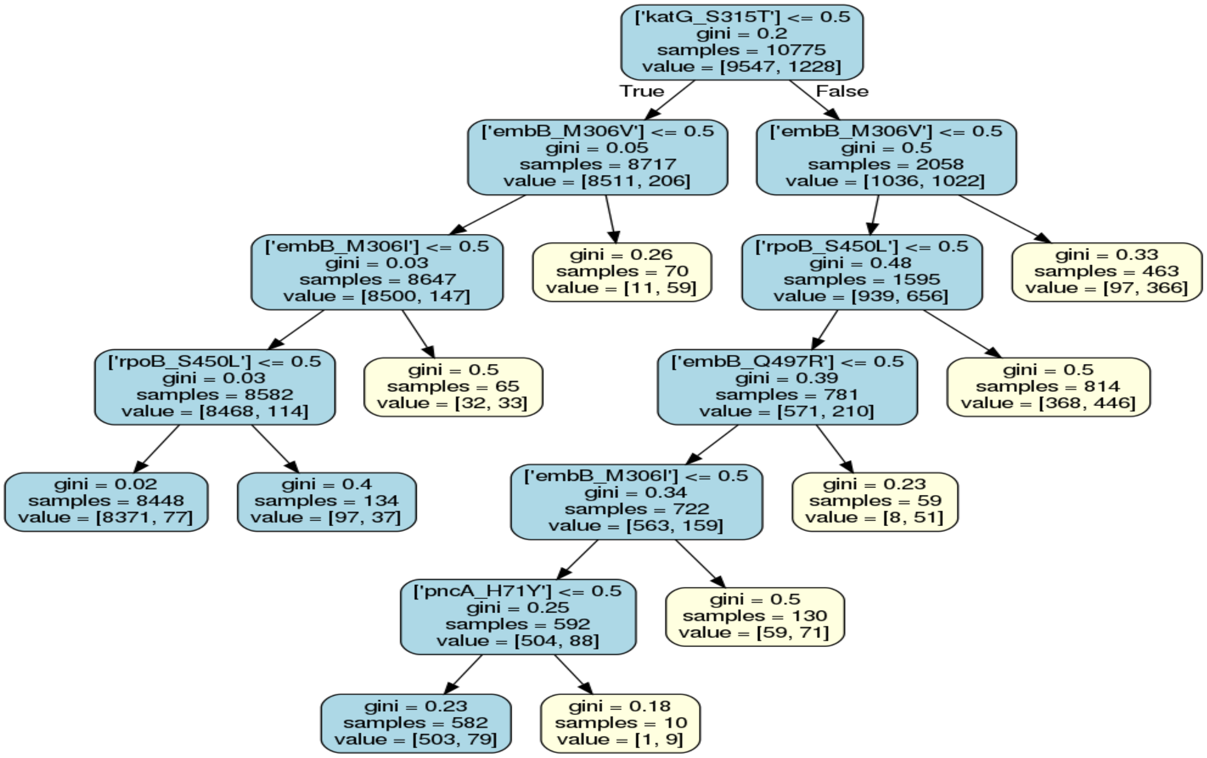

Figure S2. A decision tree from the forest trained considering SLRF + F3 for EMB prediction. Only 10 leaf nodes presented here.

SUPPLEMENTARY C

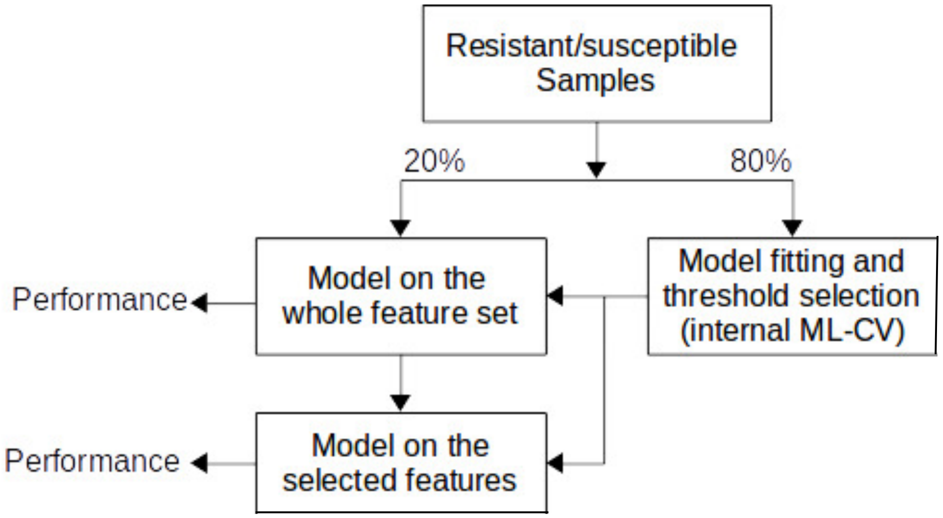

Figure S3. Flowchart for testing MLRF and SLRF classifiers. In each iteration, 20% of the dataset was used as the test set and the remaining 80% of the data as the training set. The models were trained: (1) on the whole feature space; and (2) most highly-ranked features.

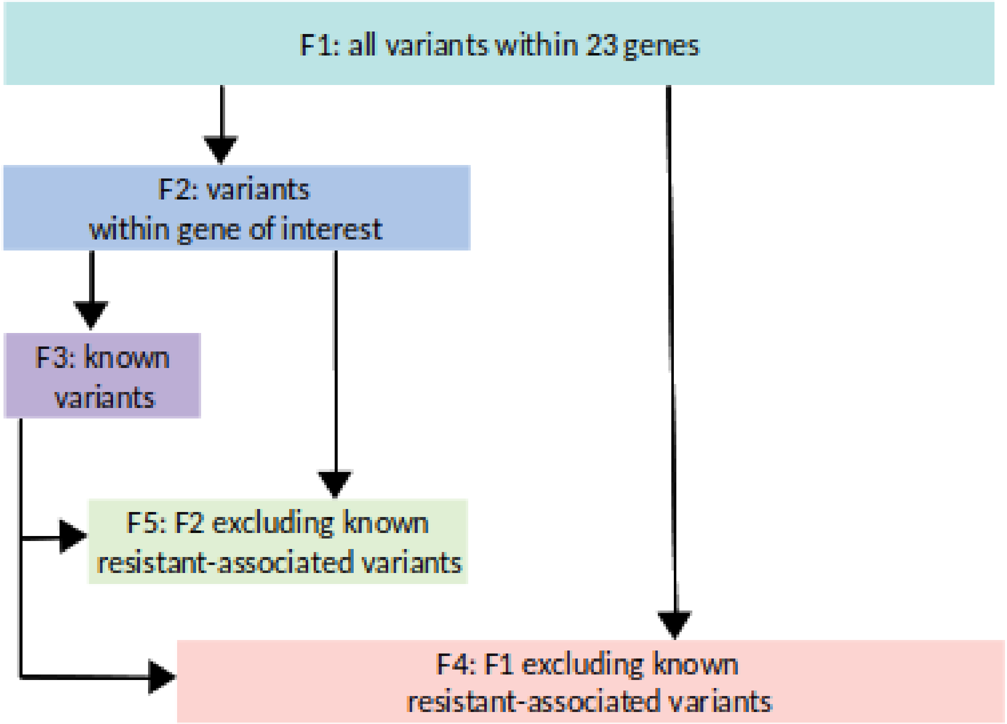

Figure S4. Five feature sets were considered: [F1] the baseline feature space of all variants found within 23 candidate genes (N = 5918); [F2] as a subset of F1 includes only drug-associated genes for a particular drug (N = 3366 Supplementary B); [F3] known variants for all first-line drugs (N = 1874); [F4] and [F5] are obtained by dropping isolates with any known resistance-associated mutations from F1 and F2 respectively – that is, F4 and F5 allows us to investigate whether phenotypically resistant isolates without well-known resistance mutations can be identified from other sequence variations (N = 4755 and 2417, respectively).

Table S2. The phenotype profile for first-line drugs; the number of isolates that are resistant or susceptible. All missing labels were removed from our analysis.

Drug	INH	EMB	RIF	PZA
Resistant	2735	1228	2063	1178
Susceptible	8040	9547	8712	9597

Table S3. The phenotype profile for second-line drugs; the number of isolates that are resistant, susceptible, or missing.

Drug	SM	KAN	AK	CAP	CIP	OFX	MOX
Susceptible	5105	1925	2690	2741	529	2618	1249
Resistant	1729	242	273	315	77	458	262
Total tested	6834	2167	2963	3056	606	3076	1511
Missing	6568	11235	10439	10346	12796	10326	11891

Table S4. Frequency (%) of each label combination; susceptibility (S) or Resistance (R) to PZA, EMB, RIF, and INH, respectively.

Phenotypes	Frequency
SSSS	69.85
SSSR	6.02
SSRS	1.23
SSRR	6.73
SRSS	0.20
SRSR	0.31
SRRS	0.74
SRRR	5.65
RSSS	1.11
RSSR	0.32
RSRS	0.06
RSRR	2.29
RRSS	0.01
RRSR	0.20
RRRS	0.01
RRRR	5.23

SUPPLEMENTARY D

Table S5. Comparing the performance of DA, MLRF and SLRF for INH, EMB, RIF, PZA, MDR-TB, and FDR-TB for feature sets F1-F5.

		INH			EMB		
Feature set	Method	Sensitivity	Specificity	AUC	Sensitivity	Specificity	AUC
	DA	91.15 ± 1.19	98.96 ± 0.25	95.05 ± 0.60	85.10 ± 1.79	94.91 ± 0.38	90.00 ± 0.97
F1	MLRF	94.29 ± 0.74	94.32 ± 1.58	94.30 ± 0.80	91.75 ± 1.81	91.58 ± 0.77	**91.70 ± 0.75**
	SLRF	94.48 ± 0.89	94.14 ± 1.40	94.31 ± 0.59	91.76 ± 2.45	91.65 ± 0.92	91.70 ± 1.04
F2	MLRF	93.76 ± 0.78	95.36 ± 0.54	94.56 ± 0.47	91.16 ± 1.65	91.72 ± 0.48	91.44 ± 0.81
	SLRF	93.65± 0.93	95.39 ± 0.54	94.52 ± 0.53	91.51 ± 1.92	91.84 ± 0.63	91.67 ± 0.91
F3	MLRF	93.76 ± 0.80	97.79 ± 0.35	**96.01 ± 0.47**	90.72 ± 1.49	92.50 ± 0.39	91.61 ± 0.75
	SLRF	93.19 ± 1.03	97.80 ± 0.36	95.49 ± 0.50	90.91 ± 1.53	92.52 ± 0.38	**91.70 ± 0.75**
F4	MLRF	15.87 ± 5.82	97.44 ± 0.83	56.65 ± 2.85	3.50 ± 5.73	99.39 ± 0.36	51.44 ± 2.85
	SLRF	15.49 ± 5.81	97.29 ± 0.80	56.39 ± 2.79	3.49 ± 5.23	99.44 ± 0.30	51.47 ± 2.59
F5	MLRF	12.72 ± 4.64	98.19 ± 0.42	55.45 ± 2.27	2.62 ± 4.43	99.70 ± 0.19	51.16 ± 2.21
	SLRF	13.19 ± 5.93	98.17 ± 0.58	55.68 ± 2.99	3.19 ± 5.09	99.68 ± 0.20	51.43 ± 2.53
		RIF			PZA		
Feature set	Method	Sensitivity	Specificity	AUC	Sensitivity	Specificity	AUC
	DA	91.52 ± 1.34	98.68 ± 0.21	95.10 ± 0.65	43.21 ± 2.72	98.58 ± 0.23	70.89 ± 1.35
F1	MLRF	93.88 ± 1.26	94.25 ± 1.38	94.07 ± 0.75	86.86 ± 1.98	90.44 ± 0.73	88.65 ± 1.08
	SLRF	93.93 ± 1.27	93.74 ± 1.23	93.84 ± 0.56	87.27 ± 1.74	90.71 ± 0.72	**88.99 ± 0.84**
F2	MLRF	91.16 ± 0.91	92.60 ± 0.85	91.88 ± 0.58	85.75 ± 2.93	88.41 ± 1.04	87.08 ± 1.23
	SLRF	91.12 ± 1.28	92.53 ± 0.78	91.83 ± 0.68	86.08 ± 1.97	88.22 ± 0.91	87.15 ± 0.98
F3	MLRF	93.16 ± 0.80	98.02 ± 0.32	**96.00 ± 0.40**	85.03 ± 2.02	90.89 ± 0.55	87.96 ± 0.92
	SLRF	92.30 ± 1.16	97.89 ± 0.69	95.09 ± 0.60	85.15 ± 2.02	90.85 ± 0.45	88.00 ± 1.02
F4	MLRF	9.91 ± 5.64	97.99 ± 0.70	53.95 ± 2.74	12.35 ± 7.19	98.46 ± 0.60	55.41 ± 3.63
	SLRF	10.37 ± 6.80	97.82 ± 0.89	54.10 ± 3.31	12.79 ± 8.25	98.52 ± 0.45	55.65 ± 4.08
F5	MLRF	3.26 ± 3.57	99.01 ± 0.42	51.13 ± 1.74	54.10 ± 5.60	98.41 ± 0.43	76.26 ± 2.73
	SLRF	5.18 ± 4.61	98.91 ± 0.53	52.05 ± 2.27	54.27 ± 5.83	98.37 ± 0.50	76.32 ± 2.86
		FDR-TB			MDR-TB		
Feature set	Method	Sensitivity	Specificity	AUC	Sensitivity	Specificity	AUC
	DA	37.34 ± 3.97	98.59 ± 0.22	67.96 ± 1.99	89.84 ± 1.34	99.12 ± 0.17	94.48 ± 0.69
F1	MLRF	87.58 ± 2.79	92.98 ± 0.45	**90.28 ± 1.23**	95.20 ± 1.07	95.04 ± 1.14	95.12 ± 0.73
	SLRF	87.27 ± 2.69	93.10 ± 0.50	90.19 ± 1.27	95.27 ± 1.00	94.83 ± 0.90	95.05 ± 0.58
F2	MLRF	85.29 ± 2.88	92.86 ± 0.64	89.07 ± 1.44	93.60 ± 1.12	93.83 ± 0.97	93.71 ± 0.52
	SLRF	86.35 ± 2.55	92.87 ± 0.52	89.61 ± 1.29	93.53 ± 1.43	93.76 ± 0.88	93.64 ± 0.55
F3	MLRF	85.76 ± 3.13	92.62 ± 0.74	89.19 ± 1.47	93.70 ± 0.76	97.45 ± 0.36	**95.58 ± 0.41**
	SLRF	85.90 ± 3.40	92.58 ± 0.83	89.24 ± 1.51	93.77 ± 1.11	97.31 ± 0.84	95.54 ± 0.67
F4	MLRF	8.67 ± 13.43	99.94 ± 0.06	54.31 ± 6.72	8.92 ± 7.79	99.30 ± 0.41	54.11 ± 3.81
	SLRF	8.83 ± 13.68	99.95 ± 0.07	54.39 ± 6.83	10.05 ± 8.04	99.29 ± 0.40	54.67 ± 3.94
F5	MLRF	6.67 ± 13.02	99.93 ± 0.07	53.30 ± 6.51	5.14 ± 5.58	99.49 ± 0.28	52.32 ± 2.76
	SLRF	7.83 ± 12.74	99.92 ± 0.08	53.88 ± 6.38	7.74 ± 6.99	99.39 ± 0.41	53.56 ± 3.48

Most highly-ranked mutations and their importance are demonstrated in the following tables and figures for MLRF and SLRF and F1-F5 feature sets.

Table S6. A summary of top 10 mutations ranked by F1 + MLRF or SLRF. Known resistance-associated mutations to INH, EMB, RIF, and PZA drugs are indicated by ^{I}, ^{E}, ^{R} and ^{P}, respectively. Lineage-associated mutations are indicated by * and unknown mutations in boldface.

MLRF + F1	*katG*_S315T^{I}, *rpoB*_S450L^{R}, *fabG1*_C-15T^{I}, *embB*_M306V^{E}, *rrs*_A1401G, *rpoB*_D435V^{R}, *rpoB*_H445Y^{R}, *gyrA*_Q613E, *pncA*_H57D^{P}, *embB*_Q497R^{E}
SLRF + F1 (INH)	*katG*_S315T^{I}, *fabG1*_C-15T^{I}, *fabG1*_L203L^{I}, *rpoB*_S450L^{R}, *katG*_S315N^{I}, *fabG1*_G-17T^{I}, *rpoB*_V170F^{R}, *embB*_M306V^{E}, *rpoB*_D435V^{R}, *embB*_M306V^{E}
SLRF + F1 (EMB)	*katG*_S315T^{I}, *rpoB*_S450L^{R}, *embB*_M306V^{E}, *embB*_M306I^{E}, *embB*_Q497R^{E}, *rrs*_A1401G, *rpsL*_K43R, *gyrA*_Q613E, *embB*_G406S^{E}, *eis*_C-12T
SLRF + F1 (RIF)	*rpoB*_S450L^{R}, *katG*_S315T^{I}, *rpoB*_H445Y^{R}, *rpoB*_D435V^{R}, *rpoB*_H445D^{R}, *rpoB*_V170F^{R}, *rpoB*_S450W^{R}, *embB*_M306V^{E}, *embB*_Q497R^{E}, *gyrA*_Q613E
SLRF + F1 (PZA)	*katG*_S315T^{I}, *pncA*_H57D^{P}, *rpoB*_S450L^{R}, *embB*_M306V^{E}, *rrs*_A1401G, *embB*_M306I^{E}, *rpsL*_K43R, *pncA*_A-11G^{P}, *embB*_G406A^{E}, *pncA*_I6L^{P}
SLRF + F1 (FDR)	*katG*_S315T^{I}, *rpoB*_S450L^{R}, *embB*_M306V^{E}, *rrs*_A1401G, *embB*_Q497R^{E}, *embB*_M306I^{E}, *rpsL*_K43R, ***pncA*_H51D**, *pncA*_A-11G^{P}, *gyrA*]_Q613E
SLRF + F1 (MDR)	*rpoB*_S450L^{R}, *katG*_S315T^{I}, *rpoB*_D435V^{R}, *rpoB*_H445Y^{R}, *rpoB*_V170F^{R}, *rpoB*_H445D^{R}, *embB*_M306V^{E}, *embB*_Q497R^{E}, *rpoB*_S450W^{R}, *gyrA*_Q613E

Table S7. A summary of top 10 mutations ranked by F2 + MLRF or SLRF. Known resistance-associated mutations to INH, EMB, RIF, and PZA drugs are indicated by ^{I}, ^{E}, ^{R} and ^{P}, respectively. Lineage-associated mutations are indicated by * and unknown mutations in boldface.

MLRF + F2	*katG*_S315T^{I}, *rpoB*_S450L^{R}, *fabG1*_C-15T^{I}, *embB*_M306V^{E}, *rpoB*_D435V^{R}, *embB*_M3016I^{E}, *embB*_Q497R^{E}, *rpoB*_H445Y^{R}, *rpoB*_H445D^{R}, *fabG1*_L203L^{I}
SLRF + F2 (INH)	*katG*_S315T^{I}, *fabG1*_C-15T^{I}, *fabG1*_L203L^{I}, *rpoB*_S450L^{R}, *katG*_S315N^{I}, *fabG1*_G-17T^{I}, *rpoB*_V170F^{R}, *rpoB*_D435V^{R}, *embB*_M306I^{E}, ***embC*_R927R**
SLRF + F2 (EMB)	*katG*_S315T^{I}, *rpoB*_S450L^{R}, *embB*_M306V^{E}, *embB*_M306I^{E}, *embB*_Q497R^{E}, *embB*_G406S^{E}, *fabG1*_C-15T^{I}, ***embC*_R927R**, *rpoB*_I491F^{R}, *embB*_C-12T^{E}
SLRF + F2 (RIF)	*rpoB*_S450L^{R}, *katG*_S315T^{I}, *rpoB*_H445Y^{R}, *rpoB*_D435V^{R}, *rpoB*_H445D^{R}, *rpoB*_V170F^{R}, *rpoB*_S450W^{R}, *embB*_M306V^{E}, *embB*_Q497R^{E}, *embB*_M306I^{E}
SLRF + F2 (PZA)	*katG*_S315T^{I}, *embB*_M306V^{E}, *rpoB*_S450L^{R}, *rpsA*_A440T*, *embB*_N13S^{E}, *embB*_M306I^{E}, *rpoB*_E761D^{R}, *embB*_Q497R^{E}, *embB*_G406A^{E}, *rpoB*_I491F^{R}
SLRF + F2 (FDR)	*katG*_S315T^{I}, *rpoB*_S450L^{R}, *embB*_M306V^{E}, *embB*_Q497R^{E}, *embB*_M306I^{E}, *rpoB*_I491F^{R}, *embB*_G406A^{E}, *embA*_C-12T^{E}, *fabG1*_C-15T^{I}, ***embC*_R927R**
SLRF + F2 (MDR)	*rpoB*_S450L^{R}, *katG*_S315T^{I}, *rpoB*_D435V^{R}, *rpoB*_H445Y^{R}, *embB*_M306V^{E}, *rpoB*_V170F^{R}, *rpoB*_H445D^{R}, *embB*_M306I^{E}, *rpoB*_S450W^{R}, *embB*_Q497R^{E}

Table S8. A summary of top 10 mutations ranked by F3 + MLRF or SLRF. Known resistance-associated mutations to INH, EMB, RIF and PZA drugs are indicated by ^{I}, ^{E}, ^{R} and ^{P}, respectively. Lineage-associated mutations are indicated by * and unknown mutations in boldface.

MLRF + F3	*katG*_S315T^{I}, *rpoB*_S450L^{R}, *fabG1*_C-15T^{I}, *embB*_M306V^{E}, *rpoB*_D435V^{R}, *embB*_Q497R^{E}, *embB*_M3016I^{E}, *rpoB*_H445Y^{R}, *pncA*_H57D^{P}, *rpoB*_I491F^{R}
SLRF + F3 (INH)	*katG*_S315T^{I}, *fabG1*_C-15T^{I}, *fabG1*_L203L^{I}, *rpoB*_S450L^{R}, *katG*_S315N^{I}, *fabG1*_G-17T^{I}, *rpoB*_V170F^{R}, *embB*_M306V^{E}, *embB*_M306I^{E}, *rpoB*_D435V^{R}
SLRF + F3 (EMB)	*katG*_S315T^{I}, *rpoB*_S450L^{R}, *embB*_M306V^{E}, *embB*_M306I^{E}, *embB*_Q497R^{E}, *embB*_G406S^{E}, *fabG1*_C-15T^{I}, *embA*_C-12T^{E}, *rpoB*_I491F^{R}, *embB*_G406A^{E}
SLRF + F3 (RIF)	*rpoB*_S450L^{R}, *katG*_S315T^{I}, *rpoB*_H445Y^{R}, *rpoB*_D435V^{R}, *rpoB*_H445D^{R}, *rpoB*_V170F^{R}, *rpoB*_S450W^{R}, *embB*_M306I^{E}, *embB*_M306V^{E}, *embB*_Q497R^{E}
SLRF + F3 (PZA)	*katG*_S315T^{I}, *pncA*_H57D^{P}, *embB*_M306V^{E}, *rpoB*_S450L^{R}, *embB*_M306I^{E}, *pncA*_A-11G^{P}, *embB*_Q497R^{E}, *embB*_D354A^{E}, *rpoB*_I491F^{R}, *embB*_G406A^{E}
SLRF + F3 (FDR)	*katG*_S315T^{I}, *rpoB*_S450L^{R}, *embB*_M306V^{E}, *embB*_Q497R^{E}, *embB*_M306I^{E}, *rpoB*_I491F^{R}, *pncA*_A-11G^{P}, *fabG1*_C-15T^{I}, *embB*_G406A^{E}, *embA*_Q10P^{E}
SLRF + F3 (MDR)	*rpoB*_S450L^{R}, *katG*_S315T^{I}, *rpoB*_D435V^{R}, *rpoB*_H445Y^{R}, *embB*_M306V^{E}, *rpoB*_V170F^{R}, *rpoB*_H445D^{R}, *embB*_M306I^{E}, *embB*_Q497R^{E}, *rpoB*_S450W^{R}

Table S9. A summary of top 10 mutations ranked by F4 + MLRF or SLRF. Known resistance-associated mutations to INH, EMB, RIF and PZA drugs are indicated by ^{I}, ^{E}, ^{R} and ^{P}, respectively. Lineage-associated mutations are indicated by * and unknown mutations in boldface.

MLRF + F4	*gyrA*_G668D*, ***embA*_L262L**, *eis*_C-12T, ***katG*_N493K**, *rpoB*_I925V, ***rpoB*_M707T**, *gyrA*_L421L, *gyrA*_T836K, *gyrA*_S95T*, ***embC*_L121L**
SLRF + F4 (INH)	*gyrA*_G668D*, *fabG1*_T-8A, *katG*_Q461P, ***katG*_G491S**, *gyrA*_S95T*, ***embR*_S104R**, *katG*_R463L*, ***embA*_L262L**, ***embB*_G18G**, *gyrA*_L421L
SLRF + F4 (EMB)	*gyrA*_L421L, ***embA*_L262L**, ***rpoB*_M707T**, ***embC*_V469M**, *eis*_R181*, *eis*_C-12T, ***manB*_E153G**, ***embC*_L121L**, *gyrA*_G668D*, ***embC*_F324F**
SLRF + F4 (RIF)	*gidB*_R96C, ***embA*_L262L**, *eis*_L191L, *gyrA*_G668D*, ***katG*_N493K**, ***rpoB*_E959K**, *gyrB*_R173R, *eis*_R181*, *eis*_R181*, *eis*_C-12T
SLRF + F4 (PZA)	***embB*_D191D**, ***embA*_L262L**,*gyrA*_G668D*, *gyrB*_R173R, ***embC*_L121L**, ***katG*_W300R**, *gyrB*_E63K, ***rpoB*_S12T**, ***embA*_V558L**, *manB*_G57S^E
SLRF + F4 (FDR)	***embA*_L262L**, *eis*_C-12T, *gyrA*_T836K, *rpoB*_I925V, *gyrA*_G668D*, *gidB*_Y195H, ***rpoB*_A1075A**, *katG*_C-85T^I, *eis*_906_delG, *tlyA*_L11L
SLRF + F4 (MDR)	*gyrA*_G668D*, ***embA*_L262L**, ***katG*_N493K**, ***embB*_K938K**, *eis*_C-12T, *eis*_R181*, ***rpoB*_M707T**, ***manB*_E153G**, *gyrA*_L421L, *gyrA*_T836K

Table S10. A summary of top 10 mutations ranked by F5 + MLRF or SLRF. Known resistance-associated mutations to INH, EMB, RIF and PZA drugs are indicated by ^{I}, ^{E}, ^{R} and ^{P}, respectively. Lineage-associated mutations are indicated by * and unknown mutations in boldface.

MLRF + F5	*rpsA*_A440T*, *embB*_N13S^E, ***embA*_L262L**, ***katG*_N493K**, ***manB*_E153G**, ***embB*_K938K**, ***rpoB*_M707T**, ***embA*_P285P**, *katG*_T271I, ***embB*_D191D**
SLRF + F5 (INH)	*katG*_R463L*, *katG*_Q461P, *fabG1*_T-8A ***embA*_L262L**, ***embR*_S104R**, ***katG*_G491S**, ***rpoB*_A1075A**, *katG*_T271I, ***katG*_N493K**, ***embR*_M1I**
SLRF + F5 (EMB)	***embA*_L262L**, ***embC*_V469M**, ***ndh*_A247A**, ***rpoB*_M707T**, ***embC*_L121L**, ***manB*_E153G**, *rpoB*_I925V, ***embC*_F324F**, ***embA*_L426L**, ***embA*_I905V**
SLRF + F5 (RIF)	***katG*_N493K**, ***embA*_L262L**, ***katG*_S539S**, ***rpoB*_E959K**, ***embB*_K938K**, ***manB*_E153G**, *katG*_Y28*^{I}, ***rpoB*_G1157G**, ***rpoB*_M707T**, *katG*_T271I^I
SLRF + F5 (PZA)	*rpsA*_A440T*, *embB*_N13ST^E, ***embB*_D191D**, *embB*_A680T^E, ***embA*_L262L**, ***iniA*_M1I**, ***embA*_V558L**, ***embR*_A125A**, ***embC*_L121L**, ***katG*_G-28T**
SLRF + F5 (FDR)	***embA*_L262L**, *rpoB*_I925V, ***rpoB*_A1075A**, ***embC*_R927R**, ***embB*_G18G**, ***inhA*_G3G**,***rpsA*_R212R**, ***rpsA*_D35D**, *iniA*_D100G^E, ***embA*_C76C**
SLRF + F5 (MDR)	***katG*_N493K**, ***embA*_L262L**, *katG*_T271I, ***rpoB*_M707T**, ***manB*_E153G**, ***embB*_K938K**, *katG*_Y28*, *rpoB*_I925V, ***rpoB*_A1075A**, *embB*_A454T^E

The red bars demonstrate the features' importance, along with their inter-trees variability (black lines). The displayed weights are normalised (sum of weights is equal to one).

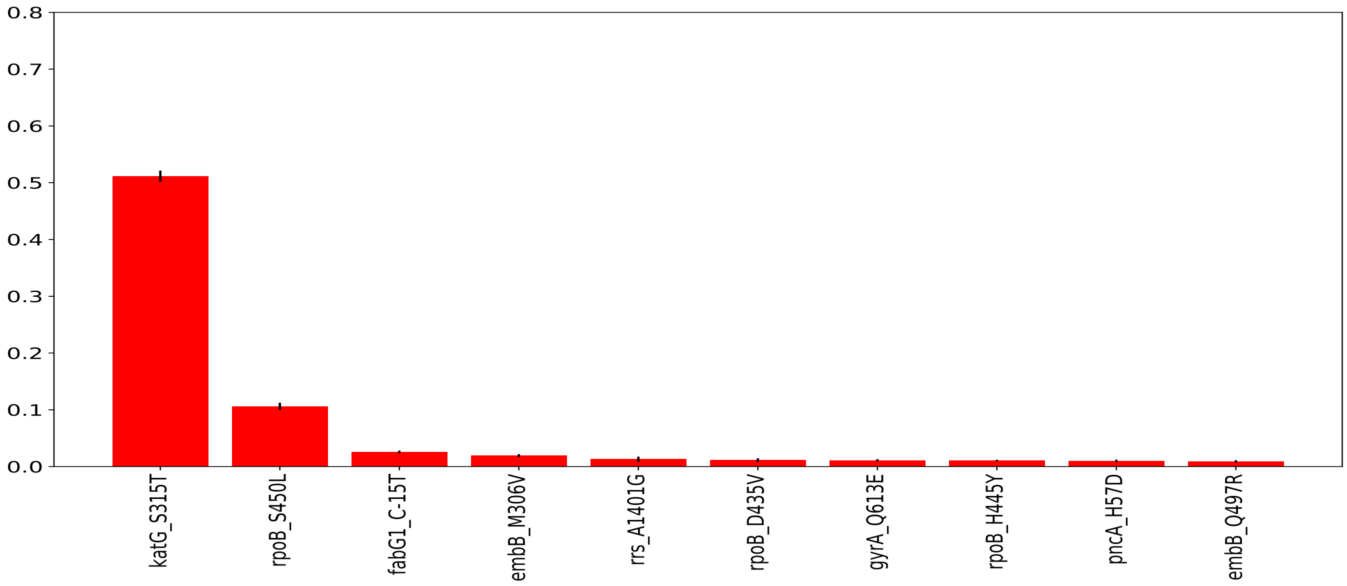

Figure S5. The top 10 mutations ranked by MLRF + F1 for all first-line drugs. *katG*_S315T and *fabG1*_C-15T for INH, *embB*_M306V and *embB*_Q497R for EMB, *rpoB*_S450L, *rpoB*_H445Y, and *rpoB*_D435V for RIF, and *pncA*_H57D for PZA are known resistance-associated markers.

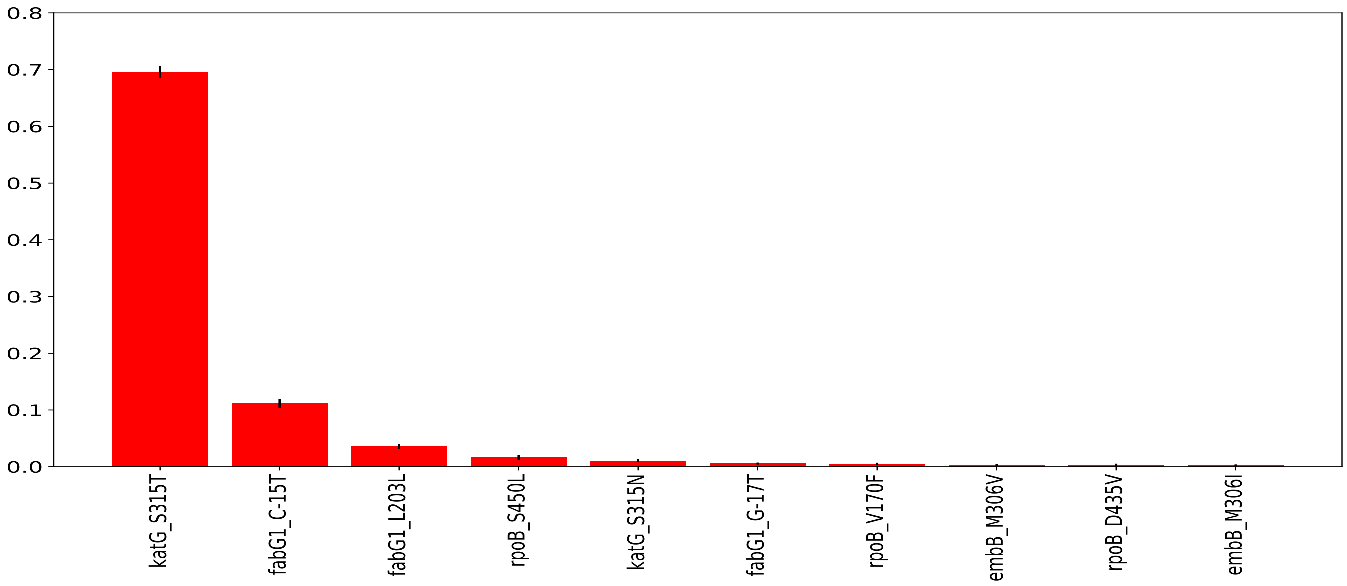

Figure S6. The top 10 mutations ranked by SLRF + F1 for INH. *katG*_S315T, *fabG1*_C-15T, *fabG1*_L203L, *KatG*_S315N, and *fabG1*_G-17T for INH, *embB*_M306V and *embB*_M306I for EMB, and *rpoB*_S450L, *rpoB*_V170F, and *rpoB*_D435V for RIF are known resistance-associated markers.

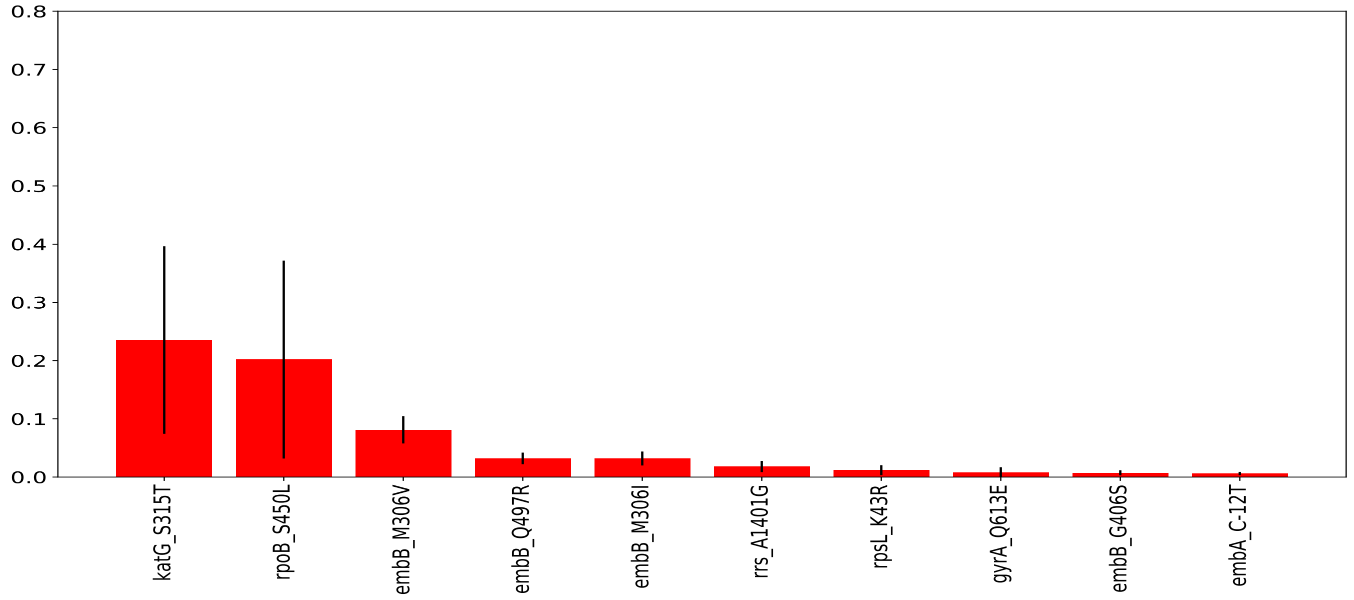

Figure S7. The top 10 mutations ranked by SLRF + F1 for EMB. *katG*_S315T for INH, *embB*_M306V, *embB*_M306I, *embB*_Q497R, and *embB*_G406S for EMB, and *rpoB*_S450L for RIF are known resistance-associated markers.

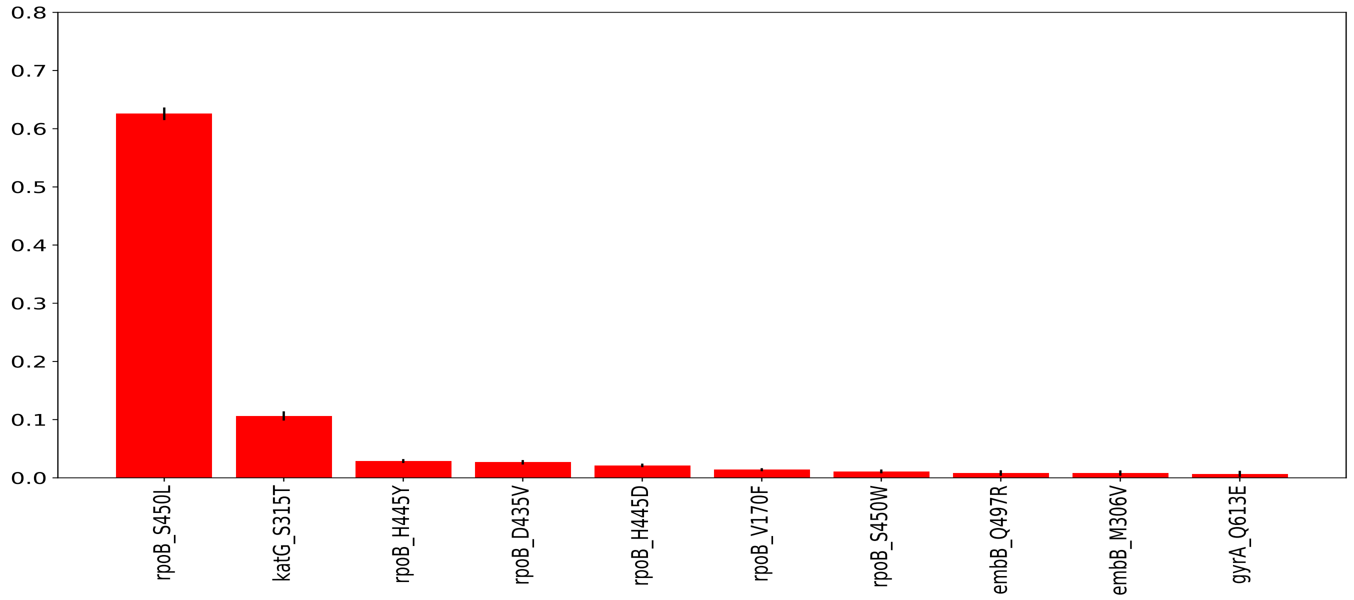

Figure S8. The top 10 mutations ranked by SLRF + F1 for RIF. *katG*_S315T for INH, *embB*_M306V and *embB*_Q497R for EMB, and *rpoB*_S450L, *rpoB*_H445Y, *rpoB*_V170F, *rpoB*_H445D, *rpoB*_S450W, and *rpoB*_D435V for RIF are known resistance-associated markers.

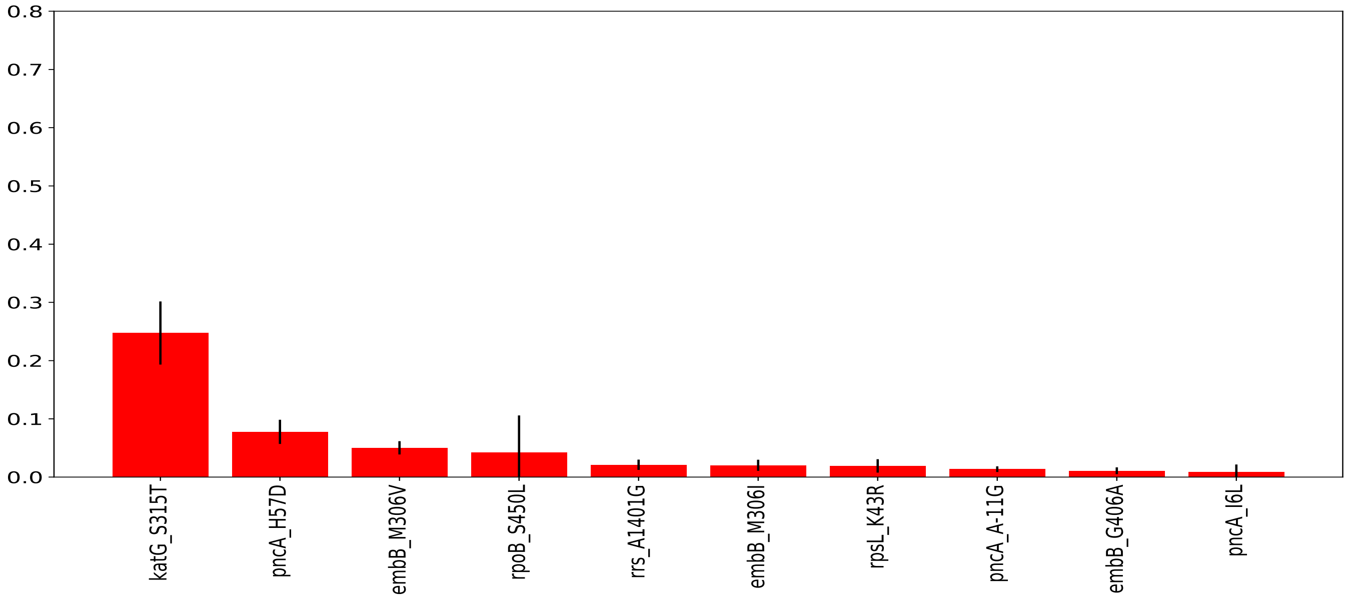

Figure S9. The top 10 mutations ranked by SLRF + F1 for PZA. *katG*_S315T for INH, *embB*_M306V, *embB*_M306I, and *embB*_G406A for EMB, *rpoB*_S450L for RIF, and *pncA*_H57D, *pncA*_A-11G and *pncA*_I6T for PZA are known resistance-associated markers.

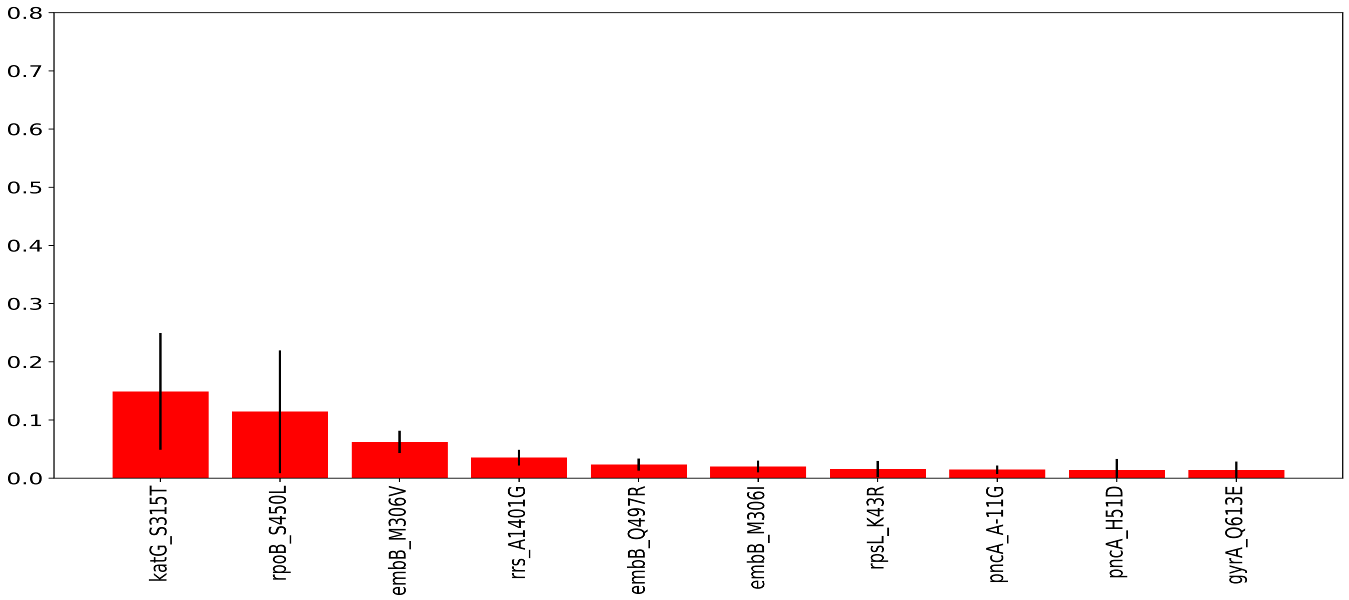

Figure S10. The top 10 mutations ranked by SLRF + F1 for FDR. *katG*_S315T for INH, *embB*_M306V, *embB*_Q497R, and *embB*_M306I for EMB, *rpoB*_S450L for RIF, and *pncA*_A-11G for PZA are known resistance-associated markers. *pncA*_H51D is not in the known catalog that can be a resistant/susceptible-associated marker.

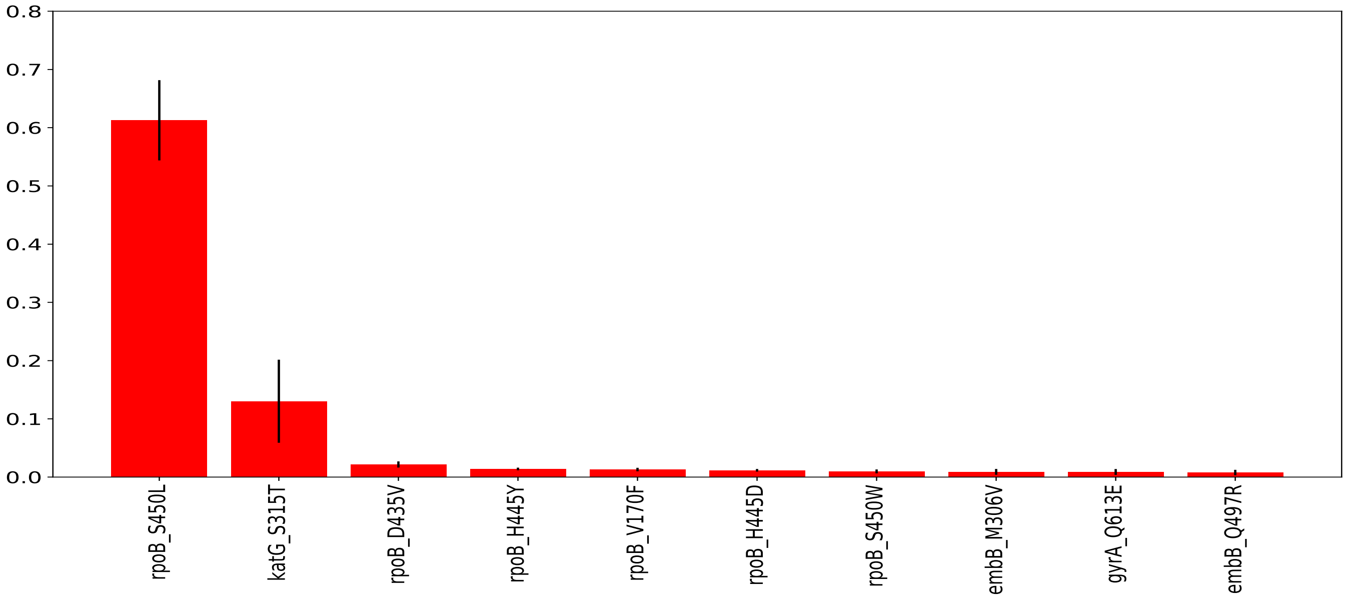

Figure S11. The top 10 mutations ranked by SLRF + F1 for MDR. *katG*_S315T for INH, *embB*_M306V and *embB*_Q497R for EMB, and *rpoB*_S450L, *rpoB*_S450W, *rpoB*_H445Y, *rpoB*_V170F, *rpoB*_H445D, and *rpoB*_D435V for RIF are known resistance-associated markers.

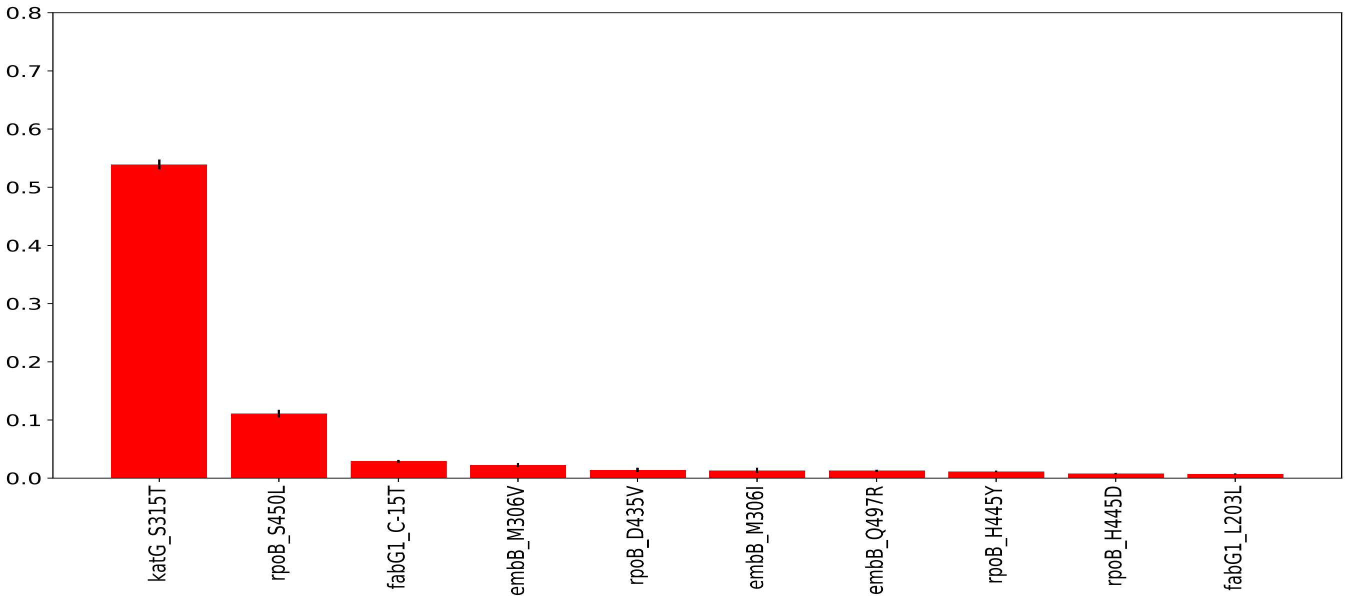

Figure S12. The top 10 mutations ranked by MLRF + F2 for all first-line drugs. *katG*_S315T, *fabG1*_L203L, and *fabG1*_C-15T for INH, *embB*_M306V, *embB*_Q497R, and *embB*_M306I for EMB, *rpoB*_S450L, *rpoB*_H445Y, *rpoB*_H445D, and *rpoB*_D435V for RIF are known resistance-associated markers.

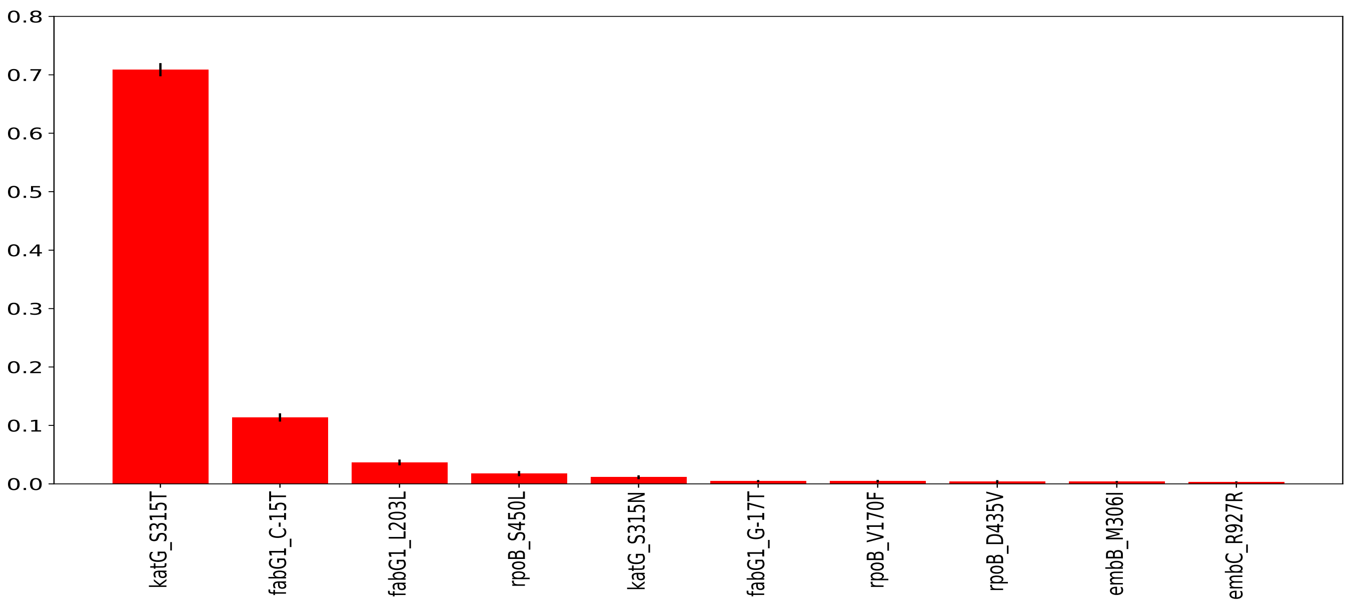

Figure S13. The top 10 mutations ranked by SLRF + F2 for INH. *katG*_S315T, *fabG1*_C-15T, *fabG1*_L203L, *KatG*_S315N, and *fabG1*_G-17T for INH, *embB*_M306V for EMB, and *rpoB*_S450L, *rpoB*_D435V, and *rpoB*_V170F for RIF are known resistance-associated markers. *embC*_R927R is not in the known catalog that can be a resistant/susceptible-associated marker.

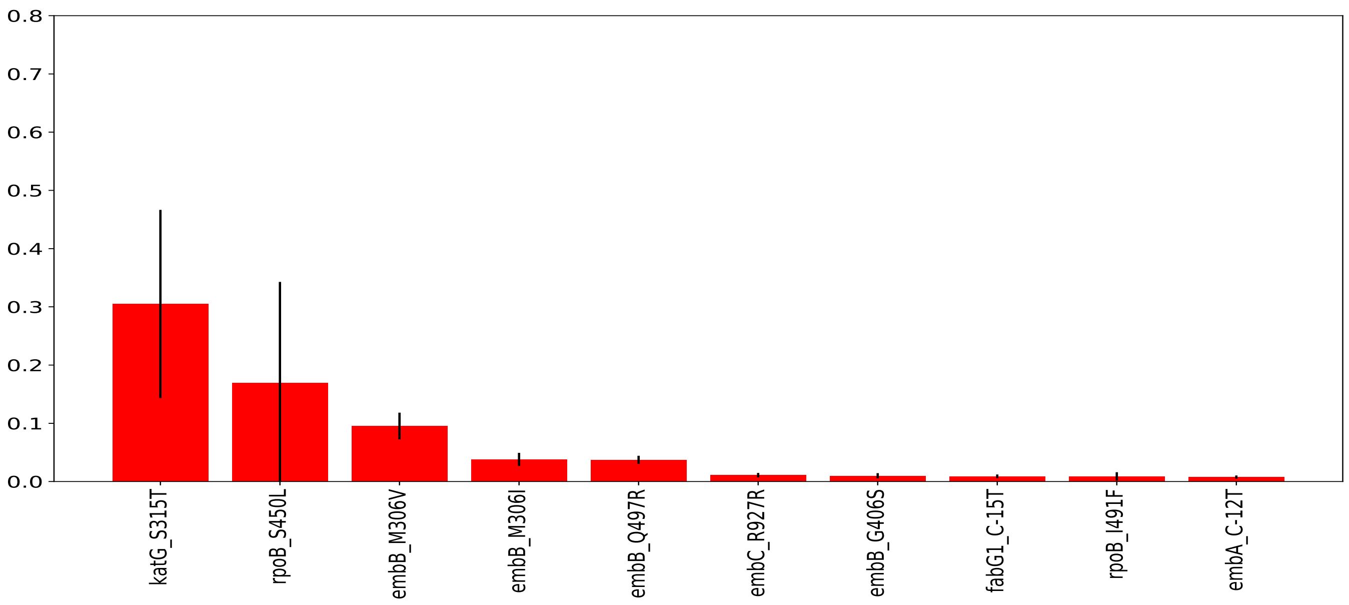

Figure S14. The top 10 mutations ranked by SLRF + F2 for EMB. *katG*_S315T and *fabG1*_C-15T for INH, *embB*_M306V, *embB*_M306I, *embB*_Q497R, and *embB*_G406S and *embB*_C-12T for EMB, and *rpoB*_S450L and *rpoB*_I491F for RIF are known resistance-associated markers. *embC*_R927R is not in the known catalog that can be a resistant/susceptible-associated marker.

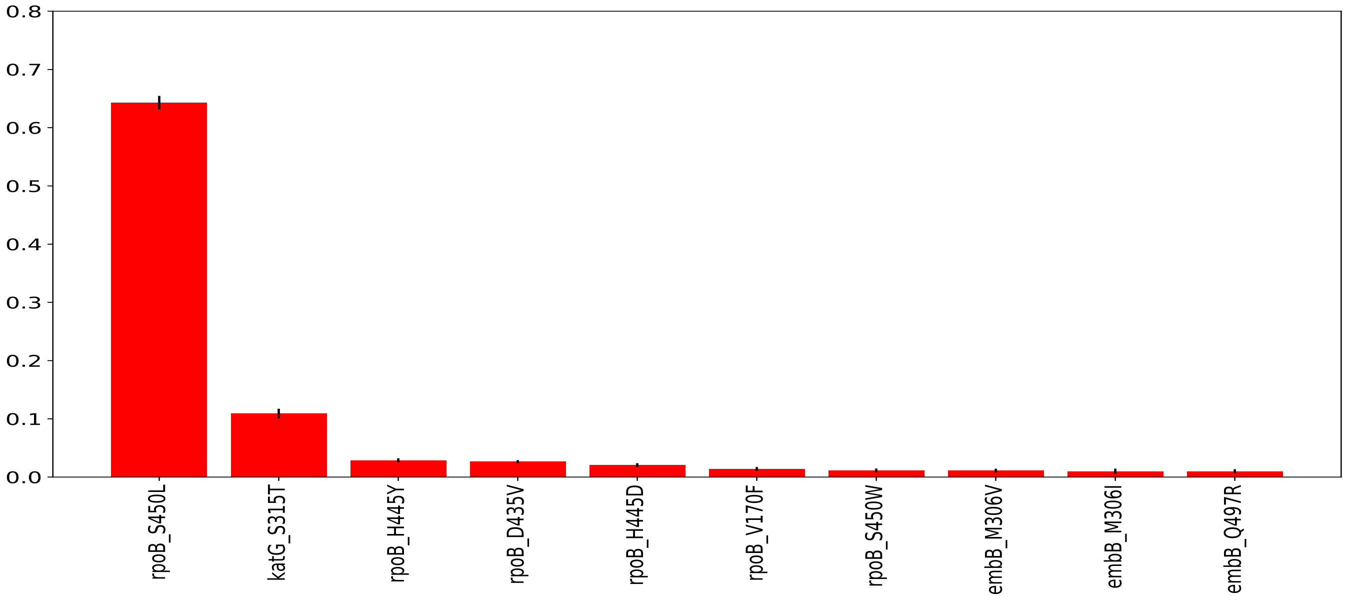

Figure S15. The top 10 mutations ranked by SLRF + F2 for RIF. *katG*_S315T for INH, *embB*_Q497R, *embB*_M306V, and *embB*_M306I for EMB, and *rpoB*_S40DL, *rpoB*_H445Y, *rpoB*_V170F, *rpoB*_H445D, *rpoB*_S450W, and *rpoB*_D435V for RIF are known resistance-associated markers.

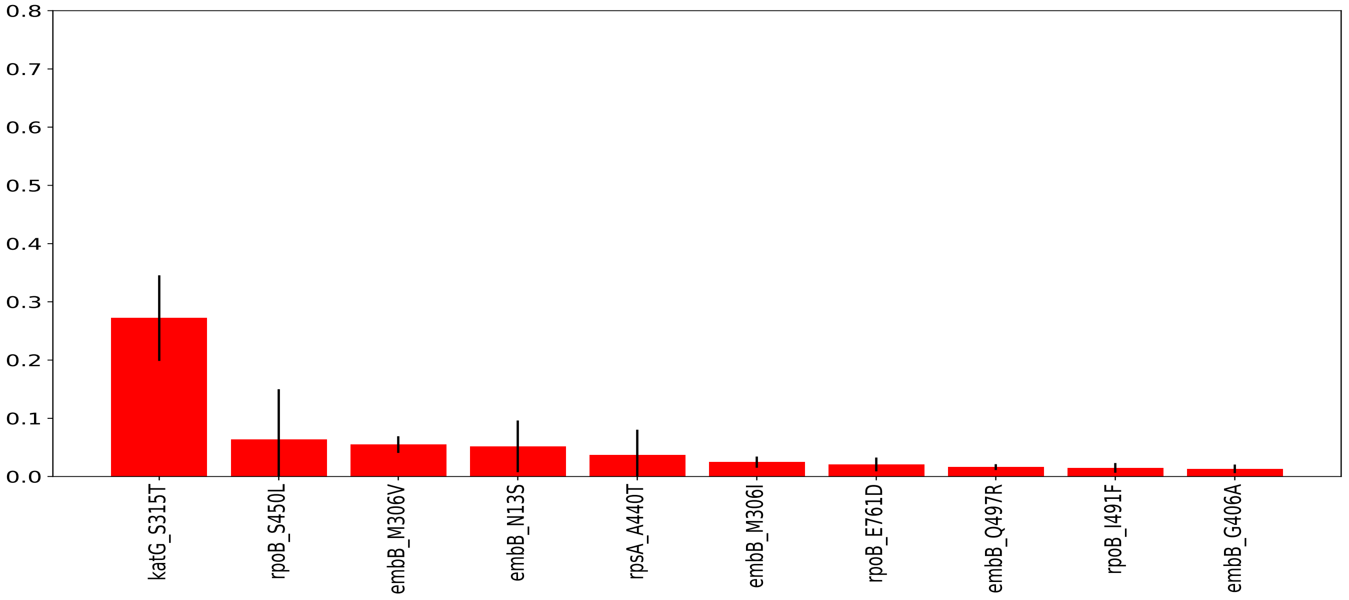

Figure S16. The top 10 mutations ranked by SLRF + F2 for PZA. *katG*_S315T for INH, *embB*_M306V, *embB*_M306I, *embB*_G406A, and *embB*_Q497R for EMB, and *rpoB*_S450L, *rpoB*_E761D, and *rpoB*_I491F for RIF are known resistance-associated markers. *embB*_N13S is a susceptible-associated marker for EMB and *rpsA*_A440T is a lineage-defining marker.

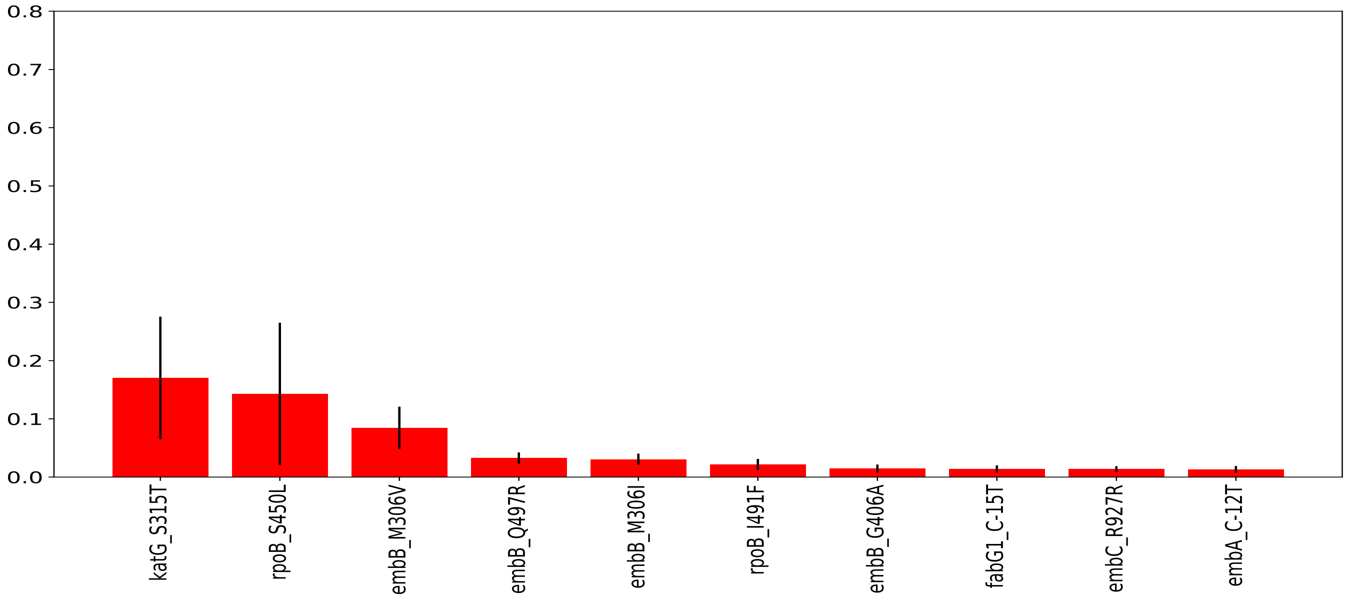

Figure S17. The top 10 mutations ranked by SLRF + F2 for FDR. *katG*_S315T and *fabG1*_C-15T for INH, *embB*_M306V, *embB*_Q497R, *embB*_G406A, *embA*_C-12T, and *embB*_M306I for EMB, and *rpoB*_S450L and *rpoB*_I491F for RIF are known resistance-associated markers. *embA*_R927R is not in the known catalog that can be a resistant/susceptible-associated marker.

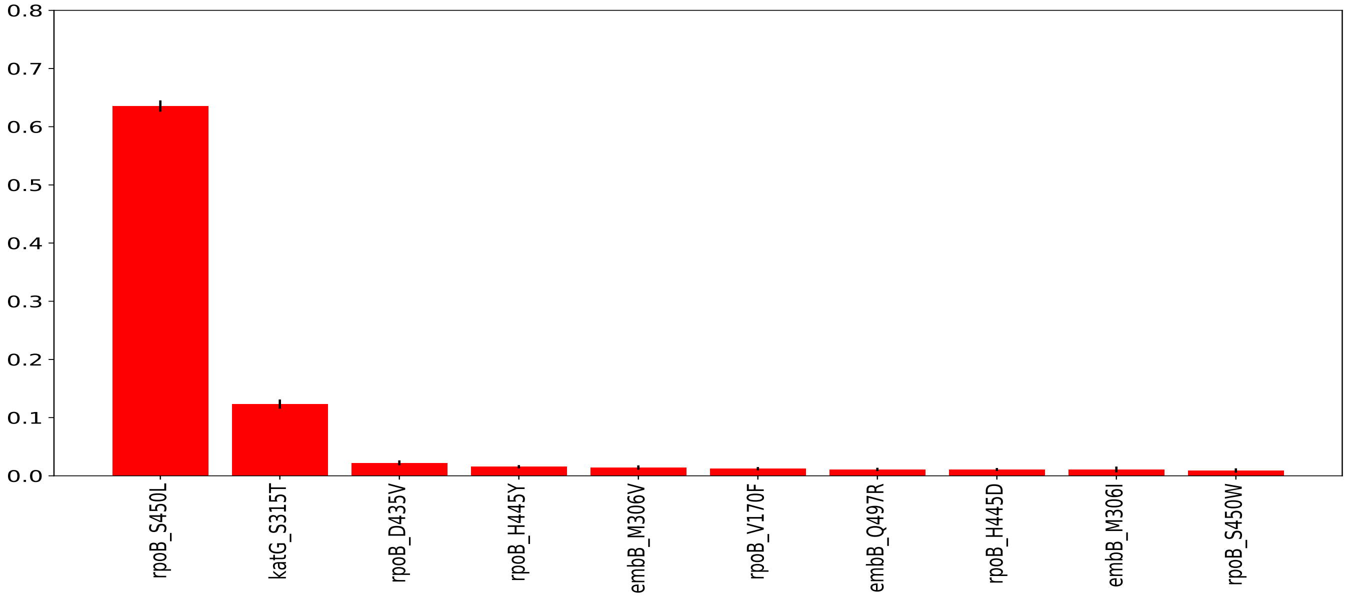

Figure S18. The top 10 mutations ranked by SLRF + F2 for MDR. *katG*_S315T for INH, *embB*_M306V, *embB*_M306I, and *embB*_Q497R for EMB and *rpoB*_S450L, *rpoB*_S450W, *rpoB*_H445Y, *rpoB*_V170F, *rpoB*_H445D, and *rpoB*_D435V for RIF are known resistance-associated markers.

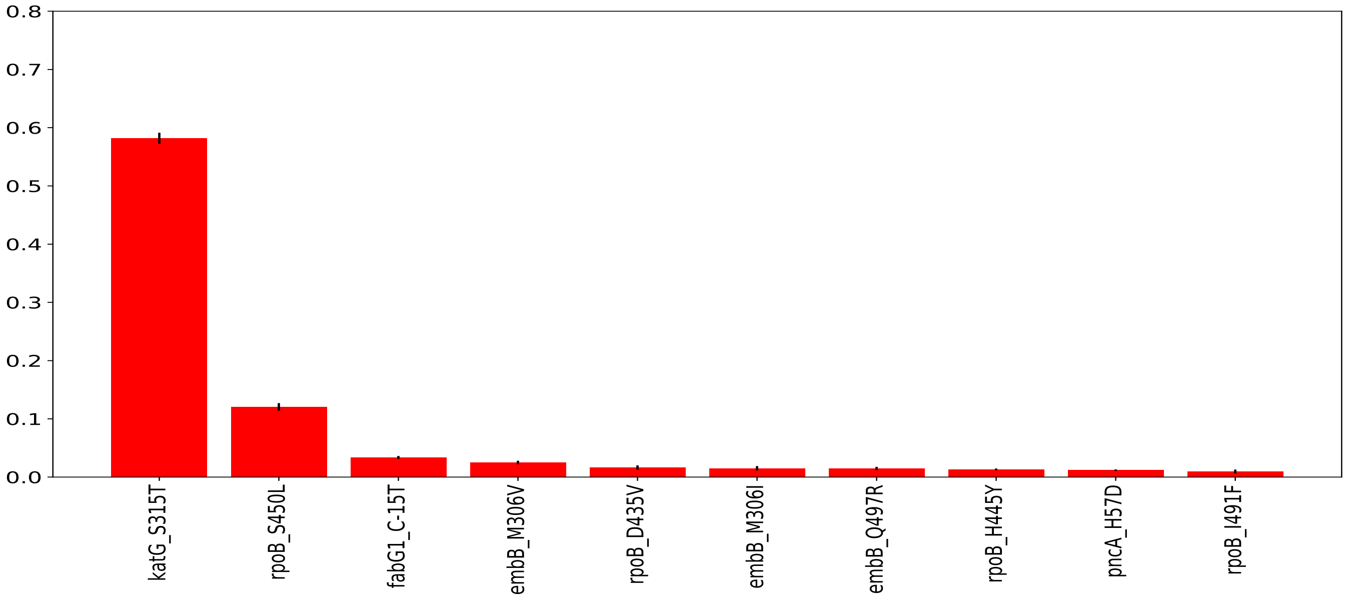

Figure S19. The top 10 mutations ranked by MLRF + F3 for all first-line drugs. *katG*_S315T and *fabG1*_C-15T for INH, *embB*_M306V, *embB*_M306I, and *embB*_Q497R for EMB, *rpoB*_S450L, *rpoB*_H445Y, *rpoB*_I491F, and *rpoB*_D435V for RIF, and *pncA*_H57D for PZA are known resistance-associated markers.

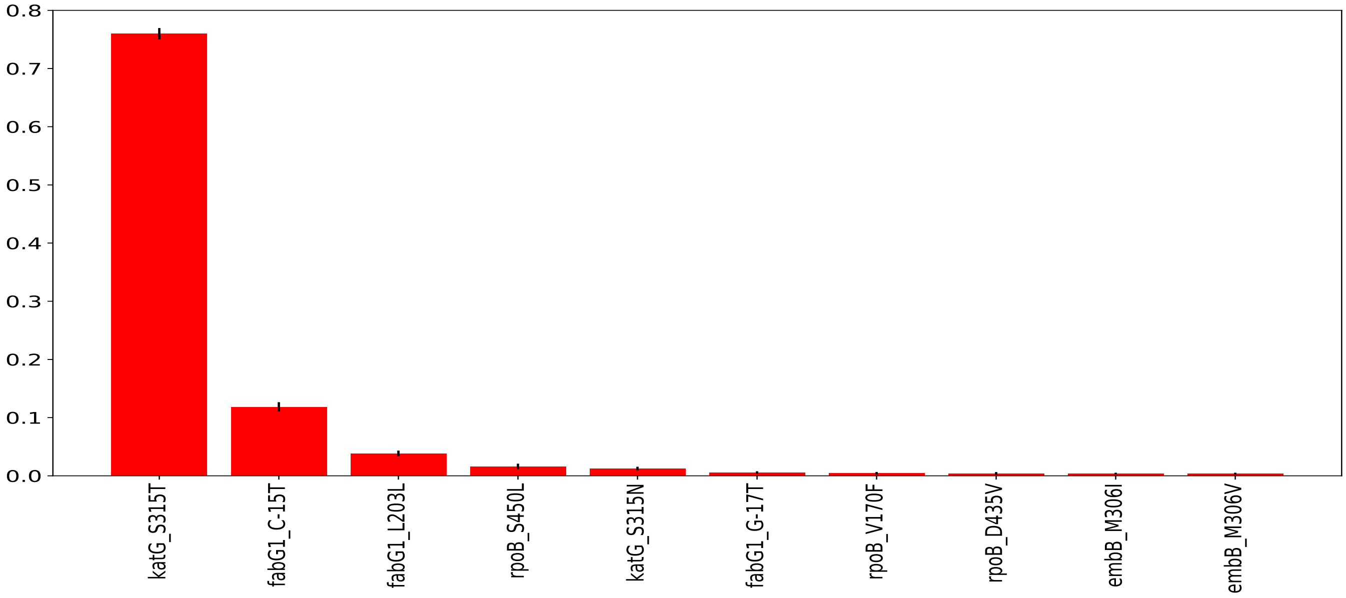

Figure S20. The top 10 mutations ranked by SLRF + F3 for INH. *katG*_S315T, *fabG1*_C-15T, *fabG1*_L203L, *KatG*_S315N, and *fabG1*_G-17T for INH, *embB*_M306V and *embB*_M306I for EMB, and *rpoB*_S450L, *rpoB*_V170F, and *rpoB*_D435V for RIF are known resistance-associated markers.

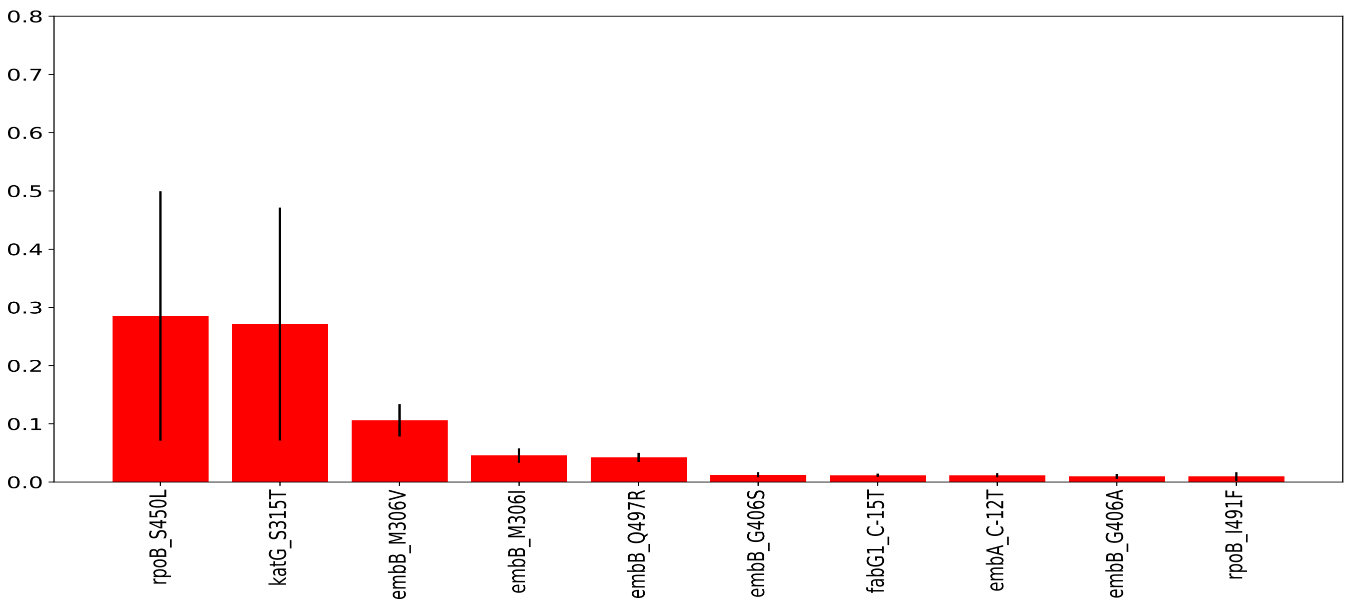

Figure S21. The top 10 mutations ranked by SLRF + F3 for EMB. *katG*_S315T and *fabG1*_C-15T for INH, *embB*_M306V, *embB*_M306I, *embB*_Q497R, *embB*_G406S, and *embB*_G406A for EMB, and *rpoB*_S450L and *rpoB*_I491F for RIF are known resistance-associated markers.

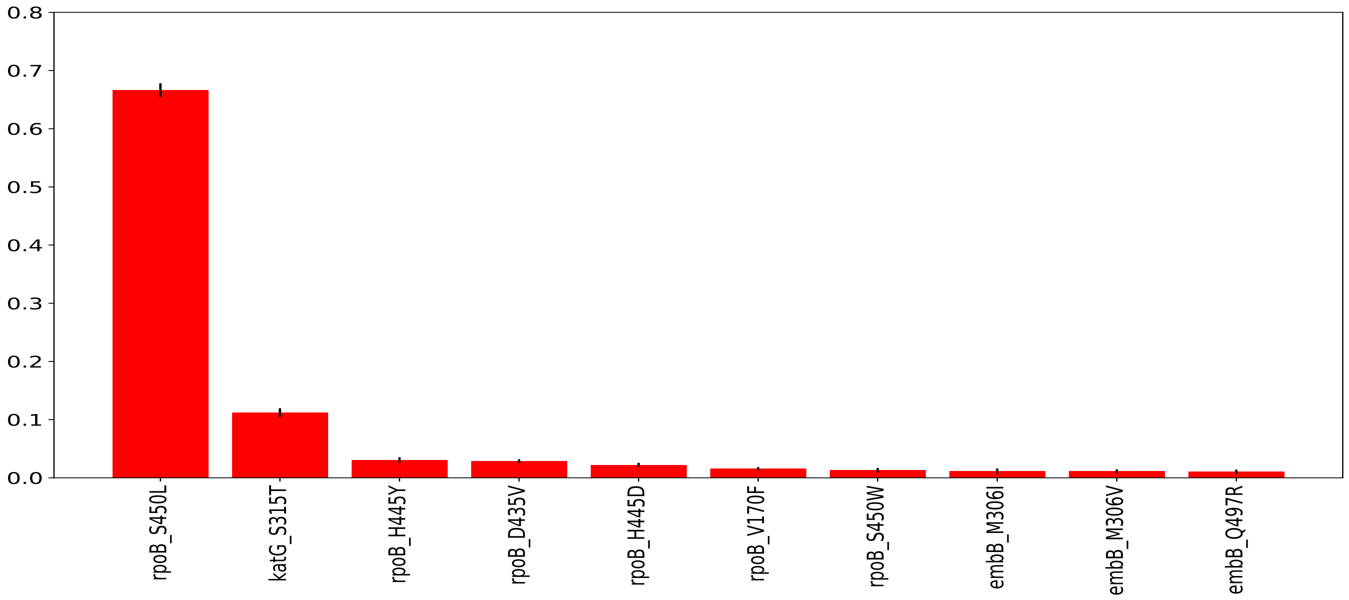

Figure S22. The top 10 mutations ranked by SLRF + F3 for RIF. *katG*_S315T for INH, *embB*_M306V, *embB*_M306I, and *embB*_Q497R for EMB, and *rpoB*_S450L, *rpoB*_H445Y, *rpoB*_V170F, *rpoB*_H445D, *rpoB*_S450W, and *rpoB*_D435V for RIF are known resistance-associated markers.

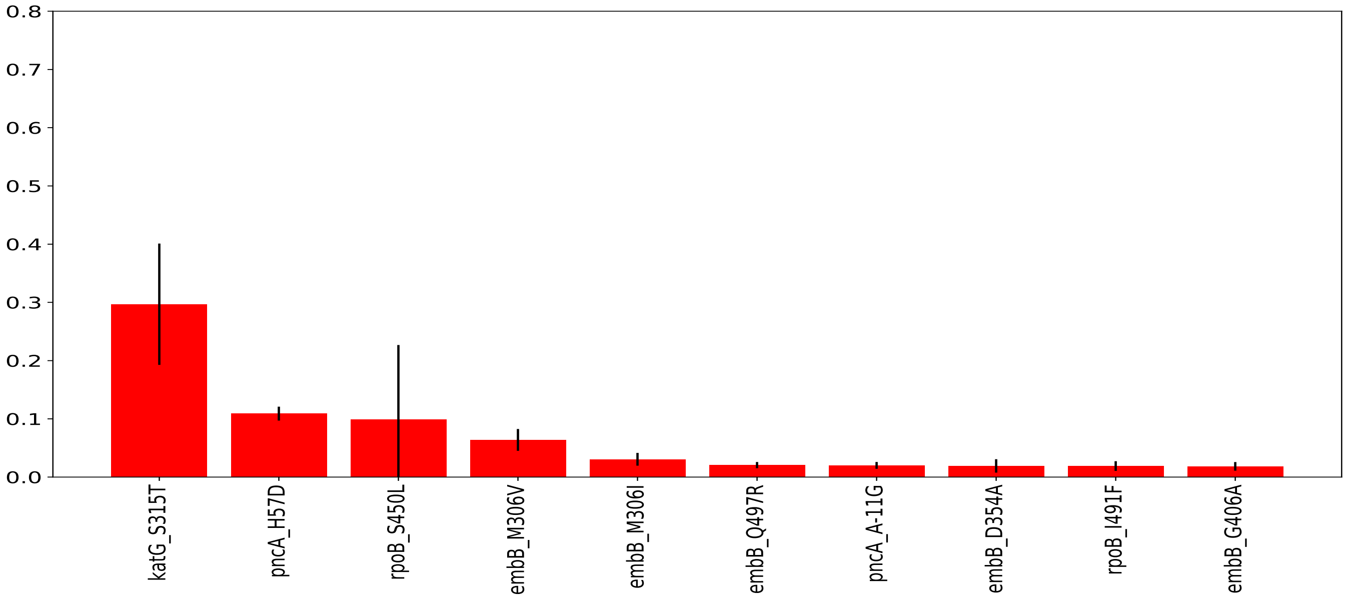

Figure S23. The top 10 mutations ranked by SLRF + F3 for PZA. *katG*_S315T for INH, *embB*_M306V, *embB*_M306I, *embB*_Q497R, *embB*_D354A, and *embB*_G406A for EMB, *rpoB*_S450L and *rpoB*_I491F for RI, and *pncA*_H57D and *pncA*_A-11G for PZA are known resistance-associated markers.

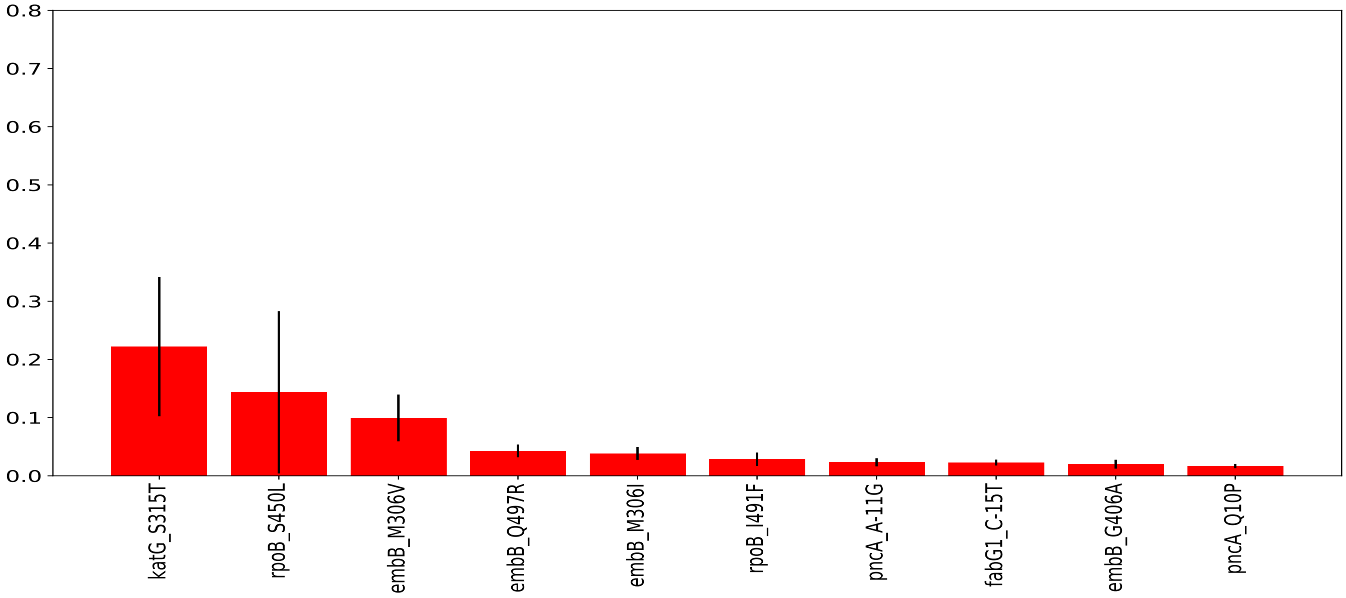

Figure S24. The top 10 mutations ranked by SLRF + F3 for FDR. *katG*_S315T and *fabG1*_C-15T for INH, *embB*_M306V, *embB*_M306I, *embB*_Q497R, and *embB*_G406A for EMB, *rpoB*_S450L and *rpoB*_I491F for RIF, and *pncA*_A-11G and *pncA*_Q10P for PZA are known resistance-associated markers.

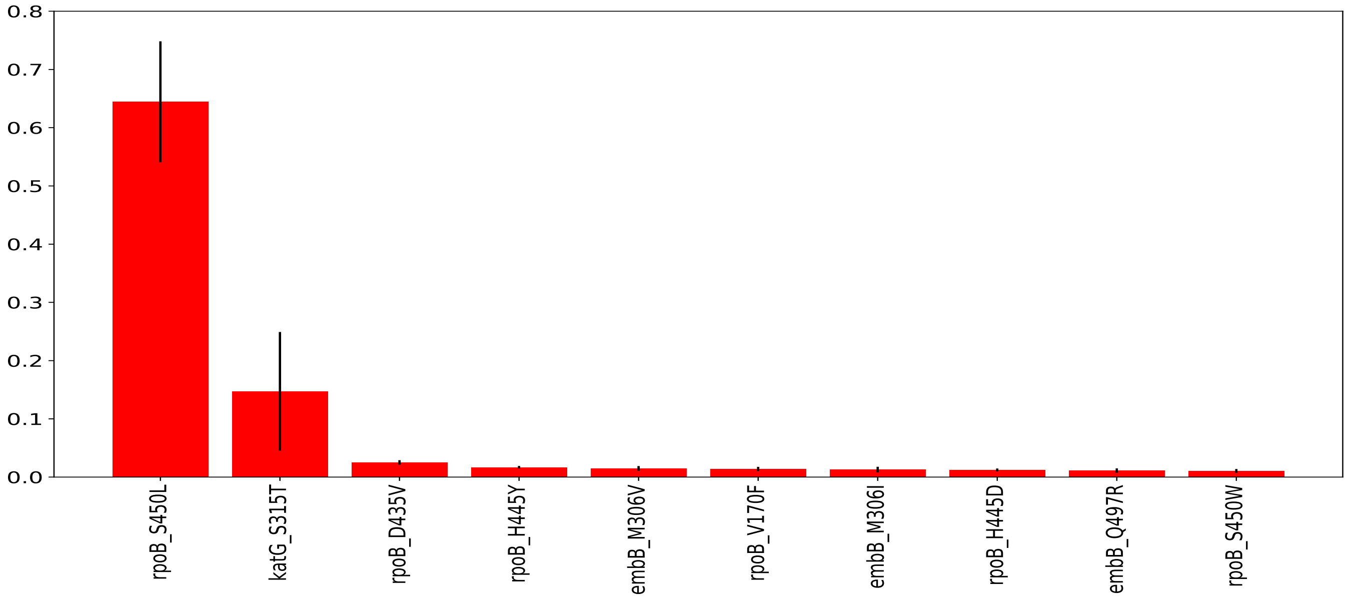

Figure S25. The top 10 mutations ranked by SLRF + F3 for MDR. *katG*_S315T for INH, *embB*_M306V, *embB*_M306I, and *embB*_Q497R for EMB, and *rpoB*_S450L, *rpoB*_D435V, *rpoB*_S450W, *rpoB*_H445Y, *rpoB*_V170F, and *rpoB*_H445D for RIF are known resistance-associated markers.

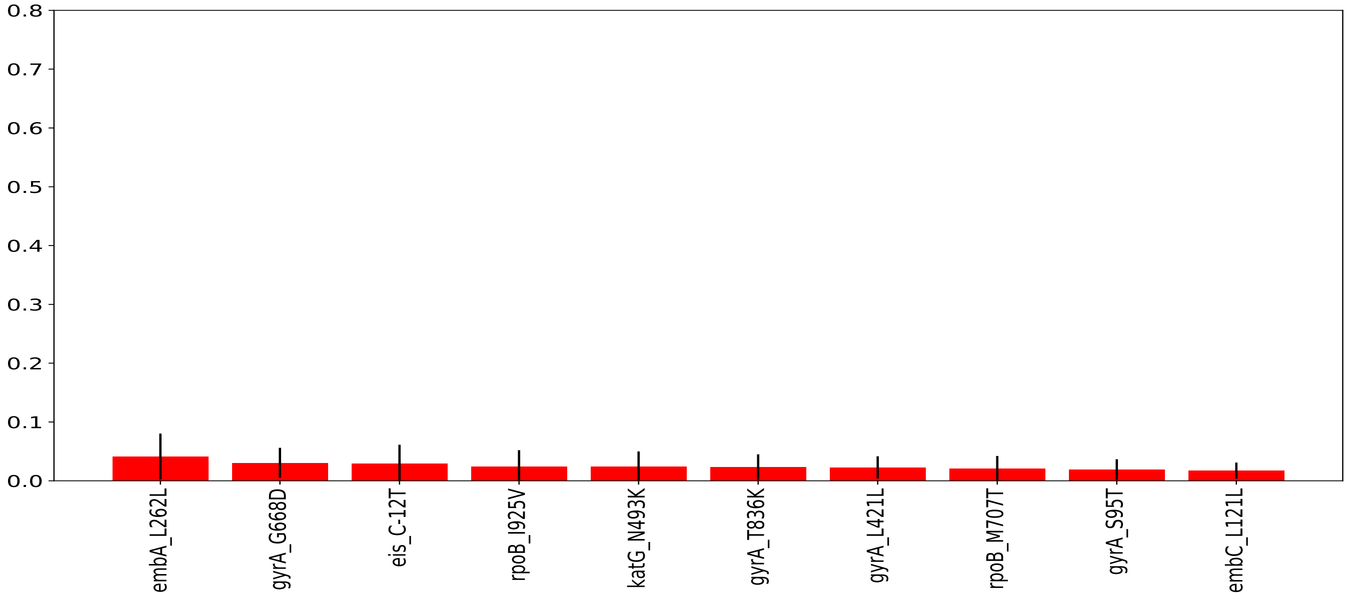

Figure S26. The top 10 mutations ranked by MLRF + F4 for all first-line drugs. *gyrA*_G668D and *gyrA*_S95T are lineage-defining mutations and *katG*_N493K, *embC*_L121L, *rpoB*_M707T, and *embA*_L262L are not in the known catalog that can be resistant/susceptible-associated markers.

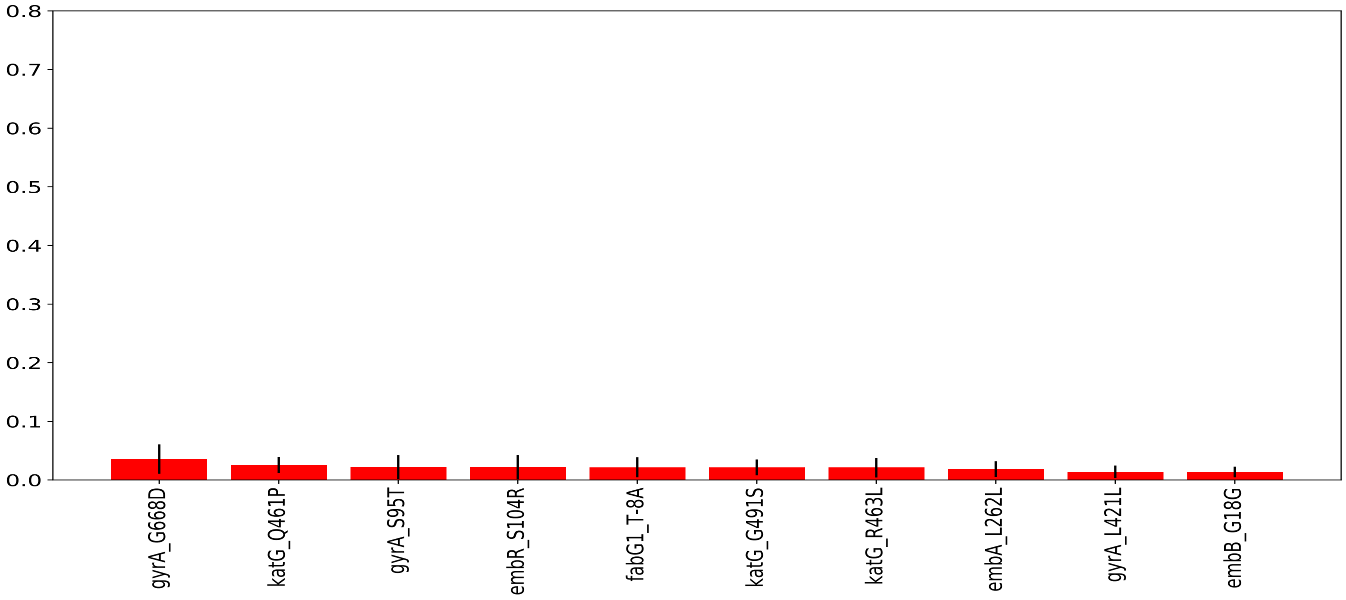

Figure S27. The top 10 mutations ranked by SLRF + F4 for INH. *katG*_R463L, *gyrA*_G668D, and *gyrA*_S95T are lineage-defining mutations and *katG*_G491S, *embR*_S104R, *embA*_L262L, and *embB*_G18G are not in the known catalog and can be resistant/susceptible-associated markers.

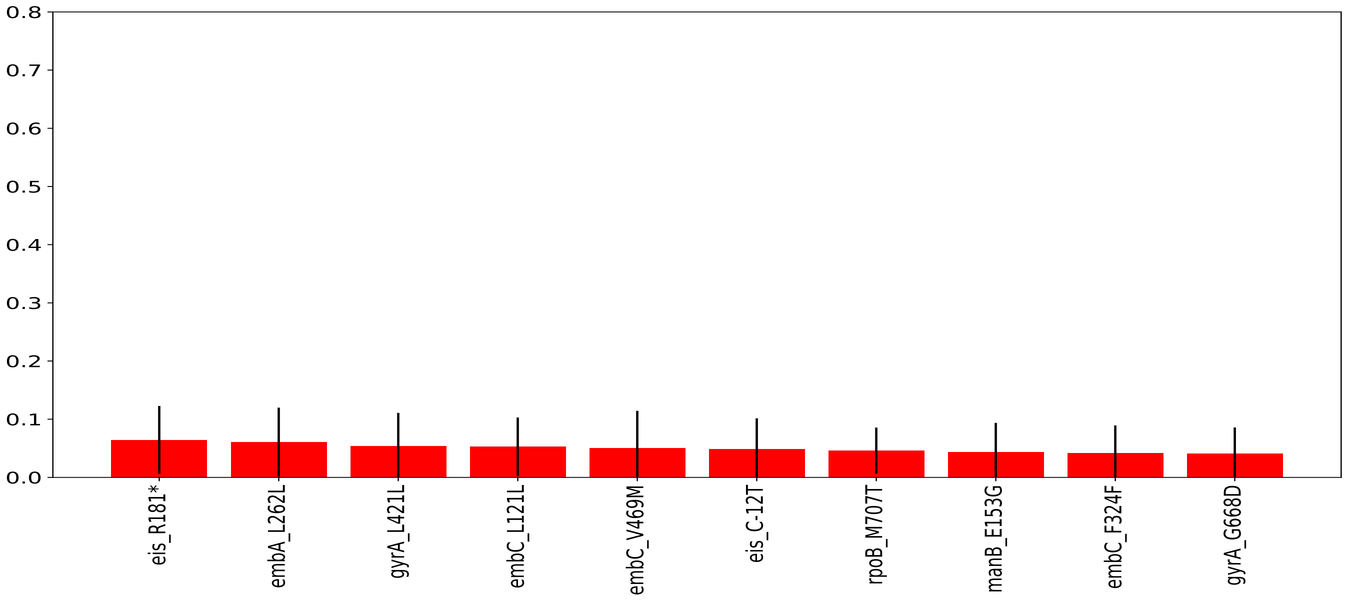

Figure S28. The top 10 mutations ranked by SLRF + F4 for EMB. *embC*_V469M, *embC*_L121L, *manB*_E153G, *embC*_F324F, *rpoB*_M707T, and *embA*_L262L are not in the known catalog and can be resistant/susceptible-associated markers. *gyrA*_G668D is a lineage-defining mutation.

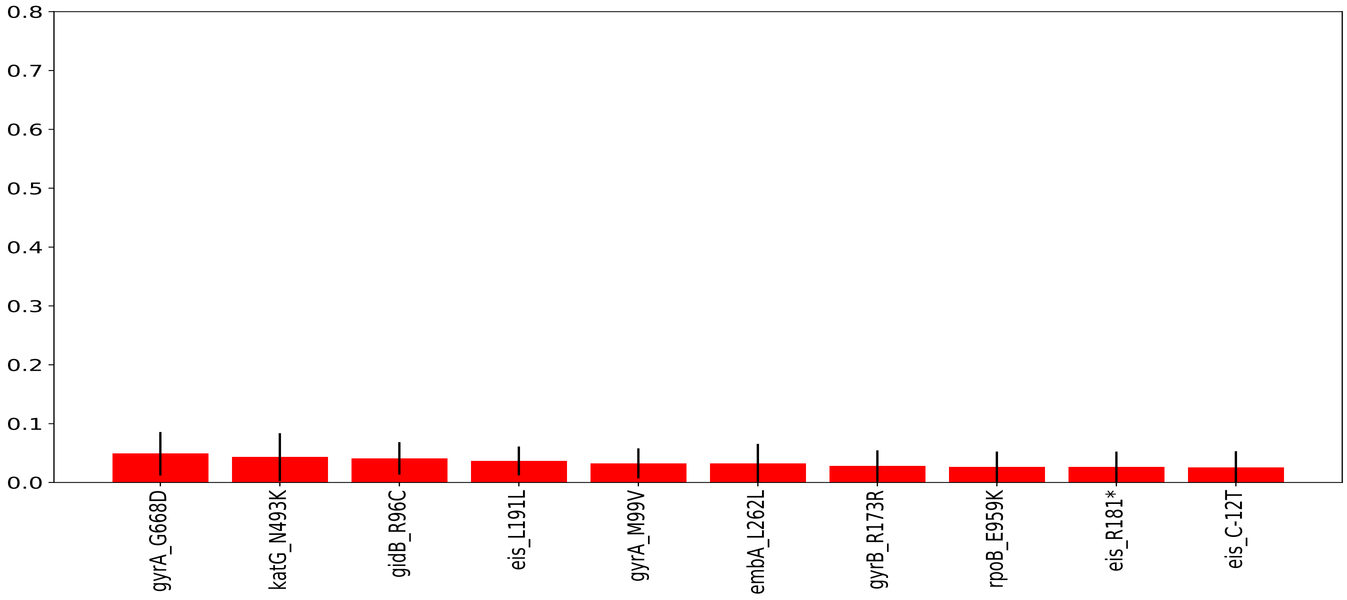

Figure S29. The top 10 mutations ranked by SLRF + F4 for RIF. *gyrA*_G668D is a lineage-defining mutation and *katG*_N493K, *rpoB*_E959K, and *embA*_L262L are not in the known catalog that can be resistant/susceptible-associated markers.

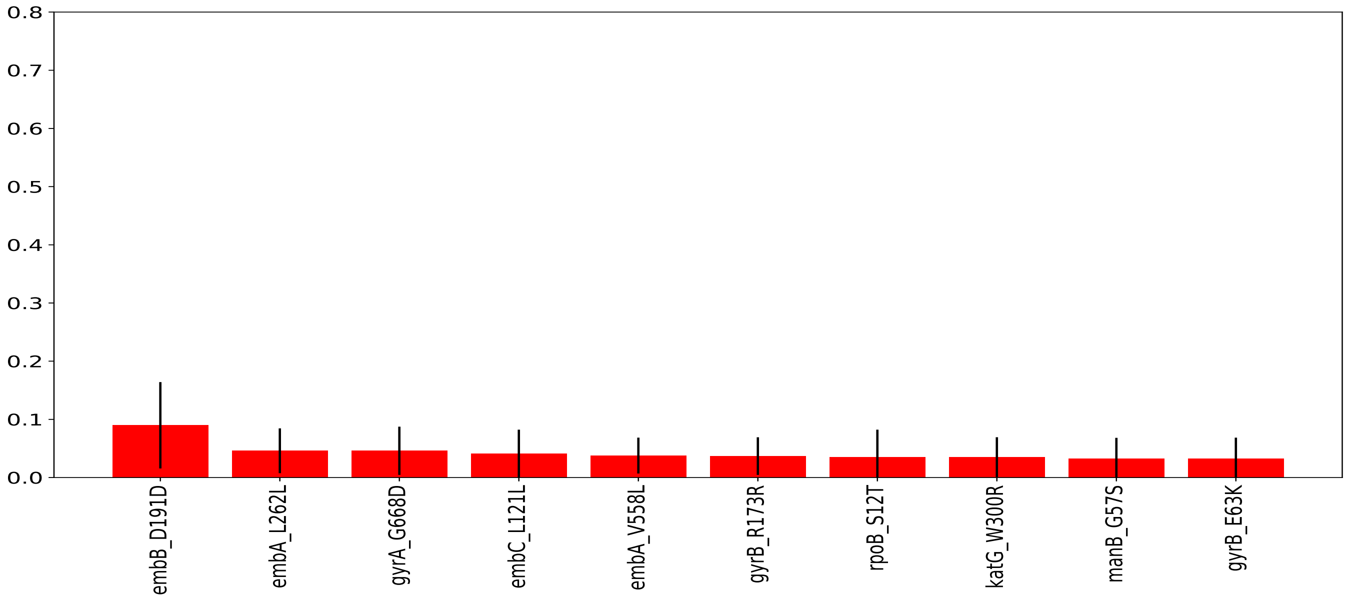

Figure S30. The top 10 mutations ranked by SLRF + F4 for PZA. *embB*_D191D, *embA*_L262L, *embC*_L121L, *embA*_V558L, *katG*_W300R, and *rpoB*_S12T are not in the known catalog that can be resistant/susceptible-associated markers. *manB*_G57S is an EMB susceptible-associated mutation.

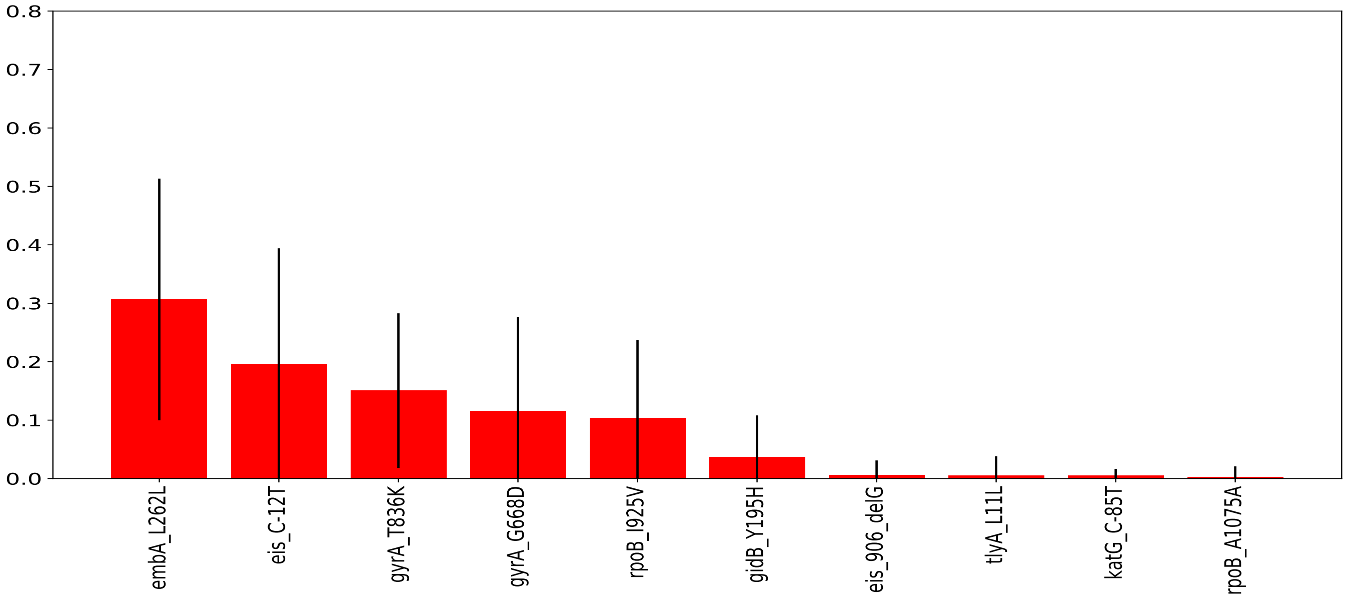

Figure S31. The top 10 mutations ranked by SLRF + F4 for FDR. *gyrA*_G668D is a lineage-defining mutation and *rpoB*_A1075A and *embA*_L262L are not in the known catalog and can be resistant/susceptible-associated markers. *katG*_C-85T is an INH susceptible-associated mutation.

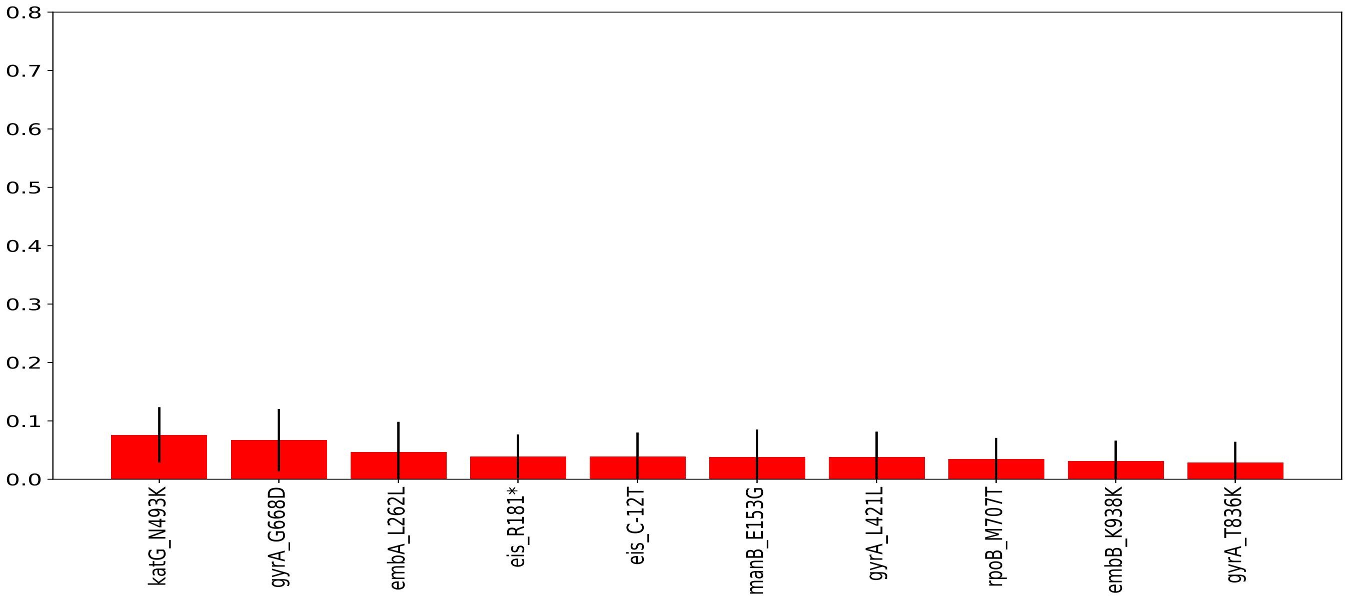

Figure S32. The top 10 mutations ranked by SLRF + F4 for MDR. *gyrA*_G668D is a lineage-defining mutation and *katG*_N493K, *embB*_K938K, *embA*_L262L, *manB*_E153G, and *rpoB*_M707T are not in the known catalog that can be resistant/susceptible-associated markers.

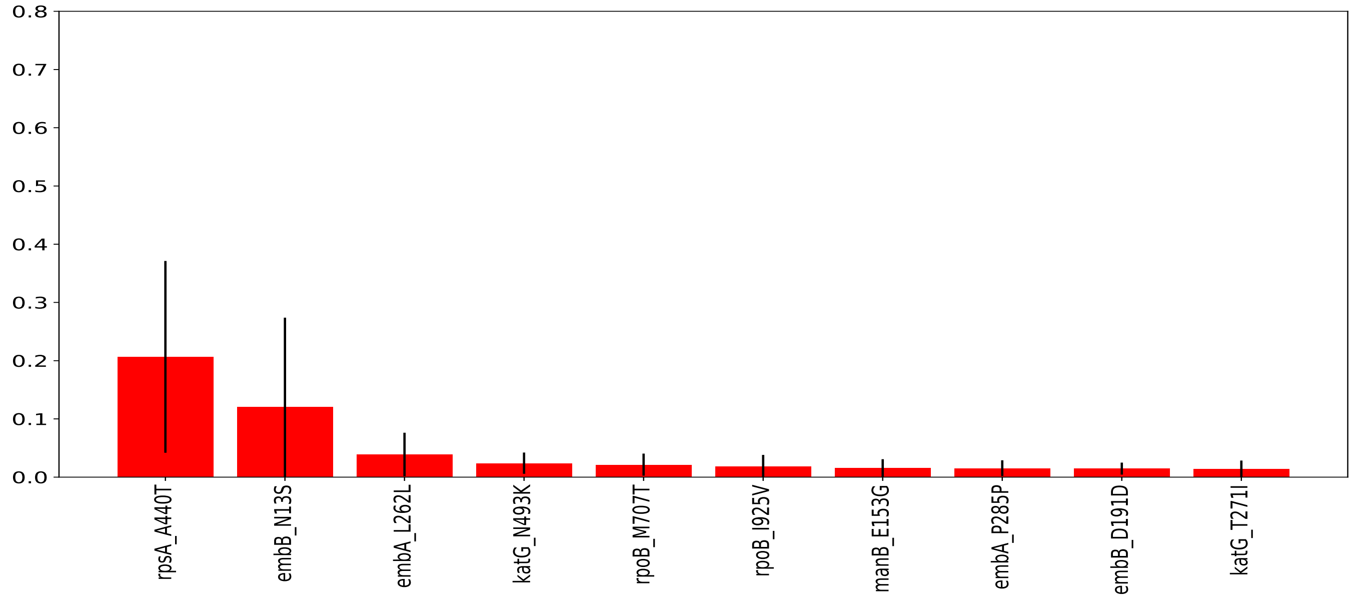

Figure S33. The top 10 mutations ranked by MLRF + F5 for all first-line drugs. *rpsA*_A440T is a lineage-defining mutation and *embB*_N13S is an EMB susceptible-associated mutation. *embA*_L262L, *katG*_N493K, *manB*_E153G, *embB*_K938K, *rpoB*_M707T, *embA*_P285P, and *embB*_D191D are not in the known catalog that can be resistant/susceptible-associated markers.

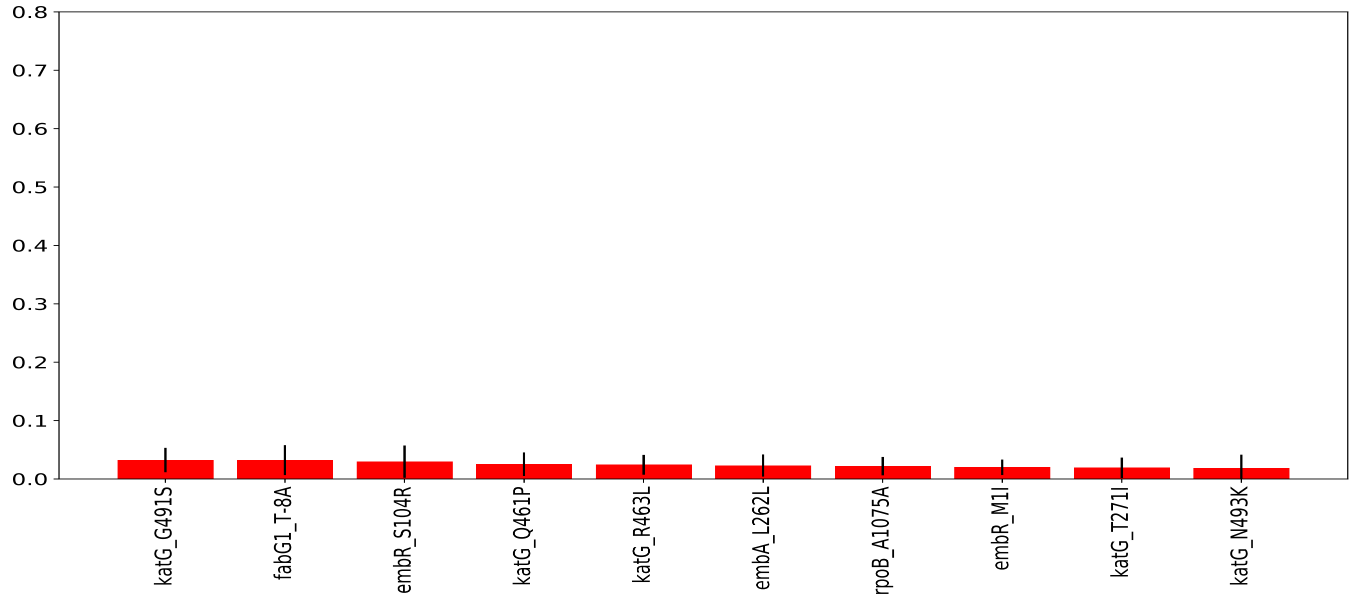

Figure S34. The top 10 mutations ranked by SLRF + F5 for INH. *embR*_S104R, *katG*_G491S, *embA*_L262L, *rpoB*_A1075A, *katG*_N493K and *embR*_M1I are not in the known catalog that can be resistant/susceptible-associated markers. *katG*_R463L is a lineage-defining marker.

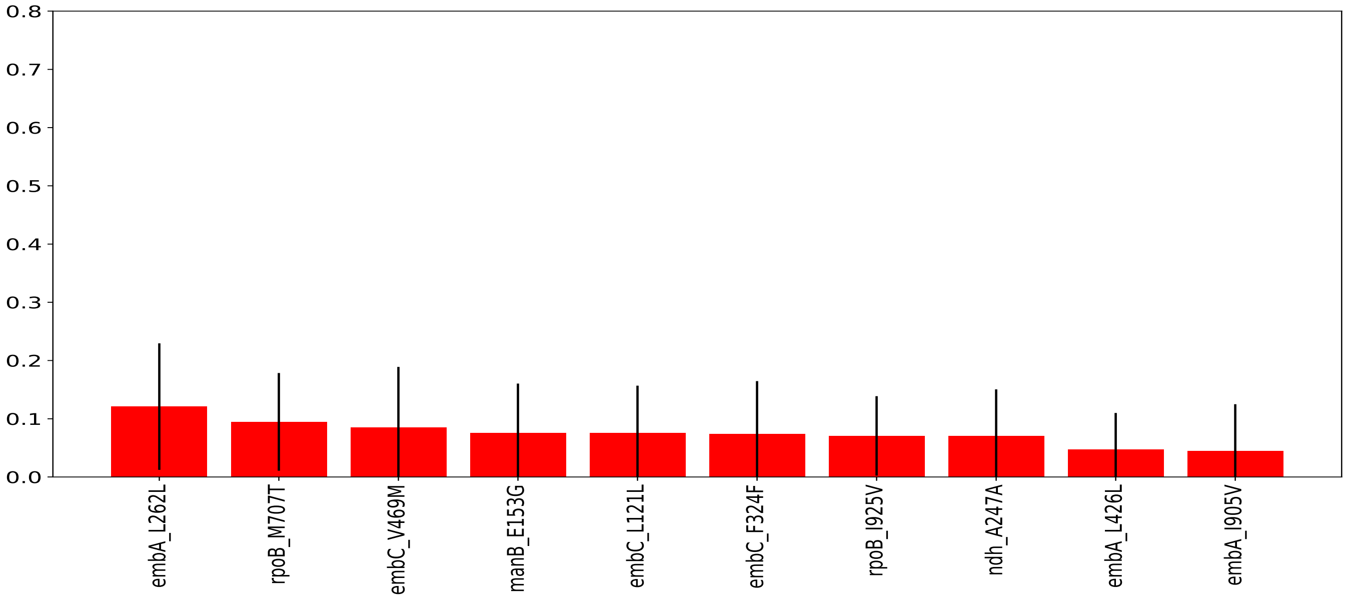

Figure S35. The top 10 mutations ranked by SLRF + F5 for EMB. *embA*_L262L, *embC*_V469M, *ndh*_A247A, *rpoB*_M707T, *embC*_L121L, *manB*_E153G, *embC*_F324F, *embA*_L426L, and *embA*_I905V are not in the known catalog that can be resistant/susceptible-associated markers.

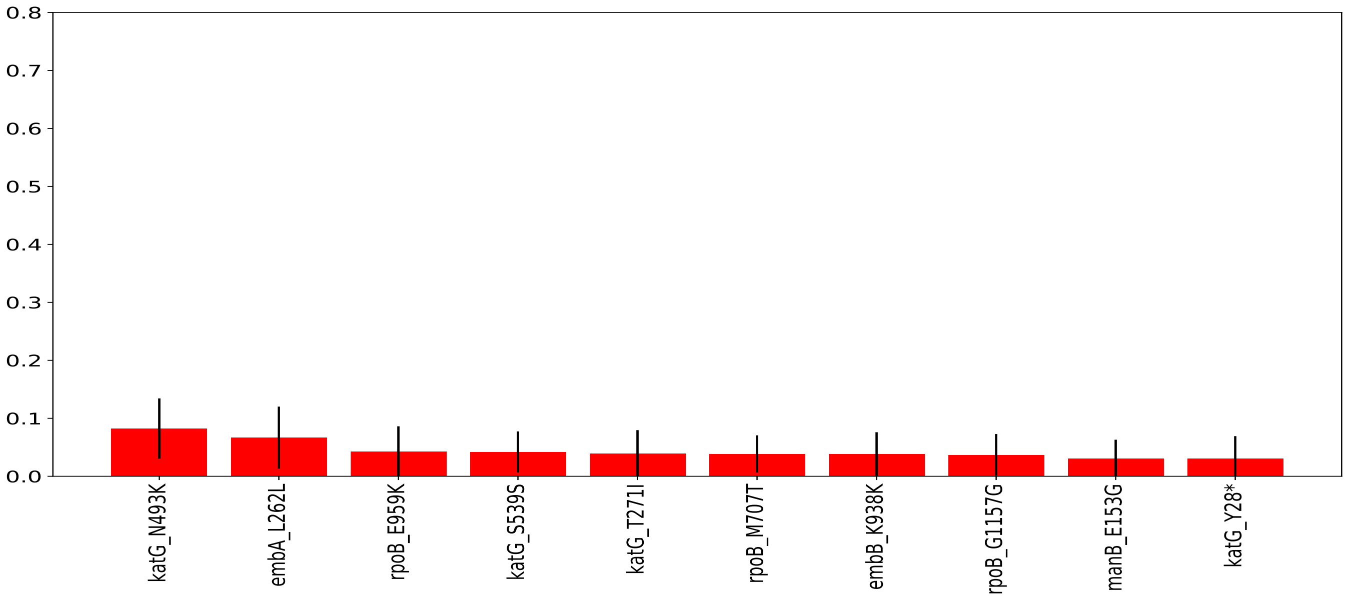

Figure S36. The top 10 mutations ranked by SLRF + F5 for RIF. *katG*_N493K, *embA*_L262L, *katG*_S539S, *rpoB*_E959K, *embB*_K938K, *manB*_E153G, *rpoB*_G1157G, and *rpoB*_M707T are not in the known catalog that can be resistant/susceptible-associated markers.

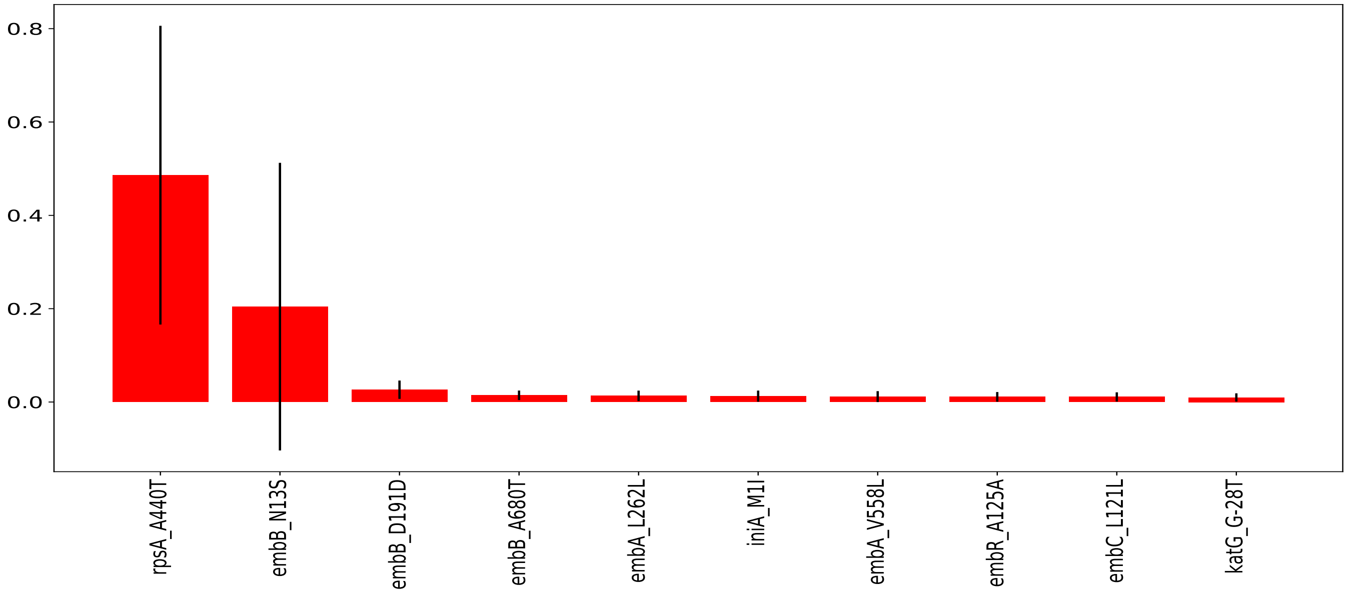

Figure S37. The top 10 mutations ranked by SLRF + F5 for PZA. *rpsA*_A440T is a lineage-defining mutation and *embB*_N13S and *embB*_A680T are EMB susceptible-associated mutations. *embB*_D191D, *embA*_L262L, *iniA*_M1I, *embA*_V558L, *embR*_A125A, *embC*_L121L, and *katG*_G-28T are not in the known catalog that can be resistant/susceptible-associated markers.

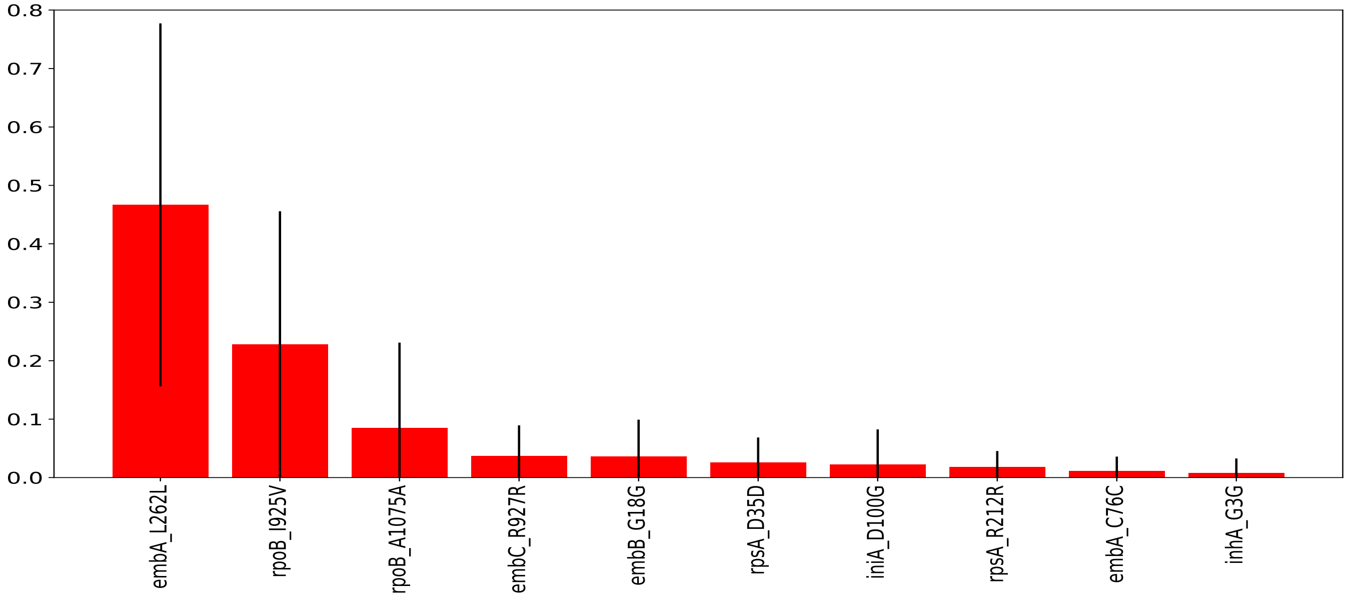

Figure S38. The top 10 mutations ranked by SLRF + F5 for FDR. *iniA*_D100G is an EMB susceptible-associated mutation. *embA*_L262L, *rpoB*_A1075A, *embC*_R927R, *embB*_G18G, *inhA*_G3G, *rpsA*_R212R, *rpsA*_D35D, and *embA*_C76C are not in the known catalog that can be resistant/susceptible-associated markers.

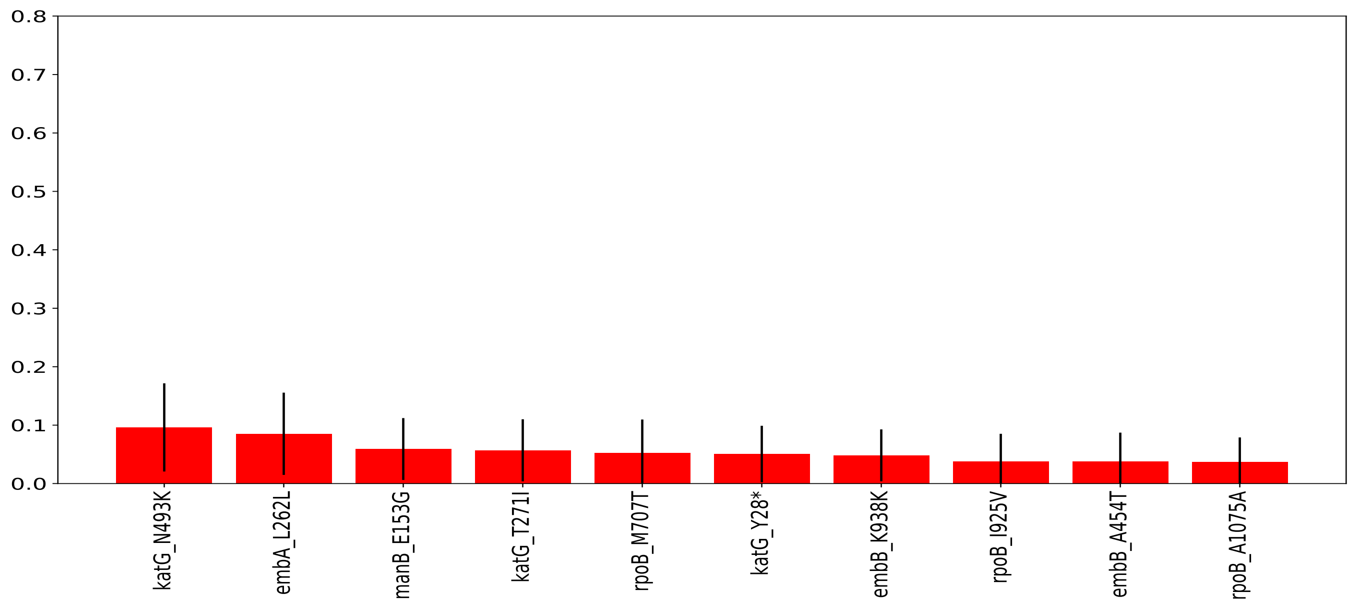

Figure S39. The top 10 mutations ranked by SLRF + F5 for MDR. *katG*_N493K, *embA*_L262L, *rpoB*_M707T, *manB*_E153G, *embB*_K938K, and *rpoB*_A1075A are not in the known catalog that can be resistant/susceptible-associated markers. *embB*_A454T is an EMB susceptible-associated mutation.

SUPPLEMENTARY E

Table S11. The number of mutations with importance of more than 0.05, 0.01, 0.005 and 0.001 ranked by SLRF + F1-F5 feature sets for INH, EMB, RIF, PZA, MDR-TB, and FDR-TB and MLRF + F1-F5 feature sets.

	SLRF						MLRF
	INH	EMB	RIF	PZA	FDR-TB	MDR-TB	All drugs
Threshold = 0.05							
F1	3	2	2	2	3	3	2
F2	3	2	2	2	3	3	2
F3	3	2	2	2	4	3	2
F4	1	12	5	9	5	10	3
F5	4	10	11	2	4	12	3
Threshold = 0.01							
F1	8	8	5	7	10	13	6
F2	6	8	5	8	13	15	10
F3	9	9	5	9	16	16	10
F4	30	27	32	26	7	25	19
F5	36	19	31	9	9	23	17
Threshold = 0.005							
F1	14	15	6	12	17	23	12
F2	20	17	5	11	23	24	10
F3	20	17	6	12	24	31	12
F4	71	37	53	42	9	46	45
F5	63	22	50	23	11	27	41
Threshold = 0.001							
F1	96	59	24	32	143	147	41
F2	93	62	20	37	121	123	43
F3	73	50	16	33	89	83	37
F4	152	50	96	97	23	75	174
F5	110	29	70	57	22	49	120

Table S12. Comparing the performance of MLRF and SLRF for INH, EMB, RIF, PZA, MDR-TB, and FDR-TB based on important mutations indicated by IF1-IF5 and 0.05 as threshold. “I” added before each feature name denotes that this corresponds to the top important features for the equivalent feature space.

			INH			EMB	
Feature set	Method	Sensitivity	Specificity	AUC	Sensitivity	Specificity	AUC
IF1	MLRF	79.89 ± 1.03	99.08 ± 0.14	89.49 ± 0.55	90.80 ± 1.59	88.02 ± 0.29	89.41 ± 0.87
	SLRF	84.31 ± 1.33	99.19 ± 0.14	91.74 ± 0.64	92.02 ± 1.32	87.98 ± 0.34	**89.99± 0.67**
IF2	MLRF	79.89 ± 1.43	99.08 ± 0.13	89.49 ± 0.73	90.80 ± 1.18	88.01 ± 0.41	89.41 ± 0.66
	SLRF	84.31 ± 1.32	99.19 ± 0.20	91.75 ± 0.70	92.02 ± 1.50	87.98 ± 0.29	89.99 ± 0.76
IF3	MLRF	79.89 ± 1.33	99.08 ± 0.21	89.48 ± 0.66	90.80 ± 1.74	88.02 ± 0.38	89.41 ± 0.89
	SLRF	84.31 ± 1.25	99.19 ± 0.16	**91.75 ± 0.62**	92.02 ± 1.59	87.97 ± 0.44	89.99 ± 0.79
IF4	MLRF	0.96 ± 1.40	99.99 ± 0.03	50.47 ± 0.70	3.14 ± 4.63	99.99 ± 0.02	51.57 ± 2.32
	SLRF	0.00 ± 0.00	100.00 ± 0.00	50.00 ± 0.00	3.66 ± 4.91	99.97 ± 0.04	51.82 ± 2.46
IF5	MLRF	0.76 ± 1.29	99.99 ± 0.03	50.37 ± 0.64	2.60 ± 4.41	99.99 ± 0.03	51.29 ± 2.20
	SLRF	3.84 ± 2.31	99.96 ± 0.04	51.90 ± 1.15	2.16 ± 4.34	99.95 ± 0.046	51.06 ± 2.17
			RIF			PZA	
Feature set	Method	Sensitivity	Specificity	AUC	Sensitivity	Specificity	AUC
IF1	MLRF	86.96 ± 1.07	94.66 ± 0.28	90.81 ± 0.61	79.80 ± 1.49	86.26 ± 0.29	83.03 ± 0.82
	SLRF	86.96 ± 1.38	94.66 ± 0.33	90.81 ± 0.71	84.81 ± 1.86	87.35 ± 0.43	86.08± 0.96
IF2	MLRF	89.64 ± 1.51	93.26 ± 0.52	91.45 ± 0.73	85.05 ± 2.21	88.42 ± 0.60	86.73 ± 1.01
	SLRF	90.15 ± 1.06	92.97 ± 0.45	**91.56 ± 0.49**	85.33 ± 2.24	89.01 ± 0.79	**87.17 ± 1.14**
IF3	MLRF	86.96 ± 1.04	94.66 ± 0.36	90.81 ± 0.52	79.80 ± 1.47	86.26 ± 0.37	83.03 ± 0.72
	SLRF	86.96 ± 1.36	94.66 ± 0.35	90.81 ± 0.66	86.95 ± 2.27	86.26 ± 0.62	86.61 ± 1.02
IF4	MLRF	1.54 ± 2.25	99.99 ± 0.02	50.76 ± 1.12	2.07 ± 3.03	99.99 ± 0.02	51.03 ± 1.52
	SLRF	4.03 ± 3.36	99.98 ± 0.04	52.00 ± 1.69	9.12 ± 5.81	99.91 ± 0.07	54.51 ± 2.91
IF5	MLRF	1.27 ± 2.15	99.99 ± 0.03	50.63 ± 1.07	47.12 ± 6.32	99.92 ± 0.06	73.52 ± 3.15
	SLRF	2.07 ± 2.67	99.98 ± 0.03	51.02 ± 1.33	46.62 ± 5.18	99.94 ± 0.06	73.28 ± 2.59
			FDR-TB			MDR-TB	
Feature set	Method	Sensitivity	Specificity	AUC	Sensitivity	Specificity	AUC
IF1	MLRF	93.85 ± 1.80	84.21 ± 0.28	89.03 ± 0.97	90.78 ± 1.05	94.27 ± 0.26	92.52 ± 0.58
	SLRF	92.79 ± 5.10	84.83 ± 1.80	88.81 ± 1.73	90.78 ± 1.28	94.27 ± 0.34	92.52 ± 0.67
IF2	MLRF	93.85 ± 1.39	84.21 ± 0.39	**89.11 ± 0.76**	90.78 ± 1.11	94.27 ± 0.36	92.52 ± 0.58
	SLRF	93.31 ± 4.59	84.65 ± 1.59	88.98 ± 1.59	90.78 ± 1.26	94.27 ± 0.28	92.52 ± 0.63
IF3	MLRF	93.87 ± 1.86	84.21 ± 0.38	89.03 ± 0.93	90.78 ± 1.07	94.27 ± 0.36	**92.52 ± 0.53**
	SLRF	93.59 ± 2.80	84.62 ± 1.26	89.11 ± 0.97	90.78 ± 1.34	94.27 ± 0.37	92.52 ± 0.65
IF4	MLRF	8.67 ± 12.80	99.99 ± 0.02	54.33 ± 6.40	2.51 ± 3.66	99.99 ± 0.02	51.25 ± 1.83
	SLRF	10.83 ± 14.65	99.98 ± 0.03	55.41 ± 7.33	5.76 ± 5.78	99.98 ± 0.03	52.87 ± 2.89
IF5	MLRF	8.00 ± 14.24	99.99 ± 0.03	53.99 ± 7.12	2.05 ± 3.47	99.99 ± 0.03	51.02 ± 1.73
	SLRF	5.50 ± 11.13	99.98 ± 0.03	52.74 ± 5.57	3.40 ± 4.42	99.97 ± 0.03	51.67 ± 2.21

Table S13. Comparing the performance of MLRF and SLRF for INH, EMB, RIF, PZA, MDR-TB, and FDR-TB based on important mutations indicated by IF1-IF5 and 0.01 as threshold. "I" added before each feature name denotes that this corresponds to the top important features for the equivalent feature space.

			INH			EMB	
Feature set	Method	Sensitivity	Specificity	AUC	Sensitivity	Specificity	AUC
IF1	MLRF	88.49 ± 1.26	98.44 ± 0.24	93.46 ± 0.63	91.58 ± 3.51	88.28 ± 1.78	89.93 ± 1.02
	SLRF	90.13 ± 1.05	98.68 ± 0.27	94.40 ± 0.54	90.66 ± 3.69	90.37 ± 2.45	90.51 ± 0.84
IF2	MLRF	89.09 ± 0.97	98.22 ± 0.26	93.65 ± 0.52	89.98 ± 3.76	90.63 ± 2.71	90.30 ± 0.79
	SLRF	90.13 ± 1.01	98.68 ± 0.23	94.40 ± 0.53	90.96 ± 3.61	89.79 ± 2.49	90.37 ± 0.78
IF3	MLRF	89.08 ± 1.07	98.21 ± 0.24	93.64 ± 0.52	89.58 ± 3.64	91.19 ± 2.49	90.39 ± 0.95
	SLRF	90.13 ± 0.98	98.68 ± 0.22	**94.41 ± 0.52**	89.32 ± 2.74	92.06 ± 2.10	**90.69 ± 0.89**
IF4	MLRF	4.01 ± 3.05	99.83 ± 0.14	51.92 ± 1.53	3.69 ± 4.74	99.84 ± 0.11	51.77 ± 2.37
	SLRF	9.22 ± 3.85	99.63 ± 0.16	54.42 ± 1.92	4.04 ± 5.00	99.64 ± 0.18	51.84 ± 2.49
IF5	MLRF	3.31 ± 2.55	99.86 ± 0.09	51.58 ± 1.29	1.89 ± 4.06	99.82 ± 0.14	50.85 ± 2.03
	SLRF	8.92 ± 4.49	99.62 ± 0.16	54.27 ± 2.24	2.12 ± 4.02	99.73 ± 0.22	50.93 ± 2.02
			RIF			PZA	
Feature set	Method	Sensitivity	Specificity	AUC	Sensitivity	Specificity	AUC
IF1	MLRF	90.79 ± 1.41	92.64 ± 0.95	91.72 ± 0.65	81.20 ± 1.48	85.98 ± 0.50	83.59 ± 0.73
	SLRF	92.10 ± 0.90	94.62 ± 0.30	93.36 ± 0.47	88.57 ± 1.80	86.96 ± 0.46	**87.77 ± 0.91**
IF2	MLRF	91.13 ± 1.21	92.90 ± 1.11	92.02 ± 0.54	81.51 ± 1.42	85.93 ± 0.48	83.72 ± 0.72
	SLRF	92.44 ± 1.11	94.56 ± 0.39	93.50 ± 0.59	87.69 ± 2.15	86.00 ± 0.65	86.85 ± 1.03
IF3	MLRF	91.11 ± 1.52	92.86 ± 1.09	91.98 ± 0.60	88.47 ± 1.88	85.99 ± 0.58	87.23 ± 0.93
	SLRF	93.12 ± 0.96	94.20 ± 0.36	**93.66 ± 0.53**	85.15 ± 2.75	89.11 ± 2.22	87.13 ± 0.87
IF4	MLRF	5.53 ± 4.16	99.82 ± 0.12	52.67 ± 2.10	10.42 ± 7.23	99.88 ± 0.10	55.15 ± 3.62
	SLRF	6.57 ± 5.32	99.83 ± 0.11	53.20 ± 2.66	12.22 ± 7.82	99.81 ± 0.11	56.01 ± 3.92
IF5	MLRF	3.06 ± 3.27	99.82 ± 0.12	51.44 ± 1.64	50.46 ± 6.02	99.76 ± 0.11	75.11 ± 3.02
	SLRF	2.04 ± 2.60	99.78 ± 0.14	50.91 ± 1.30	52.84 ± 6.69	99.84 ± 0.08	76.34 ± 3.34
			FDR-TB			MDR-TB	
Feature set	Method	Sensitivity	Specificity	AUC	Sensitivity	Specificity	AUC
IF1	MLRF	86.81 ± 2.76	90.29 ± 0.46	88.55 ± 1.37	92.92 ± 1.44	93.36 ± 1.13	93.14 ± 0.63
	SLRF	88.09 ± 3.35	90.93 ± 0.87	89.51 ± 1.42	93.66 ± 1.06	93.86 ± 0.35	93.76 ± 0.56
IF2	MLRF	89.61 ± 2.06	90.05 ± 0.54	89.83 ± 0.97	93.13 ± 1.08	93.57 ± 0.73	93.35 ± 0.53
	SLRF	89.05 ± 3.14	91.37 ± 0.83	90.21 ± 1.27	95.14 ± 1.21	93.41 ± 0.40	94.28 ± 0.60
IF3	MLRF	89.81 ± 2.67	90.03 ± 0.46	89.92 ± 1.28	93.08 ± 1.32	93.47 ± 0.93	93.27 ± 0.61
	SLRF	91.74 ± 3.37	90.06 ± 0.61	**90.90 ± 1.56**	95.22 ± 0.89	93.39 ± 0.31	**94.31 ± 0.46**
IF4	MLRF	10.50 ± 13.62	99.95 ± 0.05	55.23 ± 6.81	7.72 ± 6.61	99.90 ± 0.07	53.81 ± 3.32
	SLRF	9.67 ± 13.68	99.97 ± 0.03	54.82 ± 6.84	5.98 ± 5.40	99.85 ± 0.10	52.91 ± 2.70
IF5	MLRF	5.00 ± 11.18	99.92 ± 0.07	52.46 ± 5.59	4.96 ± 5.37	99.88 ± 0.09	52.42 ± 2.69
	SLRF	5.67 ± 11.48	99.94 ± 0.07	52.80 ± 5.74	3.60 ± 4.70	99.76 ± 0.16	51.68 ± 2.35

Table S14. Comparing the performance of MLRF and SLRF for INH, EMB, RIF, PZA, MDR-TB, and FDR-TB based on important mutations indicated by IF1-IF5 and 0.005 as threshold. “I” added before each feature name denotes that this corresponds to the top important features for the equivalent feature space.

			INH			EMB	
Feature set	Method	Sensitivity	Specificity	AUC	Sensitivity	Specificity	AUC
IF1	MLRF	91.88 ± 1.31	97.21 ± 0.41	94.55 ± 0.64	90.98 ± 1.59	91.20 ± 0.50	91.09 ± 0.73
	SLRF	90.57 ± 1.19	98.67 ± 0.19	94.62 ± 0.58	90.97 ± 2.01	91.78 ± 0.66	91.37 ± 0.95
IF2	MLRF	91.93 ± 0.98	98.04 ± 0.33	94.99 ± 0.50	89.55 ± 2.93	90.90 ± 2.06	90.23 ± 0.87
	SLRF	90.13 ± 1.26	98.68 ± 0.22	94.40 ± 0.64	91.12 ± 1.50	92.02 ± 0.92	91.57 ± 0.74
IF3	MLRF	91.98 ± 1.08	98.06 ± 0.27	**95.02 ± 0.50**	89.58 ± 2.44	91.45 ± 1.75	90.51 ± 0.79
	SLRF	90.57 ± 1.20	98.67 ± 0.26	94.62 ± 0.61	91.10 ± 1.76	92.70 ± 0.51	**91.90 ± 0.82**
IF4	MLRF	11.34 ± 4.74	99.43 ± 0.24	55.38 ± 2.36	3.52 ± 4.95	99.82 ± 0.14	51.67 ± 2.48
	SLRF	10.67 ± 4.58	99.40 ± 0.24	55.03 ± 2.27	4.43 ± 5.66	99.66 ± 0.18	52.04 ± 2.82
IF5	MLRF	8.84 ± 5.69	99.62 ± 0.12	54.23 ± 2.83	2.27 ± 4.31	99.85 ± 0.13	51.06 ± 2.15
	SLRF	9.51 ± 4.10	99.56 ± 0.16	54.53 ± 2.04	3.16 ± 4.65	99.70 ± 0.24	51.43 ± 2.35
			RIF			PZA	
Feature set	Method	Sensitivity	Specificity	AUC	Sensitivity	Specificity	AUC
IF1	MLRF	92.33 ± 1.04	94.20 ± 0.47	93.26 ± 0.51	84.84 ± 2.23	89.40 ± 0.91	87.12 ± 1.00
	SLRF	93.14 ± 1.25	94.20 ± 0.39	93.67 ± 0.62	85.45 ± 1.90	89.57 ± 1.91	87.51 ± 0.85
IF2	MLRF	92.85 ± 1.18	94.23 ± 0.33	93.54 ± 0.59	85.86 ± 2.30	87.13 ± 0.97	86.50 ± 0.95
	SLRF	93.14 ± 1.03	94.19 ± 0.39	93.66 ± 0.52	84.91 ± 2.00	89.39 ± 0.80	87.15 ± 0.92
IF3	MLRF	92.93 ± 0.86	94.23 ± 0.34	**93.68 ± 0.46**	86.06 ± 2.57	87.27 ± 1.83	86.67 ± 0.89
	SLRF	93.06 ± 1.19	94.24 ± 0.38	93.65 ± 0.64	84.72 ± 1.64	90.69 ± 0.88	**87.71 ± 0.74**
IF4	MLRF	8.98 ± 4.87	99.65 ± 0.20	54.32 ± 2.42	11.90 ± 7.67	99.78 ± 0.15	55.84 ± 3.84
	SLRF	8.83 ± 6.08	99.29 ± 0.32	54.06 ± 3.01	11.26 ± 7.69	99.68 ± 0.14	55.47 ± 3.86
IF5	MLRF	2.41 ± 2.94	99.80 ± 0.11	51.10 ± 1.45	51.72 ± 4.33	99.70 ± 0.18	75.71 ± 2.19
	SLRF	3.66 ± 3.90	99.33 ± 0.29	51.49 ± 1.93	54.09 ± 6.79	99.79 ± 0.12	76.94 ± 3.38
			FDR-TB			MDR-TB	
Feature set	Method	Sensitivity	Specificity	AUC	Sensitivity	Specificity	AUC
IF1	MLRF	90.20 ± 3.36	90.33 ± 0.98	90.26 ± 1.39	94.19 ± 1.01	93.69 ± 0.47	93.94 ± 0.44
	SLRF	86.06 ± 3.57	92.81 ± 1.21	89.44 ± 1.48	95.18 ± 1.27	93.38 ± 0.43	94.28 ± 0.61
IF2	MLRF	88.25 ± 2.61	91.33 ± 0.76	89.79 ± 1.19	94.82 ± 1.15	93.72 ± 0.64	94.27 ± 0.59
	SLRF	87.95 ± 2.70	92.69 ± 0.58	90.32 ± 1.26	95.23 ± 0.90	93.38 ± 0.41	94.30 ± 0.46
IF3	MLRF	89.41 ± 3.48	90.37 ± 0.97	89.89 ± 1.40	94.97 ± 0.90	93.69 ± 0.36	**94.37 ± 0.47**
	SLRF	89.28 ± 3.47	91.50 ± 1.11	**90.39 ± 1.43**	95.01 ± 1.09	93.73 ± 0.37	94.37 ± 0.57
IF4	MLRF	9.83 ± 13.91	99.96 ± 0.04	54.90 ± 6.96	9.23 ± 7.14	99.79 ± 0.15	54.51 ± 3.57
	SLRF	9.83 ± 13.30	99.96 ± 0.04	54.90 ± 6.65	8.11 ± 6.58	99.65 ± 0.18	53.88 ± 3.27
IF5	MLRF	6.50 ± 12.72	99.93 ± 0.07	53.22 ± 6.35	3.90 ± 4.78	99.85 ± 0.10	51.88 ± 2.38
	SLRF	8.00 ± 13.33	99.92 ± 0.07	53.96 ± 6.67	5.16 ± 5.80	99.75 ± 0.17	52.45 ± 2.90

Table S15. Comparing the performance of MLRF and SLRF for INH, EMB, RIF, PZA, MDR-TB, and FDR-TB based on important mutations indicated by IF1-IF5 and 0.001 as threshold. "I" added before each feature name denotes that this corresponds to the top important features for the equivalent feature space.

		INH			EMB		
Feature set	Method	Sensitivity	Specificity	AUC	Sensitivity	Specificity	AUC
IF1	MLRF	92.87 ± 1.06	97.56 ± 0.34	95.22 ± 0.54	89.11 ± 2.29	92.59 ± 0.44	90.85 ± 1.10
	SLRF	93.07 ± 1.04	97.59 ± 0.44	95.33 ± 0.46	90.53 ± 1.66	92.90 ± 0.55	**91.71 ± 0.80**
IF2	MLRF	92.96 ± 0.83	97.64 ± 0.52	95.30 ± 0.48	89.24 ± 1.80	92.34 ± 0.55	90.79 ± 0.87
	SLRF	92.78 ± 1.21	98.02 ± 0.30	95.40 ± 0.59	90.51 ± 1.88	92.49 ± 0.47	91.50 ± 0.90
IF3	MLRF	92.88 ± 0.93	97.88 ± 0.31	**95.48 ± 0.40**	89.34 ± 2.02	92.38 ± 0.54	90.86 ± 0.94
	SLRF	92.80 ± 0.85	98.16 ± 0.30	95.48 ± 0.45	90.26 ± 1.86	92.78 ± 0.36	91.52 ± 0.89
IF4	MLRF	12.51 ± 4.88	98.68 ± 0.43	55.60 ± 2.38	4.14 ± 5.29	99.57 ± 0.29	51.85 ± 2.67
	SLRF	12.50 ± 4.90	98.68 ± 0.42	55.59 ± 2.42	3.88 ± 5.32	99.58 ± 0.23	51.73 ± 2.65
IF5	MLRF	10.56 ± 4.31	99.24 ± 0.26	54.90 ± 2.15	3.22 ± 5.13	99.67 ± 0.20	51.45 ± 2.53
	SLRF	3.48 ± 4.88	99.77 ± 0.18	51.62 ± 2.43	10.46 ± 3.89	98.78 ± 0.33	54.62 ± 1.94
		RIF			PZA		
Feature set	Method	Sensitivity	Specificity	AUC	Sensitivity	Specificity	AUC
IF1	MLRF	92.19 ± 1.10	97.77 ± 0.52	**94.98 ± 0.53**	82.36 ± 2.16	91.77 ± 0.61	87.06 ± 1.10
	SLRF	92.29 ± 1.36	96.61 ± 0.54	94.45 ± 0.61	84.73 ± 2.49	92.83 ± 0.52	**88.78 ± 1.17**
IF2	MLRF	92.38 ± 1.10	96.35 ± 0.48	94.37 ± 0.56	83.32 ± 2.62	89.86 ± 0.60	86.59 ± 1.28
	SLRF	92.47 ± 1.19	96.45 ± 0.69	94.46 ± 0.60	84.31 ± 2.13	90.40 ± 0.77	87.35 ± 0.98
IF3	MLRF	91.31 ± 1.48	97.42 ± 1.56	94.37 ± 0.67	83.00 ± 2.33	90.74 ± 0.58	86.87 ± 1.05
	SLRF	91.67 ± 1.12	98.19 ± 0.63	94.93 ± 0.54	83.92 ± 2.24	91.31 ± 0.36	87.61 ± 1.11
IF4	MLRF	8.42 ± 5.63	99.09 ± 0.35	53.75 ± 2.85	11.16 ± 6.29	99.47 ± 0.28	55.31 ± 3.12
	SLRF	8.13 ± 4.96	98.99 ± 0.37	53.56 ± 2.52	11.22 ± 6.74	99.32 ± 0.30	55.27 ± 3.35
IF5	MLRF	2.61 ± 2.94	99.58 ± 0.22	51.09 ± 1.47	53.35 ± 5.76	99.49 ± 0.20	76.42 ± 2.86
	SLRF	2.99 ± 2.87	99.18 ± 0.37	51.08 ± 1.47	53.67 ± 6.54	99.49 ± 0.17	76.58 ± 3.27
		FDR-TB			MDR-TB		
Feature set	Method	Sensitivity	Specificity	AUC	Sensitivity	Specificity	AUC
IF1	MLRF	85.16 ± 2.82	93.19 ± 0.42	89.18 ± 1.37	93.76 ± 1.33	97.38 ± 0.49	**95.47 ± 0.62**
	SLRF	84.60 ± 3.95	94.03 ± 0.50	89.31 ± 1.93	93.43 ± 1.26	97.40 ± 0.46	95.41 ± 0.60
IF2	MLRF	85.51 ± 2.81	92.97 ± 0.50	89.24 ± 1.33	93.90 ± 1.09	96.00 ± 0.39	94.95 ± 0.53
	SLRF	84.96 ± 2.81	93.61 ± 0.45	89.29 ± 1.38	93.76 ± 1.38	95.93 ± 0.67	94.85 ± 0.66
IF3	MLRF	86.85 ± 2.72	92.34 ± 0.70	**89.60 ± 1.26**	92.82 ± 1.63	96.93 ± 1.51	94.88 ± 0.62
	SLRF	85.76 ± 3.59	92.58 ± 0.98	89.17 ± 1.47	93.08 ± 1.24	97.67 ± 0.64	95.37 ± 0.61
IF4	MLRF	11.17 ± 14.49	99.95 ± 0.06	55.56 ± 7.25	8.06 ± 7.29	99.59 ± 0.22	53.82 ± 3.66
	SLRF	9.17 ± 13.57	99.95 ± 0.04	54.56 ± 6.78	8.97 ± 7.65	99.62 ± 0.20	54.30 ± 3.81
IF5	MLRF	7.67 ± 13.42	99.93 ± 0.07	53.80 ± 6.71	4.27 ± 4.82	99.71 ± 0.19	51.99 ± 2.41
	SLRF	8.50 ± 14.09	99.95 ± 0.06	54.22 ± 7.05	5.16 ± 4.80	99.67 ± 0.22	52.42 ± 2.40

SUPPLEMENTARY F

Considering top-ranked mutations that were not in the library or known as lineage-defining, we calculated the number of their appearance in each isolate and lineage. We also included the number of isolates having a known resistant-associated mutation in addition to the given mutation. Such analysis can further filter out mutations that are lineage-associated and have a refined list of possible new mutations. Only mutations appeared in more than a lineage is shown in Table S16.

Table S16. List of unknown mutations, their number of appearance in isolates, their appearance in resistant isolates for each drug (INH, EMB, RIF and PZA respectively), the number of their joint appearance with a known resistant-associated mutation, their associated lineage, and the method they ranked by.

Variants	Lineage	Appearance	Appearance in resistant isolates	Joint appearance	Ranked by
***embC*_R927R**	All lineages	7756	2101, 914, 1546, 870	2215	MLRF and SLRF
***embA*_C76C**	Animal and Central Asia	1630	955, 549, 848, 421	962	MLRF and SLRF
***rpoB*_A1075A**	All except for European	3678	1268, 599, 950, 540	1364	MLRF and SLRF
***embB*_G18G**	Central Asia, East Asia and European	26	8, 2, 4, 3	3	MLRF and SLRF

SUPPLEMENTARY G

We carried out the following experiments in order to confirm co-occurrence of resistance and the existence of additional resistance-associated mutations to those reported in the literature:

- known drug-resistant variants for a given drug extracted from F3 (e.g., for PZA, pncA and rpsA variants from F3);
- all variants in drug-associated genes for a given drug (e.g. for PZA, all variants in pncA and rpsA from F2); and
- all variants in F1.

Considering 1 and 2 allows us to quantify mutations that are not within the current library of known mutations but which are important in classifying resistance, while steps 2 and 3 would allow the identification of co-occurrence patterns. We analysed all features with an importance of more than 0.001 and found the following mutations as possible candidates. Only mutations that appeared in more than a lineage and that are dominant in resistant isolates are listed:

- Unknown mutations that might be important for classifying resistance: *embB*_G406C, *embB*_Y319C, *embA*_C-4T, *embR*_L217V, *embB*_L402V, *rpoB*_L430R, *pncA*_Y103C, *pncA*_A-11T, *pncA*_F94C, *pncA*_V44G, *pncA*_V155M, *pncA*_V9G, and *pncA*_T100P;
- Mutations that possibly represent the resistance co-occurrence patterns: *katG*_S315T, *rpoB*_S450L, *embB*_M306V, *rpoB*_D435V, *rpoB*_H445Y, *pncA*_H57D, *embB*_M306I, *embB*_Q497R, *rpoB*_H445D, *rpoB*_V170F, *rpoB*_S450W, *katG*_S315N, *pncA*_A-11G, *rpoB*_H445L, *embB*_D354A, *pncA*_Q10P, *rpoB*_D435Y, *embA*_C-12T, *rpoB*_H445R, *embB*_G406A, *fabG1*_G-17T, *pncA*_H57R, *embA*_C-16T, *pncA*_V7G, *embB*_G406S, and *rpoB*_H445C.

SUPPLEMENTARY H

Table S17. The number of resistant isolates remained after dropping isolates with any known resistant-associated mutation.

Drug	INH	EMB	RIF	PZA	FDR	MDR
F4	168	50	104	76	17	64
F5	169	50	104	174	17	64
